# Carbohydrate-Small Molecule Hybrids as Lead Compounds Targeting IL-6 Signaling

**DOI:** 10.3390/molecules28020677

**Published:** 2023-01-09

**Authors:** Daniel C. Schultz, Li Pan, Tiffany Wang, Conner Booker, Iram Hyder, Laura Hanold, Garret Rubin, Yousong Ding, Jiayuh Lin, Chenglong Li

**Affiliations:** 1 Department of Medicinal Chemistry, College of Pharmacy, The University of Florida, Gainesville, FL 32610, USA; 2 Department of Biochemistry and Molecular Biology, University of Maryland School of Medicine, Baltimore, MD 21201, USA

**Keywords:** Interleukin-6, structure-based drug design, docking, carbohydrate

## Abstract

In the past 25 years, a number of efforts have been made toward the development of small molecule interleukin-6 (IL-6) signaling inhibitors, but none have been approved to date. Monosaccharides are a diverse class of bioactive compounds, but thus far have been unexplored as a scaffold for small molecule IL-6-signaling inhibitor design. Therefore, in this present communication, we combined a structure-based drug design approach with carbohydrate building blocks to design and synthesize novel IL-6-signaling inhibitors targeting glycoprotein 130 (gp130). Of this series of compounds, **LS-TG-2P** and **LS-TF-3P** were the top lead compounds, displaying IC_50_ values of 6.9 and 16 µM against SUM159 cell lines, respectively, while still retaining preferential activity against the IL-6-signaling pathway. The carbohydrate moiety was found to improve activity, as *N*-unsubstituted triazole analogues of these compounds were found to be less active in vitro compared to the leads themselves. Thus, **LS-TG-2P** and **LS-TF-3P** are promising scaffolds for further development and study as IL-6-signaling inhibitors.

## 1. Introduction

**Figure 1:**
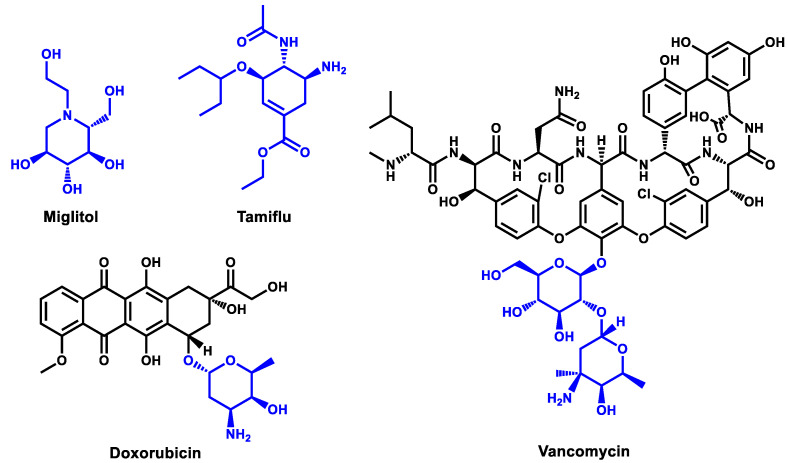
Examples of glycomimetic and carbohydrate-containing FDA-approved drugs, with glycomimetic and monosaccharide moieties highlighted in blue.

Carbohydrates are a class of compounds essential for numerous biological roles, such as communication, energy storage and metabolism, and protein and cell structure and function [1]. From 2000 to 2021, a total of 721 drugs have been approved by the FDA [2,3,4,5,6], but of these, only 54 were carbohydrate-containing entities [7]. Given that carbohydrate-containing drugs encompassed less than 10% of approvals during that time, it is evident that this chemical class is relatively unexplored when juxtaposed with their extensive role in biological processes [8,9]. Despite their low numbers, approved carbohydrate-based drugs have met with general success across a range of pathologies, such as viral and bacterial infections, cancer, and diabetes, with notable examples including oseltamivir (Tamiflu^TM^) [10], vancomycin [11], doxorubicin [12,13], and miglitol [14] (Figure 1).

While glycomimetics base their design around specific carbohydrates in order to mimic their function in vivo, many carbohydrate-containing drugs accommodate mono- and disaccharides in order to improve drug properties such as binding affinity, selectivity, and pharmacokinetics [8]. In the case of vancomycin, while its aglycon form has been shown to retain activity in vitro, the presence of its glucose and vancosamine moieties has been demonstrated to improve its in vivo properties and its ability to form homodimers [15,16]. For doxorubicin, its daunosamine unit has been shown to play a key role in its activity, improving the binding to and stability of its complex with DNA and topoisomerase II, and it has also been shown that structural modulation of this monosaccharide can affect the selectivity of the drug as a whole [8,17,18]. In general, the incorporation of carbohydrate or carbohydrate mimics into drug scaffolds can afford a number of advantages, such as improved aqueous solubility due to their innate hydrophilicity, higher likelihood of target interaction due to the diversity, density, and complexity of saccharide functional groups, and improved overall biocompatibility [8].

**Figure 2:**
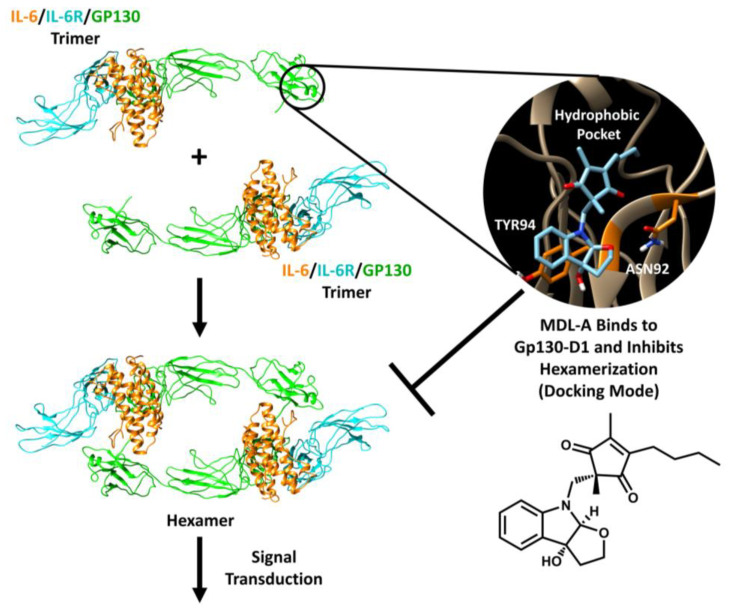
IL-6-signaling complex formation and signal transduction. MDL-A inhibits hexamer formation via binding to gp130-D1 (docking mode and structure shown on right). Signaling complex created from PDBID: 1P9M [25,48]. Docking mode recreated using AutoDock 4.2 [49] and visualized through Chimera [50].

Interleukin-6 is a key cytokine involved in the regulation of numerous processes within the body, including immune response, inflammatory response, and cell proliferation, and as a result of this role, its upregulation is associated with numerous disease states, such as multiple sclerosis, rheumatoid arthritis, and most types of cancer [19,20,21,22,23,24]. This cytokine acts through the formation of a hexameric complex on the surface of cells (Figure 2) [25]. To form this complex, IL-6 and its selective IL-6 receptor (IL-6R) form a dimer, which then binds to glycoprotein 130 (gp130), a key protein ubiquitously expressed on the surface cells in the body [25,26]. Signal transduction occurs when two of these trimers form a hexamer, which modulates the intracellular domain of gp130 and activates various signaling pathways within the cell, such as the JAK-STAT3 pathway [25,27]. Currently, several monoclonal-antibody-based inhibitors of IL-6 signaling have been approved for clinical use, but there are no approved small molecule agents at this time [28,29,30,31,32,33,34,35].

The first selective small-molecule inhibitors of IL-6 signaling were the natural product diastereomers madindoline A and B (MDL-A and MDL-B, respectively), which were isolated simultaneously in 1996 [36,37]. In later years, MDL-A was further studied in vitro and in silico, and it was determined to act via binding to the D1 domain of gp130, with several key interactions identified: hydrophobic interactions with a hydrophobic pocket, π–π interactions between its hydroxyfurindoline ring and TYR94, and hydrogen bonding with ASN92 [38,39,40]. Its docking mode has been recreated in Figure 2. Studies in vivo have also validated MDL-A’s status as a lead compound for drug development targeting IL-6 signaling [38,41]. Targeting IL-6 signaling via binding to gp130-D1 is particularly advantageous as the only cytokines within the IL-6 cytokine family that require gp130-D1 for signal complex formation are IL-6 and IL-11 [25,42].

**Figure 3:**
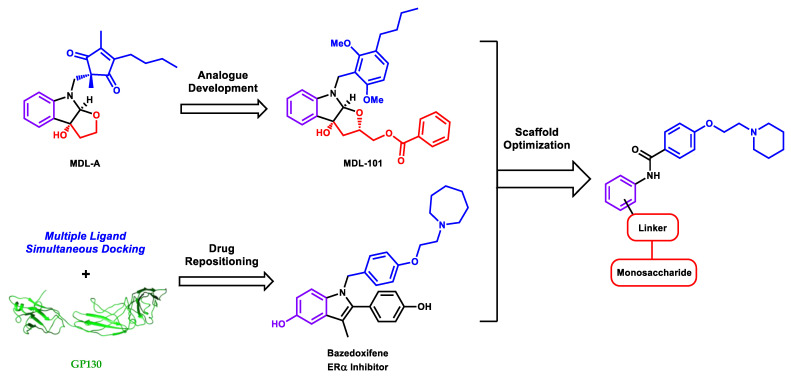
Development of a novel scaffold for IL-6 inhibitors through the combination of natural product-inspired analogue development and computer-aided drug repositioning of bazedoxifene. Key scaffold components are color coded. Blue indicates hydrophobic fragments for engaging with the gp130-D1 hydrophobic pocket, purple denotes rings which engage in π–π interactions with TYR94, and red denotes chains participating in hydrogen bonding with ASN92 and/or hydrophobic interactions with a shallow subpocket adjacent to ASN92.

Previously, we disclosed the IL-6-signaling inhibitor MDL-101 (Figure 3), which was discovered through an effort to develop more potent analogues of MDL-A [40,43,44]. This new lead was determined to be more potent than its natural product predecessor, and was notably found to suppress T helper type 17 cell development in vitro through the inhibition of IL-6 signaling [40,43,44]. This lead compound acts via binding to gp130-D1, inhibiting the formation of the IL-6/IL-6R/gp130 hexameric signaling complex [25], and docking studies have demonstrated that MDL-101 exhibits similar interactions with gp130-D1 as MDL-A, though it also possesses a benzoyl moiety that occupies a hydrophobic subpocket adjacent to ASN92 [40]. Despite its improved activity, however, this compound possessed less-than-ideal pharmacokinetic properties, which likely arises from similar metabolic liabilities as seen with MDL-A, which is readily metabolized into its 5-hydroxy analogue [44,45]. In order to circumvent these issues and also further improve potency, the use of conformationally adaptive monosaccharides was pursued as an alternative design strategy under the premise that their multiple free hydroxyls could improve ligand binding to the flat surface of gp130, both on a conformational basis and on the basis of maximizing hydrogen bonding with surface residues. By combining data from the structure–activity relationship of MDL analogues developed en route to MDL-101, along with our previous identification of bazedoxifene as a repurposed IL-6-signaling inhibitor through multiple ligand simultaneous docking [46,47], a general, synthetically accessible core scaffold was designed for subsequent modification with various linkers and carbohydrate units (Figure 3). In pursuit of this strategy, 15 novel, carbohydrate-containing compounds were developed and tested in vitro.

## 2. Results and Discussion

**Figure 4:**
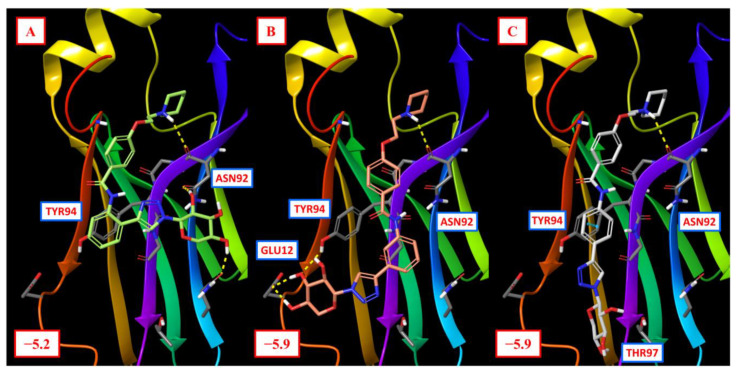
Glide SP docking results for monosaccharide-containing compounds against the D1 domain of gp130. (**A**) docking mode of **LS-TX-2**; (**B**) docking mode of **LS-TX-3**; (**C**) docking mode of **LS-TX-4**. Key residues are labeled, and hydrogen bonding and π–π interactions are denoted by dashed yellow lines and solid blue lines, respectively. Docking scores are in units of kcal/mol.

As hypothesized, initial docking studies of small molecule-carbohydrate hybrid compounds using GlideSP [51,52] suggested the formation of multiple hydrogen bonds between the monosaccharide hydroxyls and gp130-D1 (Figure 4).

Series of fructose-, glucose-, and xylose-containing compounds were then proposed. Inexpensive, commercially available carbohydrate building blocks were chosen for this study for ease of rapid analogue synthesis since the incorporation of custom monosaccharides could easily increase the number of steps to final product, thus increasing the time to overall completion, which runs counter to the immediate goal of design strategy validation. With regard to linking the monosaccharides to the parent scaffold, linkages that could be formed with robust chemistry that tolerates diverse functional groups and allows for great ease of parallel synthesis were desired. Therefore, it was determined to pursue monosaccharide building blocks containing azide and carboxylic acid functional groups for click chemistry and amide coupling, respectively.

**Scheme 1:**
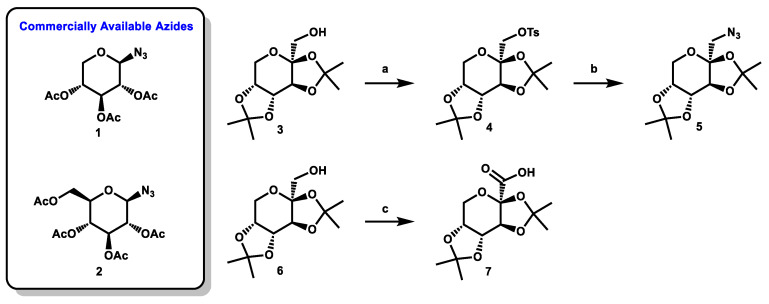
Commercially available and synthesized monosaccharide building blocks. Reagents and conditions: (a) TsCl (3.0 eq), TEA (2.0 eq), DMAP (1.0 eq), anh. CHCl_3_, rt, 48 h, 71%; (b) NaN_3_ (7.5 eq, in portions), DMF, 150 °C, 144 h, 48%; (c) TEMPO (0.2 eq.), BAIB (2.5 eq.), 4:4:1 DCM:^t^BuOH:H_2_O, rt, 4.75 h, 80%.

Two commercially available azide building blocks were purchased (Scheme 1), and a third azide building block, **5**, was synthesized from doubly acetonide-protected fructose, **3**. The primary alcohol of **3** was tosylated, and the substrate was then subjected to an S_N_2 reaction using sodium azide at high temperature (both procedures adapted from the literature [53,54]). A carboxylic acid building block was also synthesized from **3** using an adapted literature procedure [55] involving TEMPO and 2.5 equivalents of BAIB to afford the desired acid, **7**, in good yield.

**Scheme 2:**
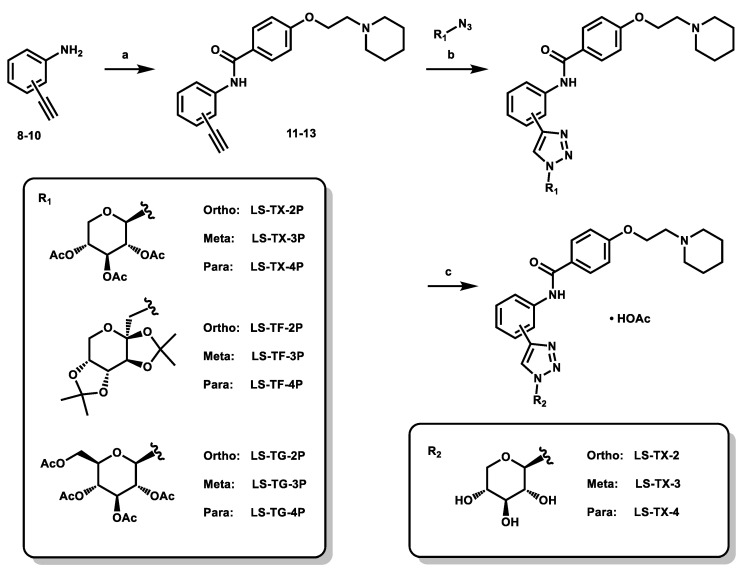
Synthesis of triazole-linked monosaccharide-small molecule hybrid compounds. Reagents and conditions: (a) 4-(2-(piperidin-1-yl)ethoxy)benzoyl chloride (1.5–2.6 eq), TEA (3.0–5.2 eq), DCM, rt or 0 °C-rt, 48.5–123 h, 46–88%; (b) Azide (1.0 eq), CuSO_4_ (2.0 eq), sodium ascorbate (4.0 eq), 10:2:1 DMF:THF:H_2_O, rt, overnight, 38–67%; (c) 0.5 M NaOMe (0.9 eq), MeOH, rt, 32–37 min, 26–65%.

With these building blocks in hand, a series of triazole-linked compounds were synthesized first (Scheme 2). The synthesis proceeded via amide coupling between *ortho*-, *meta*-, and *para*-ethynyl anilines **8**–**10** with 4-(2-(piperidin-1-yl)ethoxy)benzoyl chloride (**24**) to afford the desired amide intermediates **11**–**13** in moderate to good yield. These intermediates were then subjected to click chemistry using excess copper(II) sulfate to provide the desired triazole products in moderate yield. Deprotection of the xylose-based compounds via Zemplen deacetylation and subsequent purification via HPLC afforded the free-hydroxyl products in low to decent yield as acetate salts (**LS-TX-2**, **LS-TX-3**, and **LS-TX-4**).

Cytotoxicity assays were then pursued in SUM159 and MDA-MB-231 triple-negative breast cancer cell lines [56,57,58]. To our disappointment, however, these three xylose-containing compounds were found to be completely inactive in MTT assays against both of these cell lines at the tested concentrations (Table 1). It was then postulated that the free hydroxyls do not favorably interact with the surface of gp130-D1 as previously presumed. Drawing from previous literature and our experience with incorporation of hydrophobic moieties leading to increased activity against this target (as with MDL-101) [40,44], it was suggested that the intermediates containing protected carbohydrates could possess improved activity. Indeed, when these protected intermediates were tested in the same MTT assay conditions, activity was restored against both cell lines (Table 1). In this short series of analogues, there are some general trends that are observed, both with respect to substitution pattern and monosaccharide protecting group choice. For the protected xylose- and glucose-containing analogues, the *ortho* and *para*-substituted compounds exhibited improved activity compared to their respective *meta* regioisomers. Indeed, the glucose-containing compounds are the most potent of this series, with compounds **LS-TG-2P** and **LS-TG-4P** exhibiting 6.9 and 2.5 µM activity in SUM159 cells, respectively. Among the protected fructose-containing compounds, which contain acetonide-protecting groups instead of acetyl groups, the *meta* regioisomer, **LS-TF-3P**, exhibited the best activity with an IC_50_ of 16 µM in SUM159 cells. While the stereochemical arrangement of the hydroxyls of the fructose unit differs from those of xylose and glucose, it is likely that the primary driving force in the differentiation of activity patterns among regioisomers is due to the acetonide-protecting group, which forces the fructose carbohydrate unit to adopt a more rigid and bulky conformation compared its analogous xylose and glucose compounds. Additionally, despite the increase in lipophilicity due to the incorporation of protecting groups, the predicted logP values for these compounds still remain under the threshold of 5 as set by Lipinski’s Rule of 5 for oral bioavailability [59].

**Scheme 3:**
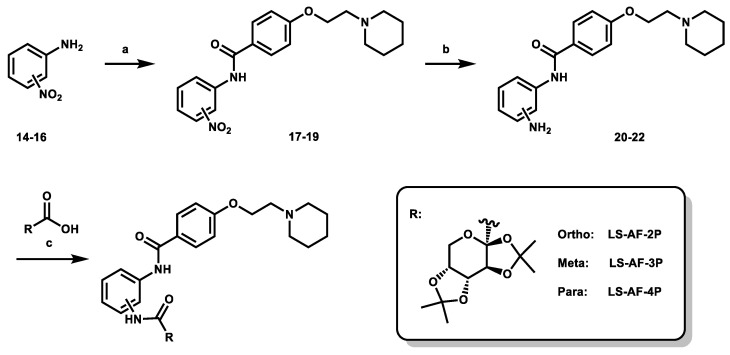
Synthesis of amide-linked fructose-small molecule hybrid compounds. Reagents and conditions: (a) 4-(2-(piperidin-1-yl)ethoxy)benzoyl chloride (1.5 eq), TEA (3.0 eq), DCM, rt, overnight, 69–93%; (b) H_2_, Pd/C (0.05 eq Pd), MeOH, rt, overnight, 64–80%; (c) 1. Carboxylic acid (1.0 eq), *N*-methyl imidazole (2.5 eq), DCM, 0 °C, 10 min; 2. MsCl (1.1 eq), DCM, 0 °C, 30 min; 3. Aniline (1.0 eq), DCM, 0 °C-rt, overnight, 12–55%.

Given that retaining protecting groups led to the recovery of activity, it was then desired to probe the effect of linker types on in vitro efficacy. A series of amide-linked fructose-containing regioisomers were then synthesized (Scheme 3). Starting with commercially available *ortho*-, *meta*-, and *para*-nitroanilines **14**–**16**, amide coupling was conducted with 4-(2-(piperidin-1-yl)ethoxy)benzoyl chloride (**24**) to afford the desired products **17**–**19** in moderate to good yield. Subsequent hydrogen over palladium reduction of the nitro groups afforded the desired aniline intermediates **20**–**22**, which were then subjected to amide coupling with fructose derivative **7** using methanesulfonyl chloride and *N*-methyl imidazole (procedure adapted from the literature [60]) to afford the final products in low to decent yield. While these three compounds also exhibited activity in MTT assays (see Table 1), it was apparent that these amide-linked scaffolds were less active compared to their triazole-linked analogues, save for the performance of **LS-AF-4P** against SUM159 cells. It is noted, though, that these amide-linked compounds displayed a different pattern of activity compared to the triazole-linked fructose compounds, **LS-TF-2P**, **3P**, and **4P**, in that the *para*-substituted amide-linked analogue, **LS-AF-4P**, was the most active, with an IC_50_ of 18.1 µM against SUM159 cells. Given the overall worse performance in vitro compared to the triazole-linked analogues, however, further exploration of carbohydrate diversity within the amide-linked series was not pursued.

**Scheme 4:**
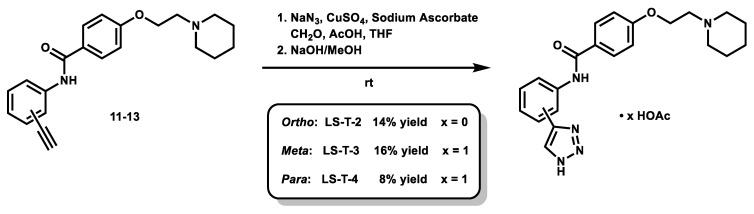
Synthesis of *N*-unsubstituted triazole compounds.

As a means of assessing the effect of incorporating carbohydrates on in vitro activity, three *N*-unsubstituted triazole compounds were synthesized using a modified click reaction adapted from the literature (Scheme 4) [61,62]. In this procedure, alkynes **11**–**13** were reacted with azidomethanol (formed in situ from sodium azide and formaldehyde) under mildly acidic click conditions, after which the hydroxymethyl group was removed under basic conditions to afford the desired *N*-unsubstituted triazoles **LS-T-2**, **LS-T-3**, and **LS-T-4** in low yield (Note: Compounds **LS-T-3** and **LS-T-4** were purified via HPLC to afford their acetate salt). These compounds were then subjected to MTT assays (Table 2). When controlled for substitution pattern, these compounds were generally less potent compared to their carbohydrate-containing companions in SUM159 cell lines. Key exceptions to this rule include *ortho* analogues **LS-TF-2P** and **LS-AF-2P**, which were weaker than **LS-T-2**, as well as *para* analogues **LS-TF-4P** and **LS-AF-4P**, which were weaker than **LS-T-4**. Unexpectedly, however, among all compounds disclosed herein, only **LS-TG-2P** exhibited better activity against MDA-MB-231 cells than **LS-T-4**.

With these in vitro results in hand, two compounds, **LS-TF-3P** and **LS-TG-2P**, were chosen for further analysis. **LS-TG-4P** was not chosen as **LS-TG-2P** performed better against MDA-MB-231 cells, and the former also tended to plateau in cell viability assays rather than kill one-hundred percent of cells. These two lead compounds were then assayed against PC3 and LNCaP prostate cancer cell lines (CCK-8 assay). It was anticipated that these compounds would display different activity against PC3 cells compared to LNCaP cells since the former produces IL-6, while the latter does not [63]. The results of viability assays against these cells are shown in Table 3. Similar to the results seen in the breast cancer MTT data, **LS-TG-2P** was found to be comparably active to the positive control, **bazedoxifene**, while **LS-TF-3P** was shown to be less potent. As anticipated, both **bazedoxifene** and **LS-TF-3P** exhibited less potent activity in LNCaP cell lines compared to their PC3 results. While the former has been reported to have no antiproliferative effect in LNCaP cell lines, it was tested at much lower concentrations than in the present study [64]. **LS-TG-2P**, however, had nearly equivalent activity in both prostate cancer cell lines. In both MTT and CCK-8 assays, this compound was observed to form aggregates at high concentrations, so it is possible that part of its cytotoxicity at higher doses is due to these aggregates rather than its binding to gp130-D1.

**Figure 5:**
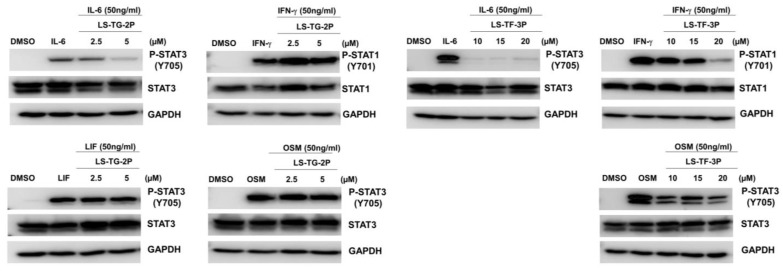
Cytokine selectivity assay Western Blot data. T47D cells were treated with **LS-TG-2P** or **LS-TF-3P** for 2 h, then stimulated with IL-6, IFN-γ, LIF, or OSM.

Cytokine selectivity assays were then conducted in order to validate the selective inhibition of IL-6 signaling. Monitoring downstream STAT3 or STAT1 phosphorylation provides a quantitative assessment of the inhibition of each of the indicated signaling pathways. While LIF and OSM are members of the IL-6 cytokine family, they do not require a hexameric assembly containing two gp130 units for signaling, and therefore their signaling should not be inhibited by compounds targeting gp130-D1 [65]. IFN-γ, however, does not signal via gp130, and is included to better monitor off-target activity. While not a direct means of assessing ligand-gp130-D1 binding, this provides a good determination of selectivity for this target as only IL-6 and IL-11 require binding to the D1 domain of gp130 to facilitate signaling [25,42]. The results of these assays can be seen in Figure 5. From these data, it is evident that both **LS-TG-2P** and **LS-TF-3P** selectively inhibit IL-6 signaling at lower concentrations. The latter exhibits some off-target effects at higher concentrations, but this provides a good springboard for further lead development.

**Figure 6:**
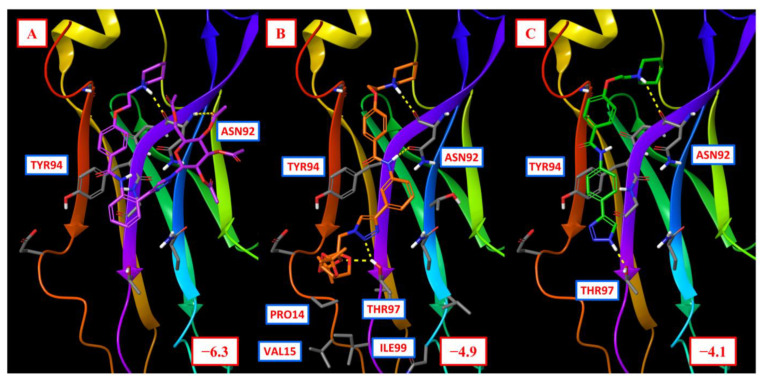
Glide SP docking results for lead carbohydrate-containing compounds against gp130-D1. (**A**) docking mode of **LS-TG-2P**; (**B**) docking mode of **LS-TF-3P**; (**C**) docking mode of **LS-T-4**. Docking scores are in units of kcal/mol. Key residues are indicated, and hydrogen bonding is shown by dotted yellow lines.

These two lead compounds were docked to gp130-D1 using GlideSP, along with **LS-T-4**, in order to better rationalize their activity (Figure 6). While the docking scores are not significantly different from the compounds discussed in Figure 4, some conclusions are able to be drawn from the proposed binding modes. For **LS-TG-2P**, the acetyl groups afford additional hydrogen bonding with ASN92. **LS-TF-3P**, however, exhibits hydrogen bonding between THR97 and both its monosaccharide and its triazole moieties. Several hydrophobic residues are adjacent to THR97 (PRO14, VAL15, and ILE99), and this could form a small hydrophobic patch that favorably interacts with its acetonide-protecting groups as well. Finally, **LS-T-4** displays a favorable binding mode, exhibiting key aromatic interactions with TYR94 as well as hydrogen bonding with THR97. The scores of these three compounds do not correlate well with activity, however, so further modelling is warranted to better understand the relationship between various functionalities of this class of compounds and activity, which would prompt better-directed analogue design.

Overall, carbohydrate incorporation into an optimized IL-6-signaling inhibitor scaffold was pursued as an alternative means of improving compound activity, leading to the development of lead compounds **LS-TG-2P** and **LS-TF-3P**, which exhibited 6.9 and 16 µM activity, respectively, against SUM159 breast cancer cell lines in vitro. **LS-TG-2P** was demonstrated to have single-digit micromolar activity against prostate cancer cell lines as well, though its activity could be skewed by aggregation at higher concentrations. Cytokine selectivity assays indicate that both of these compounds act as selective inhibitors of IL-6 signaling at lower concentrations. Furthermore, these compounds were more active than their carbohydrate-deficient analogues, demonstrating the viability of this strategy as a means of improving compound activity against flat, difficult-to-target proteins such as gp130. Further tests of these lead compounds are ongoing in order to further validate their activity and mechanism of action, as are drug development efforts focused on improving their drug properties and further increasing their potency.

## 3. Materials and Methods

### 3.1. Chemistry—General Information

All reactions were carried out under air unless stated otherwise. Reagents and solvents were purchased from commercial vendors and used without further purification. All reactions were monitored using thin layer chromatography (silica gel 60 F254 pre-coated aluminum plates). For non-UV active compounds, visualization of TLC spots was conducted using a 5% H_2_SO_4_ in ethanol stain. Purification was conducted using automated flash column chromatography (Biotage Isolera One Purification System) or High-Performance Liquid Chromatography (Dionex UltiMate 3000 with a pump and DAD-3000 in-line Diode Array Detector; reverse-phase C18 Luna, 5 µM, 100 Å, 250 × 21.2 mm column).

NMR spectra were obtained using the Bruker Avance NEO-600 (^1^H NMR: 600 MHz; ^13^C NMR: 151 MHz). All spectra are visualized using MestReNova 11.0, and all structures shown were drawn using ChemDraw 18.1. The following solvents were used for obtaining spectral data, and their corresponding reference peaks are shown here: CDCl_3_ (^1^H NMR: 7.26 ppm; ^13^C NMR: 77.16 ppm) and DMSO-*d6* (^1^H NMR: 2.50 ppm, ^13^C NMR: 39.52 ppm). All peaks were referenced either to these solvent peaks or to TMS (^1^H NMR: 0.00 ppm, ^13^C NMR: 0.00 ppm). All NMR experiments were conducted at room temperature. For ^1^H NMR, multiplicities are reported as follows: s = singlet, d = doublet, t = triplet, quint = quintet, and m = multiplet. Multiplets containing chemically inequivalent protons are further annotated as overlapping signals (os).

Low-resolution mass spectra for certain known compounds were obtained from the University of Florida Department of Medicinal Chemistry 3200 QTrap LC/MS/MS spectrometer via direct injection, and high-resolution mass spectra were obtained from the Mass Spectrometry Facility within the University of Florida Chemistry Department Agilent 6220 or Agilent 6230 Time-of-Flight Spectrometer with Electrospray Ionization or from the University of Florida Department of Medicinal Chemistry Thermo Scientific™ Q Exactive Focus mass spectrometer with Dionex™ Ultimate™ RSLC 3000 UHPLC system, equipped with H-ESI II probe on Ion Max API Source.

### 3.2. Chemistry—Synthesis and Characterization Data

**2,3;4,5-di-*O*-isopropylidene-1-*O*-tosyl-β-D-fructopyranose (4):** To an oven-dried vial was added 2,3;4,5-di-*O*-isopropylidene-β-D-fructopyranose (1.001 g, 3.85 mmol, 1.0 eq), *p*-toluenesulfonyl chloride (2.231 g, 11.70 mmol, 3.0 eq), and 4-(dimethylamino)pyridine (462.5 mg, 3.79 mmol, 1.0 eq). The reagents were then dissolved in 8 mL anhydrous CHCl_3_. Triethylamine (1.07 mL, 7.68 mmol, 2.0 eq) was added to the solution, which was then stirred at room temperature for 48 h. The reaction solution was then diluted with 30 mL DCM and washed with 30 mL portions of H_2_O, saturated NH_4_Cl, and brine. The solvent was removed via rotovap to afford a crude red oil. The crude material was then dry loaded onto silica gel and purified via automated flash column chromatography using a gradient of 0–25% EtOAc in hexanes to afford the desired product as a yellow translucent oil (1.13 g, 2.74 mmol, 71%).


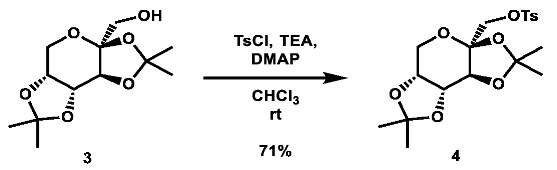


**^1^H NMR:** (600 MHz, CDCl_3_) δ 7.75 (m, 2H), 7.31 (m, 2H), 4.52 (dd, *J* = 7.9, 2.6 Hz, 1H), 4.25 (d, *J* = 2.6 Hz, 1H), 4.16 (dd, *J* = 7.9, 1.1 Hz, 1H), 4.02 (d, *J* = 10.3 Hz, 1H), 3.98 (d, *J* = 10.3 Hz, 1H), 3.82 (dd, *J* = 13.0, 1.8 Hz, 1H), 3.66 (d, *J* = 13.0 Hz, 1H), 2.40 (s, 3H), 1.46 (s, 3H), 1.32 (s, 3H), 1.32 (s, 3H), 1.27 (s, 3H).

**^13^C NMR:** (151 MHz, CDCl_3_) δ 145.0, 132.5, 129.9, 128.1, 109.2, 109.0, 100.7, 70.6, 70.0, 69.9, 69.2, 61.3, 26.5, 25.7, 25.2, 24.0, 21.6.

**LRMS (ESI):** calc. for C_19_H_27_O_8_S [M + H]^+^: 415, found: 415.

Note: ^1^H and ^13^C NMR data in accordance with the literature [53].


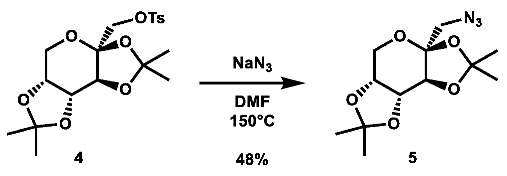


**1-azido-1-deoxy-2,3:4,5-di-*O*-isopropylidene-β-D-fructopyranose (5):** 2,3;4,5-di-*O*-isopropylidene-1-*O*-tosyl-β-D-fructopyranose (502.0 mg, 1.21 mmol, 1.0 eq) was added to a vial and dissolved in 6 mL DMF, after which sodium azide (395.1 mg, 6.08 mmol, 5.0 eq) was added. The mixture was then heated to 150 °C overnight. After two days, additional sodium azide (193.7 mg, 2.98 mmol, 2.5 eq) in 1 mL DMF was added. After four more days, the reaction was quenched with 50 mL brine and extracted with 3 × 100 mL portions of DCM. The organic layers were combined and rotovaped to afford a red oil. The crude material was then purified via automated flash column chromatography using a gradient of 0–30% EtOAc in hexanes to afford the desired compound as an off-white solid (166.2 mg, 0.583 mmol, 48%).

**^1^H NMR:** (600 MHz, CDCl_3_) δ 4.60 (dd, *J* = 7.9, 2.6 Hz, 1H), 4.28 (d, *J* = 2.7 Hz, 1H), 4.22 (dd, *J* = 7.9, 1.2 Hz, 1H), 3.91 (dd, *J* = 13.0, 1.9 Hz, 1H), 3.76 (dd, *J* = 13.0, 0.5 Hz, 1H), 3.58 (d, *J* = 13.0 Hz, 1H), 3.26 (d, *J* = 13.0 Hz, 1H), 1.55 (s, 3H), 1.48 (s, 3H), 1.46 (s, 3H), 1.33 (s, 3H).

**^13^C NMR:** (151 MHz, CDCl_3_) δ 109.3, 109.2, 102.8, 70.88, 70.87, 70.2, 61.2, 55.6, 26.7, 26.0, 24.9, 24.1.

**LRMS (ESI):** calc. for C_12_H_20_N_3_O_5_ [M + H]^+^: 286, found: 286.

Note: ^1^H NMR data in accordance with the literature [66]. No ^13^C data reported in the literature.


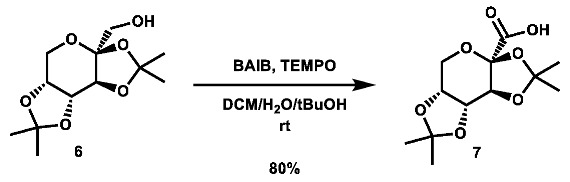


**2,3:4,5-di-*O*-isopropylidene-2-oxo-D-gluconic acid (7):** To a 50 mL flask was added 2,3;4,5-di-*O*-isopropylidene-β-D-fructopyranose (499.6 mg, 1.919 mmol, 1.0 eq), TEMPO (59.6 mg, 0.381 mmol, 0.2 eq), and bis(acetoxy) iodobenzene (1.5488 g, 4.808 mmol, 2.5 eq), which were then suspended in 13.5 mL 4:4:1 DCM:^t^BuOH:H_2_O. The mixture was stirred vigorously for 4.75 h, after which it was quenched with 70 mL 10 wt.% Na_2_S_2_O_3_ in H_2_O and extracted with 2 × 75 mL portions of EtOAc. The organic layers were combined, dried over anh. Na_2_SO_4_, and concentrated. The crude material was purified via automated flash column chromatography using a gradient of 89:10:1 to 29:70:1 Hex:EtOAc:AcOH to afford the desired product as a yellow-orange residue (420.2 mg, 1.532 mmol, 80%).

**^1^H NMR:** (600 MHz, CDCl_3_) δ 4.66–4.62 (m, 2H, os), 4.27 (d, *J* = 7.4 Hz, 1H), 3.99–3.89 (m, 2H, os), 1.57 (s, 3H), 1.53 (s, 3H), 1.46 (s, 3H), 1.35 (s, 3H).

**^13^C NMR:** (151 MHz, CDCl_3_) δ 168.2, 111.6, 109.6, 98.9, 73.1, 70.1, 69.8, 62.1, 26.3, 25.9, 24.5, 24.0.

Note: ^1^H NMR data in accordance with the literature [67]. No ^13^C NMR data have been reported.


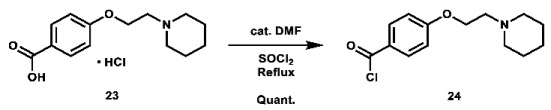


**General Procedure for the Preparation of 4-(2-piperidin-1-yl)ethoxy)benzoyl chloride (24):** To an oven-dried 50 mL round bottom flask was added 4-(2-piperidin-1-yl)ethoxy)benzoic acid hydrochloride (1.8269 g, 6.393 mmol, 1.0 eq), which was suspended in 2.5 mL thionyl chloride. Anhydrous DMF (3 drops, catalytic) was added, and the mixture was refluxed for 2 h. The solvent was then evaporated to afford the desired product as a pale yellow solid in quantitative yield, which was taken directly to the next step.


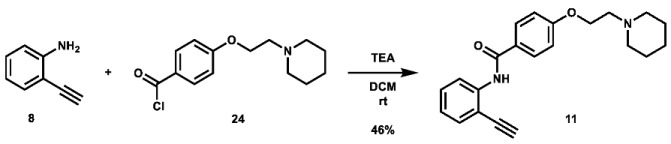


***N*-(2-ethynylphenyl)-4-(2-(piperidin-1-yl)ethoxy)benzamide (11):** To an oven-dried 100 mL round bottom flask was added 2-ethynyl aniline (0.28 mL, 2.462 mmol, 1.0 eq), which was dissolved in 15 mL DCM. Triethylamine (1.8 mL, 12.91 mmol, 5.2 eq) was added, followed by a solution of 4-(2-(piperidin-1-yl)ethoxy)benzoyl chloride (1.7149 g, 6.405 mmol, 2.6 eq) in 15 mL DCM. The solution was stirred at room temperature overnight. After 123 h, the reaction was quenched with 10 mL H_2_O and extracted with 180 mL DCM. The organic layer was separated and concentrated. The crude material was purified via automated flash column chromatography using a gradient of 92:6:2 to 38:60:2 Hex:EtOAc:TEA and then recrystallized from MeOH to afford the desired product as an off-white crystalline solid (394.6 mg, 1.132 mmol, 46%).

**^1^H NMR:** (600 MHz, CDCl_3_) δ 8.71 (s, 1H), 8.59 (dd, *J* = 8.4, 1.1 Hz, 1H), 7.92–7.81 (m, 2H), 7.50 (dd, *J* = 7.7, 1.6 Hz, 1H), 7.45–7.38 (m, 1H), 7.07 (td, *J* = 7.6, 1.1 Hz, 1H), 7.03–6.96 (m, 2H), 4.17 (t, *J* = 6.0 Hz, 2H), 3.59 (s, 1H), 2.80 (t, *J* = 6.0 Hz, 2H), 2.62–2.41 (m, 4H), 1.62 (quint, *J* = 5.6 Hz, 4H), 1.50–1.39 (m, 2H).

**^13^C NMR:** (151 MHz, CDCl_3_) δ 164.8, 162.0, 140.0, 132.1, 130.4, 128.9, 127.0, 123.2, 119.2, 114.7, 110.8, 84.6, 79.6, 66.3, 57.8, 55.1, 26.0, 24.2.

**HRMS (ESI):** calc. for C_22_H_25_N_2_O_2_ [M + H]^+^: 349.1911, found: 349.1906.


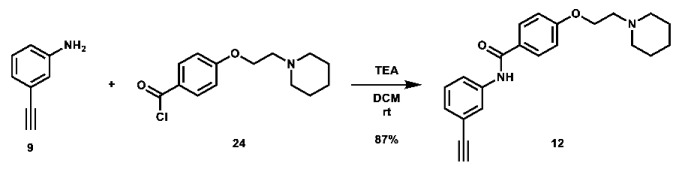


***N*-(3-ethynylphenyl)-4-(2-(piperidin-1-yl)ethoxy)benzamide (12):** To an oven-dried 100 mL round bottom flask was added 3-ethynyl aniline (0.45 mL, 4.302 mmol, 1.0 eq), 15 mL DCM, and TEA (1.81 mL, 12.986 mmol, 3.0 eq). The solution was then subjected to portionwise addition of a solution of 4-(2-(piperidin-1-yl)ethoxy)benzoyl chloride (1.7117 g, 6.393 mmol, 1.5 eq) in 25 mL DCM. After addition, the solution was stirred at room temperature overnight. After 48.5 h, the reaction was quenched with 20 mL H_2_O and extracted with 200 mL DCM. The organic layer was separated and concentrated. The crude material was purified via automated flash column chromatography using a gradient of 74:25:1 to 34:65:1 Hex:EtOAc:TEA to afford the desired product as a salmon-colored solid (1.3079 g, 3.753 mmol, 87%).

**^1^H NMR:** (600 MHz, CDCl_3_) δ 7.85–7.78 (m, 2H), 7.76–7.72 (m, 2H), 7.71–7.66 (m, 1H), 7.32 (t, *J* = 7.9 Hz, 1H), 7.27–7.25 (m, 1H), 7.00–6.94 (m, 2H), 4.17 (t, *J* = 6.0 Hz, 2H), 3.08 (s, 1H), 2.80 (t, *J* = 6.0 Hz, 2H), 2.61–2.40 (m, 4H), 1.62 (quint, *J* = 5.6 Hz, 4H), 1.51–1.42 (m, 2H).

**^13^C NMR:** (151 MHz, CDCl_3_) δ 165.2, 161.9, 138.1, 129.1, 128.9, 128.0, 126.8, 123.5, 122.9, 120.6, 114.6, 83.2, 77.5, 66.3, 57.8, 55.1, 25.9, 24.2.

**HRMS (ESI):** calc. for C_22_H_25_N_2_O_2_ [M + H]^+^: 349.1911, found: 349.1903.


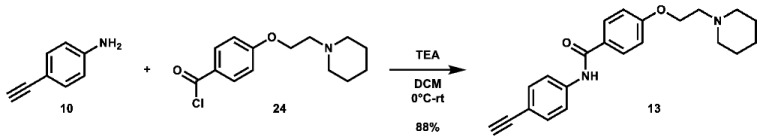


***N*-(4-ethynylphenyl)-4-(2-(piperidin-1-yl)ethoxy)benzamide (13):** To an oven-dried 100 mL round bottom flask was added 4-ethynyl aniline (502.6 mg, 4.290 mmol, 1.0 eq), which was dissolved in 15 mL DCM. The solution was cooled to 0 °C and TEA (1.81 mL, 12.986 mmol, 3.0 eq) was added, followed by portionwise addition of a solution of 4-(2-(piperidin-1-yl)ethoxy)benzoyl chloride (1.7144 g, 6.403 mmol, 1.5 eq) in 20 mL DCM. The solution was stirred overnight and allowed to gradually warm to room temperature. After 48.5 h, the reaction was quenched with 30 mL AQ K_2_CO_3_ and extracted with 200 mL DCM. The organic layer was separated and concentrated. The crude material was purified via automated flash column chromatography using a gradient of 75:24:1 to 29:70:1 Hex:EtOAc:TEA to afford the desired product as a light pink crystalline solid (1.3151 g, 3.774 mmol, 88%).

**^1^H NMR:** (600 MHz, CDCl_3_) δ 7.86–7.78 (m, 2H), 7.76 (s, 1H), 7.63–7.58 (m, 2H), 7.52–7.47 (m, 2H), 7.01–6.95 (m, 2H), 4.17 (t, *J* = 6.0 Hz, 2H), 3.06 (s, 1H), 2.80 (t, *J* = 6.0 Hz, 2H), 2.63–2.43 (m, 4H), 1.62 (quint, *J* = 5.6 Hz, 4H), 1.51–1.43 (m, 2H).

**^13^C NMR:** (151 MHz, CDCl_3_) δ 165.2, 162.1, 138.8, 133.2, 129.0, 126.9, 119.7, 117.8, 114.8, 83.6, 66.4, 57.9, 55.3, 26.1, 24.3.

**^13^C NMR:** (151 MHz, DMSO-*d6*) δ 165.0, 161.3, 140.0, 132.2, 129.7, 126.6, 120.0, 116.2, 114.2, 83.6, 79.9, 65.9, 57.3, 54.4, 25.6, 23.9.

**HRMS (ESI):** calc. for C_22_H_25_N_2_O_2_ [M + H]^+^: 349.1911, found: 349.1902.


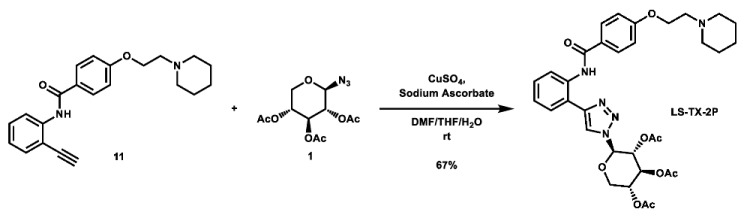


**(*2R,3R,4S,5R*)-2-(4-(2-(4-(2-(piperidin-1-yl)ethoxy)benzamido)phenyl)-1*H*-1,2,3-triazol-1-yl)tetrahydro-2*H*-pyran-3,4,5-triyl triacetate (LS-TX-2P):** To a vial was added *N*-(2-ethynylphenyl)-4-(2-(piperidin-1-yl)ethoxy)benzamide (133.3 mg, 0.383 mmol, 1.0 eq) and 2,3,4-tri-*O*-acetyl-β-D-xylopyranosyl azide (115.8 mg, 0.384 mmol, 1.0 eq), which were then dissolved in 6 mL DMF, 1.2 mL THF, and 0.6 mL H_2_O. To the solution was then added CuSO_4_ (122.3 mg, 0.766 mmol, 2.0 eq) and sodium ascorbate (462.2 mg, 2.333 mmol, 6.1 eq). The mixture was stirred at room temperature overnight. After 25 h, the reaction was stopped and the solvent was evaporated. The crude material was purified via automated flash column chromatography using a gradient of 90:8:2 to 0:98:2 Hex:EtOAc:TEA to afford the desired product as a pale yellow solid (165.7 mg, 0.255 mmol, 67%).

**^1^H NMR:** (600 MHz, CDCl_3_) δ 11.81 (s, 1H), 8.83 (dd, *J* = 8.4, 1.1 Hz, 1H), 8.13–8.04 (m, 3H), 7.52 (dd, *J* = 7.8, 1.5 Hz, 1H), 7.45–7.38 (m, 1H), 7.14 (td, *J* = 7.5, 1.2 Hz, 1H), 7.05–6.97 (m, 2H), 5.85 (d, *J* = 8.6 Hz, 1H), 5.49 (d, *J* = 9.3 Hz, 1H), 5.46 (t, *J* = 9.1 Hz, 1H), 5.20 (ddd, *J* = 10.0, 8.8, 5.6 Hz, 1H), 4.36 (dd, *J* = 11.7, 5.7 Hz, 1H), 4.18 (t, *J* = 6.0 Hz, 2H), 3.65 (dd, *J* = 11.7, 10.3 Hz, 1H), 2.81 (t, *J* = 6.0 Hz, 2H), 2.64–2.41 (m, 4H), 2.10 (s, 3H), 2.07 (s, 3H), 1.89 (s, 3H), 1.62 (quint, *J* = 5.7 Hz, 4H), 1.50–1.40 (m, 2H).

**^13^C NMR:** (151 MHz, CDCl_3_) δ 169.9, 169.7, 169.0, 165.4, 161.7, 148.5, 137.1, 129.7, 129.4, 127.5, 127.4, 123.4, 121.7, 119.1, 117.1, 114.5, 86.7, 71.9, 70.4, 68.3, 66.2, 65.7, 57.8, 55.1, 26.0, 24.2, 20.6, 20.6, 20.2.

**HRMS (ESI):** calc. for C_33_H_40_N_5_O_9_ [M + H]^+^: 650.2821, found: 650.2816.


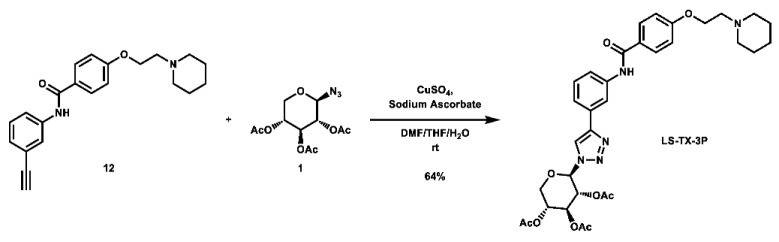


**(*2R,3R,4S,5R*)-2-(4-(3-(4-(2-(piperidin-1-yl)ethoxy)benzamido)phenyl)-1*H*-1,2,3-triazol-1-yl)tetrahydro-2*H*-pyran-3,4,5-triyl triacetate (LS-TX-3P):** To a vial was added *N*-(3-ethynylphenyl)-4-(2-(piperidin-1-yl)ethoxy)benzamide (134.6 mg, 0.386 mmol, 1.0 eq) and 2,3,4-tri-*O*-acetyl-β-D-xylopyranosyl azide (116.4 mg, 0.386 mmol, 1.0 eq), which were then dissolved in 6 mL DMF, 1.2 mL THF, and 0.6 mL H_2_O. The solution was stirred and CuSO_4_ (121.8 mg, 0.763 mmol, 2.0 eq) and sodium ascorbate (303.0 mg, 1.529 mmol, 4.0 eq) were added. The mixture was stirred vigorously at room temperature overnight. After 13.5 h, the reaction was complete and the solvent was evaporated. The crude material was twice purified via automated flash column chromatography, first with a 27:72:1 to 0:99:1 Hex:EtOAc:TEA gradient, and then with a 100:0 to 90:10 DCM:MeOH gradient to afford the desired product as a yellow solid (161.2 mg, 0.248 mmol, 64%).

**^1^H NMR:** (600 MHz, CDCl_3_) δ 8.08 (t, *J* = 1.9 Hz, 1H), 8.04 (s, 1H), 8.02 (s, 1H), 7.89–7.84 (m, 2H), 7.80 (m, 1H), 7.60 (dt, *J* = 7.7, 1.4 Hz, 1H), 7.43 (t, *J* = 7.9 Hz, 1H), 7.00–6.95 (m, 2H), 5.83 (m, 1H), 5.49–5.39 (m, 2H, os), 5.18 (m, 1H), 4.42–4.22 (m, 3H, os), 3.63 (dd, *J* = 11.7, 10.4 Hz, 1H), 3.07–2.95 (m, 2H), 2.94–2.56 (m, 4H), 2.09 (s, 3H), 2.07 (s, 3H), 1.90 (s, 3H), 1.82–1.74 (m, 4H), 1.57–1.49 (m, 2H).

**^13^C NMR:** (151 MHz, CDCl_3_) δ 169.9, 169.8, 169.0, 165.2, 161.2, 148.0, 138.8, 130.7, 129.7, 129.0, 127.4, 121.7, 120.2, 118.2, 117.3, 114.6, 86.4, 72.1, 70.4, 68.5, 65.6, 65.2, 57.2, 54.8, 24.8, 23.4, 20.7, 20.6, 20.2.

**HRMS (ESI):** calc. for C_33_H_40_N_5_O_9_ [M + H]^+^: 650.2821, found: 650.2807.


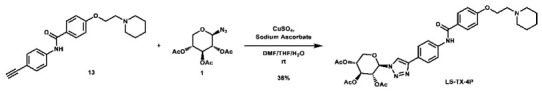


**(*2R,3R,4S,5R*)-2-(4-(4-(4-(2-(piperidin-1-yl)ethoxy)benzamido)phenyl)-1*H*-1,2,3-triazol-1-yl)tetrahydro-2*H*-pyran-3,4,5-triyl triacetate (LS-TX-4P):** To a vial was added *N*-(4-ethynylphenyl)-4-(2-(piperidin-1-yl)ethoxy)benzamide (133.0 mg, 0.382 mmol, 1.0 eq) and 2,3,4-tri-*O*-acetyl-β-D-xylopyranosyl azide (116.4 mg, 0.386 mmol, 1.0 eq), which were then dissolved in 6 mL DMF, 1.2 mL THF, and 0.6 mL H_2_O. To the solution was added CuSO_4_ (121.3 mg, 0.760 mmol, 2.0 eq) and sodium ascorbate (302.8 mg, 1.528 mmol, 4.0 eq). The mixture was stirred vigorously at room temperature overnight. After 13.5 h, the reaction was complete and the solvent was evaporated. The crude material was twice purified via automated flash column chromatography, first using a gradient of 24:75:1 to 0:99:1 Hex:EtOAc:TEA, then using a gradient of 100:0 to 90:10 DCM:MeOH to afford the desired product as a dark yellow solid (94.0 mg, 0.145 mmol, 38%).

**^1^H NMR:** (600 MHz, CDCl_3_) δ 7.97 (s, 1H), 7.95 (s, 1H), 7.88–7.84 (m, 2H), 7.85–7.81 (m, 2H), 7.77–7.72 (m, 2H), 7.00–6.95 (m, 2H), 5.84 (d, *J* = 8.6 Hz, 1H), 5.47 (t, *J* = 9.2 Hz, 1H), 5.44 (t, *J* = 9.6 Hz, 1H), 5.22–5.15 (m, 1H), 4.39–4.25 (m, 3H, os), 3.62 (dd, *J* = 11.6, 10.4 Hz, 1H), 3.06–2.93 (m, 2H), 2.88–2.61 (m, 4H), 2.09 (s, 3H), 2.06 (s, 3H), 1.90 (s, 3H), 1.81–1.74 (m, 4H), 1.56–1.49 (m, 2H).

**^13^C NMR:** (151 MHz, CDCl_3_) δ 169.9, 169.8, 169.1, 165.1, 161.0, 148.0, 138.5, 129.1, 127.6, 126.6, 126.0, 120.4, 117.4, 114.6, 86.4, 72.2, 70.4, 68.5, 65.6, 64.8, 57.1, 54.7, 24.5, 23.2, 20.7, 20.6, 20.3.

**HRMS (ESI):** calc. for C_33_H_40_N_5_O_9_ [M + H]^+^: 650.2821, found: 650.2809.


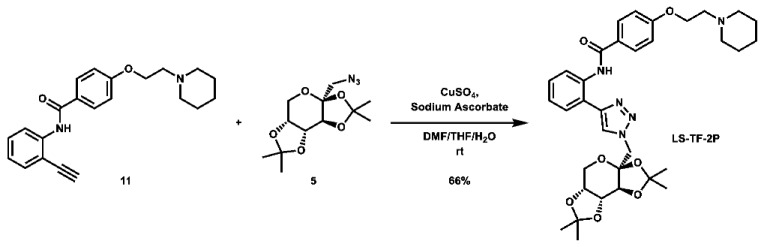


**4-(2-(piperidin-1-yl)ethoxy)-*N*-(2-(1-(((*3aS,5aR,8aR,8bS*)-2,2,7,7-tetramethyltetrahydro-3a*H*-bis**([1,3]**dioxolo)[4,5-*b*:4′,5′-d]pyran-3a-yl)methyl)-1*H*-1,2,3-triazol-4-yl)phenyl)benzamide (LS-TF-2P):** To a vial was added *N*-(2-ethynylphenyl)-4-(2-(piperidin-1-yl)ethoxy)benzamide (134.5 mg, 0.386 mmol, 1.0 eq) and 1-azido-1-deoxy-2,3;4,5-di-*O*-isopropylidene-β-D-fructose (108.3 mg, 0.380 mmol, 1.0 eq), which were then dissolved in 6 mL DMF, 1.2 mL THF, and 0.6 mL H_2_O. To the solution was then added CuSO_4_ (121.1 mg, 0.759 mmol, 2.0 eq) and sodium ascorbate (302.0 mg, 1.524 mmol, 4.0 eq). The mixture was stirred vigorously at room temperature overnight. Upon completion of the reaction, the solvent was evaporated. The crude material was twice purified via automated flash column chromatography, first using a gradient of 100:0 to 85:15 DCM:MeOH, then 90:9:1 to 40:59:1 Hex:EtOAc:TEA to afford the desired product as a yellow flaky solid (158.3 mg, 0.250 mmol, 66%).

**^1^H NMR:** (600 MHz, CDCl_3_) δ 12.01 (s, 1H), 8.84 (d, *J* = 8.5 Hz, 1H), 8.15–8.07 (m, 2H), 8.03 (s, 1H), 7.53 (dd, *J* = 7.8, 1.5 Hz, 1H), 7.42–7.36 (m, 1H), 7.15–7.10 (m, 1H), 7.05–6.99 (m, 2H), 4.83 (d, *J* = 14.4 Hz, 1H), 4.68 (dd, *J* = 7.8, 2.7 Hz, 1H), 4.64 (d, *J* = 14.4 Hz, 1H), 4.52 (d, *J* = 2.8 Hz, 1H), 4.27 (dd, *J* = 7.9, 1.7 Hz, 1H), 4.19 (t, *J* = 6.0 Hz, 2H), 3.95 (dd, *J* = 13.0, 1.9 Hz, 1H), 3.83 (d, *J* = 12.9 Hz, 1H), 2.82 (t, *J* = 6.0 Hz, 2H), 2.63–2.45 (m, 4H), 1.63 (quint, *J* = 5.7 Hz, 4H), 1.51 (s, 3H), 1.49 (s, 3H), 1.48–1.42 (m, 2H), 1.38 (s, 3H), 0.84 (s, 3H).

**^13^C NMR:** (151 MHz, CDCl_3_) δ 165.5, 161.6, 147.7, 137.0, 129.4, 129.2, 127.5, 127.1, 123.7, 123.2, 121.6, 117.6, 114.5, 109.7, 109.4, 100.7, 70.8, 70.5, 70.1, 66.1, 61.9, 57.8, 55.4, 55.1, 26.4, 26.0, 25.9, 24.4, 24.2, 24.0.

**HRMS (ESI):** calc. for C_34_H_44_N_5_O_7_ [M + H]^+^: 634.3241, found: 634.3259.


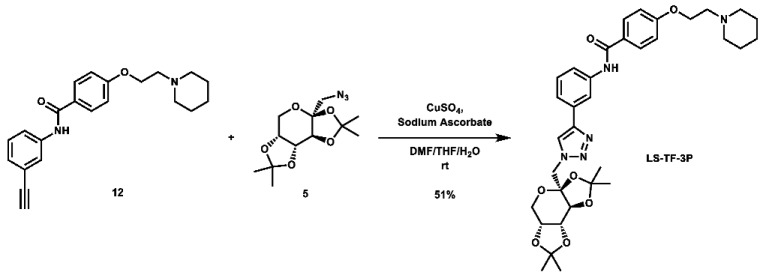


**4-(2-(piperidin-1-yl)ethoxy)-*N*-(3-(1-(((*3aS,5aR,8aR,8bS*)-2,2,7,7-tetramethyltetrahydro-3a*H*-bis**([1,3]**dioxolo)[4,5-*b*:4′,5′-d]pyran-3a-yl)methyl)-1*H*-1,2,3-triazol-4-yl)phenyl)benzamide (LS-TF-3P):** To a vial was added *N*-(3-ethynylphenyl)-4-(2-(piperidin-1-yl)ethoxy)benzamide (132.0 mg, 0.379 mmol, 1.0 eq) and 1-azido-1-deoxy-2,3;4,5-di-*O*-isopropylidene-β-D-fructose (108.9 mg, 0.382 mmol, 1.0 eq), which were then dissolved in 6 mL DMF, 1.2 mL THF, and 0.6 mL H_2_O. To the solution was then added CuSO_4_ (122.7 mg, 0.769 mmol, 2.0 eq) and sodium ascorbate (303.2 mg, 1.530 mmol, 4.0 eq). The mixture was stirred vigorously at room temperature overnight. After 22 h, the reaction was stopped and the solvent was evaporated. The crude material was twice purified via automated flash column chromatography, first using a gradient of 74:25:1 to 0:99:1 Hex:EtOAc:TEA, then 100:0 to 90:10 DCM:MeOH to afford the desired product as a yellow-orange foam (122.7 mg, 0.194 mmol, 51%).

**^1^H NMR:** (600 MHz, CDCl_3_) δ 8.11 (s, 1H), 8.09 (t, *J* = 1.9 Hz, 1H), 7.96 (s, 1H), 7.91–7.85 (m, 2H), 7.80–7.73 (m, 1H), 7.61 (dt, *J* = 7.7, 1.3 Hz, 1H), 7.42 (t, *J* = 7.9 Hz, 1H), 7.00–6.91 (m, 2H), 4.78 (d, *J* = 14.4 Hz, 1H), 4.65 (dd, *J* = 7.8, 2.7 Hz, 1H), 4.58 (d, *J* = 14.5 Hz, 1H), 4.52 (d, *J* = 2.7 Hz, 1H), 4.42–4.31 (m, 2H), 4.25 (dd, *J* = 7.8, 1.7 Hz, 1H), 3.93 (dd, *J* = 12.9, 1.9 Hz, 1H), 3.81 (d, *J* = 12.8 Hz, 1H), 3.13–3.00 (m, 2H), 2.98–2.66 (m, 4H), 1.88–1.75 (m, 4H), 1.58–1.52 (m, 2H), 1.50 (s, 3H), 1.48 (s, 3H), 1.36 (s, 3H), 0.84 (s, 3H).

**^13^C NMR:** (151 MHz, CDCl_3_) δ 165.2, 160.9, 147.2, 138.8, 131.4, 129.6, 129.1, 127.6, 122.8, 121.6, 119.9, 117.2, 114.6, 109.6, 109.3, 100.9, 70.8, 70.6, 70.1, 64.7, 61.9, 57.0, 55.2, 54.7, 26.4, 26.1, 24.3, 24.0, 23.1.

**HRMS (ESI):** calc. for C_34_H_44_N_5_O_7_ [M + H]^+^: 634.3241, found: 634.3231.


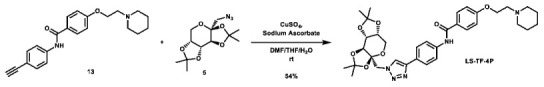


**4-(2-(piperidin-1-yl)ethoxy)-*N*-(4-(1-(((*3aS,5aR,8aR,8bS*)-2,2,7,7-tetramethyltetrahydro-3a*H*-bis**([1,3]**dioxolo)[4,5-*b*:4′,5′-d]pyran-3a-yl)methyl)-1*H*-1,2,3-triazol-4-yl)phenyl)benzamide (LS-TF-4P):** To a vial was added *N*-(4-ethynylphenyl)-4-(2-(piperidin-1-yl)ethoxy)benzamide (133.2 mg, 0.382 mmol, 1.0 eq) and 1-azido-1-deoxy-2,3;4,5-di-*O*-isopropylidene-β-D-fructose (110.1 mg, 0.386 mmol, 1.0 eq), which were then dissolved in 6 mL DMF, 1.2 mL THF, and 0.6 mL H_2_O. To the solution was then added CuSO_4_ (123.2 mg, 0.772 mmol, 2.0 eq) and sodium ascorbate (304.1 mg, 1.535 mmol, 4.0 eq). The mixture was stirred vigorously at room temperature overnight. Upon completion, the reaction was stopped and the solvent was evaporated. The crude material was twice purified via automated flash column chromatography, first using a gradient of 69:30:0:1 to 0:99:0:1 to 0:89:10:1 Hex:EtOAc:MeOH:TEA, then 100:0 to 90:10 DCM:MeOH to afford the desired product as a yellow-orange solid (141.1 mg, 0.223 mmol, 58%).

**^1^H NMR:** (600 MHz, CDCl_3_) δ 7.93 (s, 1H), 7.89 (s, 1H), 7.88–7.80 (m, 4H), 7.75–7.68 (m, 2H), 6.99–6.92 (m, 2H), 4.79 (d, *J* = 14.4 Hz, 1H), 4.65 (dd, *J* = 7.8, 2.7 Hz, 1H), 4.58 (d, *J* = 14.5 Hz, 1H), 4.53 (d, *J* = 2.7 Hz, 1H), 4.29–4.20 (m, 3H, os), 3.94 (dd, *J* = 12.9, 1.9 Hz, 1H), 3.81 (d, *J* = 12.9 Hz, 1H), 2.90 (t, *J* = 5.7 Hz, 2H), 2.72–2.56 (m, 4H), 1.75–1.63 (m, 4H), 1.53–1.45 (m, 2H), 1.49 (s, 3H), 1.49 (s, 3H), 1.36 (s, 3H), 0.85 (s, 3H).

**^13^C NMR:** (151 MHz, CDCl3) δ 165.5, 161.4, 147.2, 138.4, 129.2, 127.2, 126.4, 126.3, 122.3, 120.6, 114.4, 109.5, 109.3, 100.9, 70.8, 70.5, 70.1, 65.5, 61.9, 57.4, 55.2, 54.9, 26.4, 26.0, 25.3, 24.3, 24.0, 23.7.

**HRMS (ESI):** calc. for C_34_H_44_N_5_O_7_ [M + H]^+^: 634.3241, found: 634.3231.


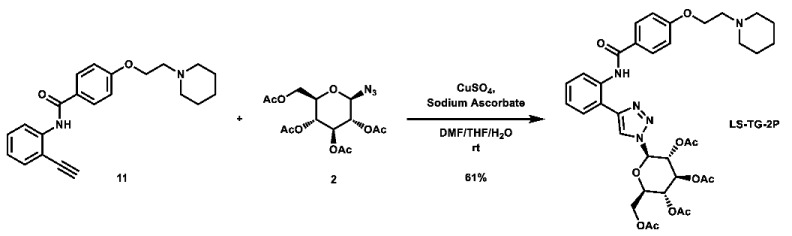


**(*2R,3R,4S,5R,6R*)-2-(acetoxymethyl)-6-(4-(2-(4-(2-(piperidin-1-yl)ethoxy)benzamido)phenyl)-1*H*-1,2,3-triazol-1-yl)tetrahydro-2*H*-pyran-3,4,5-triyl triacetate (LS-TG-2P):** To a vial was added *N*-(2-ethynylphenyl)-4-(2-(piperidin-1-yl)ethoxy)benzamide (117.5 mg, 0.337 mmol, 1.0 eq) and 1-azido-1-deoxy-β-D-glucose tetraacetate (126.4 mg, 0.339 mmol, 1.0 eq), which were then dissolved in 6 mL DMF, 1.2 mL THF, and 0.6 mL H_2_O. To the solution was then added CuSO_4_ (107.9 mg, 0.676 mmol, 2.0 eq) and sodium ascorbate (267.3 mg, 1.349 mmol, 4.0 eq). The mixture was stirred vigorously at room temperature overnight. Upon completion of the reaction, the solvent was evaporated. The crude material was purified via automated flash column chromatography, using a gradient of 69:30:1 to 0:99:1 Hex:EtOAc:TEA to afford the desired product as a yellow flaky solid (149.5 mg, 0.207 mmol, 61%).

**^1^H NMR:** (600 MHz, CDCl_3_) δ 11.80 (s, 1H), 8.83 (dd, *J* = 8.4, 1.2 Hz, 1H), 8.12 (s, 1H), 8.11–8.07 (m, 2H), N7.54 (dd, *J* = 7.8, 1.5 Hz, 1H), 7.42 (ddd, *J* = 8.5, 7.3, 1.6 Hz, 1H), 7.15 (td, *J* = 7.5, 1.2 Hz, 1H), 7.04–6.99 (m, 2H), 5.94 (d, *J* = 9.4 Hz, 1H), 5.55 (t, *J* = 9.5 Hz, 1H), 5.47 (t, *J* = 9.5 Hz, 1H), 5.29 (t, *J* = 9.6 Hz, 1H), 4.34 (dd, *J* = 12.7, 5.1 Hz, 1H), 4.21–4.14 (m, 3H, os), 4.06 (ddd, *J* = 10.2, 5.1, 2.2 Hz, 1H), 2.81 (t, *J* = 6.0 Hz, 2H), 2.61–2.44 (m, 4H), 2.09 (s, 3H), 2.09 (s, 3H), 2.05 (s, 3H), 1.88 (s, 3H), 1.62 (quint, *J* = 5.7 Hz, 4H), 1.50–1.42 (m, 2H).

**^13^C NMR:** (151 MHz, CDCl_3_) δ 170.5, 169.9, 169.3, 168.9, 165.4, 161.7, 148.5, 137.0, 129.7, 129.4, 127.5, 127.4, 123.4, 121.7, 119.3, 117.1, 114.5, 86.1, 75.4, 72.6, 70.3, 67.7, 66.2, 61.5, 57.8, 55.1, 25.9, 24.2, 20.7, 20.5, 20.5, 20.2.

**HRMS (ESI):** calc. for C_36_H_44_N_5_O_11_ [M + H]^+^: 722.3032, found: 722.3018.


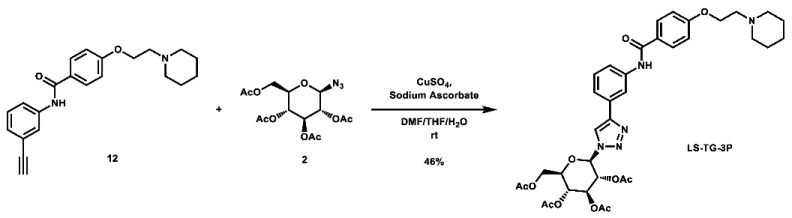


**(*2R,3R,4S,5R,6R*)-2-(acetoxymethyl)-6-(4-(3-(4-(2-(piperidin-1-yl)ethoxy)benzamido)phenyl)-1*H*-1,2,3-triazol-1-yl)tetrahydro-2*H*-pyran-3,4,5-triyl triacetate (LS-TG-3P):** To a vial was added *N*-(3-ethynylphenyl)-4-(2-(piperidin-1-yl)ethoxy)benzamide (118.3 mg, 0.340 mmol, 1.0 eq) and 1-azido-1-deoxy-β-D-glucose tetraacetate (126.4 mg, 0.339 mmol, 1.0 eq), which were then dissolved in 6 mL DMF, 1.2 mL THF, and 0.6 mL H_2_O. The solution was stirred and CuSO_4_ (110.0 mg, 0.689 mmol, 2.0 eq) and sodium ascorbate (269.8 mg, 1.362 mmol, 4.0 eq) were added. The mixture was stirred vigorously at room temperature overnight. Upon completion of the reaction, the solvent was evaporated. The crude material was twice purified via automated flash column chromatography, first with a 24:75:1 to 0:99:1 Hex:EtOAc:TEA gradient, and then with a 100:0 to 90:10 DCM:MeOH gradient to afford the desired product as a light orange solid (113.5 mg, 0.157 mmol, 46%).

**^1^H NMR:** (600 MHz, CDCl_3_) δ 8.10 (t, *J* = 1.9 Hz, 1H), 8.07 (s, 1H), 7.94 (s, 1H), 7.89–7.84 (m, 2H), 7.78–7.73 (m, 1H), 7.62 (dt, *J* = 8.0, 1.5 Hz, 1H), 7.44 (t, *J* = 7.9 Hz, 1H), 7.02–6.96 (m, 2H), 5.92 (d, *J* = 9.2 Hz, 1H), 5.51 (t, *J* = 9.4 Hz, 1H), 5.45 (t, *J* = 9.4 Hz, 1H), 5.27 (dd, *J* = 10.1, 9.2 Hz, 1H), 4.38–4.28 (m, 3H, os), 4.17 (dd, *J* = 12.6, 2.1 Hz, 1H), 4.03 (ddd, *J* = 10.1, 5.0, 2.1 Hz, 1H), 3.09–2.90 (m, 2H), 2.91–2.56 (m, 4H), 2.10 (s, 3H), 2.08 (s, 3H), 2.04 (s, 3H), 1.89 (s, 3H), 1.81–1.73 (m, 4H), 1.56–1.51 (m, 2H).

**^13^C NMR:** (151 MHz, CDCl_3_) δ 170.6, 170.0, 169.4, 169.0, 165.2, 161.3, 148.1, 138.8, 130.7, 129.7, 129.0, 127.4, 121.7, 120.2, 118.3, 117.4, 114.6, 85.9, 75.2, 72.7, 70.3, 67.7, 65.2, 61.6, 57.3, 54.8, 24.9, 23.4, 20.7, 20.6, 20.6, 20.2.

**HRMS (ESI):** calc. for C_36_H_44_N_5_O_11_ [M + H]^+^: 722.3032, found: 722.3020.


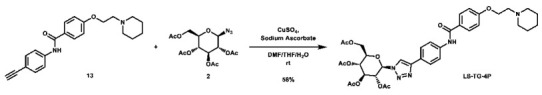


**(*2R,3R,4S,5R,6R*)-2-(acetoxymethyl)-6-(4-(4-(4-(2-(piperidin-1-yl)ethoxy)benzamido)phenyl)-1*H*-1,2,3-triazol-1-yl)tetrahydro-2*H*-pyran-3,4,5-triyl triacetate (LS-TG-4P):** To a vial was added *N*-(4-ethynylphenyl)-4-(2-(piperidin-1-yl)ethoxy)benzamide (117.4 mg, 0.337 mmol, 1.0 eq) and 1-azido-1-deoxy-β-D-glucose tetraacetate (125.3 mg, 0.336 mmol, 1.0 eq), which were then dissolved in 6 mL DMF, 1.2 mL THF, and 0.6 mL H_2_O. The solution was stirred and CuSO_4_ (108.2 mg, 0.678 mmol, 2.0 eq) and sodium ascorbate (266.4 mg, 1.345 mmol, 4.0 eq) were added. The mixture was stirred vigorously at room temperature overnight. Upon completion of the reaction, the solvent was evaporated. The crude material was twice purified via automated flash column chromatography with a gradient of 24:75:0:1 to 0:99:0:1 to 0:94:5:1 Hex:EtOAc:MeOH:TEA gradient to afford the desired product as an off-white solid (140.9 mg, 0.195 mmol, 58%).

**^1^H NMR:** (600 MHz, CDCl_3_) δ 7.99 (s, 1H), 7.87–7.83 (m, 4H), 7.82 (s, 1H), 7.75–7.71 (m, 2H), 7.02–6.97 (m, 2H), 5.93 (d, *J* = 9.4 Hz, 1H), 5.53 (t, *J* = 9.5 Hz, 1H), 5.45 (t, *J* = 9.4 Hz, 1H), 5.28 (dd, *J* = 10.2, 9.3 Hz, 1H), 4.33 (dd, *J* = 12.7, 5.1 Hz, 1H), 4.30–4.20 (m, 2H), 4.17 (dd, *J* = 12.7, 2.2 Hz, 1H), 4.03 (ddd, *J* = 10.2, 5.1, 2.2 Hz, 1H), 2.96–2.84 (m, 2H), 2.80–2.46 (m, 4H), 2.10 (s, 3H), 2.08 (s, 3H), 2.05 (s, 3H), 1.90 (s, 3H), 1.74–1.66 (m, 4H), 1.52–1.48 (m, 2H).

**^13^C NMR:** (151 MHz, CDCl_3_) δ 170.5, 169.9, 169.4, 169.0, 165.1, 161.6, 148.1, 138.4, 128.9, 127.2, 126.7, 125.9, 120.3, 117.4, 114.6, 85.8, 75.2, 72.8, 70.2, 67.7, 61.6, 57.5, 55.0, 25.4, 23.8, 20.7, 20.6, 20.5, 20.2. Note: ^13^C signal missing for CH_2_ vicinal to phenolic oxygen, but clear correlation to a carbon at 65.8 ppm can be seen via HSQC (see Supplementary Materials).

**HRMS (ESI):** calc. for C_36_H_44_N_5_O_11_ [M + H]^+^: 722.3032, found: 722.3020.


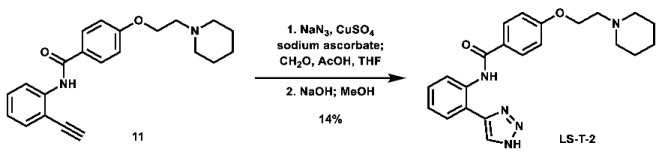


***N*-(2-(1*H*-1,2,3-triazol-4-yl)phenyl)-4-(2-(piperidin-1-yl)ethoxy)benzamide (LS-T-2):** To a vial was added formaldehyde (37 wt.% in H_2_O, 0.19 mL, 2.55 mmol, 11.0 eq), acetic acid (0.03 mL, 0.524 mmol, 2.3 eq), and 1.5 mL THF. The solution was stirred for 15 min. To the solution was added sodium azide (26.2 mg, 0.403 mmol, 1.7 eq) and a solution of *N*-(2-ethynylphenyl)-4-(2-(piperidin-1-yl)ethoxy)benzamide (80.8 mg, 0.231 mmol, 1.0 eq) in 1.5 mL THF. The mixture was stirred for 10 min. To the mixture was added a solution of CuSO_4_ (5.0 mg, 31.3 μmol, 0.14 eq) in 0.5 mL H_2_O, followed by sodium ascorbate (26.0 mg, 0.131 mmol, 0.57 eq). The mixture was stirred at room temperature overnight. After 12 h, the solvent was removed via rotovap. The residue was then dissolved in 3 mL MeOH and 2N NaOH (1.0 mL, 2.0 mmol, 8.7 eq) and stirred at room temperature for 6 h. Upon completion, the mixture was filtered and concentrated. The crude material was purified via automated flash column chromatography using a gradient of 100:0 to 90:10 DCM:MeOH, followed by recrystallization from MeOH/Acetone to afford the desired product as white crystals (12.8 mg, 32.7 μmol, 14%).

**^1^H NMR:** (600 MHz, DMSO-*d6*) δ 11.71 (s, 1H), 8.60–8.44 (m, 2H), 8.05–7.98 (m, 2H), 7.89 (dd, *J* = 7.8, 1.5 Hz, 1H), 7.40 (m, 1H), 7.23 (td, *J* = 7.6, 1.3 Hz, 1H), 7.16–7.08 (m, 2H), 4.18 (t, *J* = 5.9 Hz, 2H), 2.71 (t, *J* = 5.9 Hz, 2H), 2.48–2.37 (m, 4H), 1.51 (quint, *J* = 5.6 Hz, 4H), 1.42–1.35 (m, 2H).

**^13^C NMR:** (151 MHz, DMSO-*d6*) δ 164.3, 161.2, 145.0, 135.8, 129.0, 128.4, 127.8, 126.7, 126.2, 123.8, 121.7, 119.4, 114.5, 65.6, 57.0, 54.2, 25.3, 23.6.

**HRMS (ESI):** calc. for C_22_H_26_N_5_O_2_ [M + H]^+^: 392.2087, found: 392.2076.


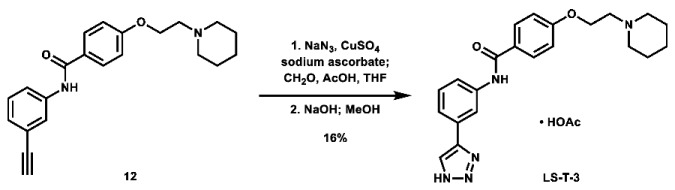


**1-(2-(4-((3-(1*H*-1,2,3-triazol-4-yl)phenyl)carbamoyl)phenoxy)ethyl)piperidin-1-ium acetate (LS-T-3):** To a vial was added formaldehyde (37 wt.% in H_2_O, 0.19 mL, 2.55 mmol, 9.9 eq), acetic acid (0.03 mL, 0.524 mmol, 2.0 eq), and 1.5 mL THF. The solution was stirred for 15 min. To the solution was added sodium azide (27.2 mg, 0.418 mmol, 1.6 eq) and a solution of *N*-(3-ethynylphenyl)-4-(2-(piperidin-1-yl)ethoxy)benzamide (89.4 mg, 0.257 mmol, 1.0 eq) in 1.5 mL THF. The mixture was stirred for 10 min. To the mixture was added a solution of CuSO_4_ (9.1 mg, 57.0 µmol, 0.22 eq) in 0.1 mL H_2_O, followed by sodium ascorbate (31.0 mg, 0.156 mmol, 0.61 eq). The mixture was stirred at room temperature overnight. Starting material was still not consumed, so to a separate vial was added formaldehyde (37 wt.% in H_2_O, 0.19 mL, 2.55 mmol, 9.9 eq), acetic acid (0.03 mL, 0.524 mmol, 2.0 eq), and 1.5 mL THF, which was stirred for 15 min, followed by addition of sodium azide (26.8 mg, 0.412 mmol, 1.6 eq) and stirring for 10 min. The new mixture was added to the initial reaction flask, followed by a solution of CuSO_4_ (8.4 mg, 52.6 µmol, 0.20 eq) in 0.1 mL H_2_O, followed by sodium ascorbate (31.0 mg, 0.156 mmol, 0.61 eq). The mixture was stirred at room temperature overnight. After a total of 46 h, the reaction was stopped, filtered, and evaporated. The crude material was then dissolved in 2 mL MeOH, and 2N NaOH (1.0 mL, 2.0 mmol, 7.8 eq) was added. The mixture was stirred at room temperature for 6 h, after which the solvent was evaporated. The crude material was purified via preparative HPLC using an H_2_O:MeCN:AcOH solvent system to afford the acetate salt of the desired product as a yellow solid (18.7 mg, 41.4 µmol, 16%).

**^1^H NMR:** (600 MHz, DMSO-*d6*) δ 10.19 (s, 1H), 8.33–8.29 (m, 1H), 8.27 (s, 1H), 8.01–7.96 (m, 2H), 7.80–7.75 (m, 1H), 7.58–7.54 (m, 1H), 7.41 (t, *J* = 7.9 Hz, 1H), 7.10–7.04 (m, 2H), 4.15 (t, *J* = 5.9 Hz, 2H), 2.68 (t, *J* = 5.9 Hz, 2H), 2.48–2.37 (m, 4H), 1.87 (s, 3H), 1.50 (quint, *J* = 5.6 Hz, 4H), 1.43–1.34 (m, 2H).

**^13^C NMR:** (151 MHz, DMSO-*d6*) δ 172.3, 164.8, 161.1, 145.2, 139.8, 130.7, 129.5, 129.0, 127.0, 126.6, 120.7, 119.9, 117.3, 114.0, 65.8, 57.2, 54.3, 25.5, 23.8, 21.5.

**HRMS (ESI):** calc. for C_22_H_26_N_5_O_2_ [M + H]^+^: 392.2087, found: 392.2082.


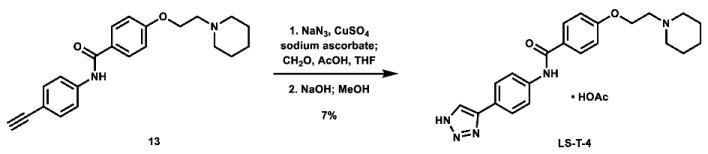


**1-(2-(4-((4-(1*H*-1,2,3-triazol-4-yl)phenyl)carbamoyl)phenoxy)ethyl)piperidin-1-ium acetate (LS-T-4):** To a vial was added formaldehyde (37 wt.% in H_2_O, 0.19 mL, 2.55 mmol, 11.0 eq), acetic acid (0.03 mL, 0.524 mmol, 2.3 eq), and 1.5 mL THF. The solution was stirred for 15 min. To the solution was added sodium azide (27.2 mg, 0.418 mmol, 1.6 eq) and a solution of *N*-(4-ethynylphenyl)-4-(2-(piperidin-1-yl)ethoxy)benzamide (90.6 mg, 0.260 mmol, 1.0 eq) in 1.5 mL THF. The mixture was stirred for 10 min. To the mixture was added a solution of CuSO_4_ (7.1 mg, 44.5 µmol, 0.17 eq) in 0.5 mL H_2_O, followed by sodium ascorbate (31.2 mg, 0.157 mmol, 0.60 eq). The mixture was stirred at room temperature overnight, after which the solvent was removed via rotovap. The residue was then dissolved in 2 mL MeOH and 2N NaOH (1.0 mL, 2.0 mmol, 7.7 eq) and stirred at room temperature for 7 h, after which the solvent was evaporated. The crude material was purified via preparative HPLC using an H_2_O:MeCN:AcOH solvent system to afford the acetate salt of the desired product as a white solid (8.8 mg, 18.3 µmol, 7%).

**^1^H NMR:** (600 MHz, DMSO-*d6*) δ 10.18 (s, 1H), 8.26 (s, 1H), 7.98–7.93 (m, 2H), 7.89–7.85 (m, 2H), 7.85–7.80 (m, 2H), 7.11–7.04 (m, 2H), 4.15 (t, *J* = 5.9 Hz, 2H), 2.68 (t, *J* = 5.9 Hz, 2H), 2.48–2.38 (m, 4H), 1.87 (s, 3H), 1.50 (quint, *J* = 5.6 Hz, 4H), 1.43–1.34 (m, 2H).

**^13^C NMR:** (151 MHz, DMSO-*d6*) δ 172.2, 164.8, 161.1, 144.8, 139.1, 129.5, 126.6, 125.7, 125.2, 120.4, 114.0, 65.8, 57.2, 54.3, 25.5, 23.8, 21.5. Note: One ^13^C signal is missing and is not observable through any 2D correlations.

**HRMS (ESI):** calc. for C_22_H_26_N_5_O_2_ [M + H]^+^: 392.2087, found: 392.2098.


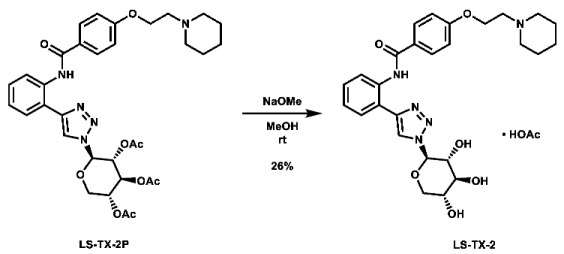


**1-(2-(4-((2-(1-((2*R*,3*R*,4*S*,5*R*)-3,4,5-trihydroxytetrahydro-2*H*-pyran-2-yl)-1*H*-1,2,3-triazol-4-yl)phenyl)carbamoyl) phenoxy)ethyl)piperidin-1-ium acetate (LS-TX-2):** To a vial was added LS-TX-2P (65.3 mg, 0.101 mmol, 1.0 eq), which was dissolved in 4 mL MeOH and 10 mL DMF. To the solution was added 0.5 M NaOMe (0.18 mL, 0.09 mmol, 0.9 eq). The solution was stirred at room temperature for 33 min, after which it was quenched with DOWEX 1x2-200 ion-exchange resin. The mixture was filtered and concentrated. The crude material was purified via preparative HPLC using an H_2_O:MeCN:AcOH solvent system to afford the acetate salt of the desired product as a white solid (15.4 mg, 26.4 µmol, 26%).

**^1^H NMR:** (600 MHz, DMSO-*d6*) δ 11.96 (s, 1H), 9.03 (s, 1H), 8.61 (d, *J* = 8.1 Hz, 1H), 8.05–7.97 (m, 2H), 7.85 (dd, *J* = 7.9, 1.5 Hz, 1H), 7.44–7.35 (m, 1H), 7.25–7.18 (m, 1H), 7.16–7.10 (m, 2H), 5.60 (d, *J* = 9.2 Hz, 1H), 4.18 (t, *J* = 5.9 Hz, 2H), 3.89 (dd, *J* = 11.0, 5.2 Hz, 1H), 3.82 (t, *J* = 9.0 Hz, 1H), 3.54–3.49 (m, 1H), 3.44 (t, *J* = 11.0 Hz, 1H), 3.39 (t, *J* = 9.0 Hz, 1H), 2.68 (t, *J* = 5.9 Hz, 2H), 2.48–2.40 (m, 4H), 1.84 (s, 3H), 1.50 (quint, *J* = 5.6 Hz, 4H), 1.42–1.34 (m, 2H). Note: hydroxyl O-H not observed.

**^13^C NMR:** (151 MHz, DMSO-*d6*) δ 173.5, 164.4, 161.5, 146.3, 136.1, 129.1, 128.7, 127.7, 126.7, 123.8, 122.1, 121.2, 118.3, 114.7, 88.7, 76.8, 72.3, 69.1, 68.5, 66.0, 57.3, 54.4, 25.6, 23.9, 22.0. Note: Note: Acetate carbonyl carbon signal at 173.5 ppm is not observable in the ^13^C spectrum and was assigned using the HMBC correlation from the adjacent CH_3_ (See Supplementary Materials).

**HRMS (ESI):** calc. for C_27_H_34_N_5_O_6_ [M + H]^+^: 524.2509, found: 524.2502.


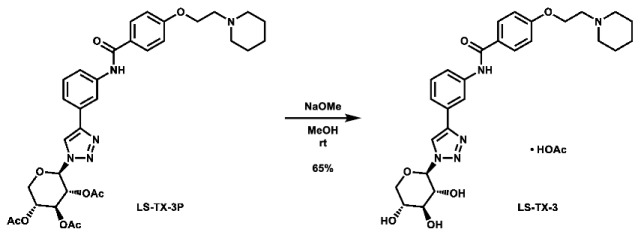


**1-(2-(4-((3-(1-((2*R*,3*R*,4*S*,5*R*)-3,4,5-trihydroxytetrahydro-2*H*-pyran-2-yl)-1*H*-1,2,3-triazol-4-yl)phenyl)carbamoyl) phenoxy)ethyl)piperidin-1-ium acetate (LS-TX-3):** To a vial was added LS-TX-3P (64.4 mg, 99.1 µmol, 1.0 eq), which was dissolved in 4 mL MeOH and 0.5 mL DMF. To the solution was added 0.5 M NaOMe (0.18 mL, 0.09 mmol, 0.9 eq). The solution was stirred at room temperature for 37 min, after which it was quenched with DOWEX 1x2-200 ion-exchange resin. The mixture was filtered and concentrated. The crude material was purified via preparative HPLC using an H_2_O:MeCN:AcOH solvent system to afford the acetate salt of the desired product as a yellow solid (37.6 mg, 64.4 µmol, 65%).

**^1^H NMR:** (600 MHz, DMSO-*d6*) δ 10.20 (s, 1H), 8.78 (s, 1H), 8.39–8.35 (m, 1H), 8.02–7.96 (m, 2H), 7.78–7.74 (m, 1H), 7.56–7.53 (m, 1H), 7.41 (t, *J* = 7.9 Hz, 1H), 7.11–7.05 (m, 2H), 5.53 (d, *J* = 9.2 Hz, 1H), 5.47 (s, 1H), 5.36 (s, 1H), 5.21 (s, 1H), 4.20 (t, *J* = 5.8 Hz, 2H), 3.87 (dd, *J* = 11.0, 5.3 Hz, 1H), 3.83 (t, *J* = 9.1 Hz, 1H), 3.53–3.49 (m, 1H), 3.40 (t, *J* = 10.9 Hz, 1H), 3.37 (t, *J* = 9.1 Hz, 1H), 2.86–2.72 (m, 2H), 2.63–2.51 (m, 4H), 1.91 (s, 3H), 1.58–1.50 (m, 4H), 1.44–1.36 (m, 2H).

**^13^C NMR:** (151 MHz, DMSO-*d6*) δ 172.0, 164.9, 161.1, 146.3, 139.9, 130.9, 129.6, 129.2, 126.8, 120.5, 120.5, 119.9, 117.1, 114.2, 88.4, 77.0, 72.1, 69.2, 68.4, 65.5, 56.9, 54.2, 25.2, 23.6, 21.1.

**HRMS (ESI):** calc. for C_27_H_34_N_5_O_6_ [M + H]^+^: 524.2509, found: 524.2524.


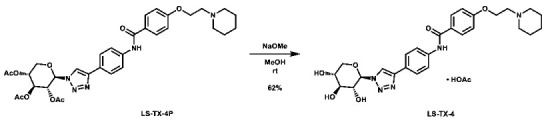


**1-(2-(4-((4-(1-((2*R*,3*R*,4*S*,5*R*)-3,4,5-trihydroxytetrahydro-2*H*-pyran-2-yl)-1*H*-1,2,3-triazol-4-yl)phenyl)carbamoyl) phenoxy)ethyl)piperidin-1-ium acetate (LS-TX-4):** To a vial was added LS-TX-4P (64.7 mg, 99.6 µmol, 1.0 eq), which was dissolved in 4 mL MeOH and 0.5 mL DMF. To the solution was added 0.5 M NaOMe (0.18 mL, 0.09 mmol, 0.9 eq). The solution was stirred at room temperature for 32 min, after which it was quenched with DOWEX 1x2-200 ion-exchange resin. The mixture was filtered and concentrated. The crude material was purified via preparative HPLC using an H_2_O:MeCN:AcOH solvent system to afford the acetate salt of the desired product as a light yellow solid (35.9 mg, 61.5 µmol, 62%).

**^1^H NMR:** (600 MHz, DMSO-*d6*) δ 10.18 (s, 1H), 8.74 (s, 1H), 8.00–7.90 (m, 2H), 7.90–7.80 (m, 4H), 7.13–7.01 (m, 2H), 5.51 (d, *J* = 9.2 Hz, 1H), 4.15 (t, *J* = 5.9 Hz, 2H), 3.86 (dd, *J* = 11.0, 5.3 Hz, 1H), 3.79 (t, *J* = 9.1 Hz, 1H), 3.53–3.48 (m, 1H), 3.41 (t, *J* = 10.4 Hz, 1H), 3.37 (t, *J* = 8.8 Hz, 1H), 2.68 (t, *J* = 5.9 Hz, 2H), 2.48–2.38 (m, 4H), 1.87 (s, 3H), 1.50 (quint, *J* = 5.6 Hz, 4H), 1.42–1.34 (m, 2H). Note: hydroxyl O-H peaks not observed.

**^13^C NMR:** (151 MHz, DMSO-*d6*) δ 172.6, 164.9, 161.2, 146.2, 139.1, 129.6, 126.8, 125.7, 125.5, 120.5, 119.7, 114.1, 88.3, 77.0, 72.2, 69.2, 68.4, 65.9, 57.3, 54.4, 25.6, 23.9, 21.7.

**HRMS (ESI):** calc. for C_27_H_34_N_5_O_6_ [M + H]^+^: 524.2509, found: 524.2528.


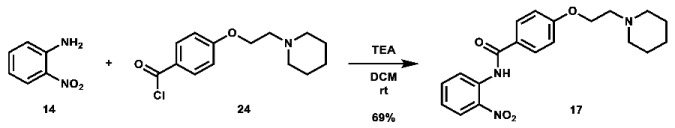


***N*-(2-nitrophenyl)-4-(2-(piperidin-1-yl)ethoxy)benzamide (17):** To an oven-dried 50 mL round bottom flask was added 2-nitroaniline (468.6 mg, 3.393 mmol, 1.0 eq), which was dissolved in 10 mL DCM. Triethylamine (1.5 mL, 10.08 mmol, 3.0 eq) was added, followed by a solution of 4-(2-(piperidin-1-yl)ethoxy)benzoyl chloride (1.3677 g, 5.108 mmol, 1.5 eq) in 10 mL DCM. The solution was stirred at room temperature overnight. Upon completion of the reaction, it was quenched with 10 mL AQ K_2_CO_3_ and extracted with 100 mL DCM. The organic phase was separated and concentrated. The crude material was purified via automated flash column chromatography using a gradient of 75:24:1 to 28:71:1 Hex:EtOAc:TEA to afford the desired product as a light yellow solid (865.2 mg, 2.342 mmol, 69%).

**^1^H NMR:** (600 MHz, CDCl_3_) δ 11.30 (s, 1H), 9.00 (dd, *J* = 8.6, 1.4 Hz, 1H), 8.28 (dd, *J* = 8.5, 1.6 Hz, 1H), 7.99–7.92 (m, 2H), 7.70 (ddd, *J* = 8.7, 7.2, 1.6 Hz, 1H), 7.20 (ddd, *J* = 8.5, 7.2, 1.4 Hz, 1H), 7.05–6.99 (m, 2H), 4.19 (t, *J* = 6.0 Hz, 2H), 2.81 (t, *J* = 6.0 Hz, 2H), 2.62–2.43 (m, 4H), 1.62 (quint, *J* = 5.7 Hz, 5H), 1.49–1.42 (m, 2H).

**^13^C NMR:** (151 MHz, CDCl_3_) δ 165.3, 162.5, 136.3, 136.2, 135.7, 129.4, 126.2, 125.9, 123.0, 122.1, 114.8, 66.4, 57.8, 55.1, 26.0, 24.2.

**HRMS (ESI):** calc. for C_20_H_24_N_3_O_4_ [M + H]^+^: 370.1762, found: 370.1755.


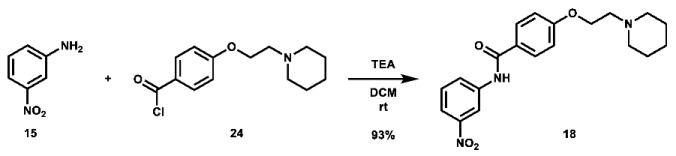


***N*-(3-nitrophenyl)-4-(2-(piperidin-1-yl)ethoxy)benzamide (18):** To an oven-dried 100 mL round bottom flask was added 3-nitroaniline (469.8 mg, 3.401 mmol, 1.0 eq), which was dissolved in 10 mL DCM. Triethylamine (1.5 mL, 10.08 mmol, 3.0 eq) was added, followed by a solution of 4-(2-(piperidin-1-yl)ethoxy)benzoyl chloride (1.3645 g, 5.096 mmol, 1.5 eq) in 10 mL DCM. The solution was stirred at room temperature overnight. Upon completion of the reaction, it was quenched with 10 mL AQ K_2_CO_3_ and extracted with 100 mL DCM. The organic phase was separated and concentrated. The crude material was purified via automated flash column chromatography using a gradient of 79:20:0:1 to 0:99:0:1 to 0:97:2:1 Hex:EtOAc:MeOH:TEA to afford the desired product as a yellow solid (1.1632 g, 3.149 mmol, 93%).

**^1^H NMR:** (600 MHz, CDCl_3_) δ 8.47 (t, *J* = 2.2 Hz, 1H), 8.14–8.06 (m, 2H), 7.98 (m, 1H), 7.88–7.83 (m, 2H), 7.52 (t, *J* = 8.2 Hz, 1H), 7.02–6.96 (m, 2H), 4.18 (t, *J* = 6.0 Hz, 2H), 2.80 (t, *J* = 6.0 Hz, 2H), 2.59–2.44 (m, 4H), 1.62 (quint, *J* = 5.6 Hz, 4H), 1.50–1.43 (m, 2H).

**^13^C NMR:** (151 MHz, CDCl_3_) δ 165.7, 162.1, 148.5, 139.5, 129.8, 129.2, 126.1, 126.0, 118.7, 115.0, 114.6, 66.2, 57.8, 55.1, 25.9, 24.1.

**HRMS (ESI):** calc. for C_20_H_24_N_3_O_4_ [M + H]^+^: 370.1762, found: 370.1752.


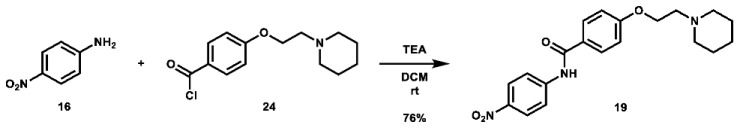


***N*-(4-nitrophenyl)-4-(2-(piperidin-1-yl)ethoxy)benzamide (19):** To an oven-dried 100 mL round bottom flask was added 4-nitroaniline (468.8 mg, 3.394 mmol, 1.0 eq), which was dissolved in 15 mL DCM. Triethylamine (1.5 mL, 10.08 mmol, 3.0 eq) was added, followed by a solution of 4-(2-(piperidin-1-yl)ethoxy)benzoyl chloride (1.3658 g, 5.101 mmol, 1.5 eq) in 10 mL DCM. The solution was stirred at room temperature overnight. After 49 h, it was quenched with 60 mL AQ K_2_CO_3_ and extracted with 100 mL DCM. The organic phase was separated and concentrated. The crude material was purified via automated flash column chromatography using a gradient of 85:14:0:1 to 0:99:0:1 to 0:96:3:1 Hex:EtOAc:MeOH:TEA. The resultant material still contained an impurity (likely acid or the ester of the benzoyl chloride), so it was dissolved in 200 mL DCM and washed with 100 mL 5% NaOH. The organic layer was separated and concentrated to afford the desired product as a light brown solid (954.0 mg, 2.582 mmol, 76%).

**^1^H NMR:** (600 MHz, CDCl_3_) δ 8.30–8.22 (m, 2H), 8.00 (s, 1H), 7.89–7.78 (m, 4H), 7.04–6.99 (m, 2H), 4.18 (t, *J* = 6.0 Hz, 2H), 2.81 (t, *J* = 6.0 Hz, 2H), 2.59–2.44 (m, 4H), 1.62 (quint, *J* = 5.6 Hz, 4H), 1.50–1.42 (m, 2H).

**^13^C NMR:** (151 MHz, CDCl_3_) δ 165.2, 162.4, 144.0, 143.5, 129.1, 126.0, 125.2, 119.3, 114.8, 66.4, 57.7, 55.2, 25.9, 24.1.

**HRMS (ESI):** calc. for C_20_H_24_N_3_O_4_ [M + H]^+^: 370.1762, found: 370.1752.


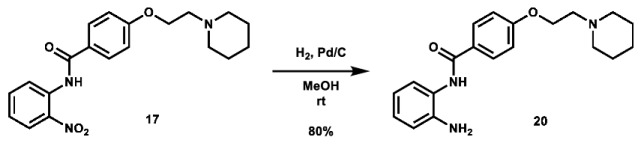


***N*-(2-aminoophenyl)-4-(2-(piperidin-1-yl)ethoxy)benzamide (20):** To a 50 mL round bottom flask was added *N*-(2-nitrophenyl)-4-(2-(piperidin-1-yl)ethoxy)benzamide (823.7 mg, 2.230 mmol, 1.0 eq), which was dissolved in 25 mL MeOH. To the solution was added palladium on charcoal (10 wt.%, 120.6 mg, 12.06 mg Pd, 0.113 mmol Pd, 0.05 eq). The flask headspace was evacuated and filled 3 times with hydrogen and the mixture was stirred under hydrogen at room temperature overnight. Upon completion of the reaction, the mixture was filtered over celite and concentrated. The crude material was purified via automated flash column chromatography using a gradient of 84:15:1 to 0:99:1 Hex:EtOAc:TEA to afford the product as a pale yellow crystalline solid (602.2 mg, 1.774 mmol, 80%).

**^1^H NMR:** (600 MHz, CDCl_3_) δ 7.91–7.83 (m, 2H), 7.74 (s, 1H), 7.31 (m, 1H), 7.09 (td, *J* = 7.6, 1.5 Hz, 1H), 7.01–6.94 (m, 2H), 6.88–6.81 (m, 2H), 4.17 (t, *J* = 6.1 Hz, 2H), 3.88 (s, 2H), 2.80 (t, *J* = 6.0 Hz, 2H), 2.65–2.38 (m, 4H), 1.62 (quint, *J* = 5.7 Hz, 4H), 1.50–1.41 (m, 2H).

**^13^C NMR:** (151 MHz, CDCl_3_) δ 165.3, 161.9, 140.7, 129.1, 127.1, 126.3, 125.2, 124.8, 119.8, 118.4, 114.6, 66.3, 57.8, 55.1, 26.0, 24.2.

**HRMS (ESI):** calc. for C_20_H_26_N_3_O_2_ [M + H]^+^: 340.2020, found: 340.2017.


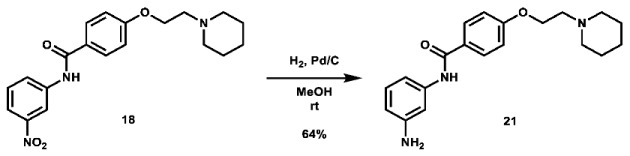


***N*-(3-aminophenyl)-4-(2-(piperidin-1-yl)ethoxy)benzamide (21):** To a 100 mL round bottom flask was added *N*-(3-nitrophenyl)-4-(2-(piperidin-1-yl)ethoxy)benzamide (1.0012 g, 2.710 mmol, 1.0 eq), which was dissolved in 55 mL MeOH. To the solution was added palladium on charcoal (10 wt.%, 145.0 mg, 14.5 mg Pd, 0.136 mmol Pd, 0.05 eq). The flask headspace was evacuated and filled 3 times with hydrogen and the mixture was stirred under hydrogen at room temperature overnight. Upon completion of the reaction, the mixture was filtered over celite and concentrated. The crude material was purified via automated flash column chromatography using a gradient of 74:25:0:1 to 0:99:0:1 to 0:94:5:1 Hex:EtOAc:MeOH:TEA to afford the product as a pale orange solid (586.0 mg, 1.742 mmol, 64%).

**^1^H NMR:** (600 MHz, CDCl_3_) δ 7.83–7.77 (m, 2H), 7.72 (s, 1H), 7.30 (t, *J* = 2.1 Hz, 1H), 7.10 (t, *J* = 8.0 Hz, 1H), 6.98–6.93 (m, 2H), 6.77 (m, 1H), 6.45 (m, 1H), 4.15 (t, *J* = 6.0 Hz, 2H), 3.73 (s, 2H), 2.79 (t, *J* = 6.0 Hz, 2H), 2.60–2.43 (m, 4H), 1.61 (quint, *J* = 5.6 Hz, 4H), 1.50–1.41 (m, 2H).

**^13^C NMR:** (151 MHz, CDCl_3_) δ 165.2, 161.7, 147.3, 139.2, 129.7, 128.8, 127.3, 114.5, 111.1, 110.0, 106.8, 66.2, 57.8, 55.1, 25.9, 24.2.

**HRMS (ESI):** calc. for C_20_H_26_N_3_O_2_ [M + H]^+^: 340.2020, found: 340.2015.


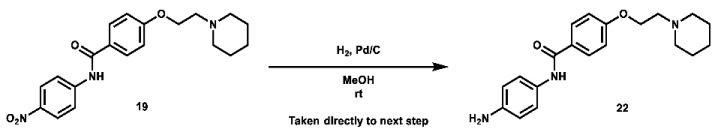


***N*-(4-aminophenyl)-4-(2-(piperidin-1-yl)ethoxy)benzamide (22):** To a 100 mL round bottom flask was added *N*-(4-nitrophenyl)-4-(2-(piperidin-1-yl)ethoxy)benzamide (895.7 mg, 2.425 mmol, 1.0 eq), which was dissolved in 55 mL MeOH. To the solution was added palladium on charcoal (10 wt.%, 131.7 mg, 13.2 mg Pd, 0.123 mmol Pd, 0.05 eq). The flask headspace was evacuated and filled 3 times with hydrogen and the mixture was stirred under hydrogen at room temperature overnight. Upon completion of the reaction, the mixture was filtered over celite and concentrated. The crude material was purified via automated flash column chromatography using a gradient of 49:50:0:1 to 0:99:0:1 to 0:94:5:1 Hex:EtOAc:MeOH:TEA. Despite multiple attempts at purification, the product was unable to be separated from residual starting material. Crude yellow solid (530.1 mg) taken directly to next steps.

**^1^H NMR:** (600 MHz, CDCl_3_) δ 7.84–7.76 (m, 2H), 7.64 (s, 1H), 7.42–7.34 (m, 2H), 6.97–6.92 (m, 2H), 6.71–6.65 (m, 2H), 4.18 (t, *J* = 6.0 Hz, 2H), 3.63 (s, 2H), 2.82 (t, *J* = 5.9 Hz, 2H), 2.63–2.47 (m, 4H), 1.64 (quint, *J* = 5.7 Hz, 4H), 1.50–1.43 (m, 2H).

**HRMS (ESI):** calc. for C_20_H_26_N_3_O_2_ [M + H]^+^: 340.2020, found: 340.2013.


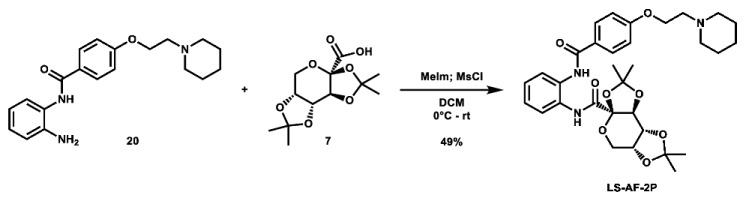


**(3a*R*,5a*R*,8a*R*,8b*S*)-2,2,7,7-tetramethyl-*N*-(2-(4-(2-(piperidin-1-yl)ethoxy)benzamido)phenyl)tetrahydro-3a*H*-bis**([1,3]**dioxolo)[4,5-*b*:4′,5′-*d*]pyran-3a-carboxamide (LS-AF-2P):** To an oven-dried vial was added 2,3;4,5-di-*O*-isopropylidene-2-oxo-D-glucuronic acid (97.7 mg, 0.356 mmol, 1.0 eq), which was dissolved in 4 mL DCM. The solution was cooled to 0 °C and *N*-methyl imidazole (0.07 mL, 0.878 mmol, 2.5 eq) was added. The solution was stirred for 10 min, after which a solution of methanesulfonyl chloride (0.03 mL, 0.388 mmol, 1.1 eq) in 1 mL DCM was added. The solution was stirred at 0 °C for 30 min. To the solution was then added a solution of *N*-(2-aminoophenyl)-4-(2-(piperidin-1-yl)ethoxy)benzamide (120.2 mg, 0.354 mmol, 1.0 eq) in 5 mL DCM. The solution was stirred overnight and allowed to slowly warm to room temperature. After 23 h, the reaction was quenched with 25 mL ice-cold H_2_O and extracted with 2 × 40 mL portions of DCM. The organic layers were combined and concentrated. The crude material was twice purified via automated flash column chromatography, first using a gradient of 100:0 to 90:10 DCM:MeOH, then 74:25:1 to 0:99:1 Hex:EtOAc:TEA to afford the desired product as a red residue (104.5 mg, 0.175 mmol, 49%).

**^1^H NMR:** (600 MHz, CDCl_3_) δ 8.92 (s, 1H), 8.84 (s, 1H), 8.03 (m, 1H), 7.93–7.88 (m, 2H), 7.34 (ddd, *J* = 8.5, 6.1, 2.8 Hz, 1H), 7.20–7.14 (m, 2H), 6.94 (d, *J* = 12.5 Hz, 2H), 4.85 (d, *J* = 2.6 Hz, 1H), 4.65 (dd, *J* = 7.9, 2.6 Hz, 1H), 4.30–4.25 (m, 1H), 4.16 (t, *J* = 6.1 Hz, 2H), 4.00 (dd, *J* = 12.9, 1.8 Hz, 1H), 3.93 (d, *J* = 13.0 Hz, 1H), 2.80 (t, *J* = 6.0 Hz, 2H), 2.61–2.42 (m, 4H), 1.64–1.60 (m, 4H), 1.59 (s, 3H), 1.59 (s, 3H), 1.49–1.41 (m, 2H), 1.29 (s, 3H), 1.24 (s, 3H).

**^13^C NMR:** (151 MHz, CDCl_3_) δ 167.7, 164.9, 161.6, 132.4, 129.4, 127.9, 127.4, 126.8, 125.9, 125.3, 124.6, 114.2, 110.9, 109.2, 99.5, 72.7, 70.2, 69.9, 66.1, 62.0, 57.8, 55.1, 26.3, 25.9, 25.9, 24.8, 24.2, 23.9.

**HRMS (ESI):** calc. for C_32_H_42_N_3_O_8_ [M + H]^+^: 596.2972, found: 596.2980.


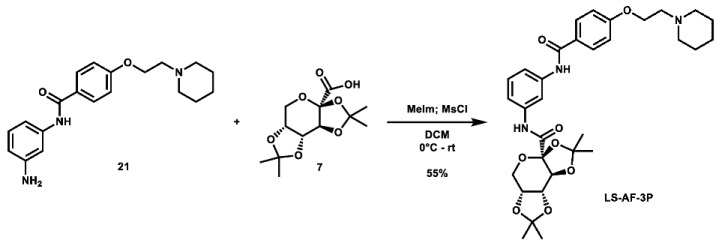


**(3a*R*,5a*R*,8a*R*,8b*S*)-2,2,7,7-tetramethyl-*N*-(3-(4-(2-(piperidin-1-yl)ethoxy)benzamido)phenyl)tetrahydro-3a*H*-bis**([1,3]**dioxolo)[4,5-*b*:4′,5′-*d*]pyran-3a-carboxamide (LS-AF-3P):** To an oven-dried vial was added 2,3;4,5-di-*O*-isopropylidene-2-oxo-D-glucuronic acid (96.0 mg, 0.350 mmol, 1.0 eq), which was dissolved in 4 mL DCM. The solution was cooled to 0 °C and *N*-methyl imidazole (0.07 mL, 0.878 mmol, 2.5 eq) was added. The solution was stirred for 10 min, after which a solution of methanesulfonyl chloride (0.03 mL, 0.388 mmol, 1.1 eq) in 1 mL DCM was added. The solution was stirred at 0 °C for 30 min. To the solution was then added a solution of *N*-(3-aminoophenyl)-4-(2-(piperidin-1-yl)ethoxy)benzamide (119.8 mg, 0.353 mmol, 1.0 eq) in 5 mL DCM. The solution was stirred overnight and allowed to slowly warm to room temperature. Upon completion, the reaction was quenched with 25 mL ice-cold H_2_O and extracted with 2 × 40 mL portions of DCM. The organic layers were combined and concentrated. The crude material was twice purified via automated flash column chromatography, first using a gradient of 69:30:0:1 to 0:99:0:1 to 0:89:10:1 Hex:EtOAc:MeOH:TEA, then 100:0 to 90:10 DCM:MeOH to afford the desired product as a red residue (114.0 mg, 0.191 mmol, 55%).

**^1^H NMR:** (600 MHz, CDCl_3_) δ 8.71 (s, 1H), 8.01 (t, *J* = 2.1 Hz, 1H), 7.85–7.80 (m, 2H), 7.79 (s, 1H), 7.60 (m, 1H), 7.32 (t, *J* = 8.1 Hz, 1H), 7.24 (m, 1H), 7.01–6.95 (m, 2H), 4.75 (d, *J* = 2.6 Hz, 1H), 4.65 (dd, *J* = 7.9, 2.5 Hz, 1H), 4.29 (d, *J* = 8.0 Hz, 1H), 4.27–4.23 (m, 2H), 4.02 (dd, *J* = 12.9, 1.8 Hz, 1H), 3.96 (dd, *J* = 12.9, 0.9 Hz, 1H), 3.00–2.87 (m, 2H), 2.82–2.48 (m, 4H), 1.75–1.68 (m, 4H), 1.60 (s, 3H), 1.59 (s, 3H), 1.54–1.47 (m, 2H), 1.41 (s, 3H), 1.33 (s, 3H).

**^13^C NMR:** (151 MHz, CDCl_3_) δ 166.2, 165.1, 161.4, 138.9, 137.7, 129.7, 129.0, 127.2, 116.3, 115.3, 114.6, 111.2, 110.8, 109.1, 99.7, 72.9, 70.2, 69.9, 65.5, 61.8, 57.4, 54.9, 26.3, 26.1, 25.2, 24.8, 23.8, 23.7.

**HRMS (ESI):** calc. for C_32_H_42_N_3_O_8_ [M + H]^+^: 596.2972, found: 596.2975.


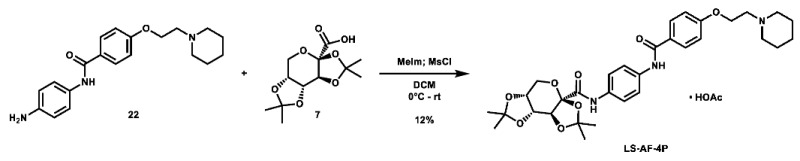


**1-(2-(4-((4-((3a*R*,5a*R*,8a*R*,8b*S*)-2,2,7,7-tetramethyltetrahydro-5*H*-bis**([1,3]**dioxolo)[4,5-*b*:4′,5′-*d*]pyran-3a-carboxamido)phenyl)carbamoyl)phenoxy)ethyl)piperidin-1-ium acetate (LS-AF-4P):** To an oven-dried vial was added 2,3;4,5-di-*O*-isopropylidene-2-oxo-D-glucuronic acid (97.1 mg, 0.354 mmol, 1.0 eq), which was dissolved in 4 mL DCM. The solution was cooled to 0 °C and *N*-methyl imidazole (0.07 mL, 0.878 mmol, 2.5 eq) was added. The solution was stirred for 10 min, after which a solution of methanesulfonyl chloride (0.03 mL, 0.388 mmol, 1.1 eq) in 1 mL DCM was added. The solution was stirred at 0 °C for 35 min. To the solution was then added a solution of *N*-(4-aminoophenyl)-4-(2-(piperidin-1-yl)ethoxy)benzamide (119.4 mg, 0.352 mmol, 1.0 eq) in 5 mL DCM and 0.5 mL anhydrous DMF. The solution was stirred overnight and allowed to slowly warm to room temperature. Upon completion, the reaction was quenched with 25 mL ice-cold H_2_O and extracted with 2 × 50 mL portions of DCM. The organic layers were combined and concentrated. The crude material was purified via preparative HPLC using an H_2_O:MeCN:AcOH gradient to afford the acetate salt of the desired product as a white solid (27.1 mg, 41.3 µmol, 12%).

**^1^H NMR:** (600 MHz, DMSO-*d6*) δ 10.05 (s, 1H), 9.71 (s, 1H), 7.96–7.91 (m, 2H), 7.70–7.67 (m, 2H), 7.67–7.62 (m, 2H), 7.07–7.04 (m, 2H), 4.69 (d, *J* = 2.5 Hz, 1H), 4.66 (dd, *J* = 7.9, 2.6 Hz, 1H), 4.32 (dd, *J* = 7.9, 1.4 Hz, 1H), 4.14 (t, *J* = 5.9 Hz, 2H), 3.91–3.83 (m, 2H, os), 2.67 (t, *J* = 5.9 Hz, 2H), 2.48–2.38 (m, 4H), 1.88 (s, 3H), 1.52 (s, 3H), 1.49 (quint, *J* = 5.6 Hz, 4H), 1.46 (s, 3H), 1.42–1.35 (m, 2H), 1.30 (s, 3H), 1.27 (s, 3H).

**^13^C NMR:** (151 MHz, DMSO-*d6*) δ 172.2, 165.8, 164.6, 161.1, 135.5, 133.5, 129.5, 126.8, 120.6, 120.5, 114.1, 109.6, 108.3, 99.4, 72.0, 69.7, 69.3, 65.9, 61.3, 57.3, 54.4, 26.0, 25.8, 25.6, 24.7, 24.1, 23.9, 21.4.

**HRMS (ESI):** calc. for C_32_H_42_N_3_O_8_ [M + H]^+^: 596.2972, found: 596.2970.

### 3.3. Docking Studies

Docking of the disclosed novel compounds was conducted using GlideSP from the Schrödinger Suite and visualized via Maestro [51,52,68]. All docking was conducted using an 8–10 Å RMSD gp130-D1 conformation generated via molecular dynamics studies previously conducted by Dr. Guqin Shi [69], which was prepared via the Protein Preparation Wizard prior to docking [68,70]. Ligands were drawn in ChemDraw and imported into Chem3D for MM2 minimization [71] and saved as .pdb or .sdf files. Ligand files were then imported into Maestro and prepared using LigPrep [68], with either the OPLS3e [68,72,73,74,75,76] or OPLS4 [77] force field. Appropriately sized grid boxes were generated centered around the gp130-D1 ligand binding site, and ligands were docked via GlideSP. Results were analyzed visually.

Docking of MDL-A was conducted using AutoDock 4.2 [49]. The same gp130-D1 conformer was used as with GlideSP, and the grid box was generated with its center on the ligand binding site, sized according to ligand geometry (0.375 Å spacing). Ligand and protein atoms were assigned appropriate Gasteiger charges [78], and docking was conducted using the Lamarckian Genetic Algorithm search method [79]. Results were clustered at an RMSD of 2.0 Å and analyzed visually.

### 3.4. Cell Culture

In this study, three human breast cancer cell lines were used: T47D, MDA-MB-231, and SUM159. Each of these cells was cultured in Dulbecco’s Modified Eagle Medium (Corning, NY, USA) that was supplemented with 10% fetal bovine serum (Sigma-Aldrich, St. Louis, MO, USA) and 1% Penicillin/Streptomycin (Sigma-Aldrich, St. Louis, MO, USA). Human prostate cancer PC3 and LNCaP cell lines were obtained from American Type Culture Collection (ATCC). Cells were maintained in RPMI-1640 (Corning, NY, USA) supplemented with 10% fetal bovine serum (R&D Systems, Minneapolis, MN, USA) and 1% Penicillin/Streptomycin (Corning, NY, USA). All cells were maintained at 37 °C in a humidified atmosphere with 5% CO_2_.

### 3.5. Cell Viability Assays

#### 3.5.1. 72 h MTT Assays against Breast Cancer Cell Lines

MDA-MB-231 and SUM159 cells were plated at a density of 3000 cells/well in a 96-well plate. After overnight incubation, cells were treated with IL-6-signaling inhibitors for 72 h. Subsequently, 20 µL of 5 mg/mL thiazolyl blue tetrazolium dye solution (Sigma-Aldrich, St. Louis, MO, USA) was added to each well of the plate, which were then incubated at 37 °C for 4 h. The formazan solution was prepared using 100 µL of *N,N*-dimethylformamide (Sigma-Aldrich, St. Louis, MO, USA). Absorbance at 595 nm was read using an EL808 Ultra Microplate Reader (BioTek, Winooski, VT, USA).

#### 3.5.2. 72 h CCK-8 Assays against Prostate Cancer Cell Lines

PC3 and LNCaP cells were seeded in triplicate at a density of 3000 cells/well in 96-well plates and allowed to adhere for 24 h. Cells were then treated with media containing 1% DMSO (vehicle) or 10 nM to 100 µM LS-TG-2P or LS-TF-3P for 72 h. Media was then replaced with fresh media containing CCK-8 reagent (MedChemExpress, South Brunswick Township, NJ, USA) according to the manufacturer’s protocol and incubated for 1 h at 37 °C. The absorbance was measured at 450 nm using an Eon Micro-plate Spectrophotometer (BioTek, Winooski, VT, USA). Background absorbance was subtracted, and the percent viable cells were calculated relative to the vehicle-treated control cells. IC_50_ values were calculated using GraphPad Prism (v 7.04). Data represent the average of three independent experiments.

### 3.6. Cytokine Selectivity Assays

Human Interleukin-6 (IL-6), Interferon-γ (IFN-γ), Leukemia inhibitory factor (LIF), and Oncostatin M (OSM) were prepared according to the manufacturer’s instructions (Cell Signaling Technology, Danvers, MA, USA). T47D breast cancer cells were pretreated with DMSO, LS-TF-3P, or LS-TG-2P in a serum-free medium for 2 h and then stimulated with 50 ng/mL of IL-6, IFN-γ, LIF, or OSM for an additional 30 min. Cells were collected and analyzed by Western blot. Briefly, equal amounts of protein were separated by 10% SDS–PAGE gels and transferred to PVDF membranes. Membranes were blocked in 5% non-fat milk at room temperature and probed with specific antibodies P-STAT3 (Y705), STAT3, P-STAT1 (Y701), STAT1, or GAPDH (1:1000, Cell Signaling Technology, Danvers, MA, USA) at 4 °C overnight. The blots were visualized using SuperSignalTM West Femto Maximum Sensitivity Substrate (Thermo Fisher Scientific, Waltham, MA, USA) and an Amersham Imager 680 (GE Healthcare Life Sciences, Marlborough, MA, USA) after incubating with horseradish peroxidase (HRP)-conjugated anti-rabbit secondary antibody (1:5000, Cell Signaling Technology, Danvers, MA, USA).

## 4. Patents

The work reported in this manuscript has been disclosed as part of a patent filing: Li, C.; Schultz, D.; Shen, Z.; Multifaceted Approach to Novel Interleukin-6 Inhibitors. WO2022226133A1, 2022.

## Figures and Tables

**Table 1:** MTT Assay Data (72-h) and logP Values for Small Molecule-Carbohydrate Analogues.

Compound	SUM159 IC_50_ (µM)	MDA-MB-231 IC_50_ (µM)	logP
**Bazedoxifene**	7.1 ± 1.1	12 ± 2	6.01
**LS-TX-2**	Inactive ^1^	Inactive ^2^	1.92
**LS-TX-3**	Inactive ^1^	Inactive ^2^	1.92
**LS-TX-4**	Inactive ^1^	Inactive ^2^	1.92
**LS-TF-2P**	41 ± 5.4	96 ± 1.6	4.39
**LS-TF-3P**	16 ± 0.8	28.7 ± 2.1	4.39
**LS-TF-4P**	26.1 ± 3.8	18.4 ± 5.5	4.39
**LS-TG-2P**	6.9 ± 0.2	10.5 ± 0.3	2.30
**LS-TG-3P**	14.8 ± 1.3	49.4 ± 1.4	2.30
**LS-TG-4P**	2.5 ± 0.3	27.6 ± 4.8	2.30
**LS-TX-2P**	12.4 ± 0.8	27.4 ± 1.6	2.61
**LS-TX-3P**	20.8 ± 4.6	60.6 ± 2.0	2.61
**LS-TX-4P**	10.6 ± 0.8	32.2 ± 2.9	2.61
**LS-AF-2P**	103 ± 6.1	196 ± 8.4	3.43
**LS-AF-3P**	44 ± 3.7	h86 ± 7	3.43
**LS-AF-4P**	18.1 ± 1.3	59.3 ± 7.4	3.43

IC_50_ ± SE. logP values calculated using ChemDraw 18.1. ^1^ Inactive up to the highest tested concentration of 20 µM. ^2^ Inactive up to the highest tested concentration of 50 µM.

**Table 2:** MTT Assay Data (72 h) and logP Values for *N*-Unsubstituted Triazole Analogues.

Compound	SUM159 IC_50_ (µM)	MDA-MB-231 IC_50_ (µM)	logP
**LS-T-2**	25.5 ± 2.3	Inactive	3.25
**LS-T-3**	55.5 ± 6.4	Inactive	3.25
**LS-T-4**	12.4 ± 0.8	13 ± 1.4	3.25

Inactive compounds displayed no activity up to the highest tested concentration of 50 µM. IC_50_ ± SE. logP values calculated using ChemDraw 18.1.

**Table 3:** CCK-8 Assay Data for Key Carbohydrate-Containing Analogues Against Prostate Cancer Cell Lines (72 h).

Compound	PC3 IC_50_ (µM)	LNCaP IC_50_ (µM)
**Bazedoxifene**	7.5 ± 0.3	11.1 ± 0.6 *
**LS-TG-2P**	6.2 ± 0.2 *	7.7 ± 0.3
**LS-TF-3P**	19.0 ± 0.8	50.0 ± 2.9

* 95% confidence interval was ambiguous due to a steep dose–response curve. IC_50_ ± SE.

## Data Availability

The data presented in this study are available in the article and Supplementary Materials.

## Supplementary Materials

The following supporting information can be downloaded at: https://www.mdpi.com/article/10.3390/molecules28020677/s1, ^1^H, ^13^C, and 2D NMR spectra for the disclosed compounds and intermediates.

## Sample Availability

Samples of select compounds are available from the corresponding author upon request.
