# Supplementary material for: Carbohydrate-Small Molecule Hybrids as Lead Compounds Targeting IL-6 Signaling
Source: Molecules. 2023 Jan 9;28(2):677. doi: 10.3390/molecules28020677 (PMC9861960; doi:10.3390/molecules28020677)
Supplement: Supplementary file 1 [file molecules-28-00677-s001.zip › molecules-2116805-supplementary.pdf]

Carbohydrate-Small Molecule Hybrids as Lead Compounds Targeting IL-6 Signaling

Daniel C. Schultz [1], Li Pan [2], Tiffany Wang [2], Conner Booker [1], Iram Hyder [1], Laura Hanold [1], Garret Rubin [1], Yousong Ding [1], Jiayuh Lin [2], and Chenglong Li [1,*]

Supplementary Information:

^{1}H, ^{13}C, and 2D NMR Spectra

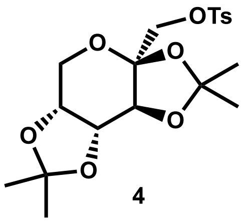

4

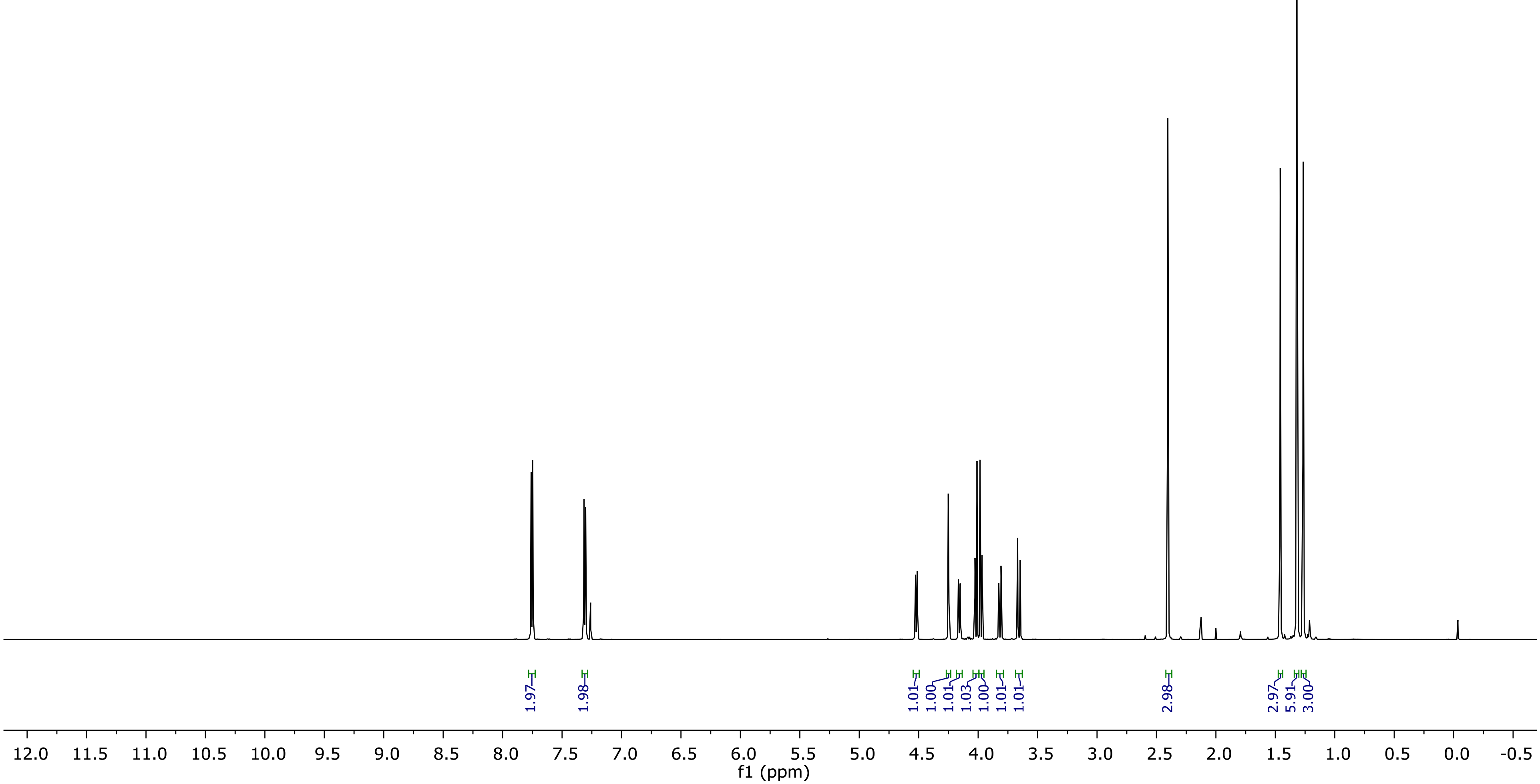

^{1}H NMR Spectrum for Compound **4** ($CDCl_3$, 600 MHz).

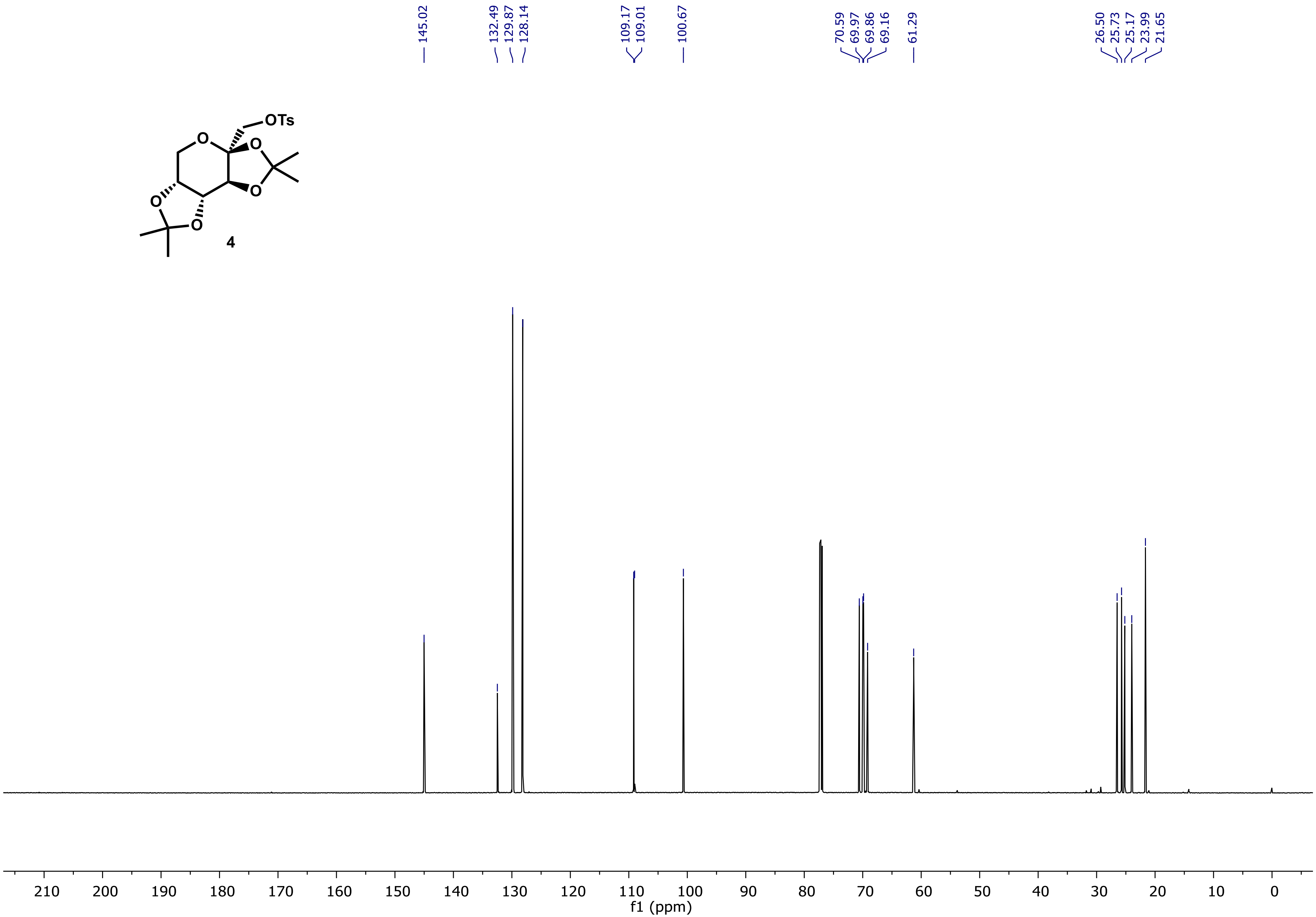

^{13}C NMR Spectrum for Compound **4** ($CDCl_3$, 151 MHz).

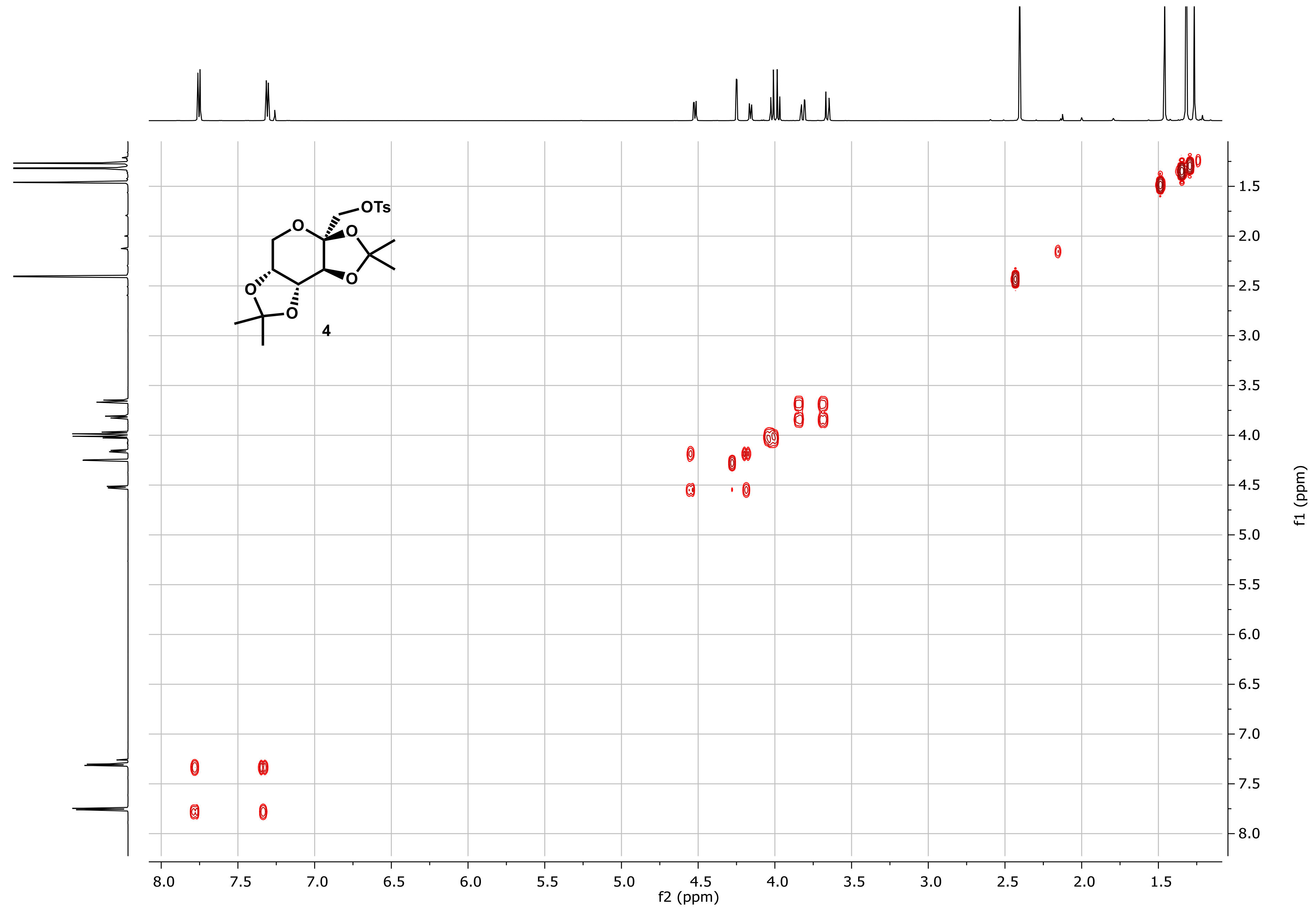

COSY Spectrum for Compound **4** ($CDCl_3$).

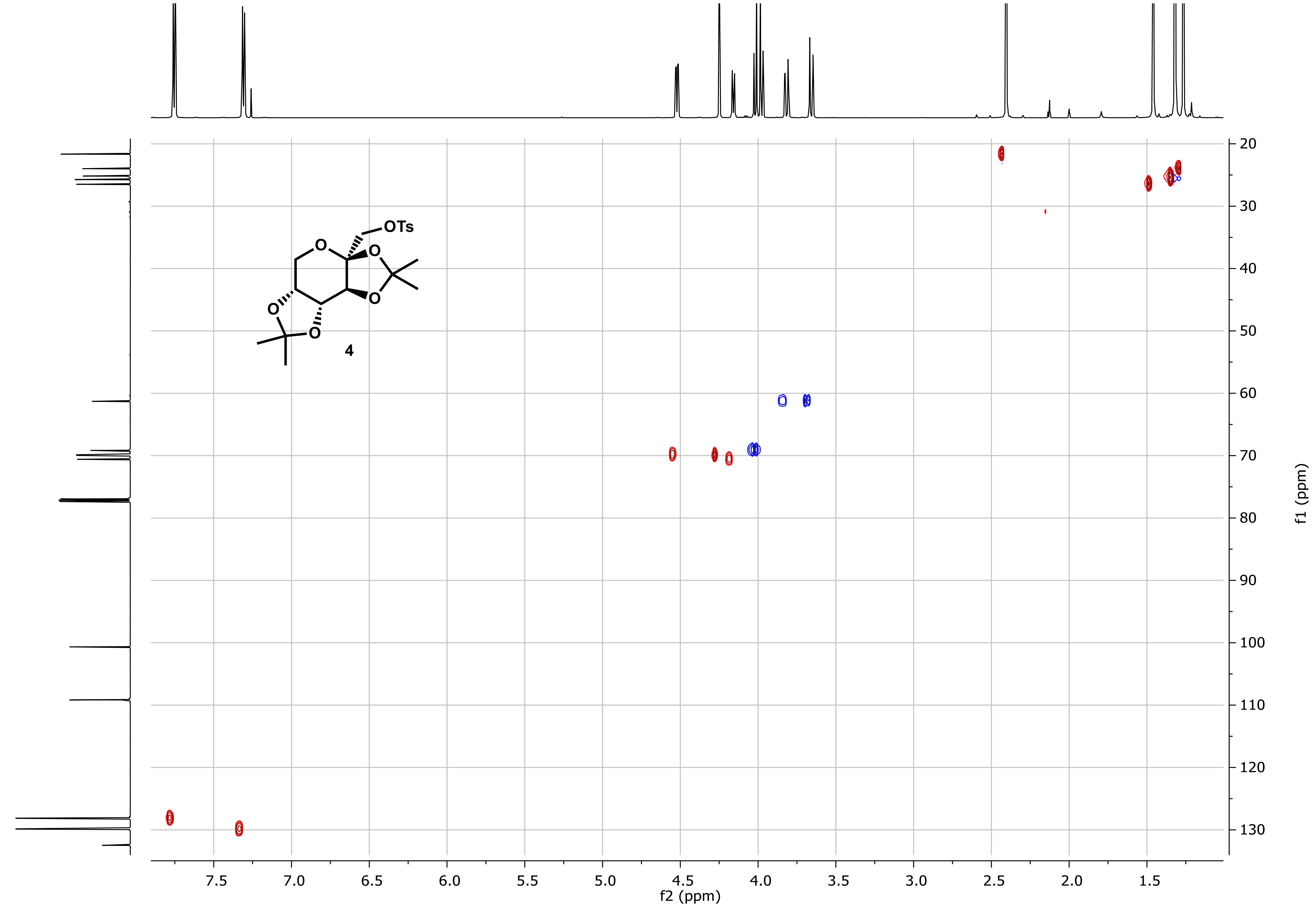

HSQC Spectrum for Compound **4** ($CDCl_3$).

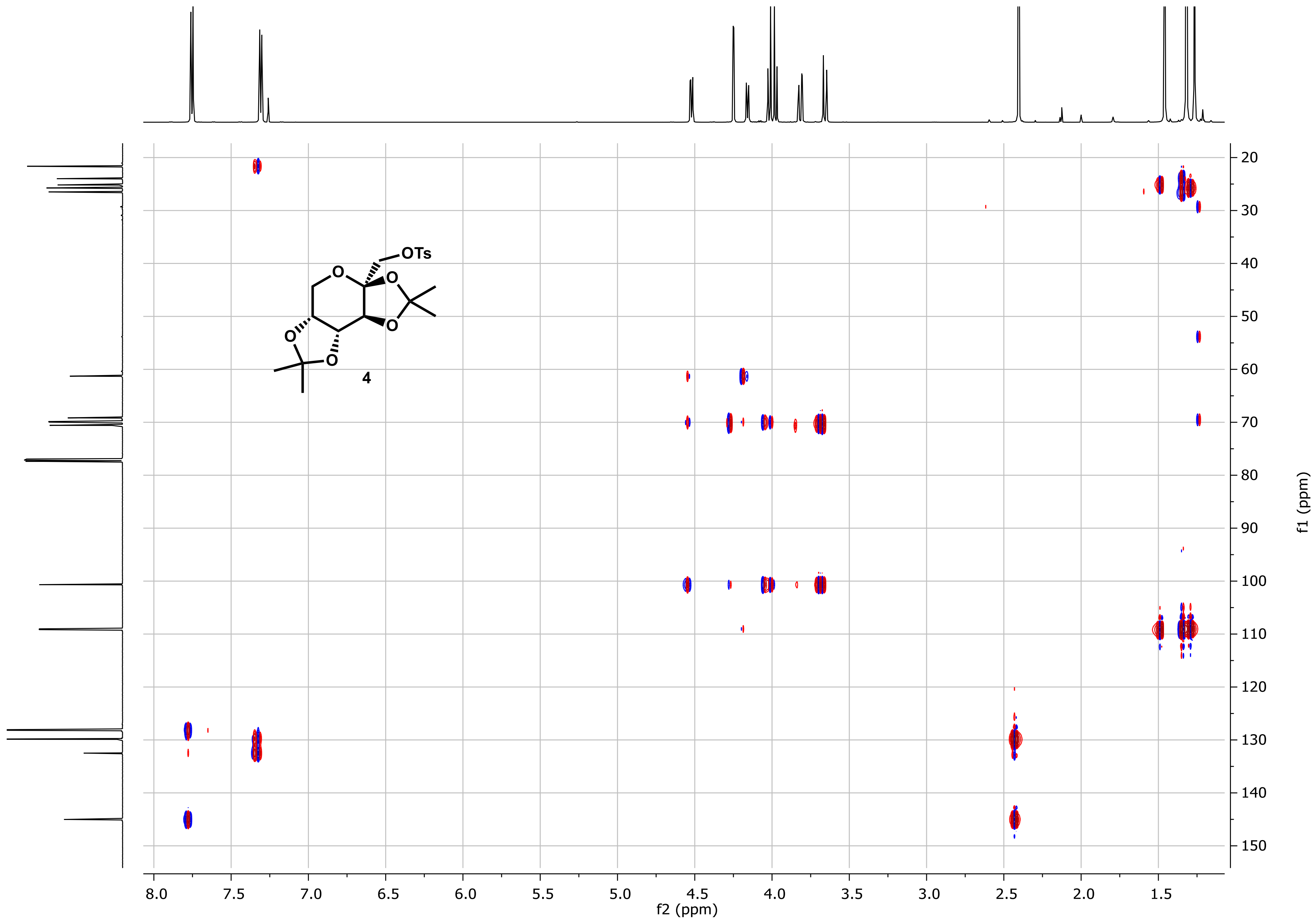

HMBC Spectrum for Compound **4** ($CDCl_3$).

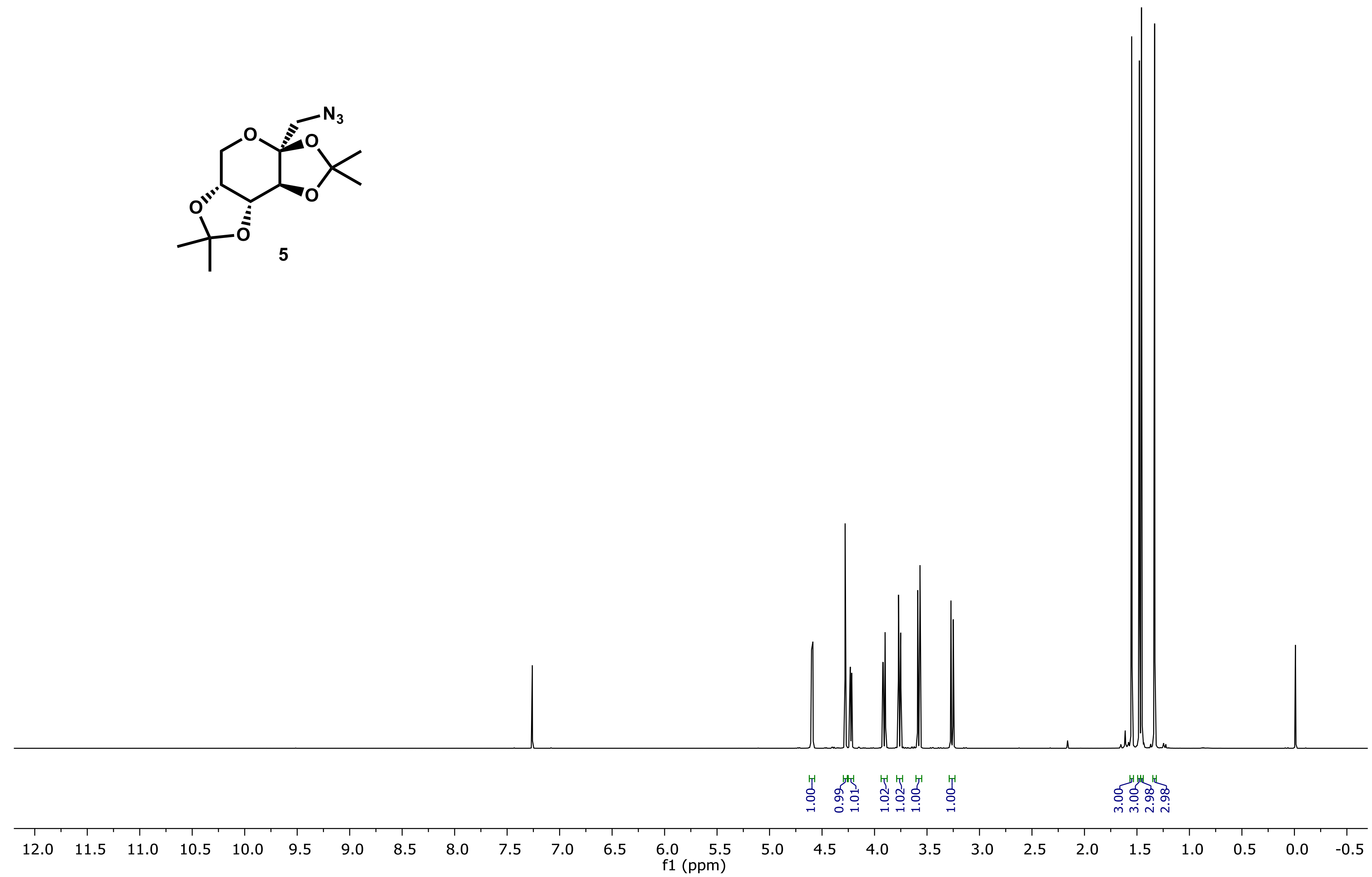

^{1}H NMR Spectrum for Compound **5** ($CDCl_3$, 600 MHz).

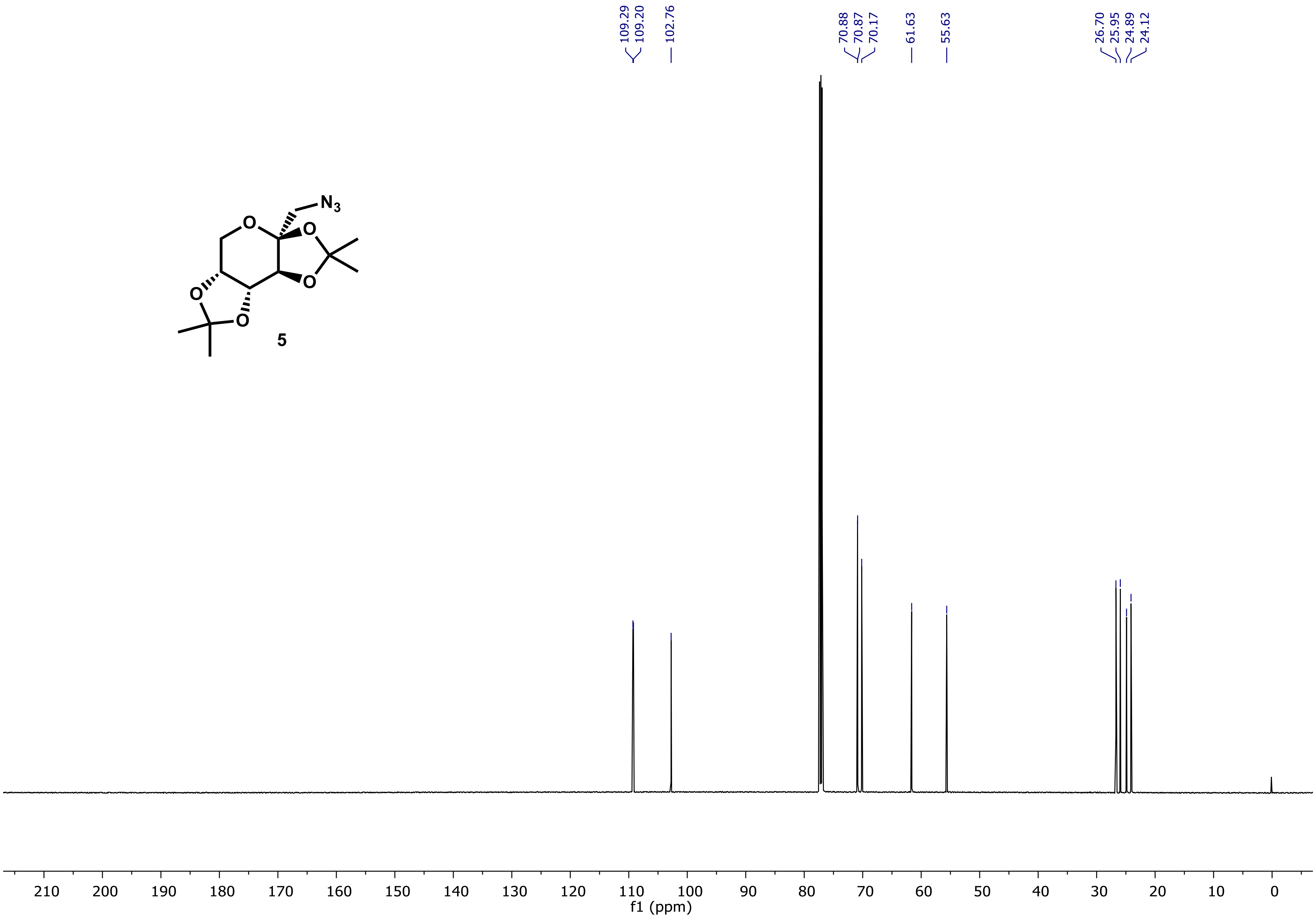

^{13}C NMR Spectrum for Compound **5** ($CDCl_3$, 151 MHz).

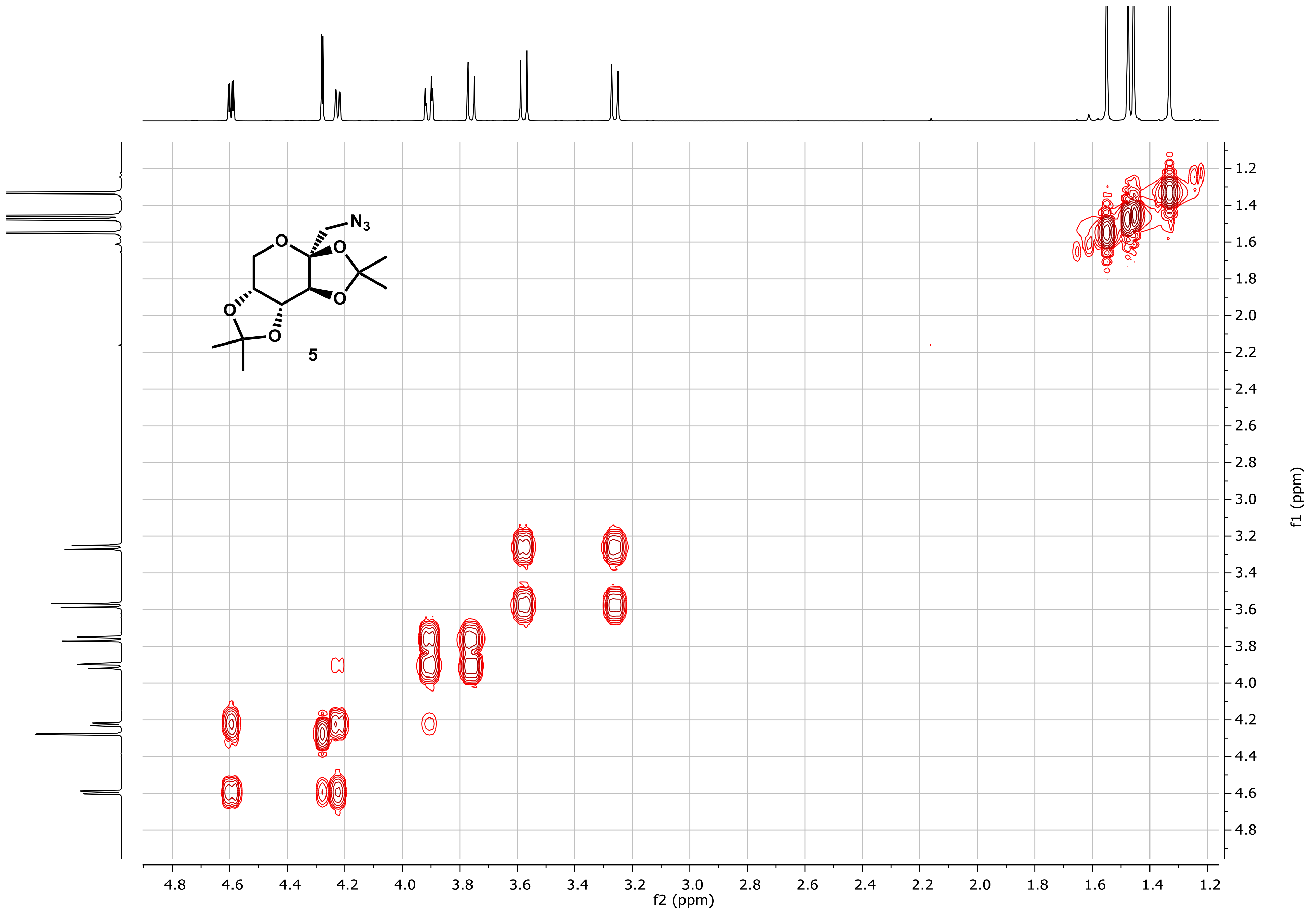

COSY Spectrum for Compound **5** ($CDCl_3$).

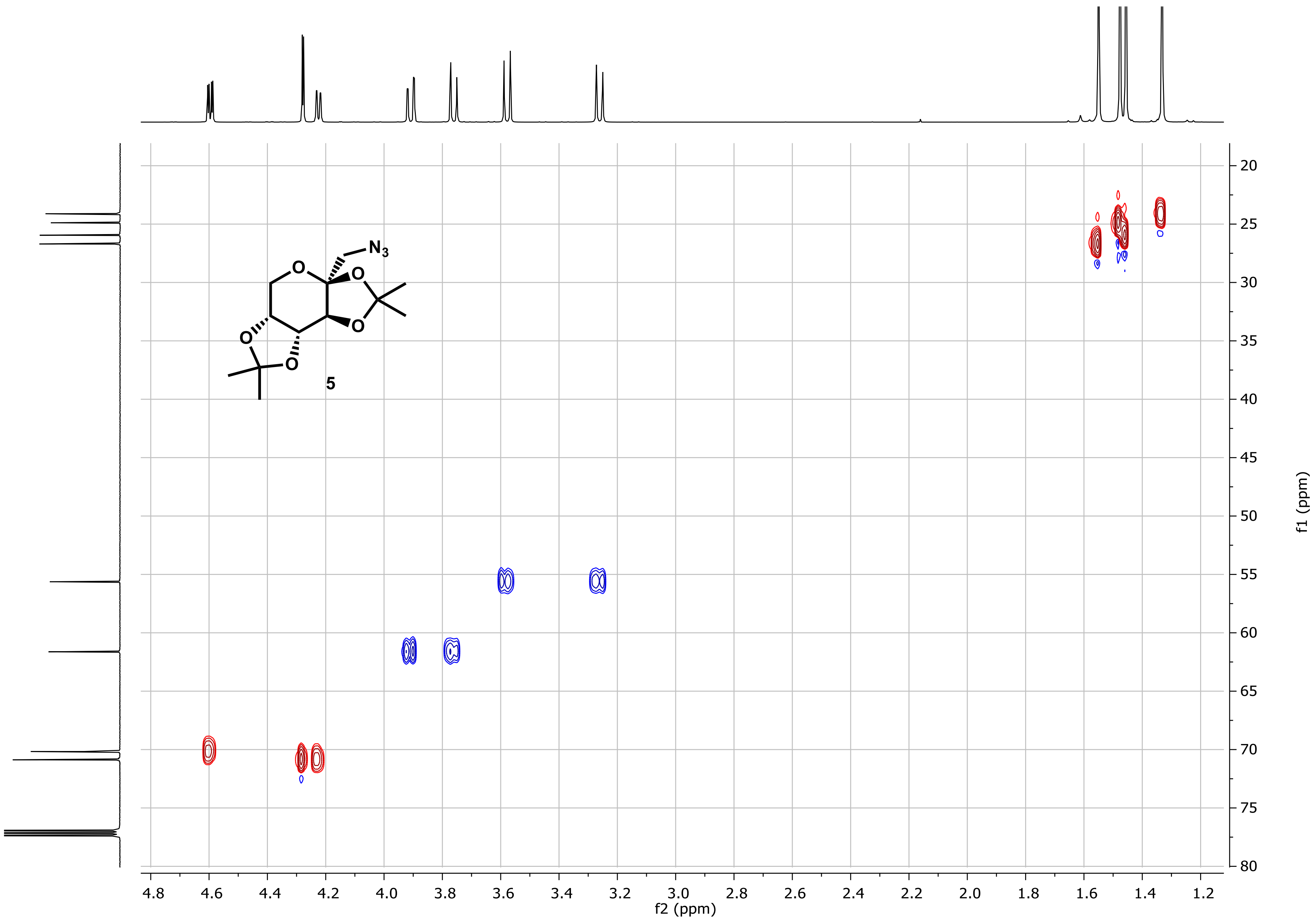

HSQC Spectrum for Compound **5** ($CDCl_3$).

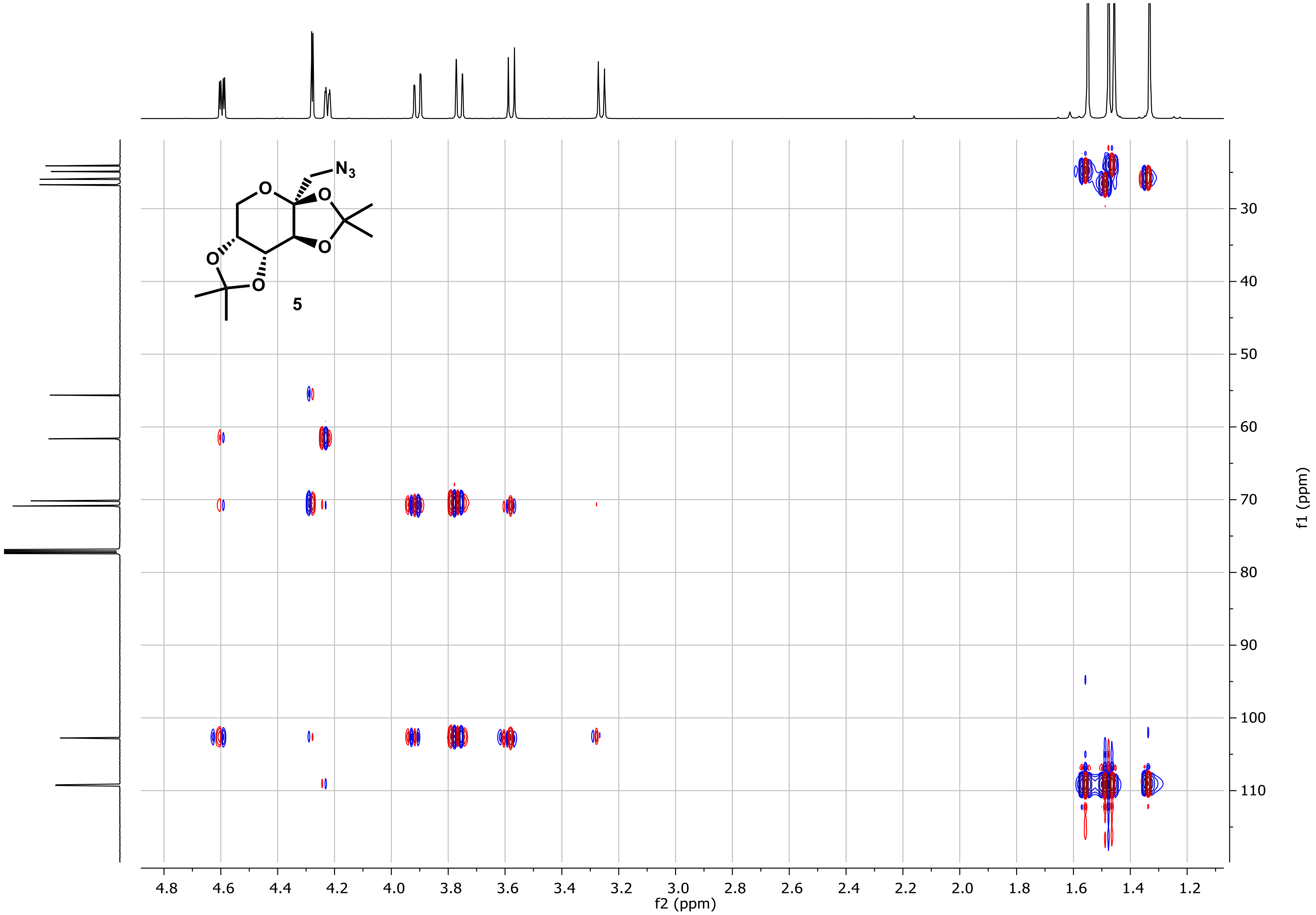

HMBC Spectrum for Compound **5** ($CDCl_3$).

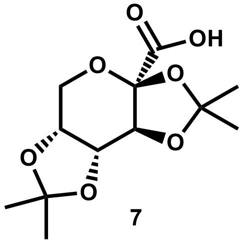

7

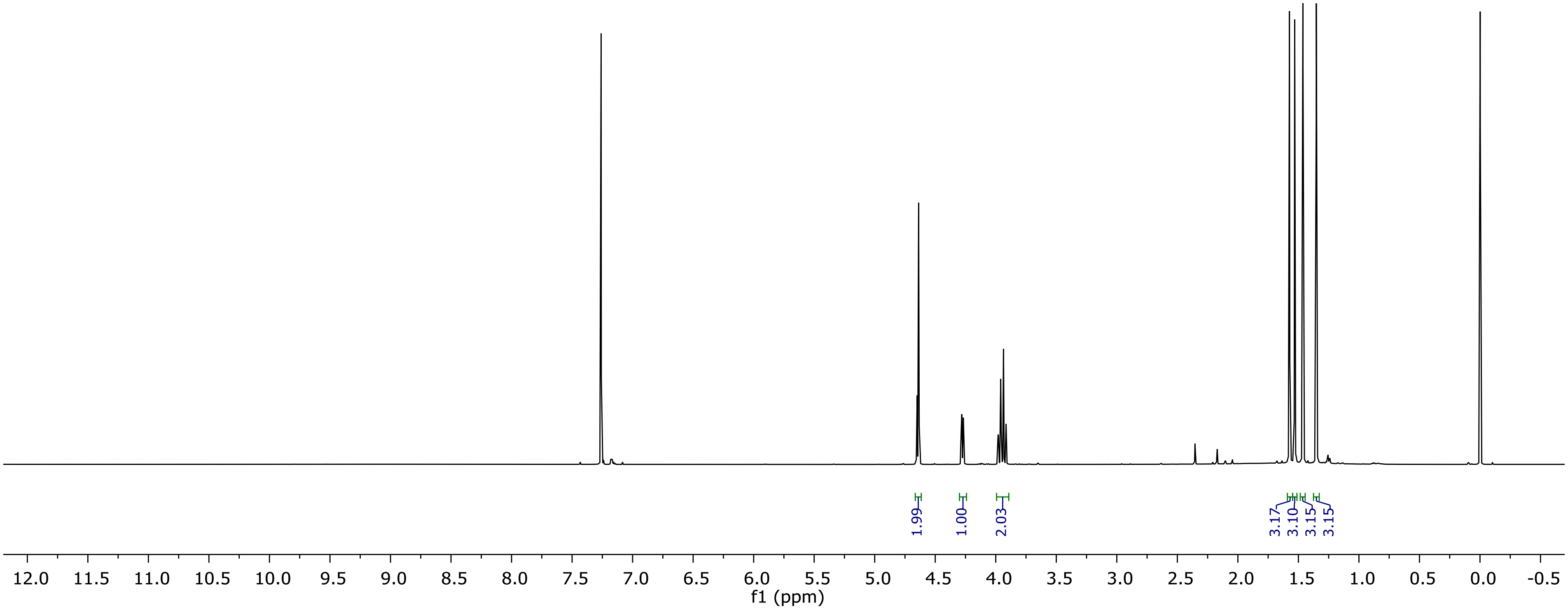

^{1}H NMR Spectrum for Compound **7** ($CDCl_3$, 600 MHz).

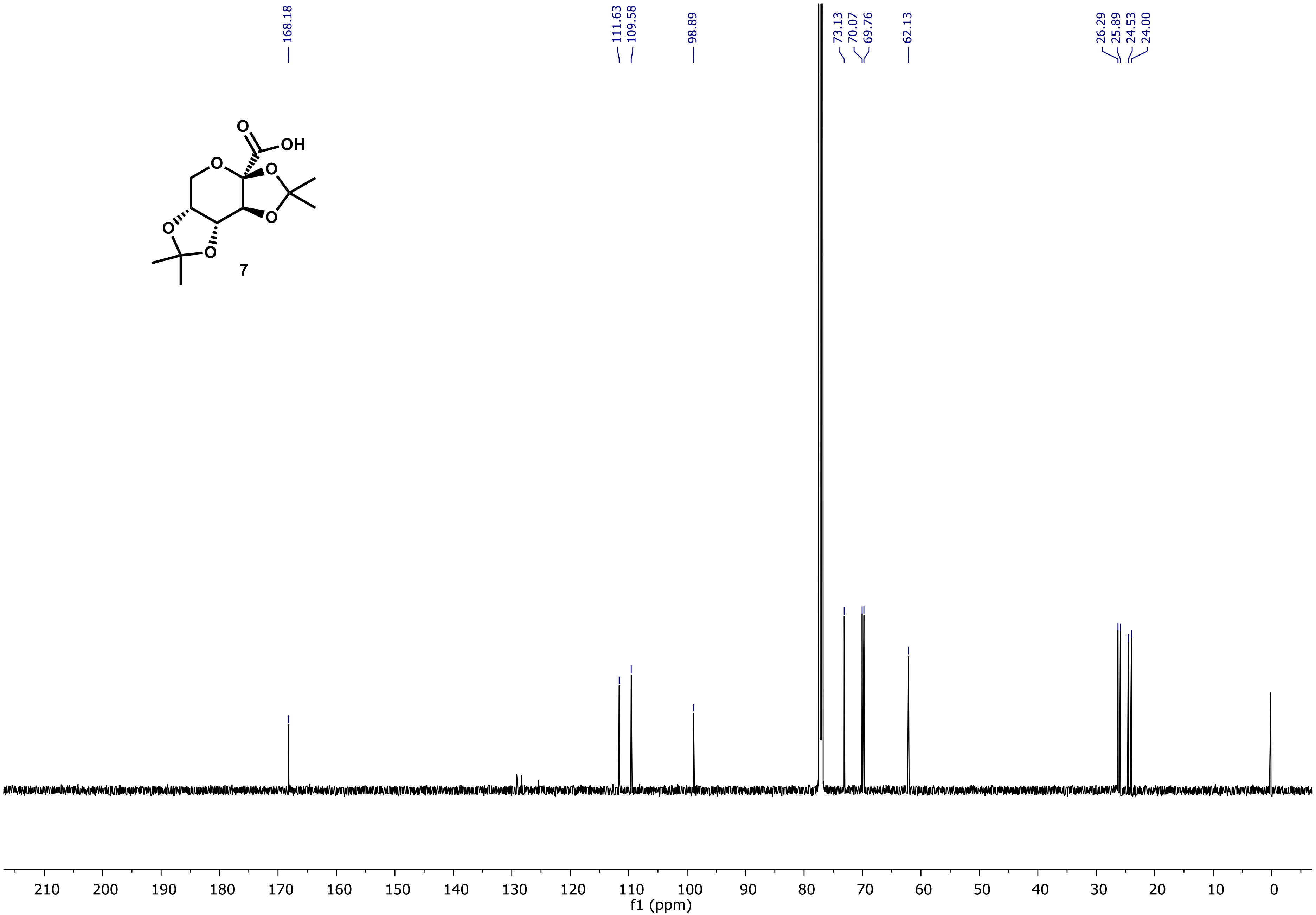

^{13}C NMR Spectrum for Compound **7** ($CDCl_3$, 151 MHz).

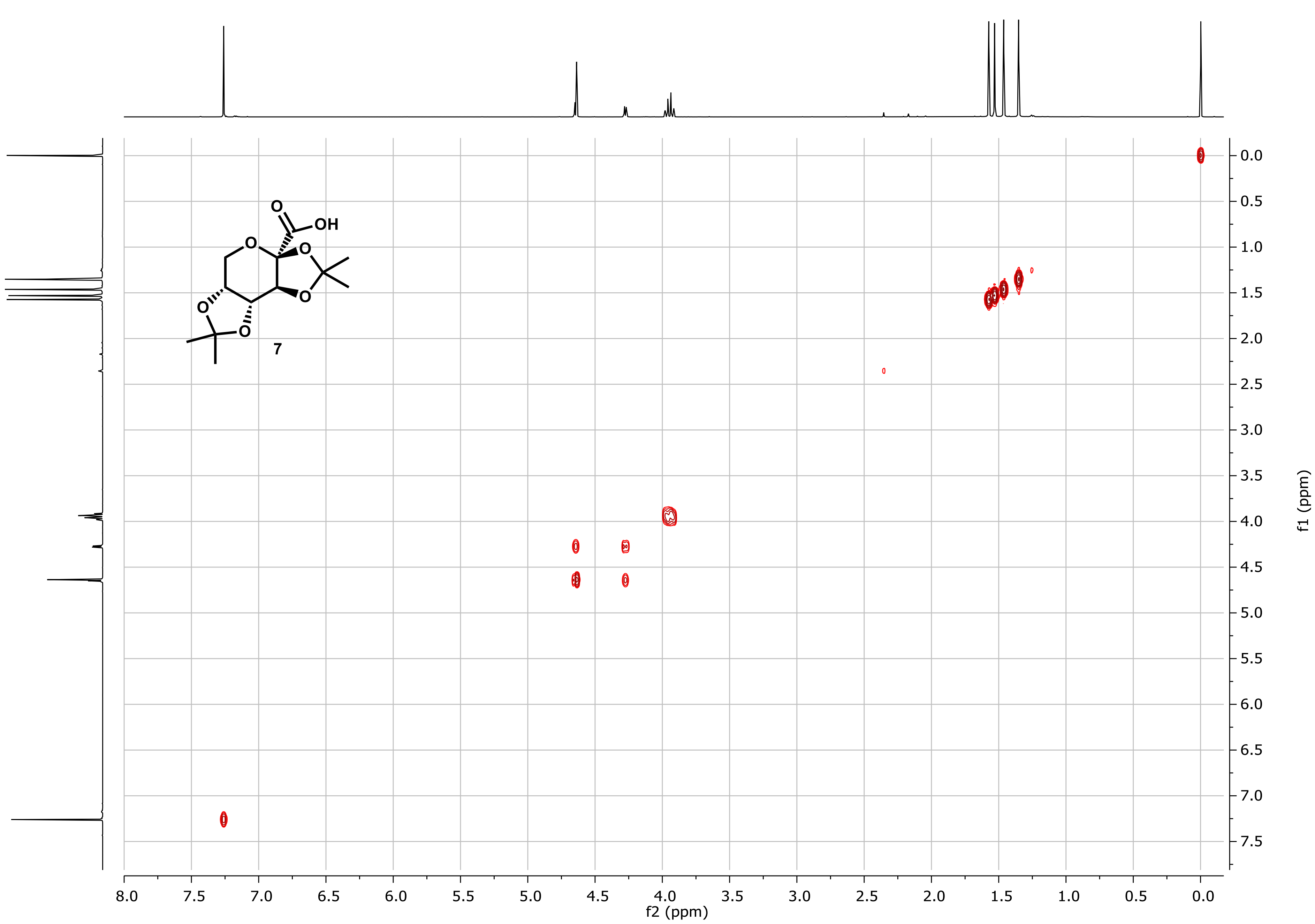

COSY Spectrum for Compound **7** ($CDCl_3$).

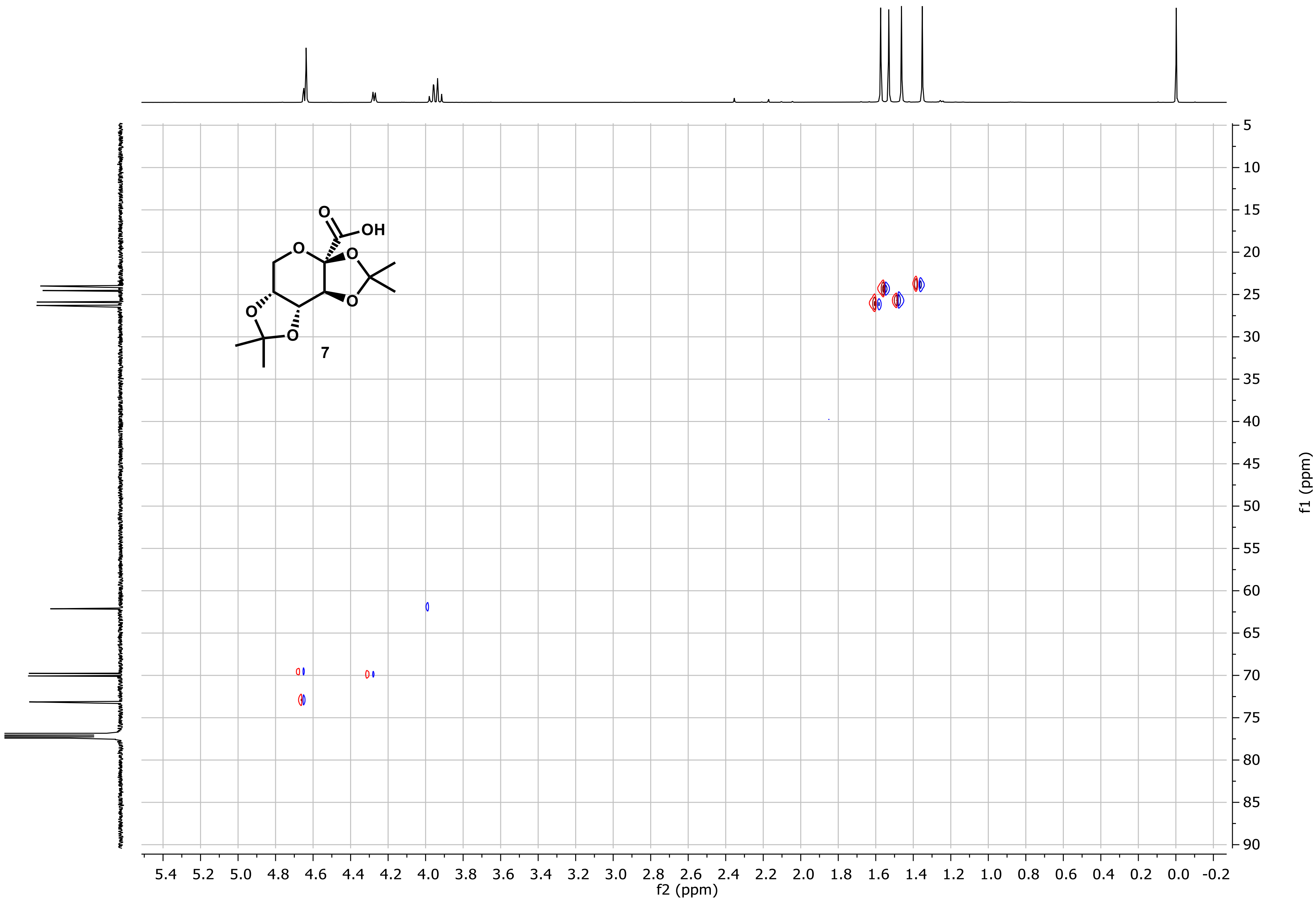

HSQC Spectrum for Compound **7** ($CDCl_3$).

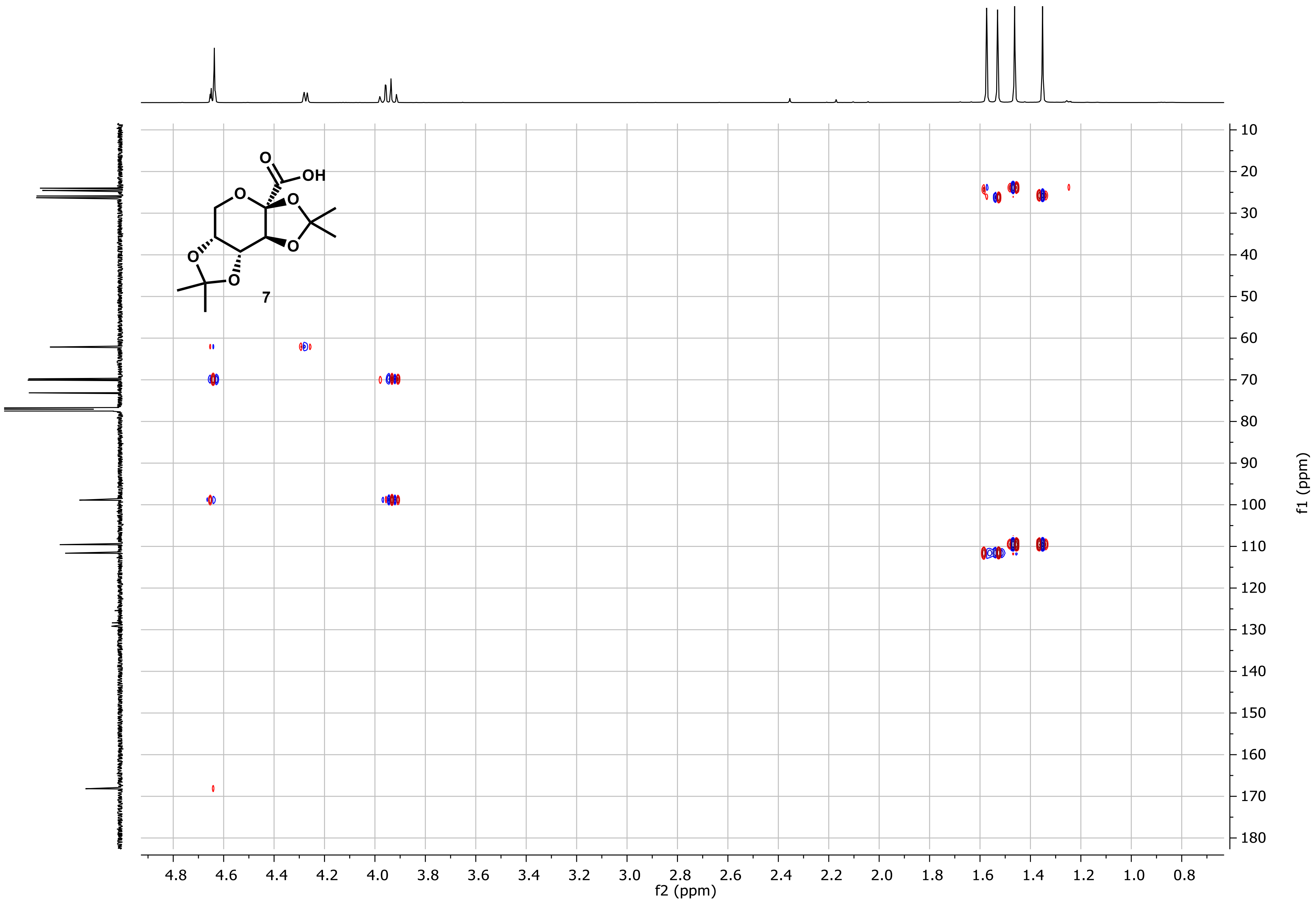

HMBC Spectrum for Compound **7** ($CDCl_3$).

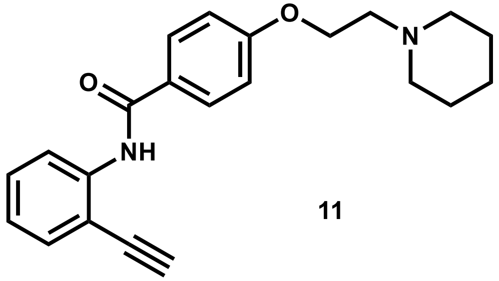

11

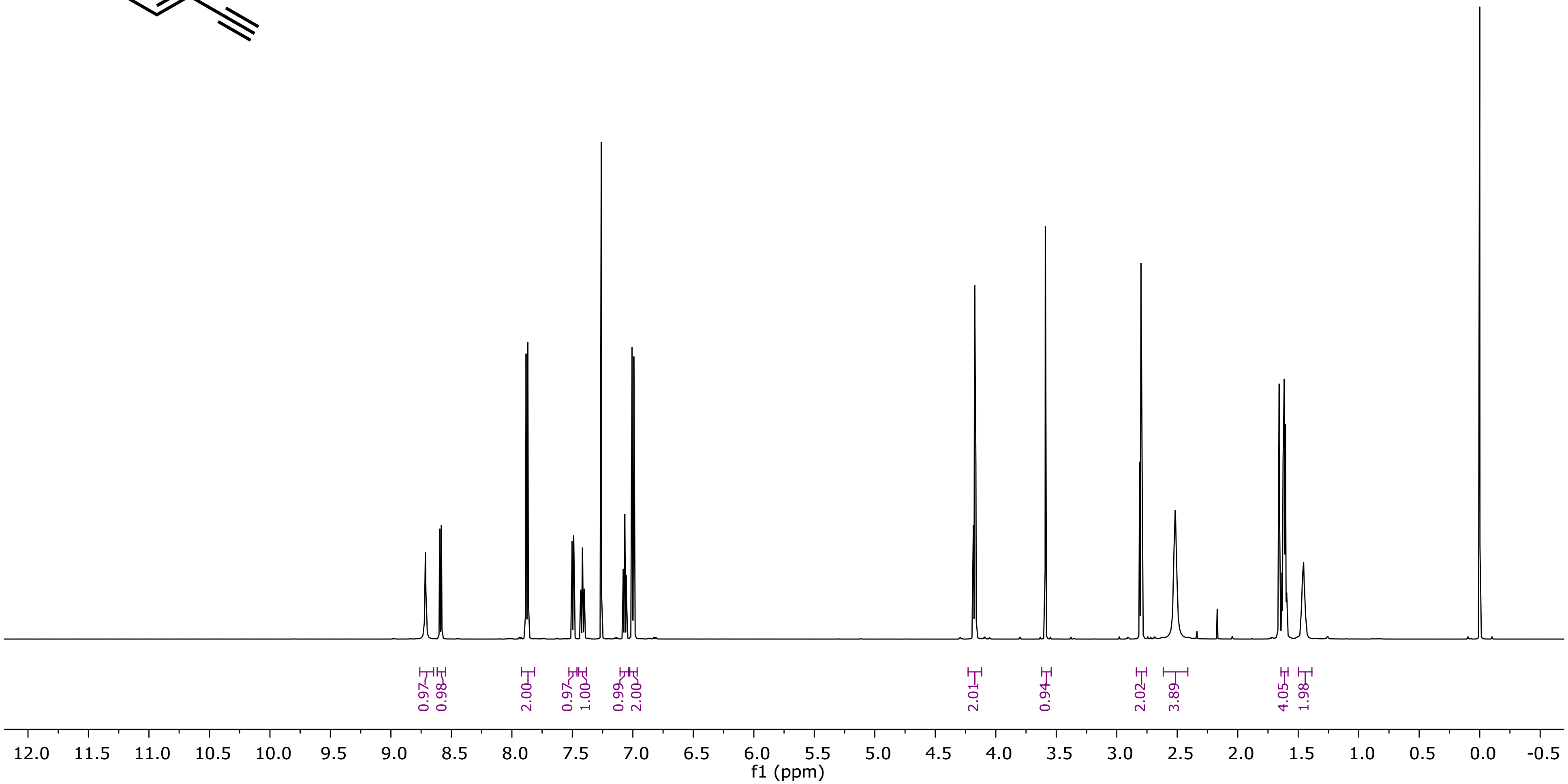

^{1}H NMR Spectrum for Compound **11** ($CDCl_3$, 600 MHz).

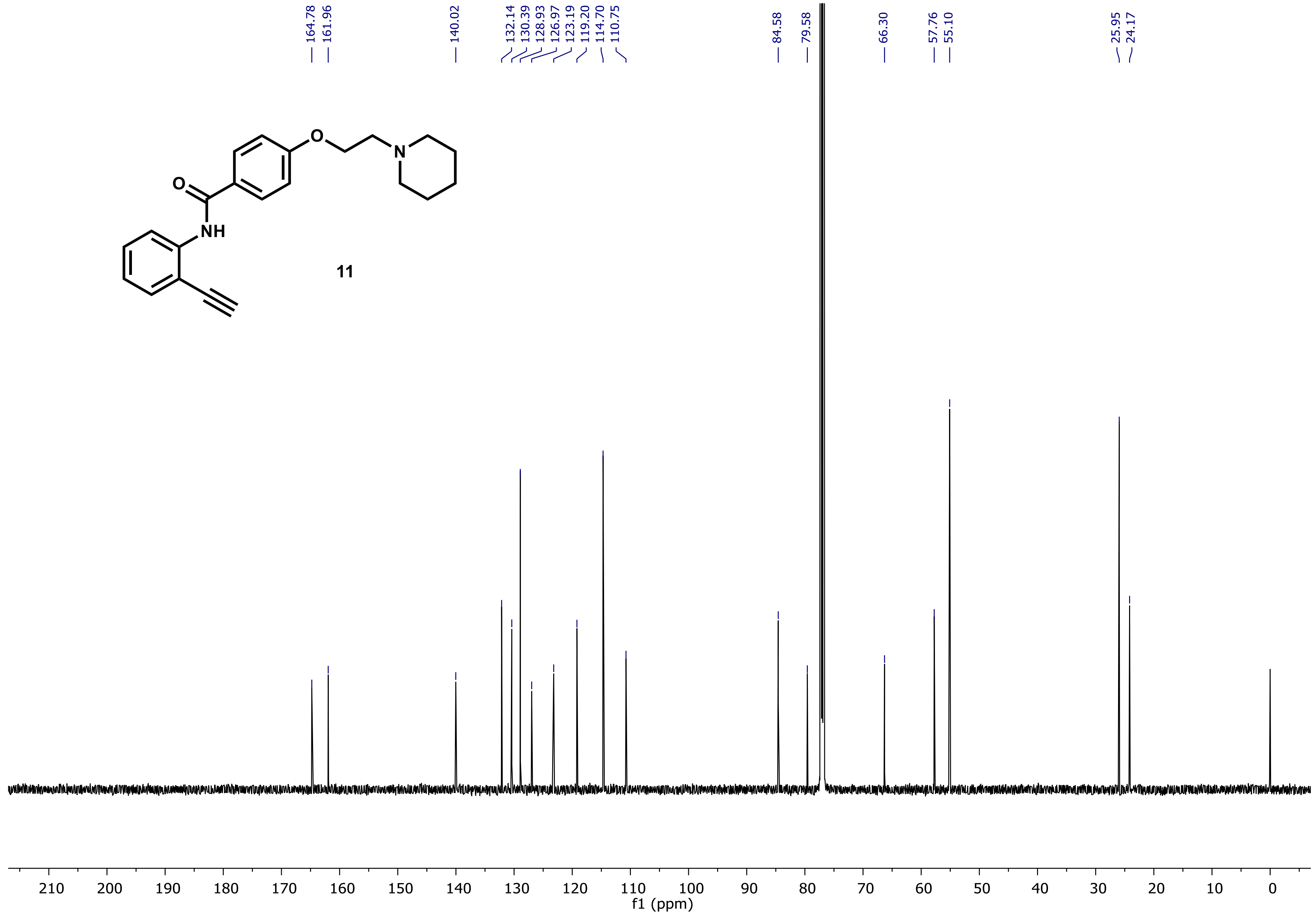

^{13}C NMR Spectrum for Compound **11** ($CDCl_3$, 151 MHz).

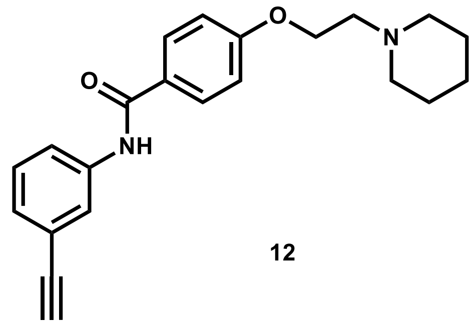

12

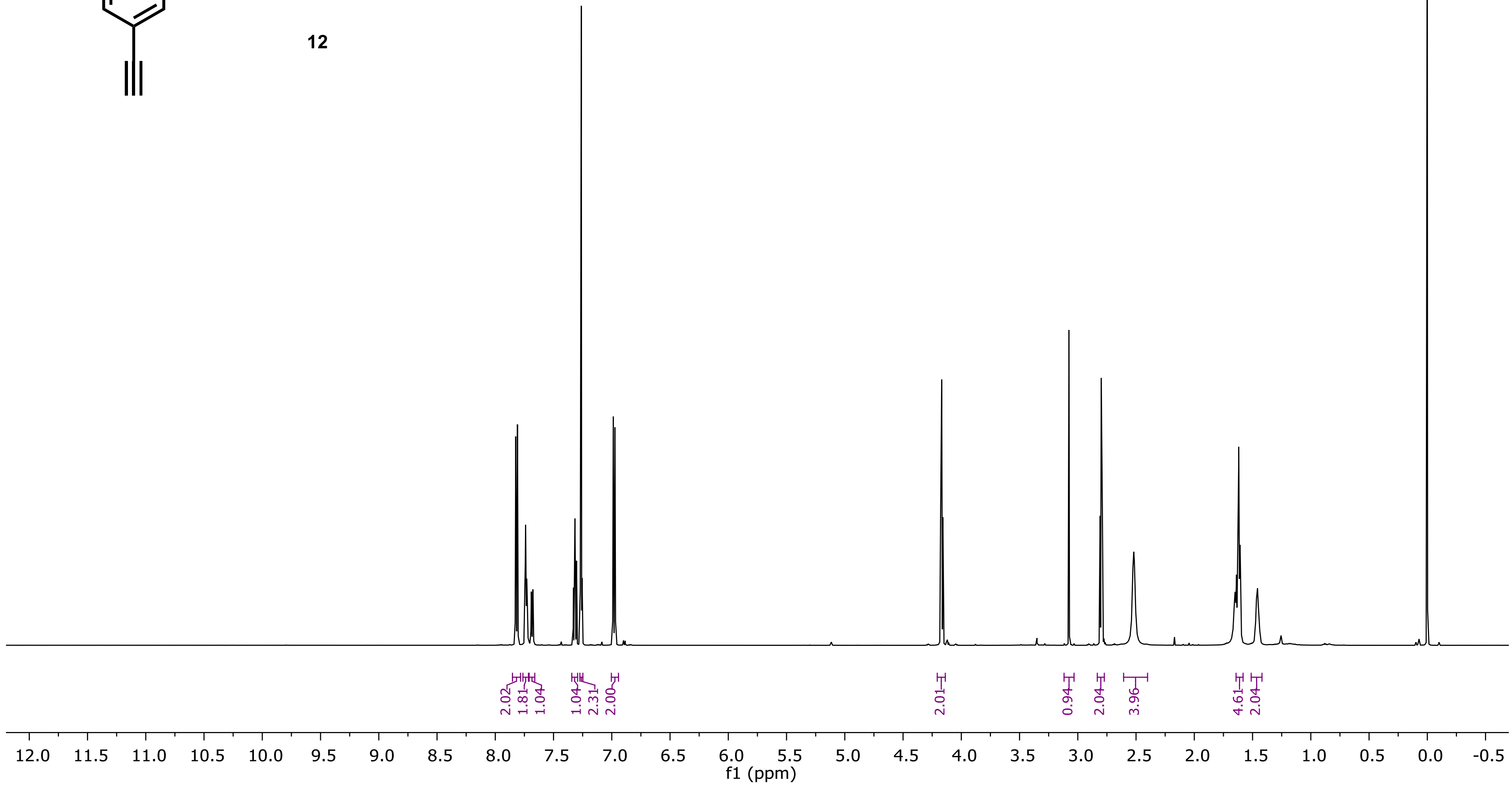

^{1}H NMR Spectrum for Compound **12** ($CDCl_3$, 600 MHz).

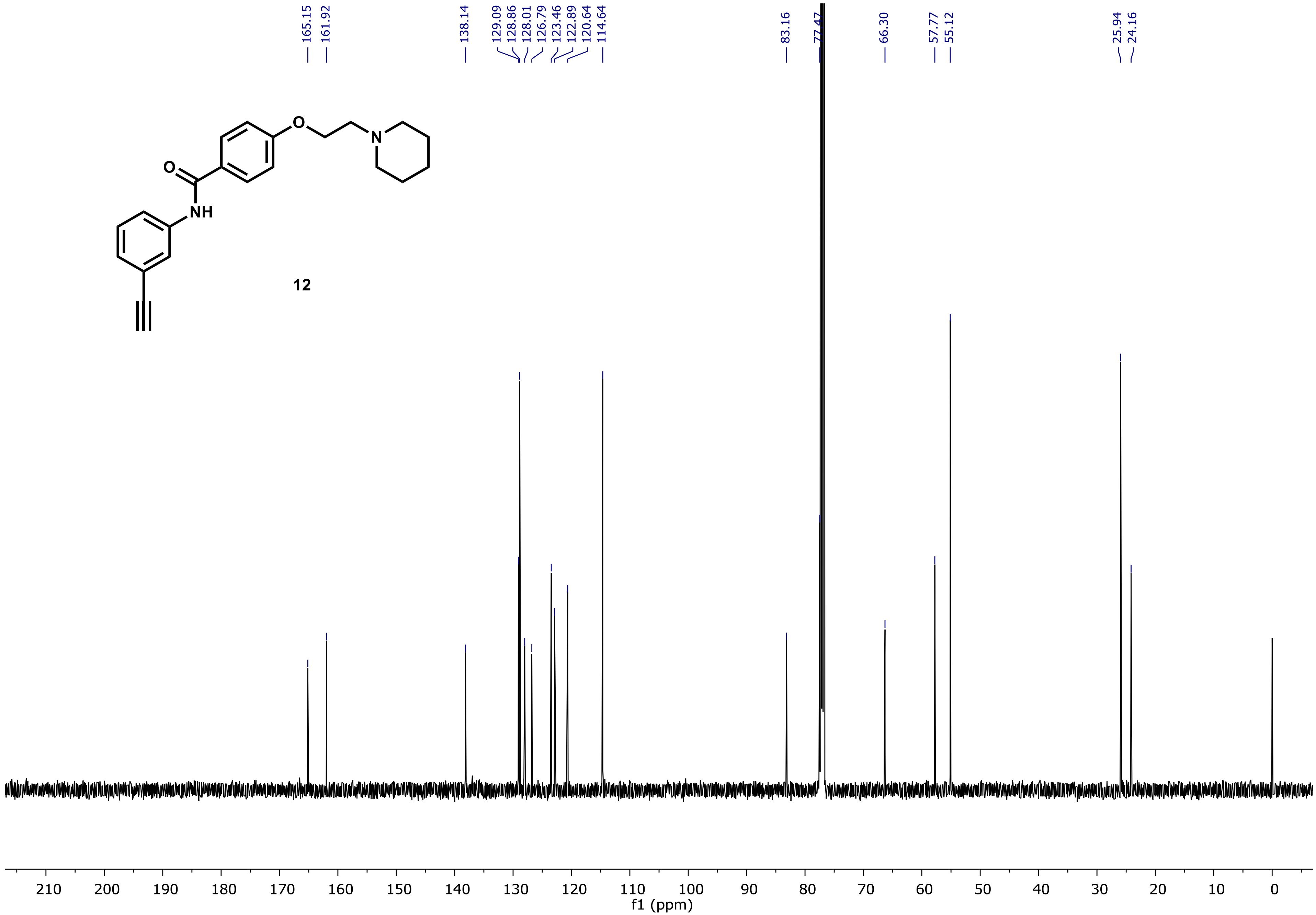

^{13}C NMR Spectrum for Compound **12** ($CDCl_3$, 151 MHz).

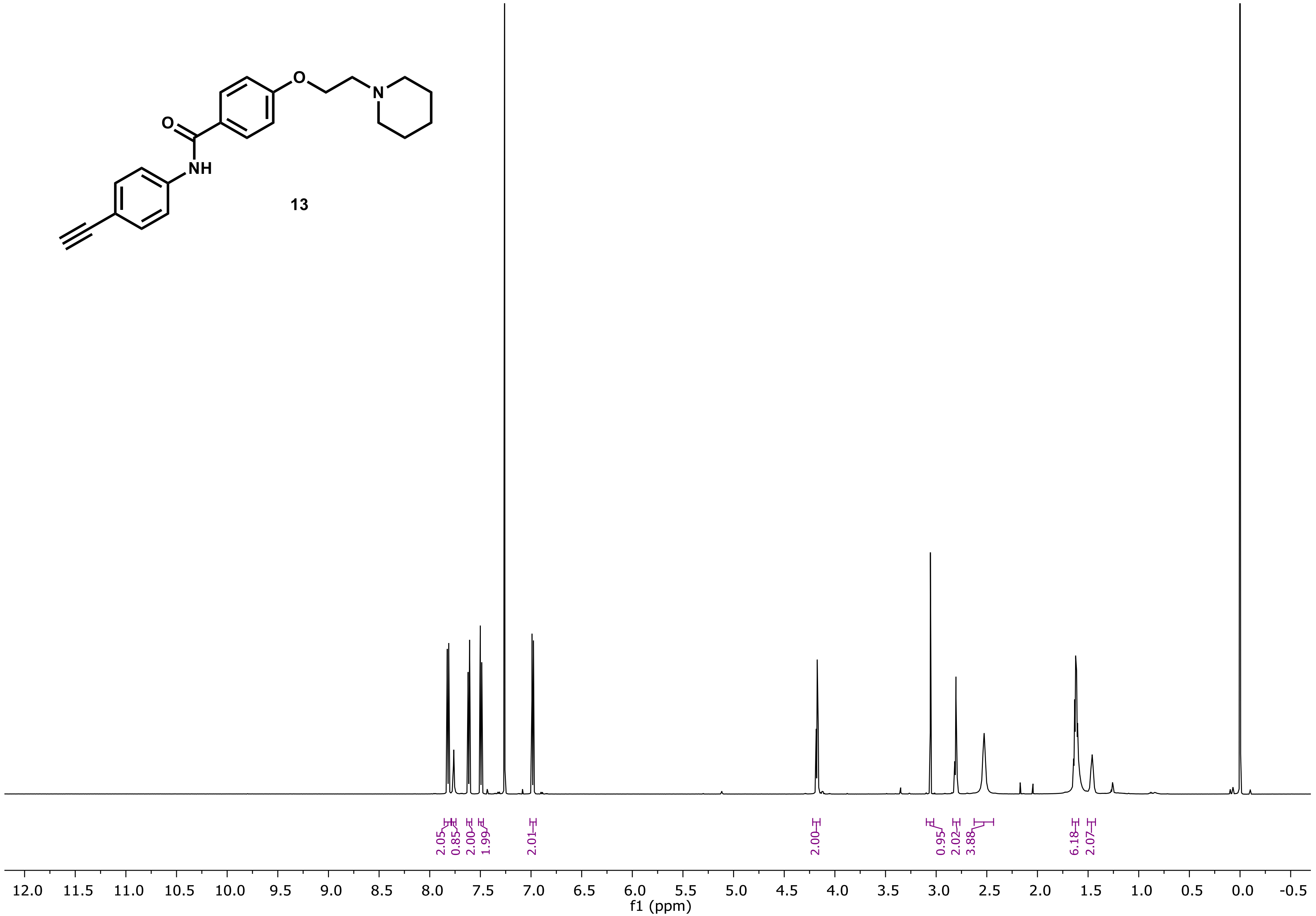

^{1}H NMR Spectrum for Compound **13** ($CDCl_3$, 600 MHz).

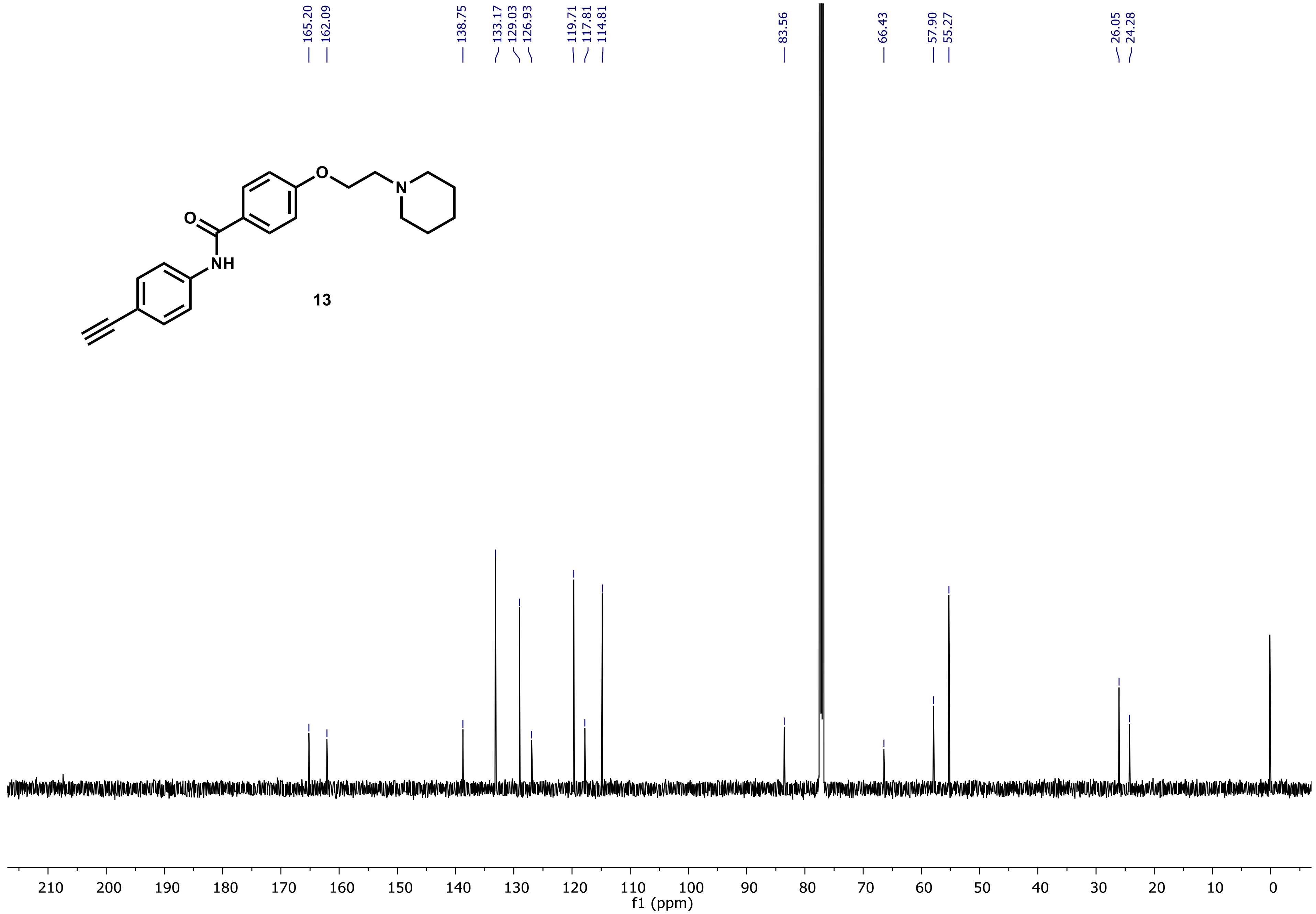

^{13}C NMR Spectrum for Compound **13** ($CDCl_3$, 151 MHz).

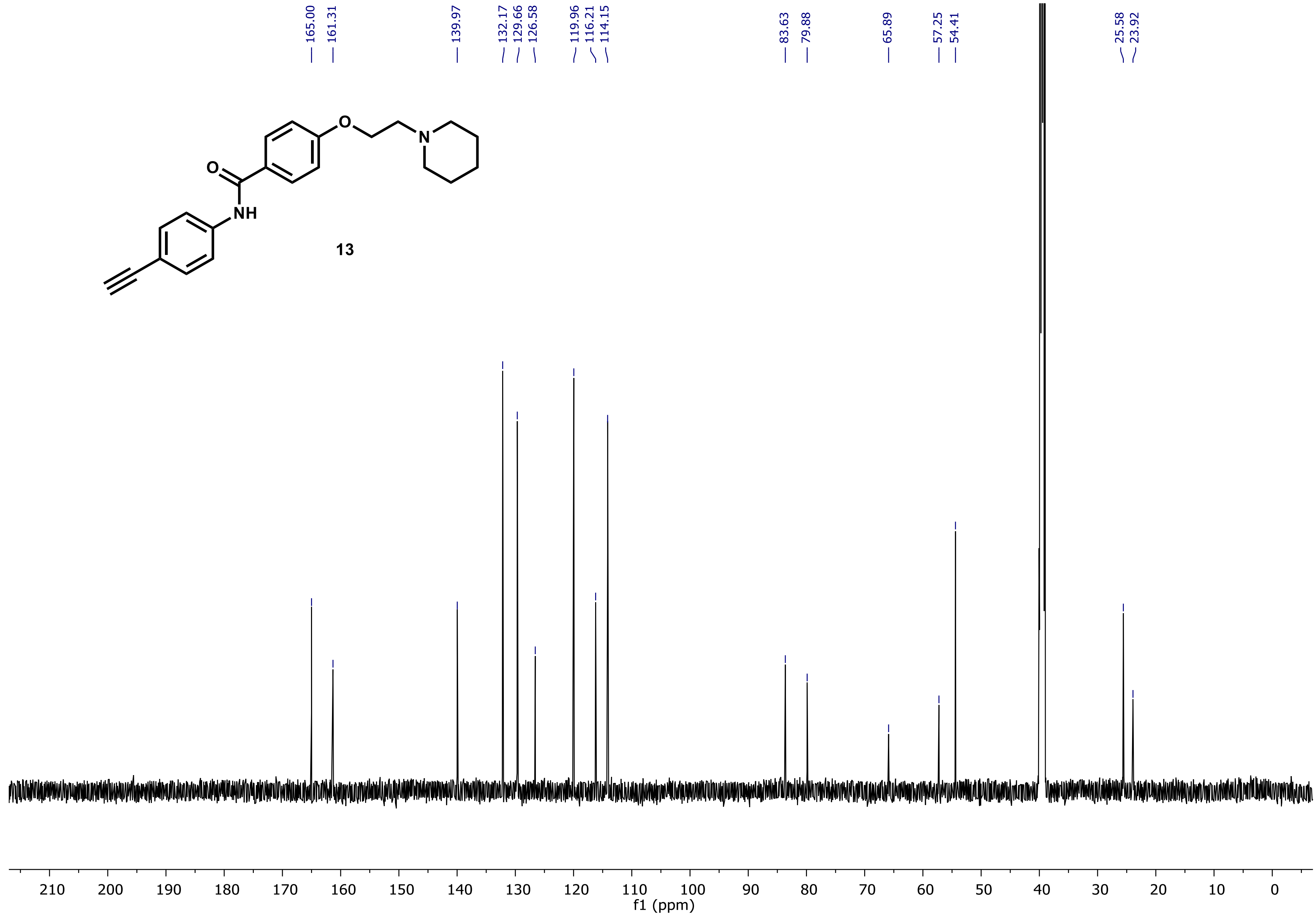

^{13}C NMR Spectrum for Compound **13** (DMSO-*d6*, 151 MHz).

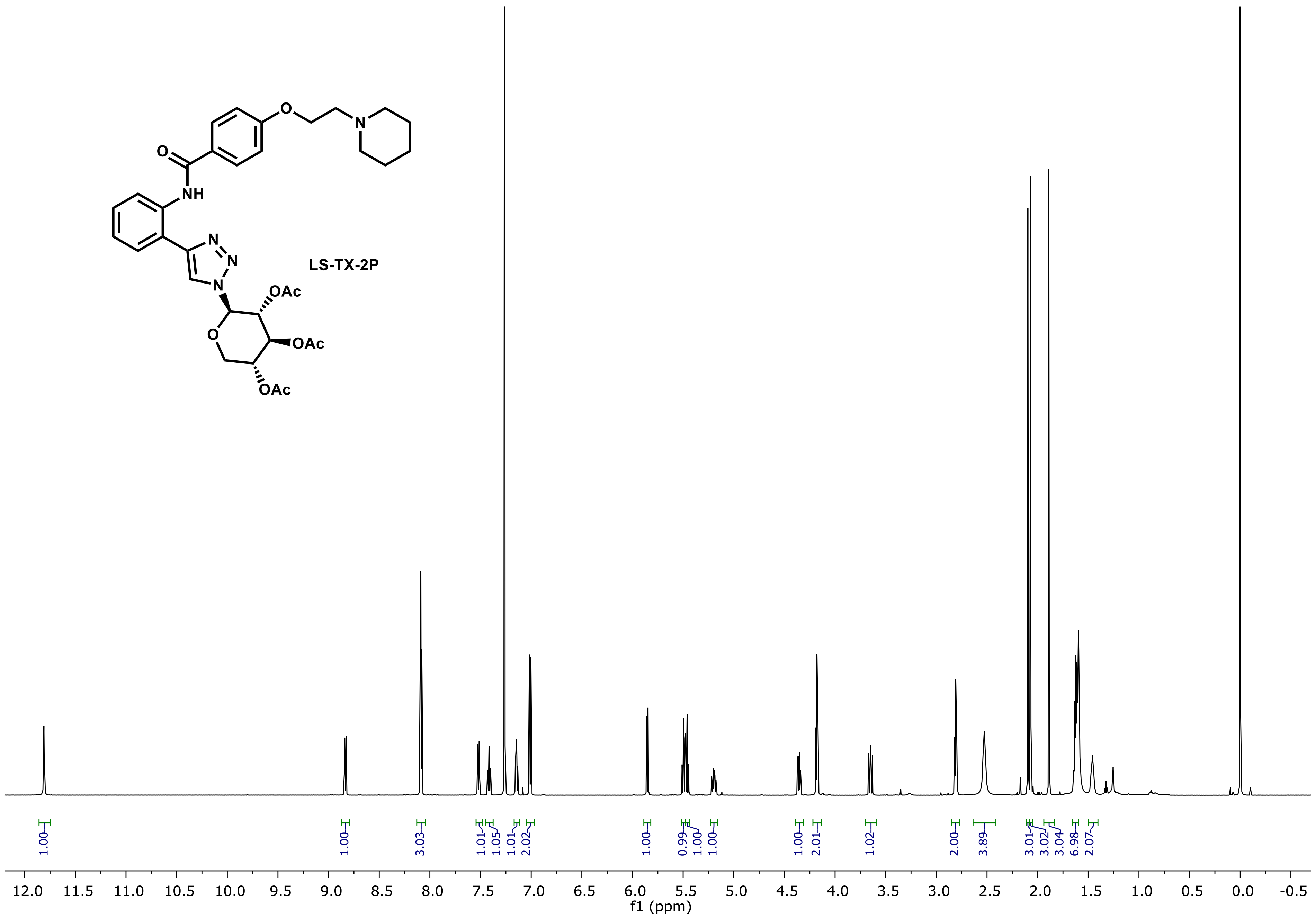

^{1}H NMR Spectrum for **LS-TX-2P** ($CDCl_3$, 600 MHz).

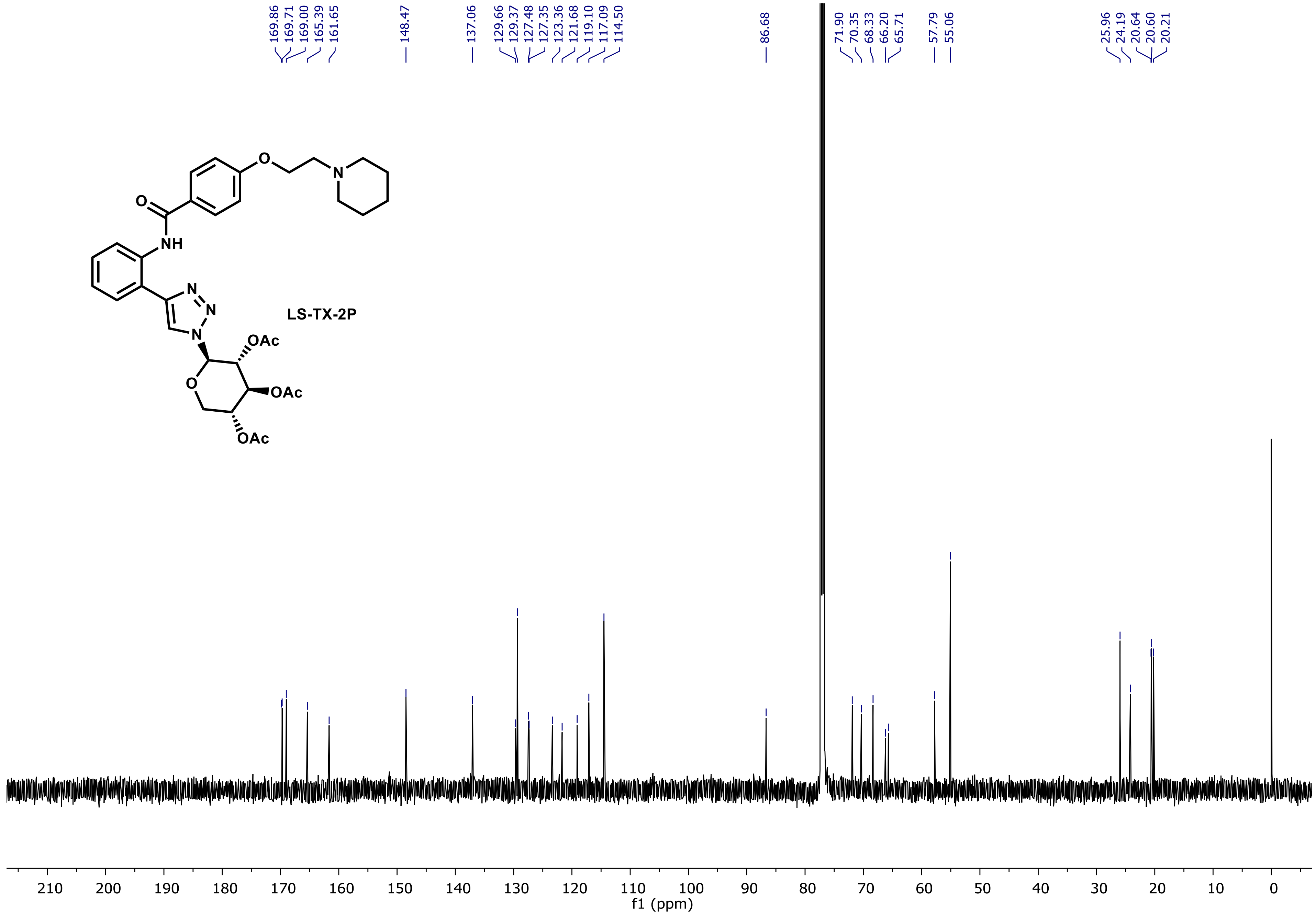

^{13}C NMR Spectrum for **LS-TX-2P** ($CDCl_3$, 151 MHz).

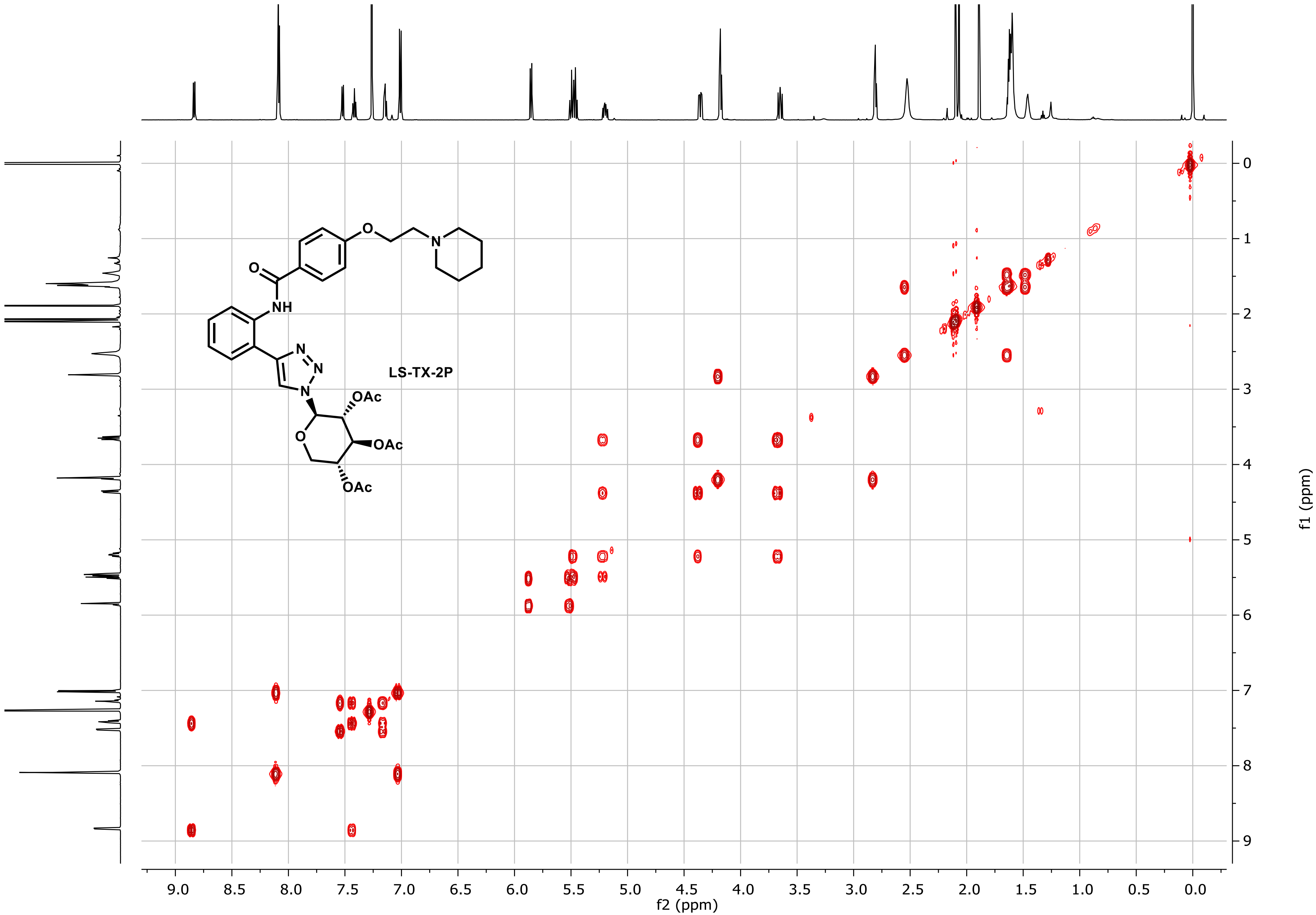

COSY Spectrum for **LS-TX-2P** ($CDCl_3$).

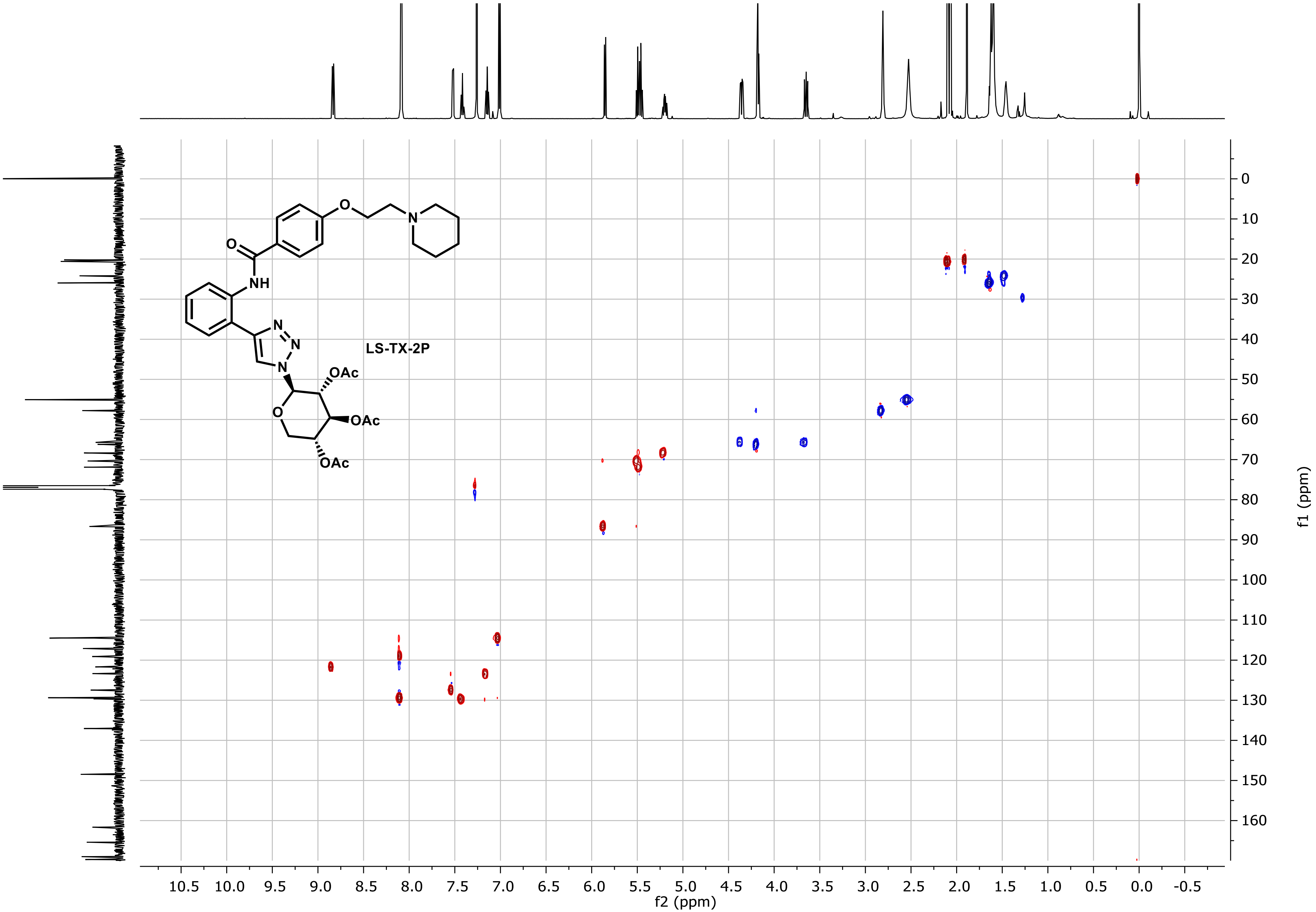

HSQC Spectrum for **LS-TX-2P** ($CDCl_3$).

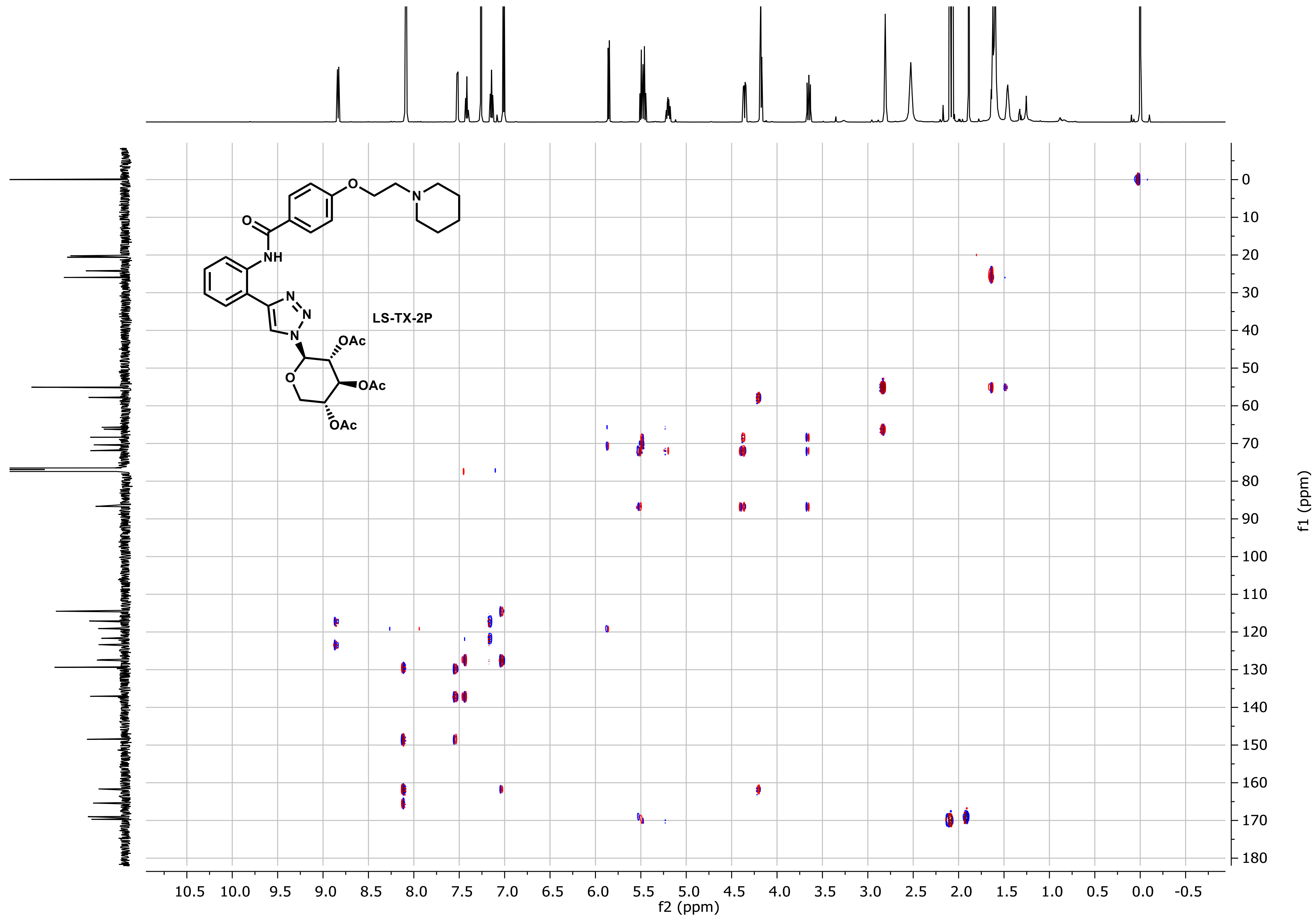

HMBC Spectrum for **LS-TX-2P** ($CDCl_3$).

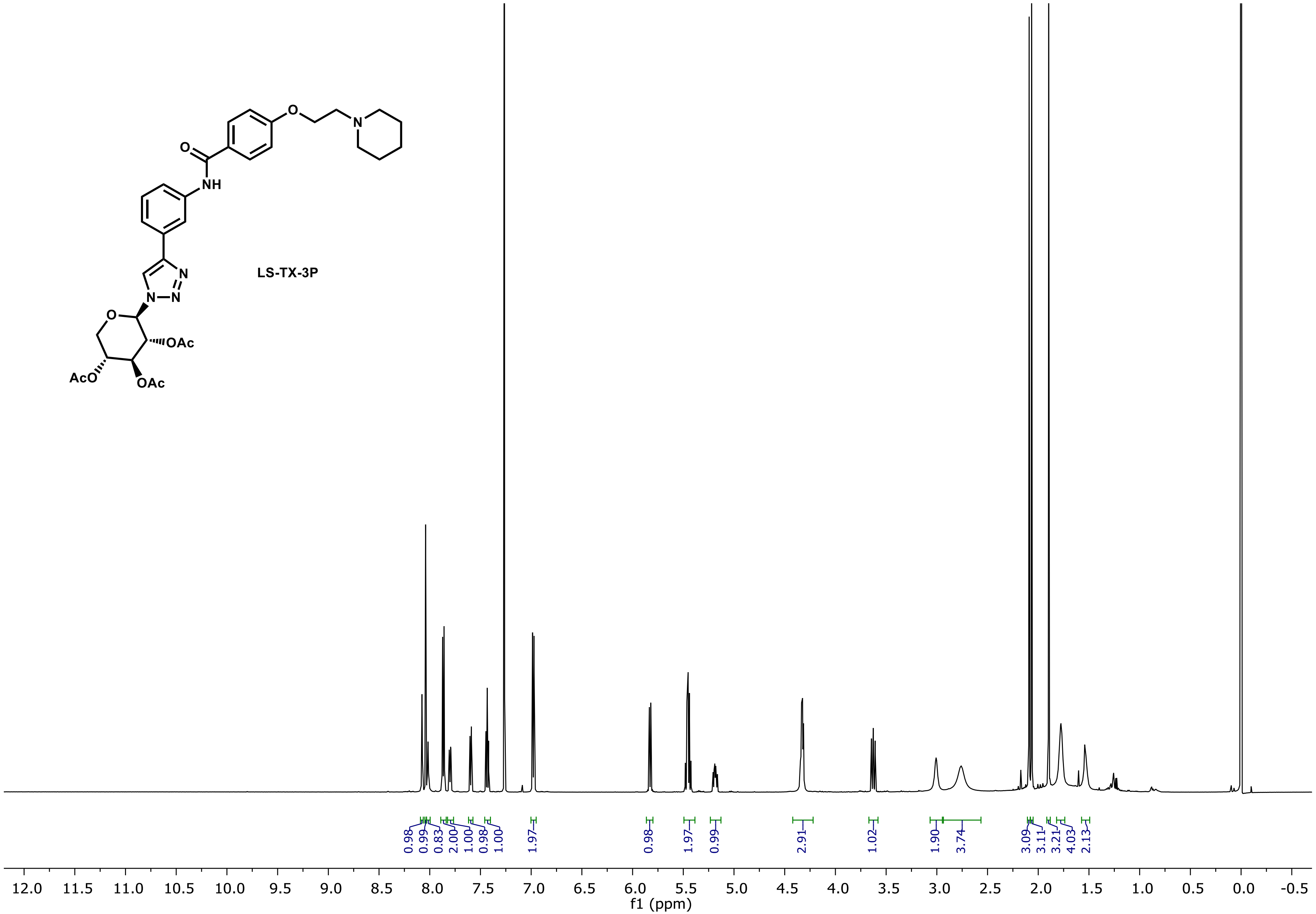

^{1}H NMR Spectrum for **LS-TX-3P** ($CDCl_3$, 600 MHz).

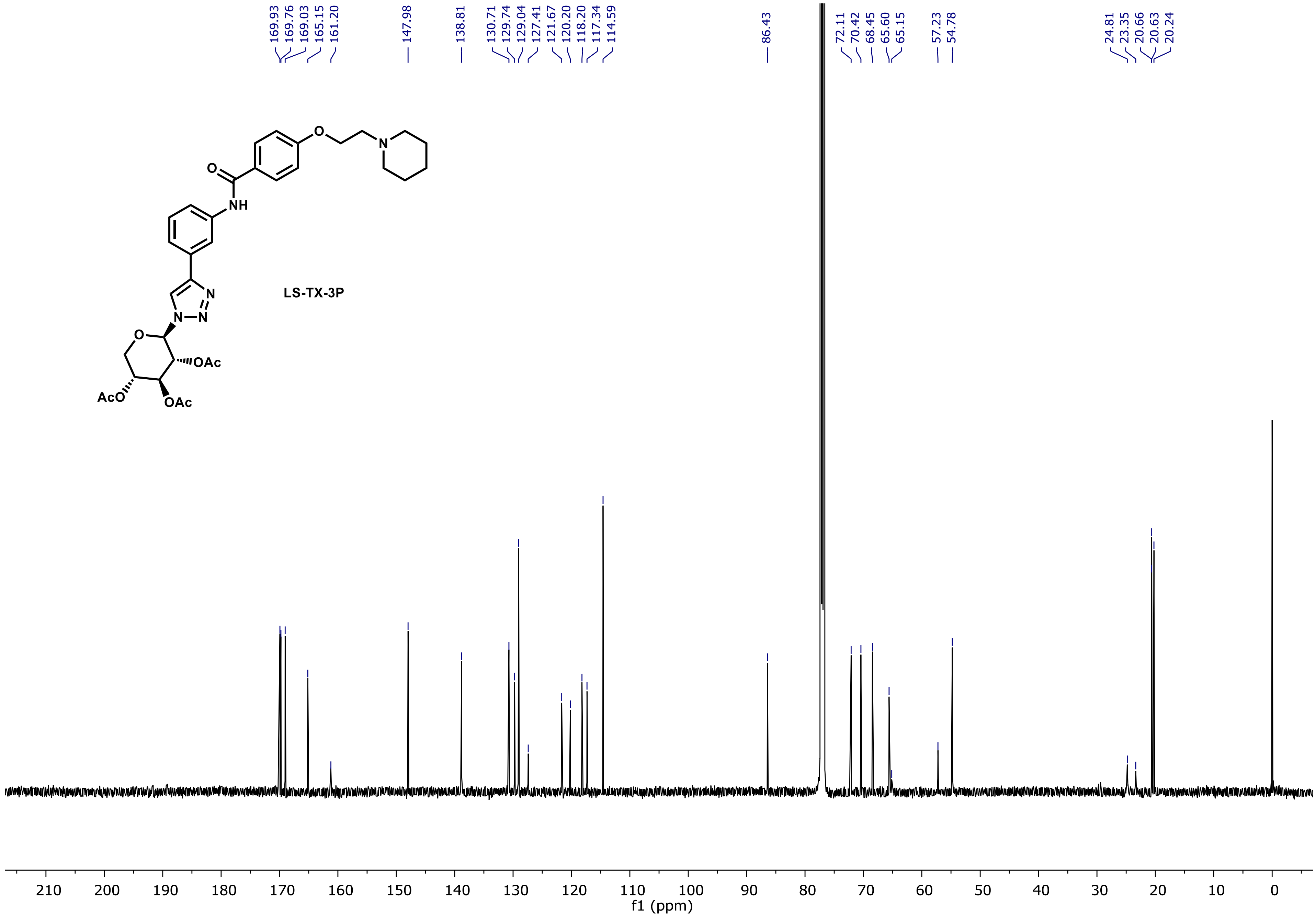

^{13}C NMR Spectrum for **LS-TX-3P** ($CDCl_3$, 151 MHz).

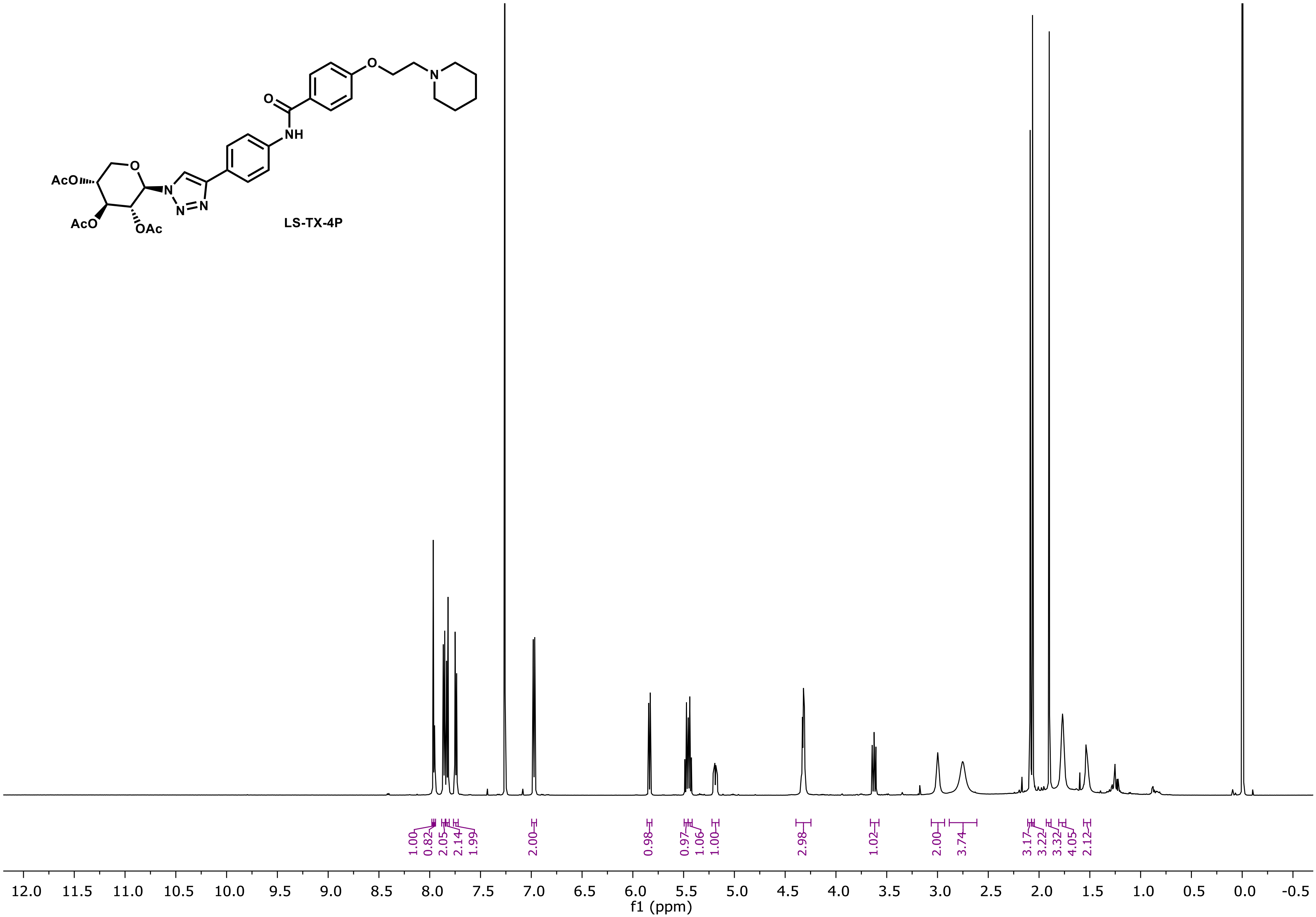

^{1}H NMR Spectrum for **LS-TX-4P** ($CDCl_3$, 600 MHz).

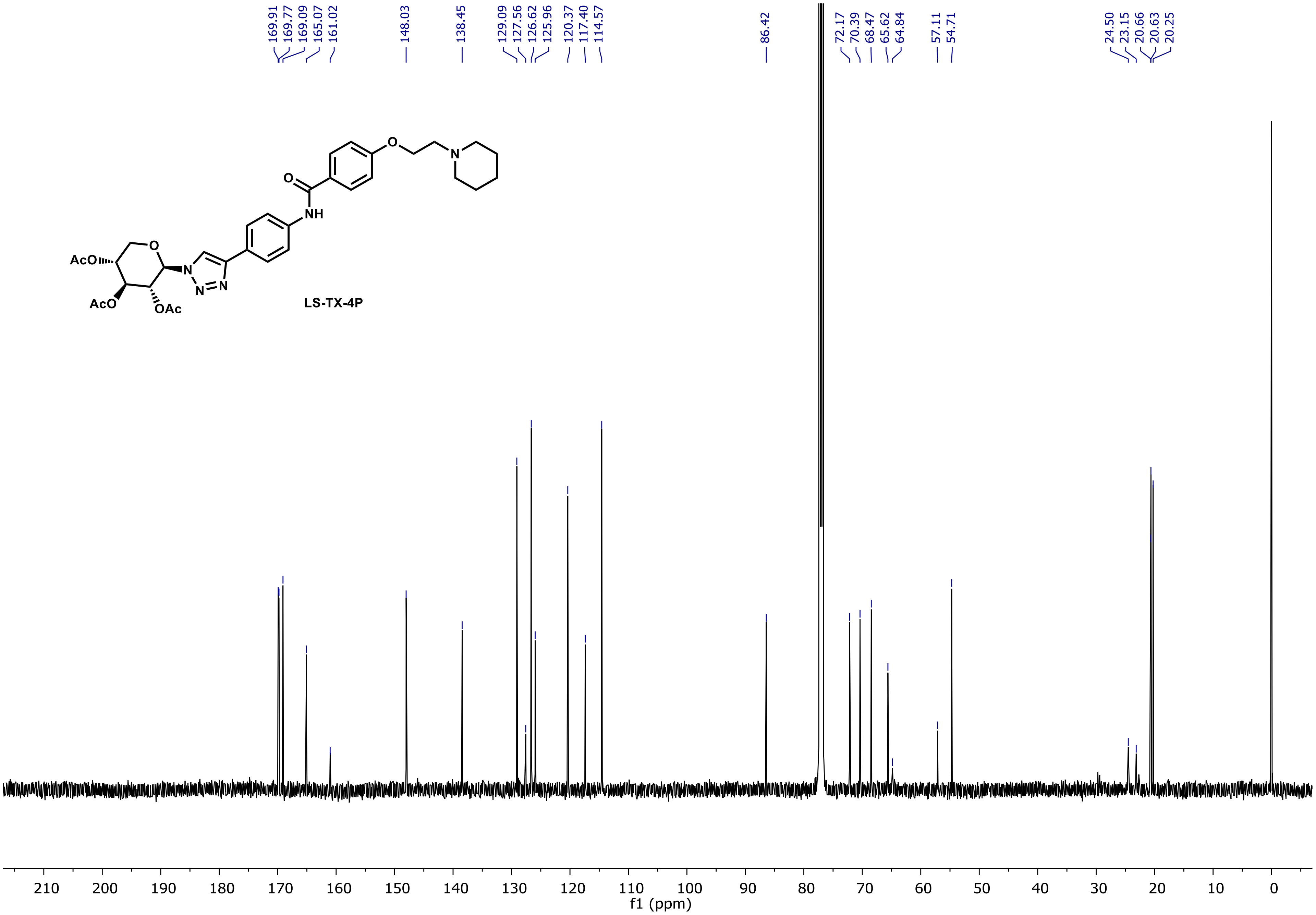

^{13}C NMR Spectrum for **LS-TX-4P** ($CDCl_3$, 151 MHz).

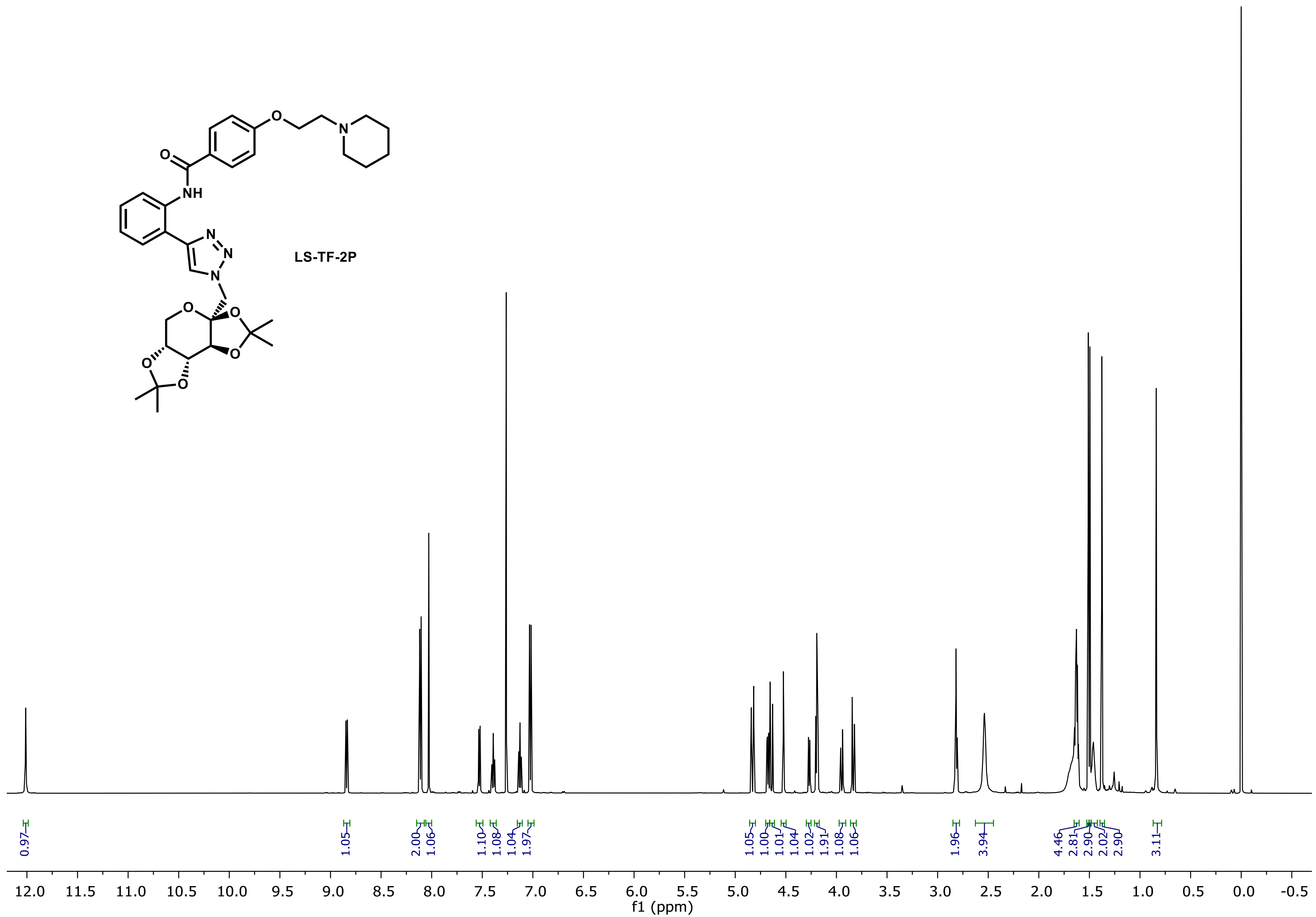

^{1}H NMR Spectrum for **LS-TF-2P** ($CDCl_3$, 600 MHz).

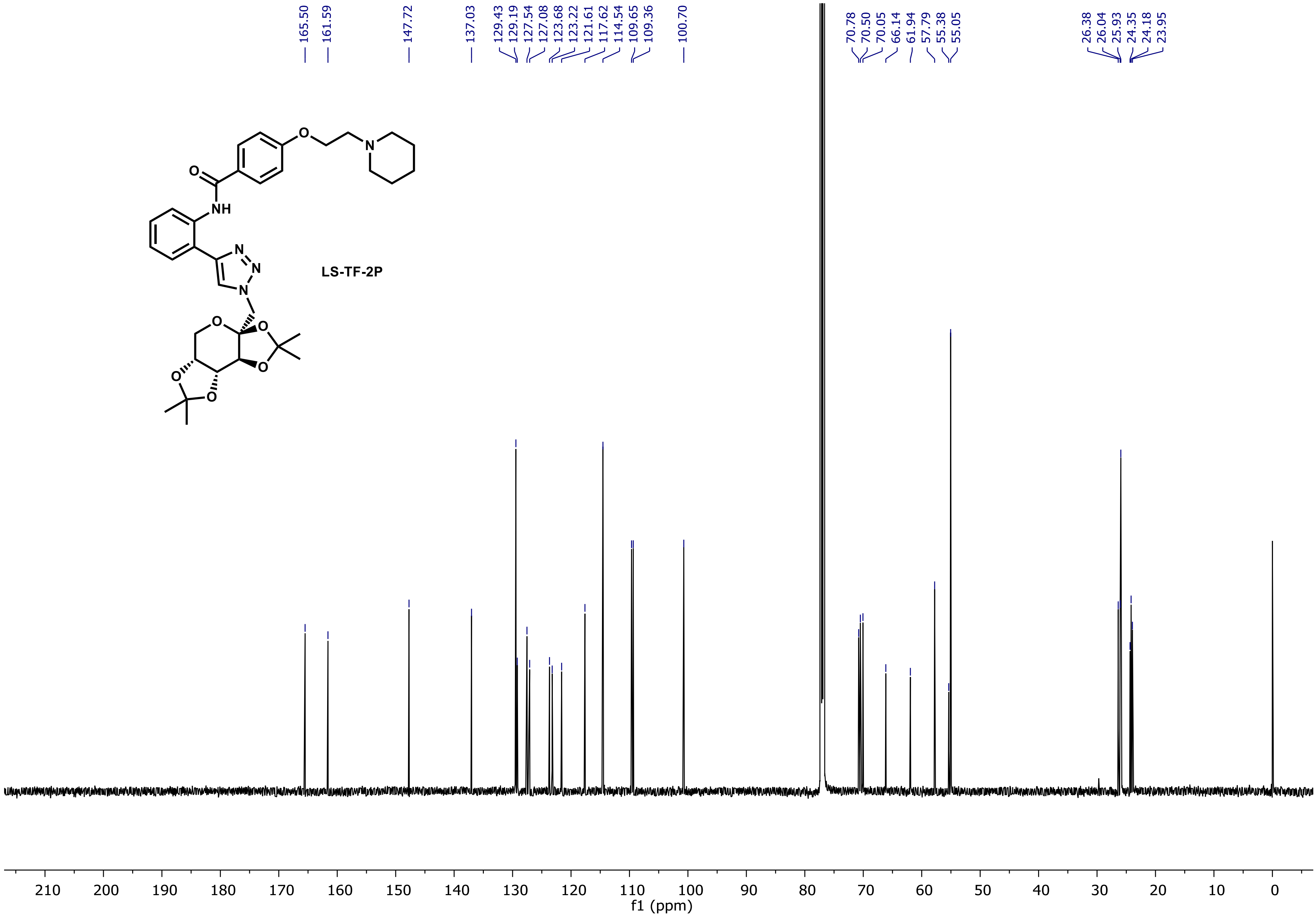

^{13}C NMR Spectrum for **LS-TF-2P** ($CDCl_3$, 151 MHz).

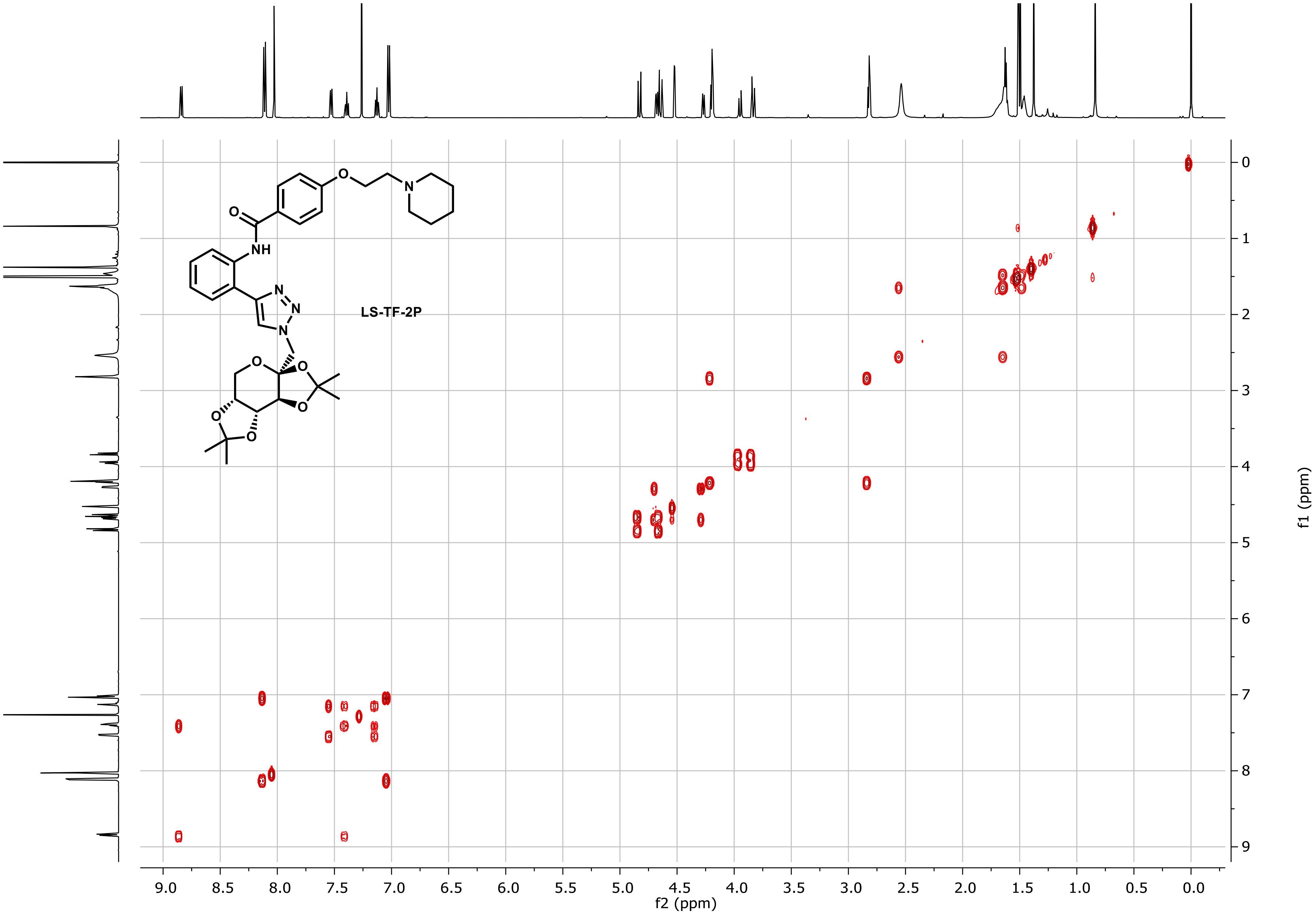

COSY Spectrum for **LS-TF-2P** ($CDCl_3$).

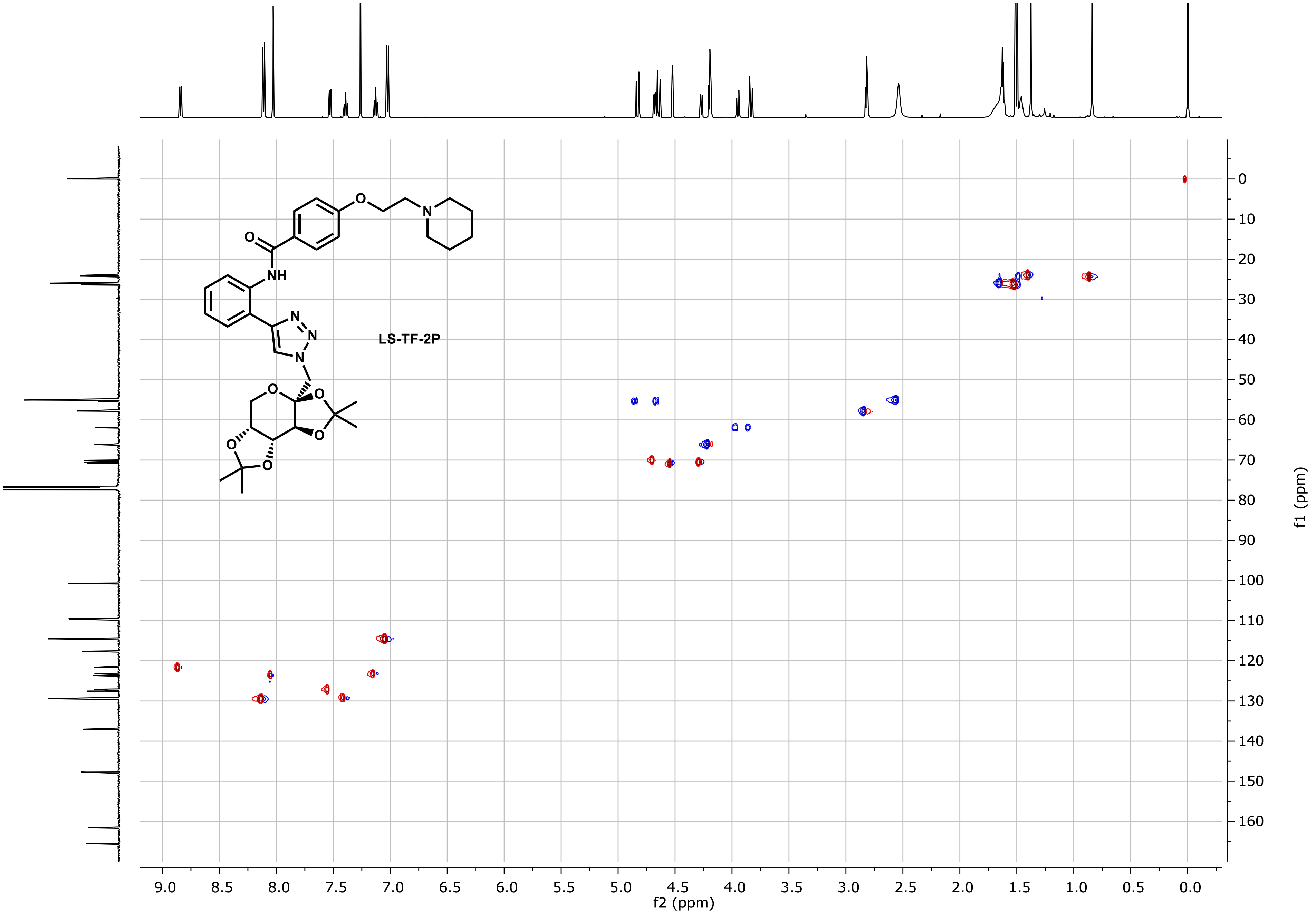

HSQC Spectrum for **LS-TF-2P** ($CDCl_3$).

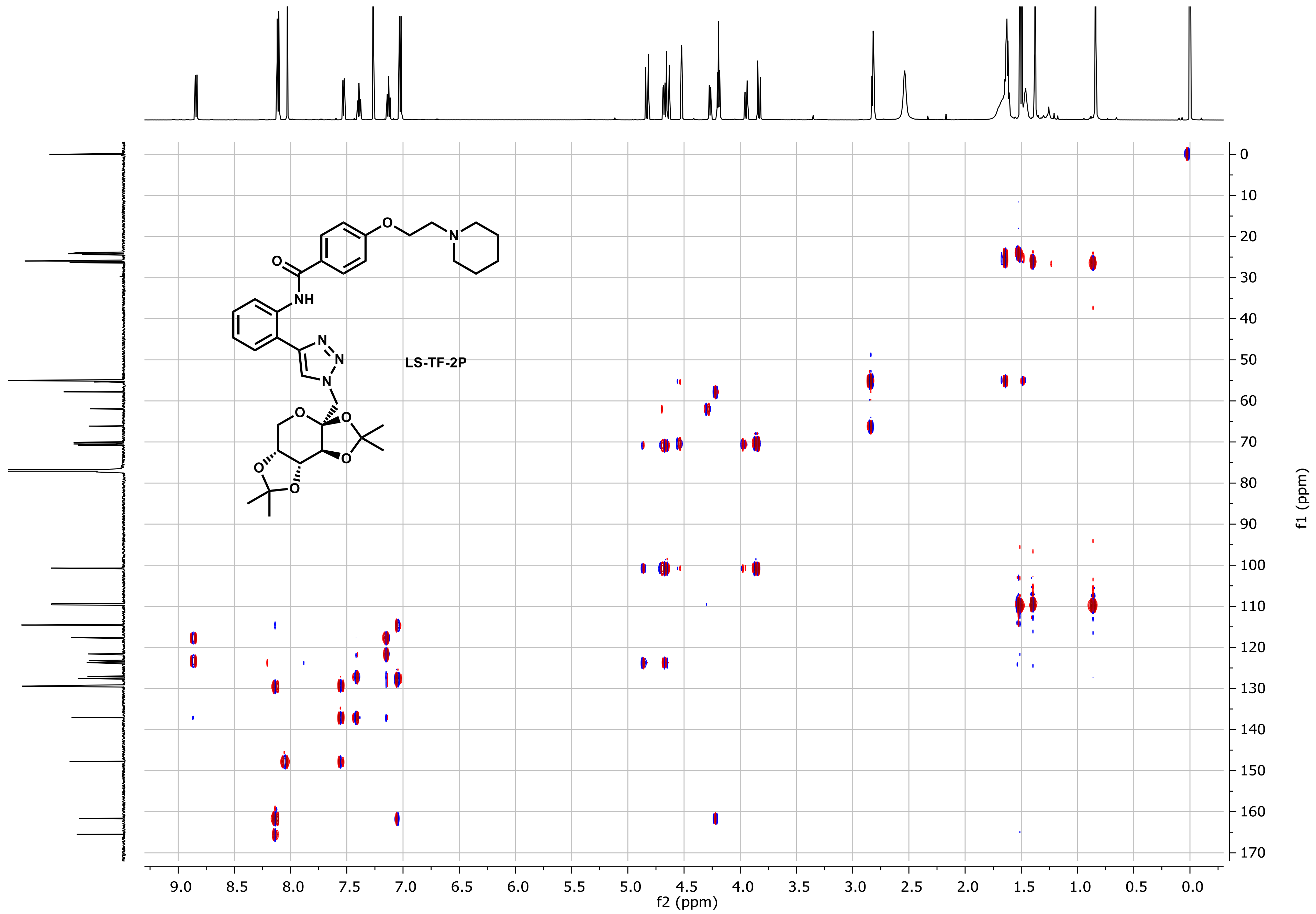

HMBC Spectrum for **LS-TF-2P** ($CDCl_3$).

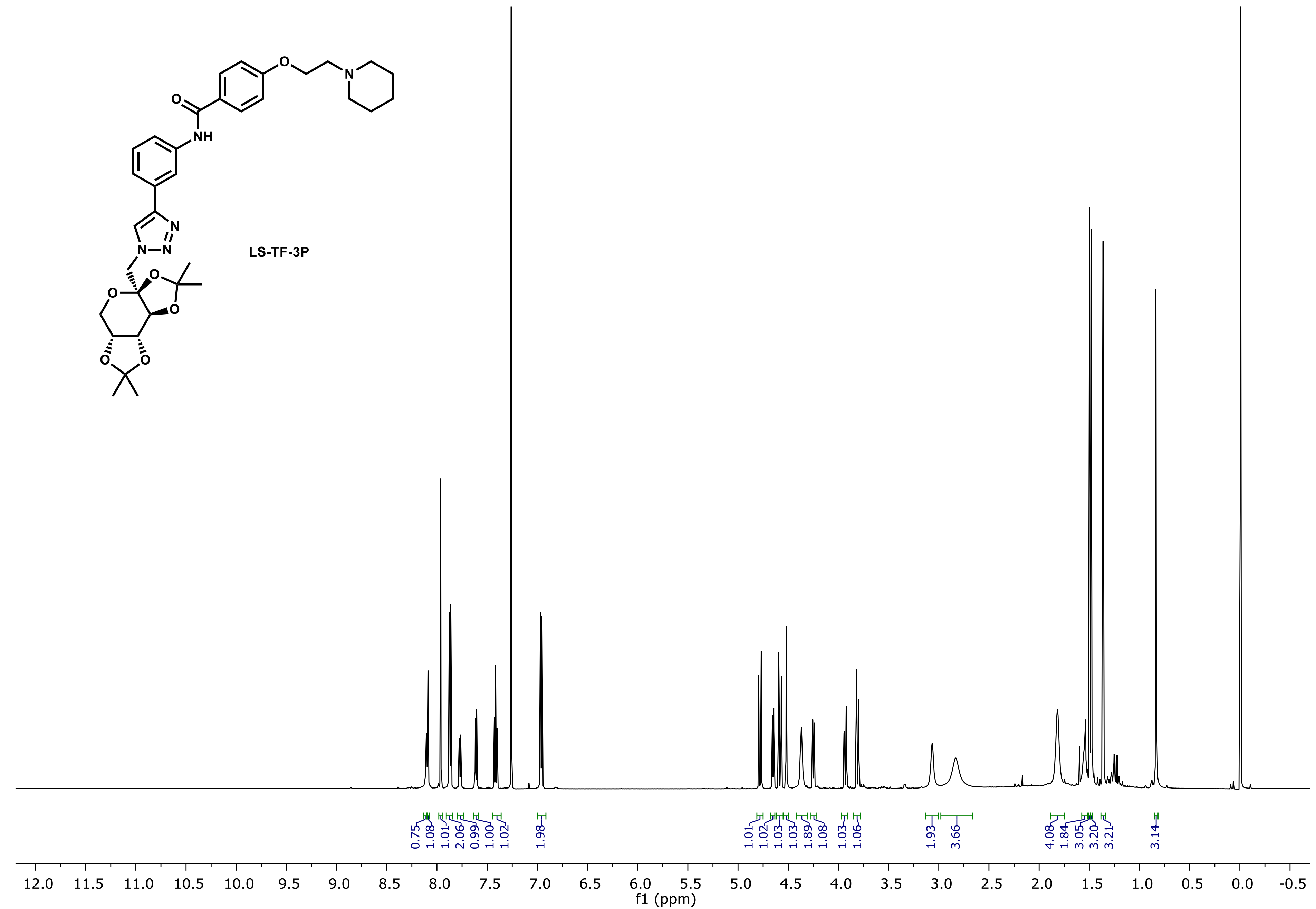

^{1}H NMR Spectrum for **LS-TF-3P** ($CDCl_3$, 600 MHz).

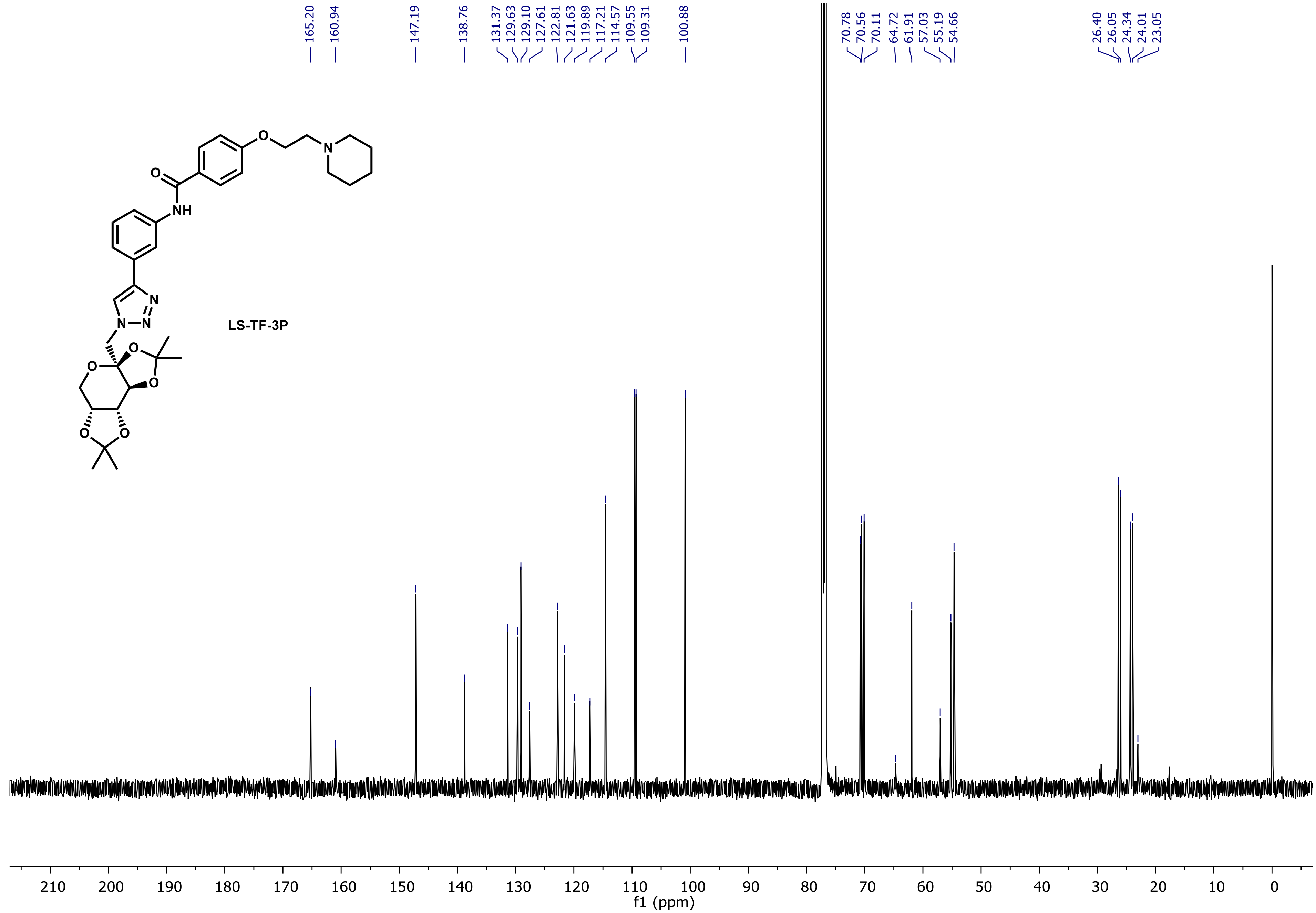

^{13}C NMR Spectrum for **LS-TF-3P** ($CDCl_3$, 151 MHz).

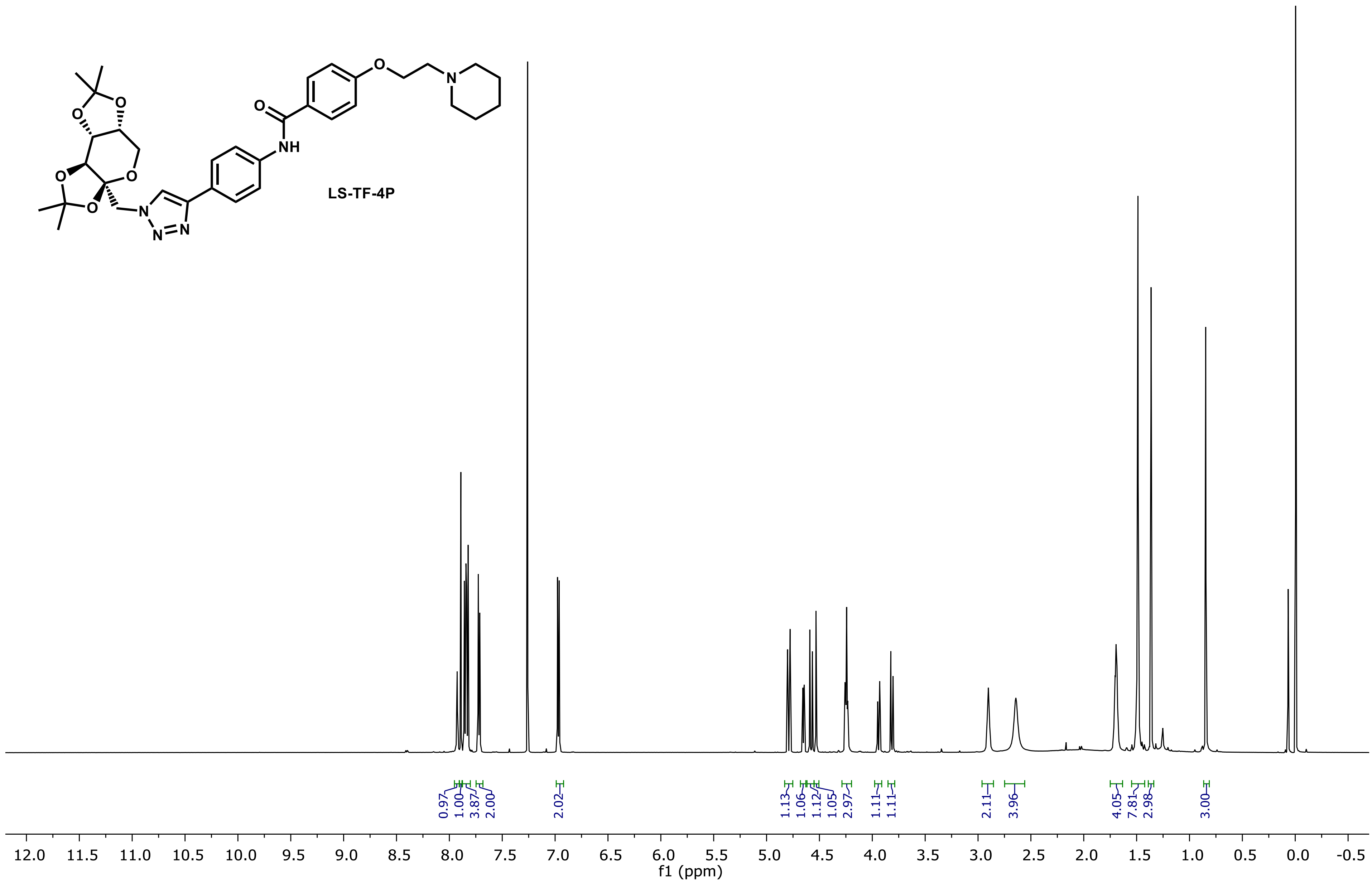

^{1}H NMR Spectrum for **LS-TF-4P** ($CDCl_3$, 600 MHz).

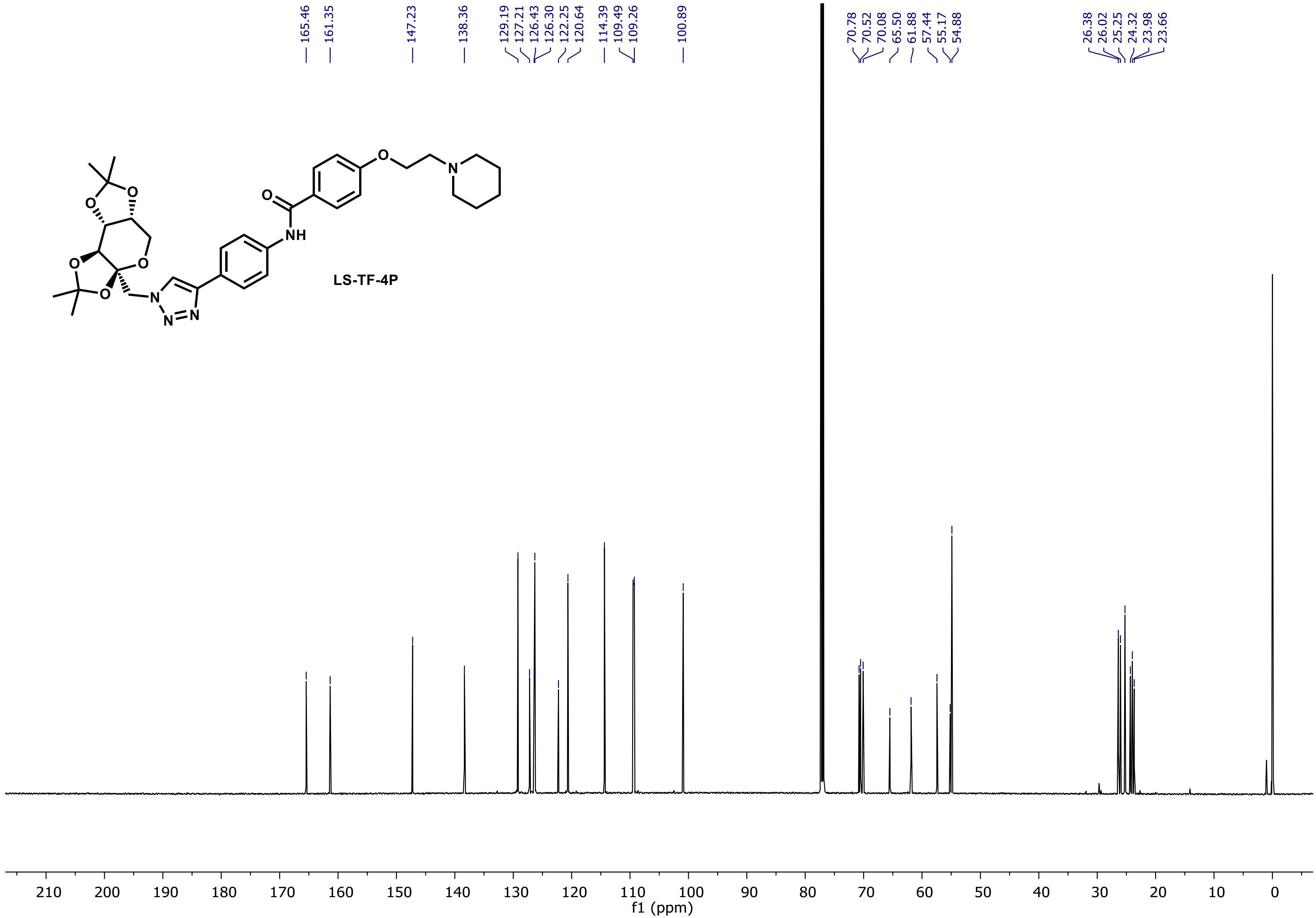

^{13}C NMR Spectrum for **LS-TF-4P** ($CDCl_3$, 151 MHz).

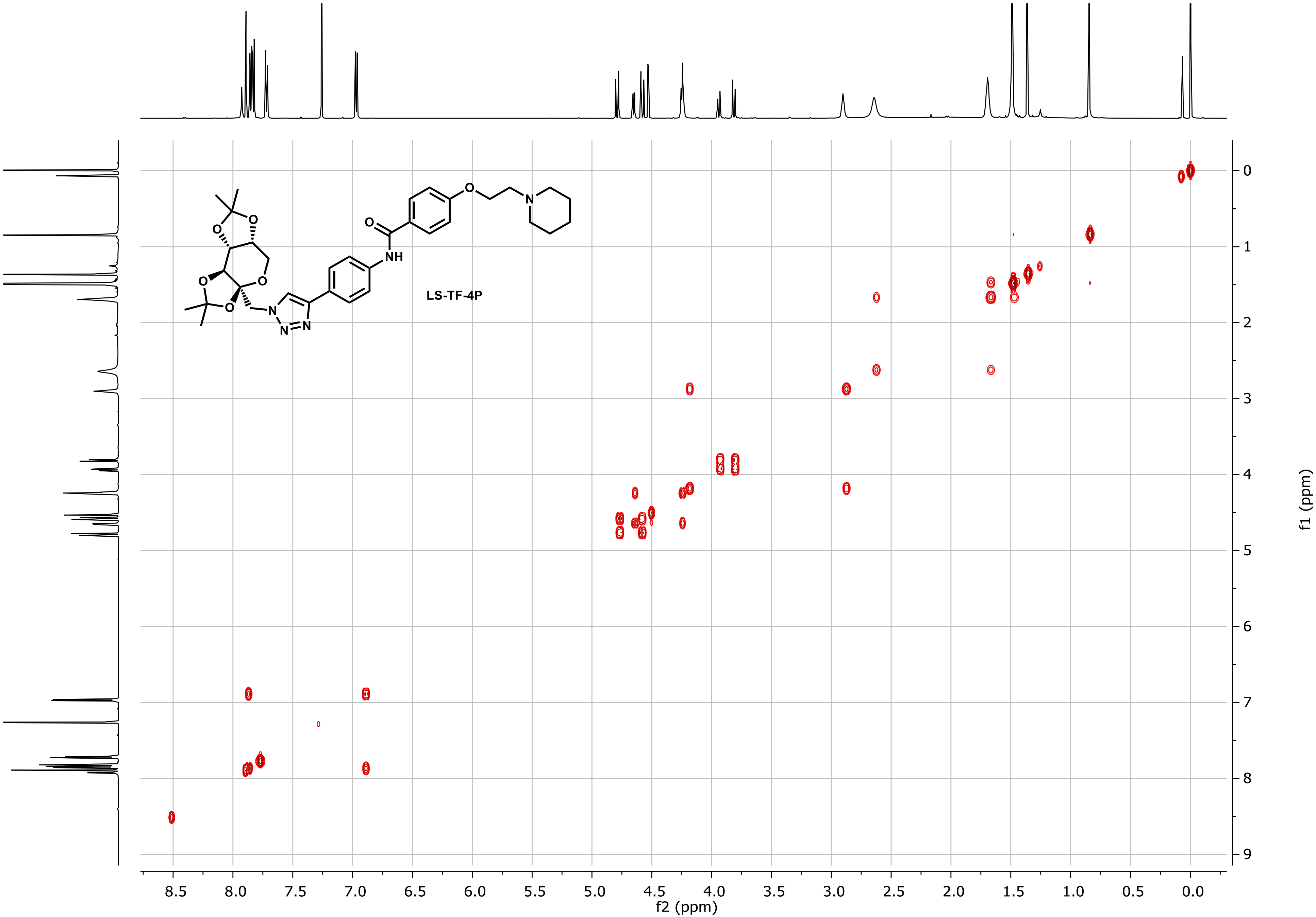

COSY Spectrum for **LS-TF-4P** ($CDCl_3$).

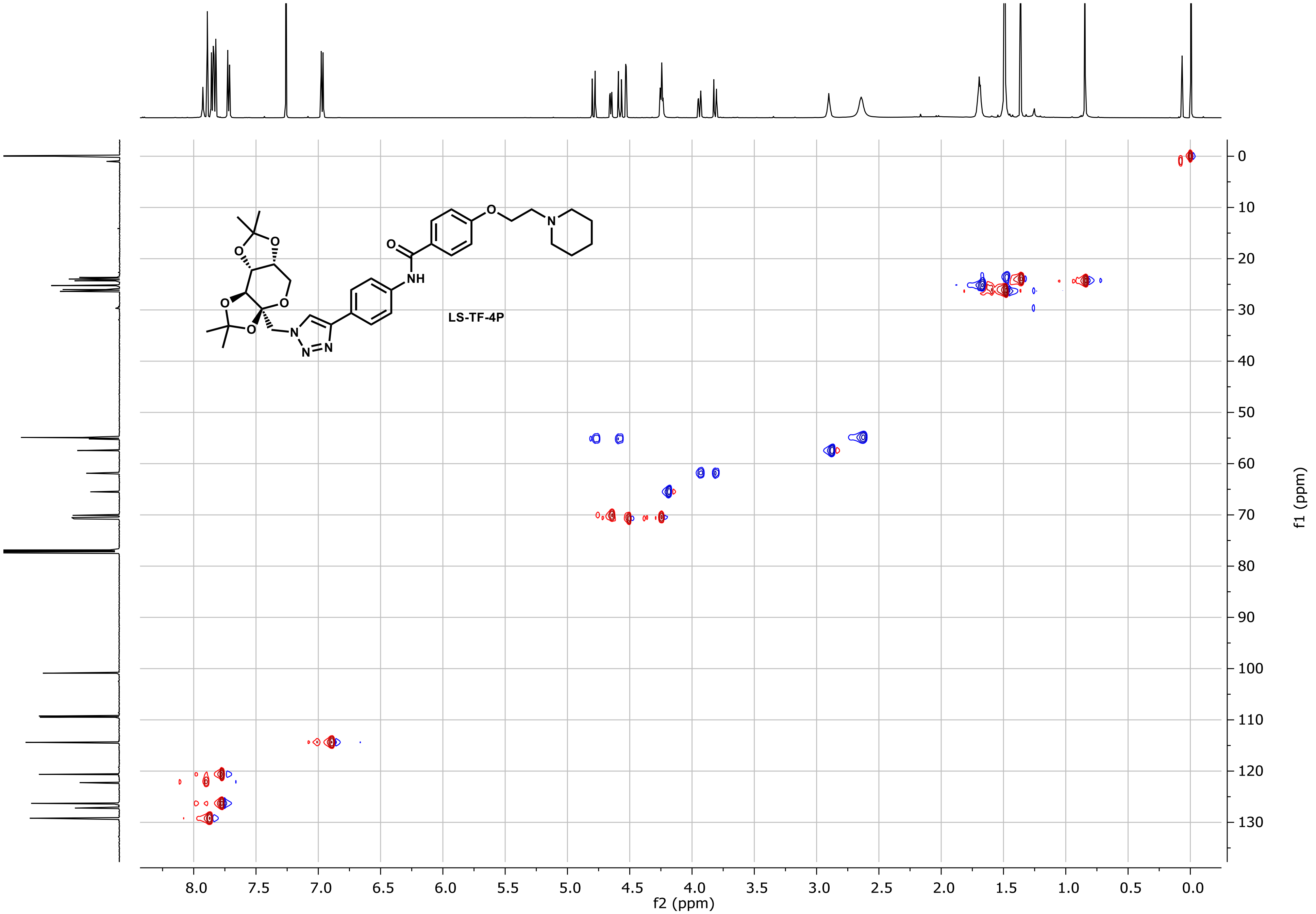

HSQC Spectrum for **LS-TF-4P** ($CDCl_3$).

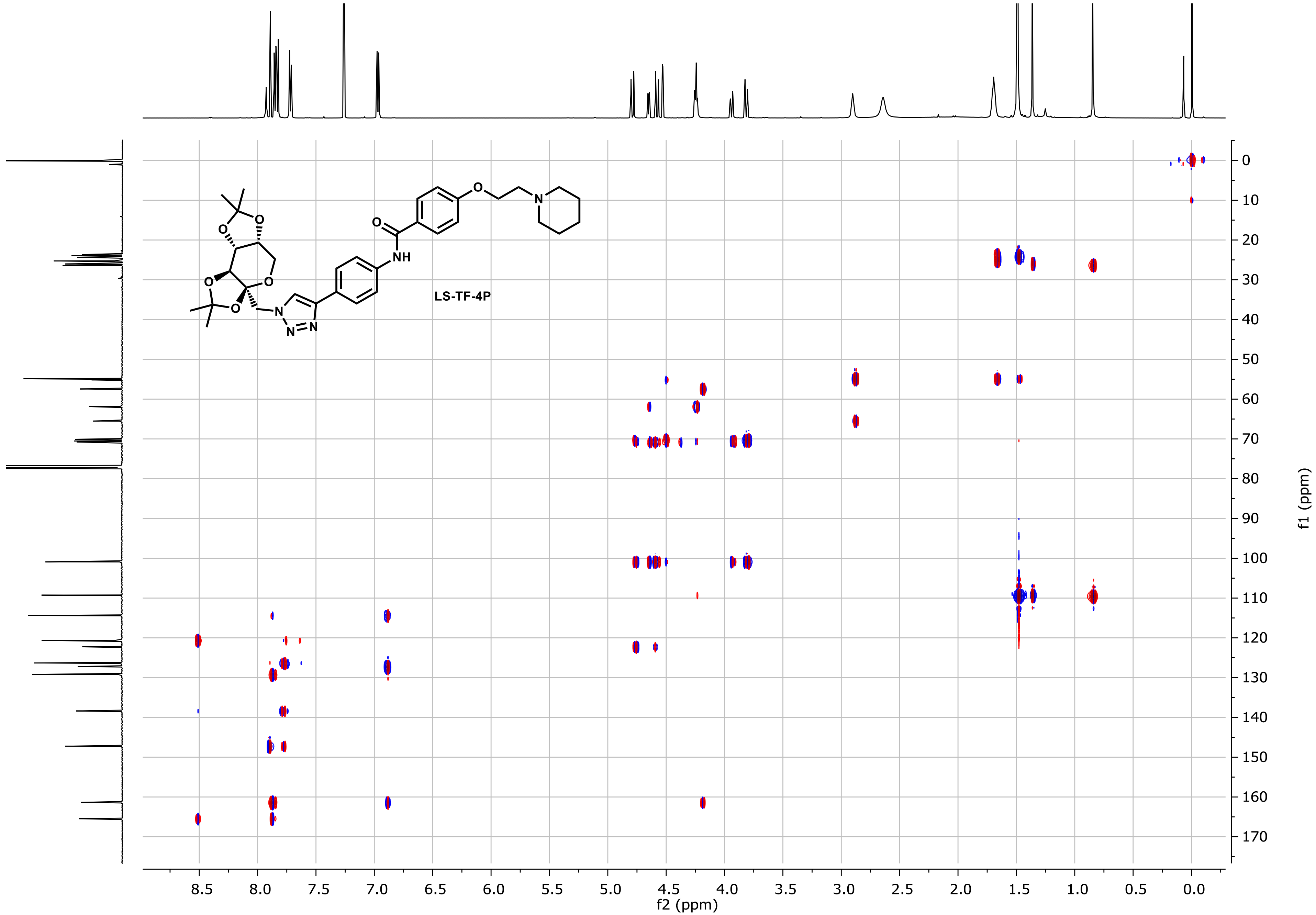

HMBC Spectrum for **LS-TF-4P** ($CDCl_3$).

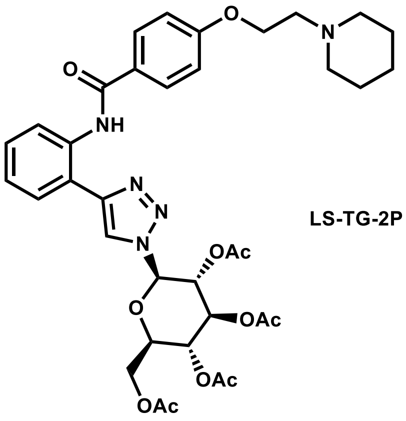

LS-TG-2P

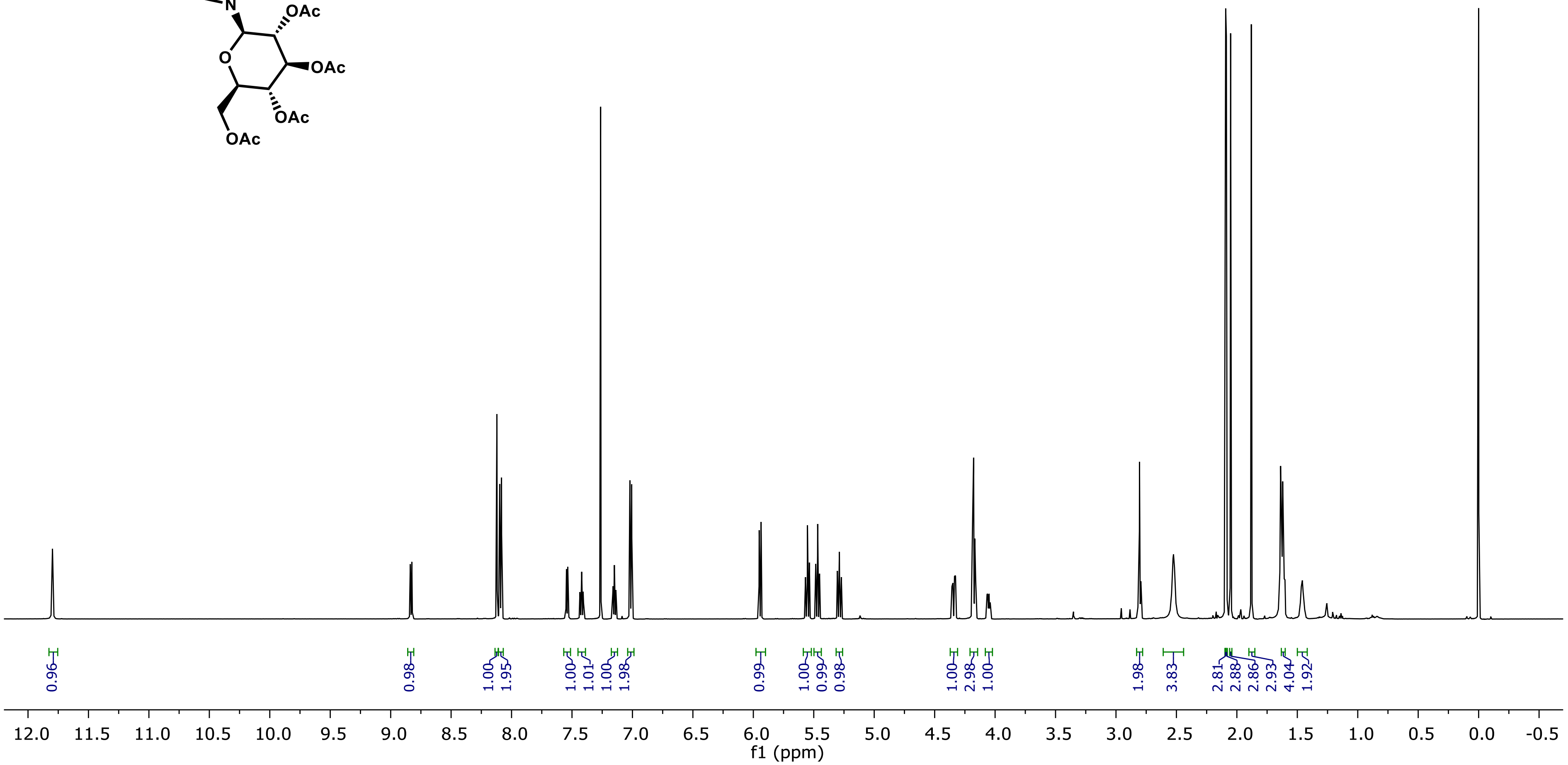

^{1}H NMR Spectrum for **LS-TG-2P** ($CDCl_3$, 600 MHz).

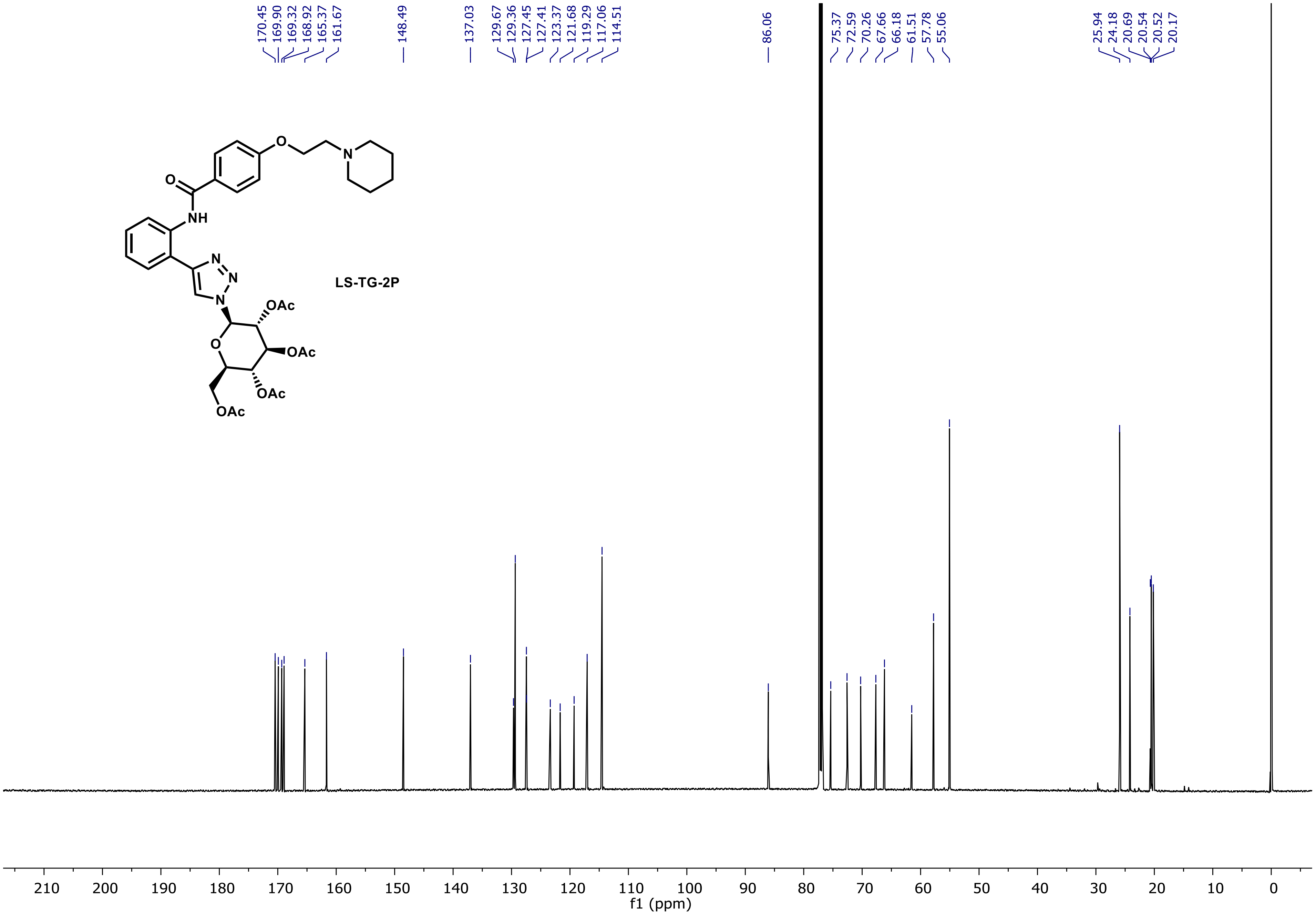

^{13}C NMR Spectrum for **LS-TG-2P** ($CDCl_3$, 151 MHz).

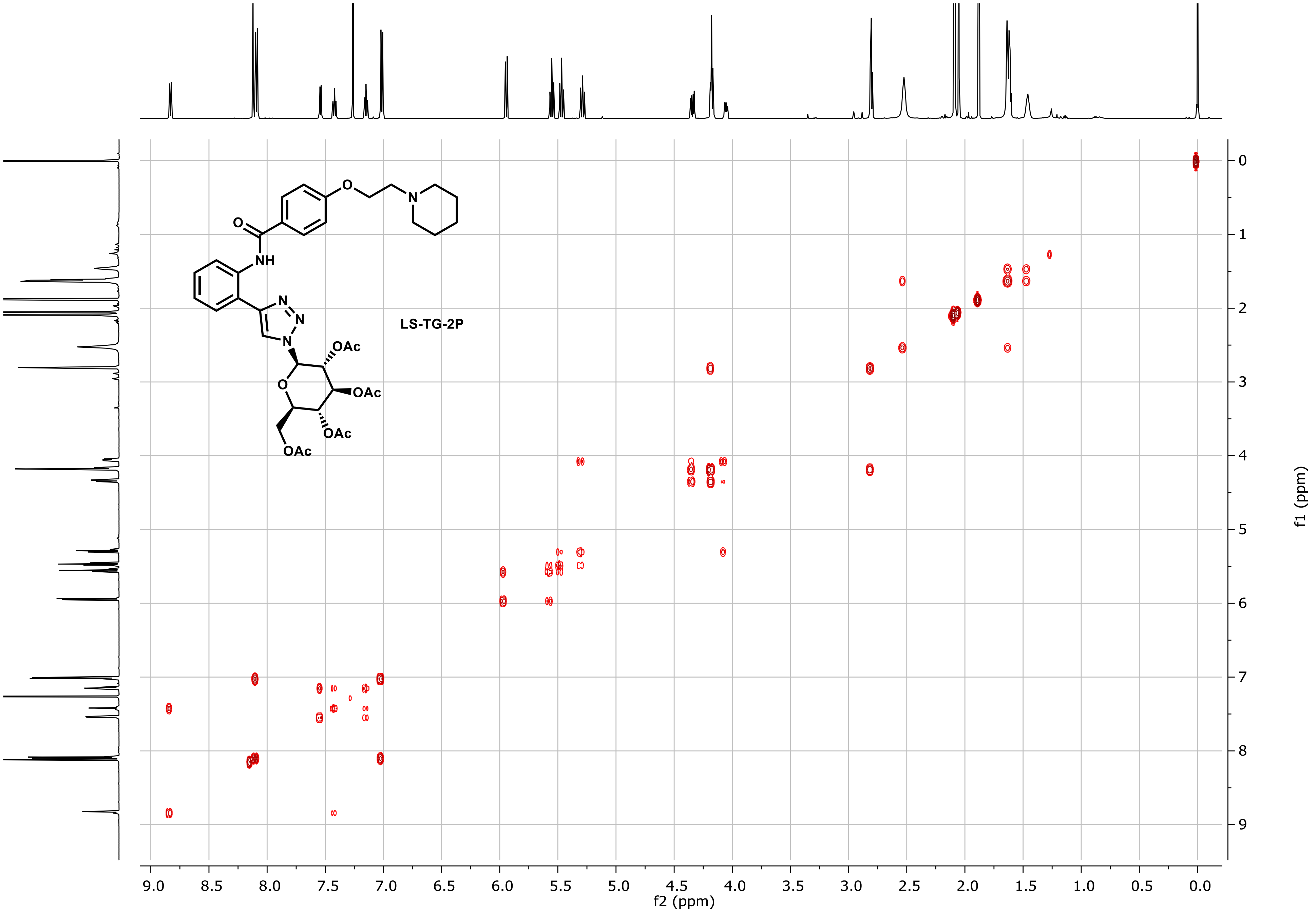

COSY Spectrum for **LS-TG-2P** ($CDCl_3$).

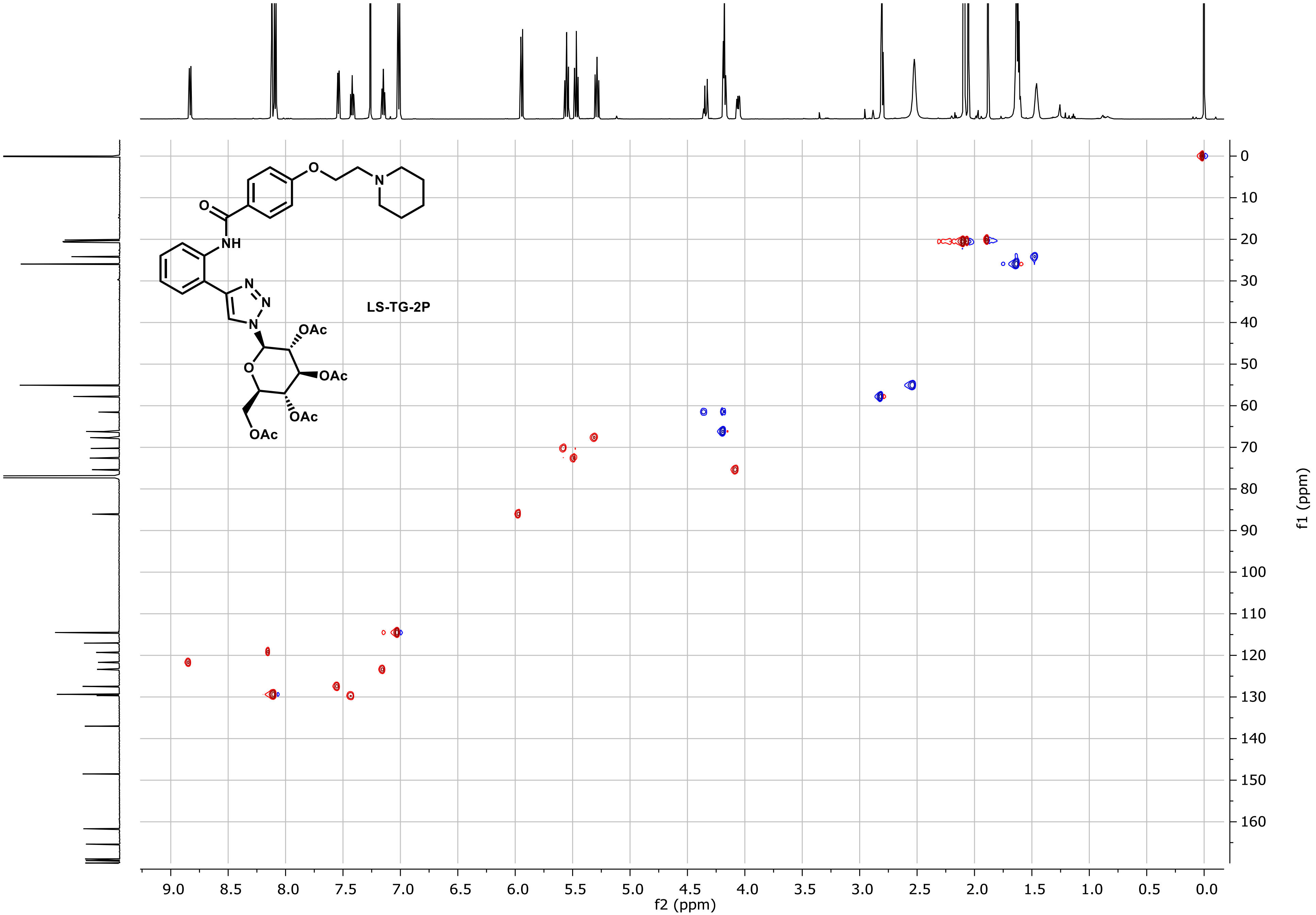

HSQC Spectrum for **LS-TG-2P** ($CDCl_3$).

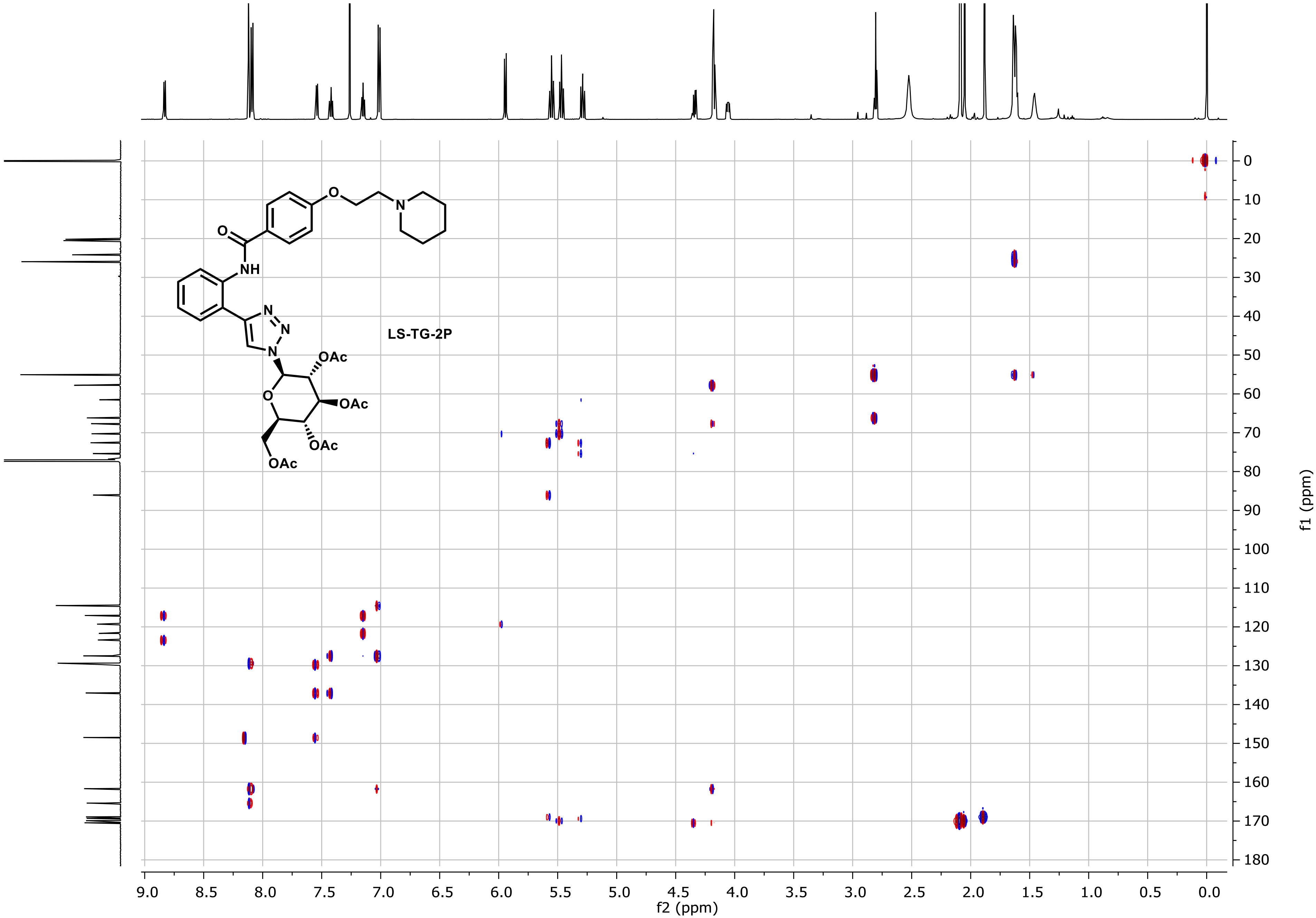

HMBC Spectrum for **LS-TG-2P** ($CDCl_3$).

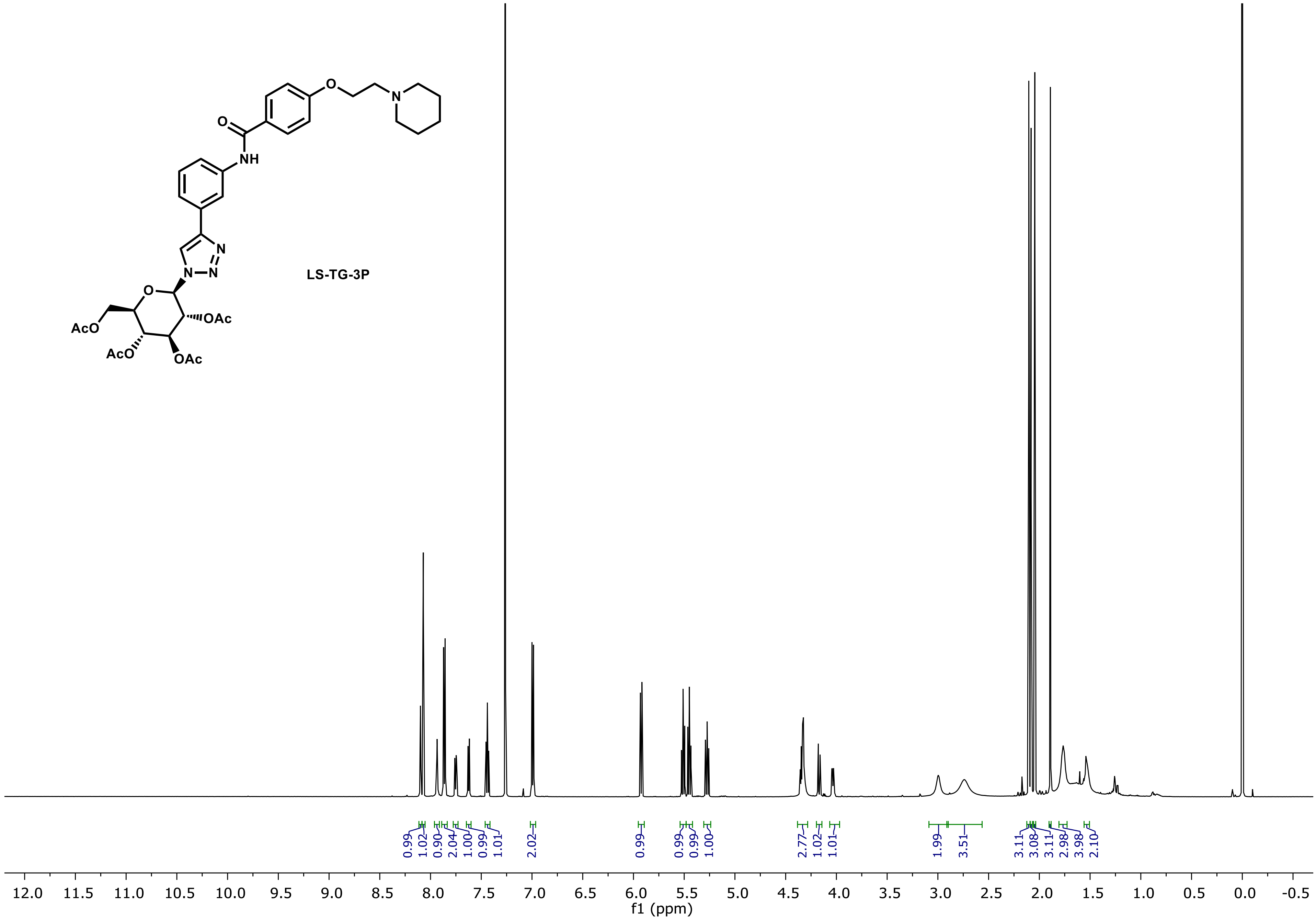

^{1}H NMR Spectrum for **LS-TG-3P** ($CDCl_3$, 600 MHz).

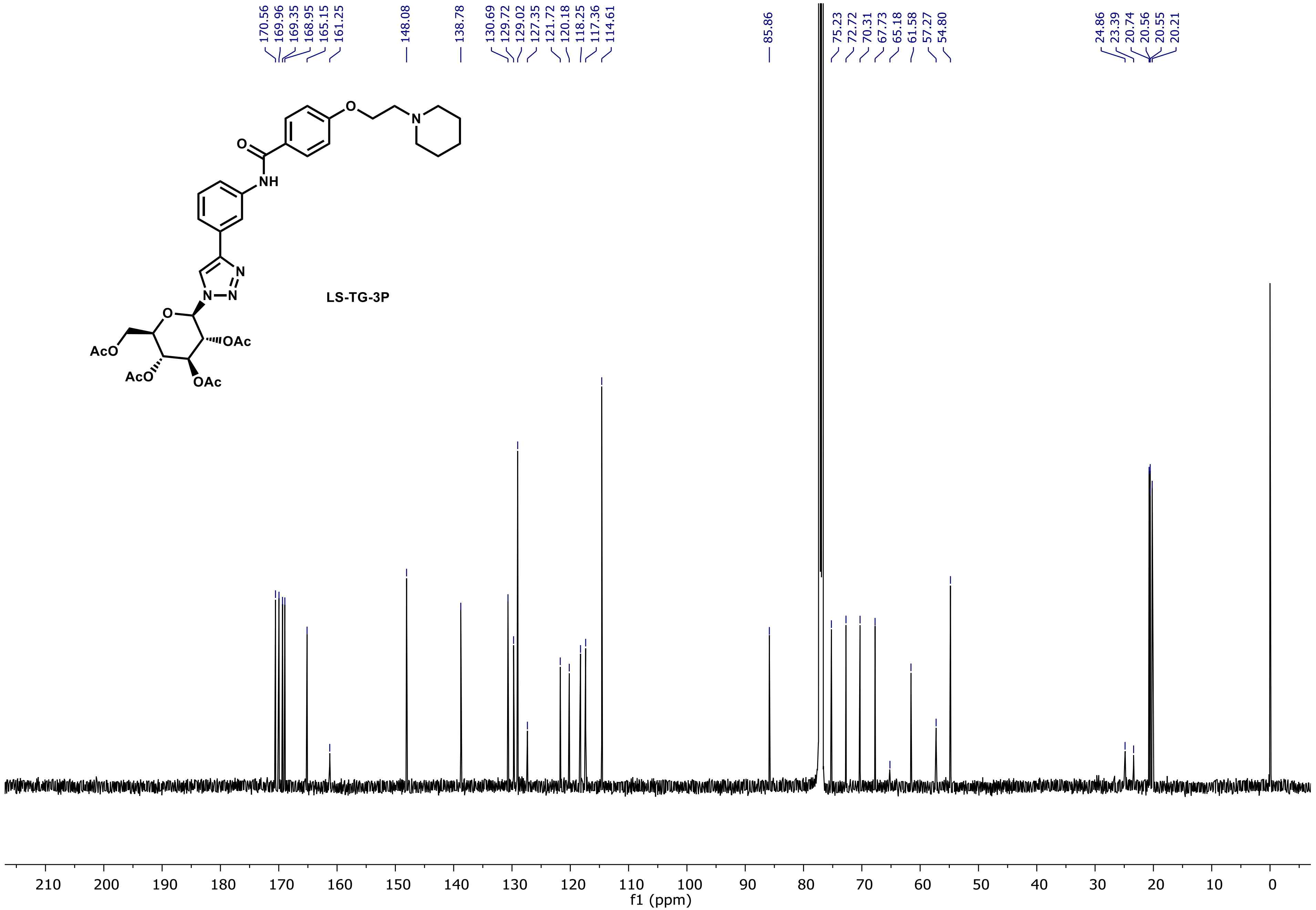

^{13}C NMR Spectrum for **LS-TG-3P** ($CDCl_3$, 151 MHz).

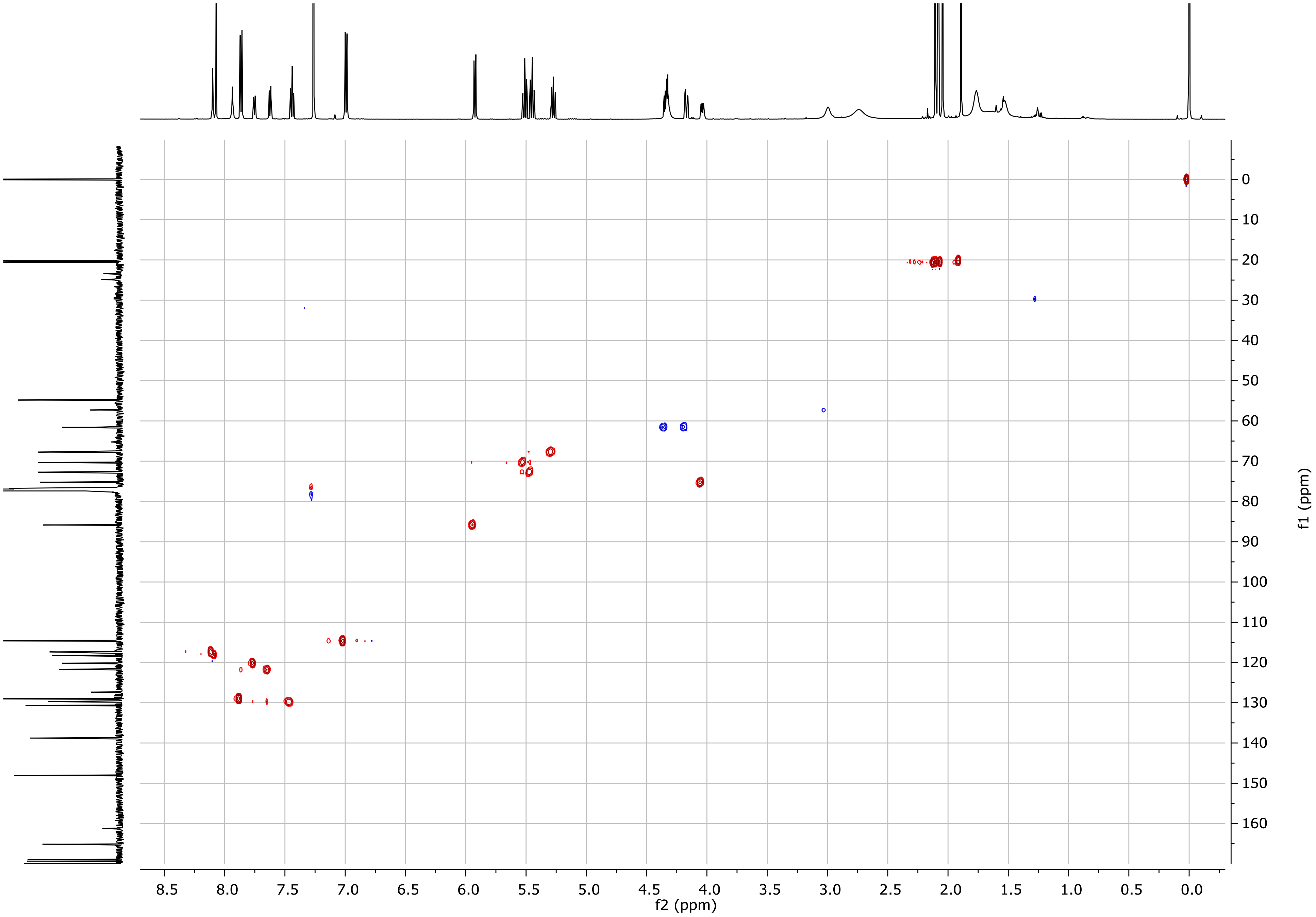

HSQC Spectrum for **LS-TG-3P** ($CDCl_3$).

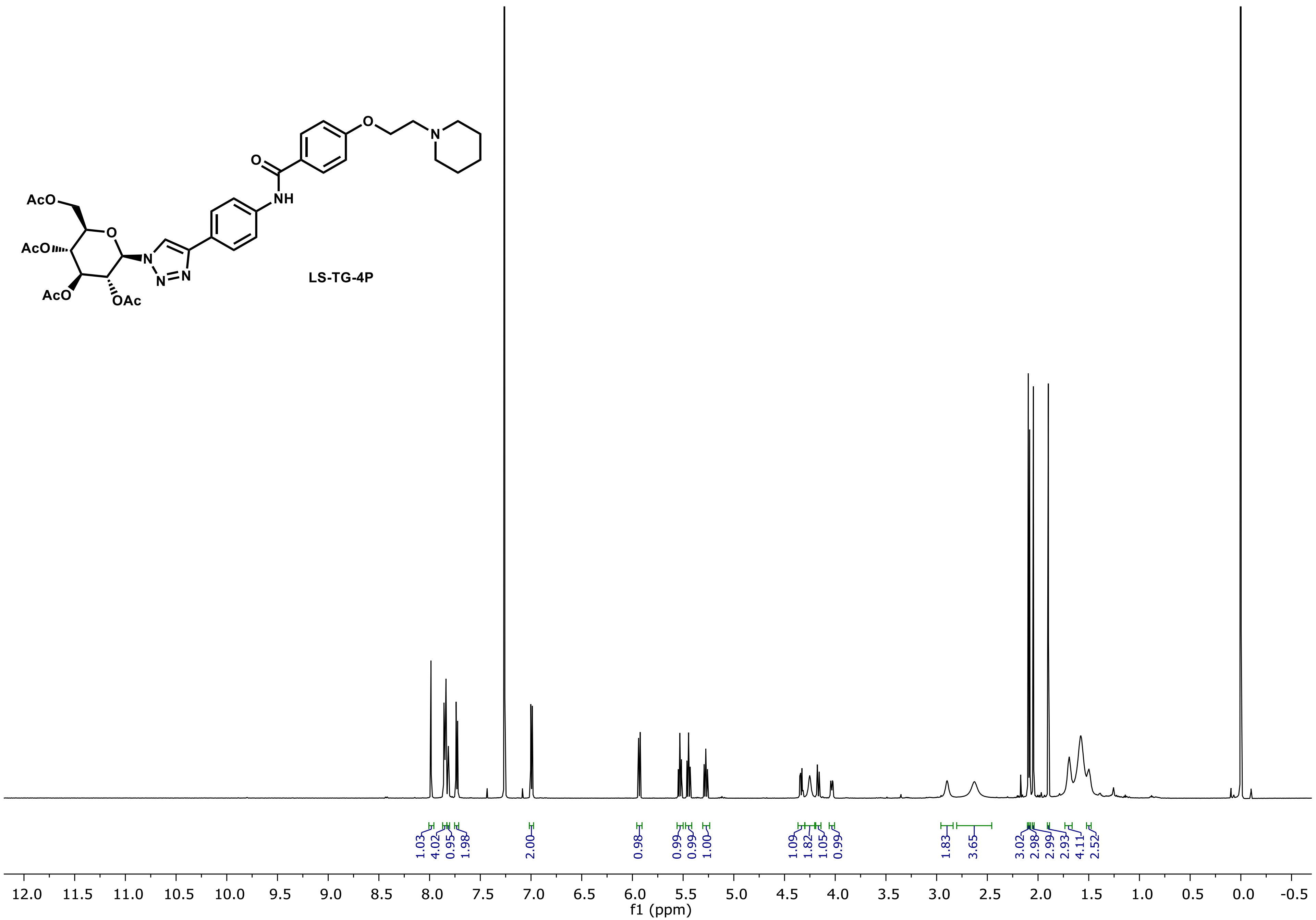

^{1}H NMR Spectrum for **LS-TG-4P** ($CDCl_3$, 600 MHz).

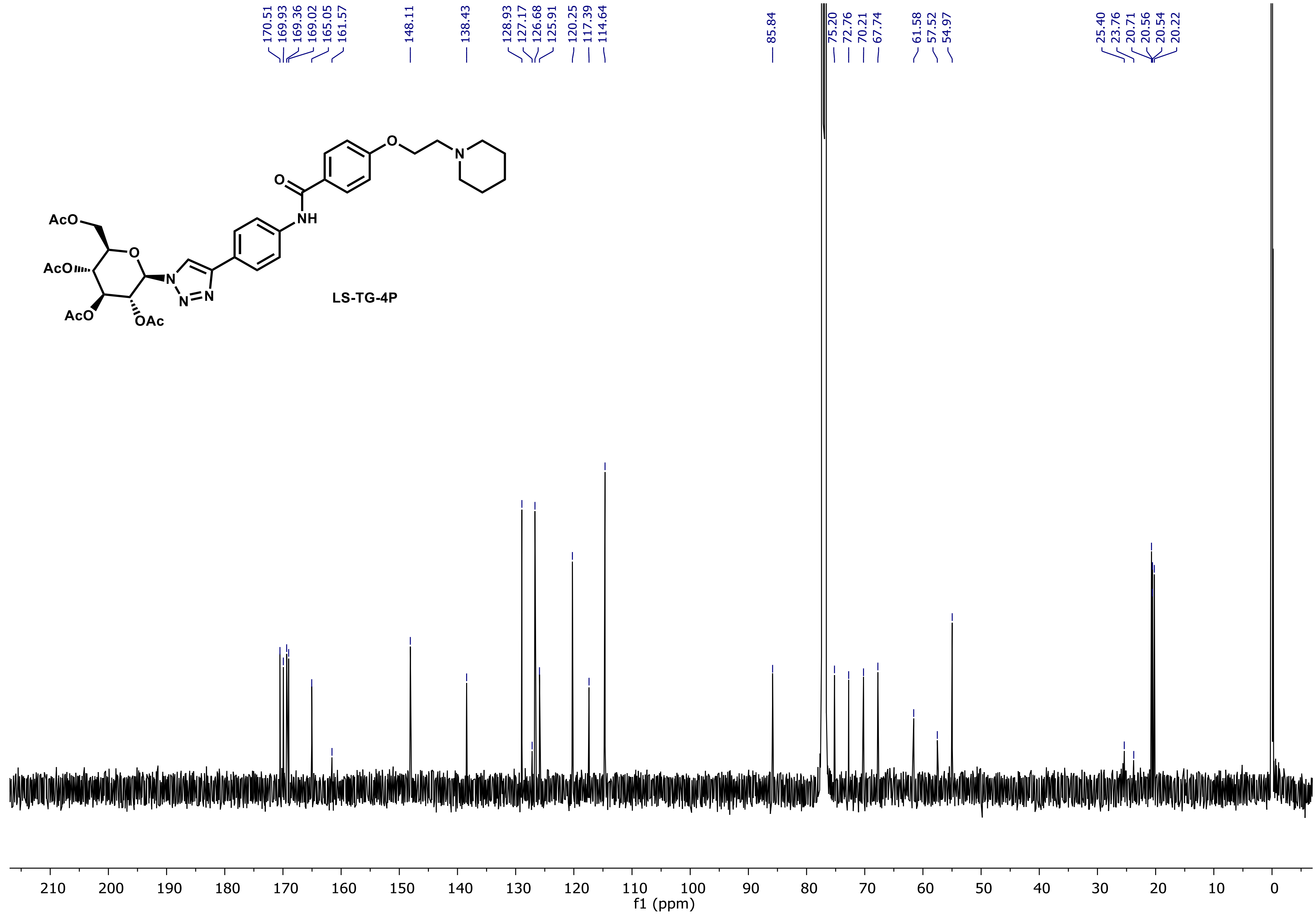

^{13}C NMR Spectrum for **LS-TG-4P** ($CDCl_3$, 151 MHz).

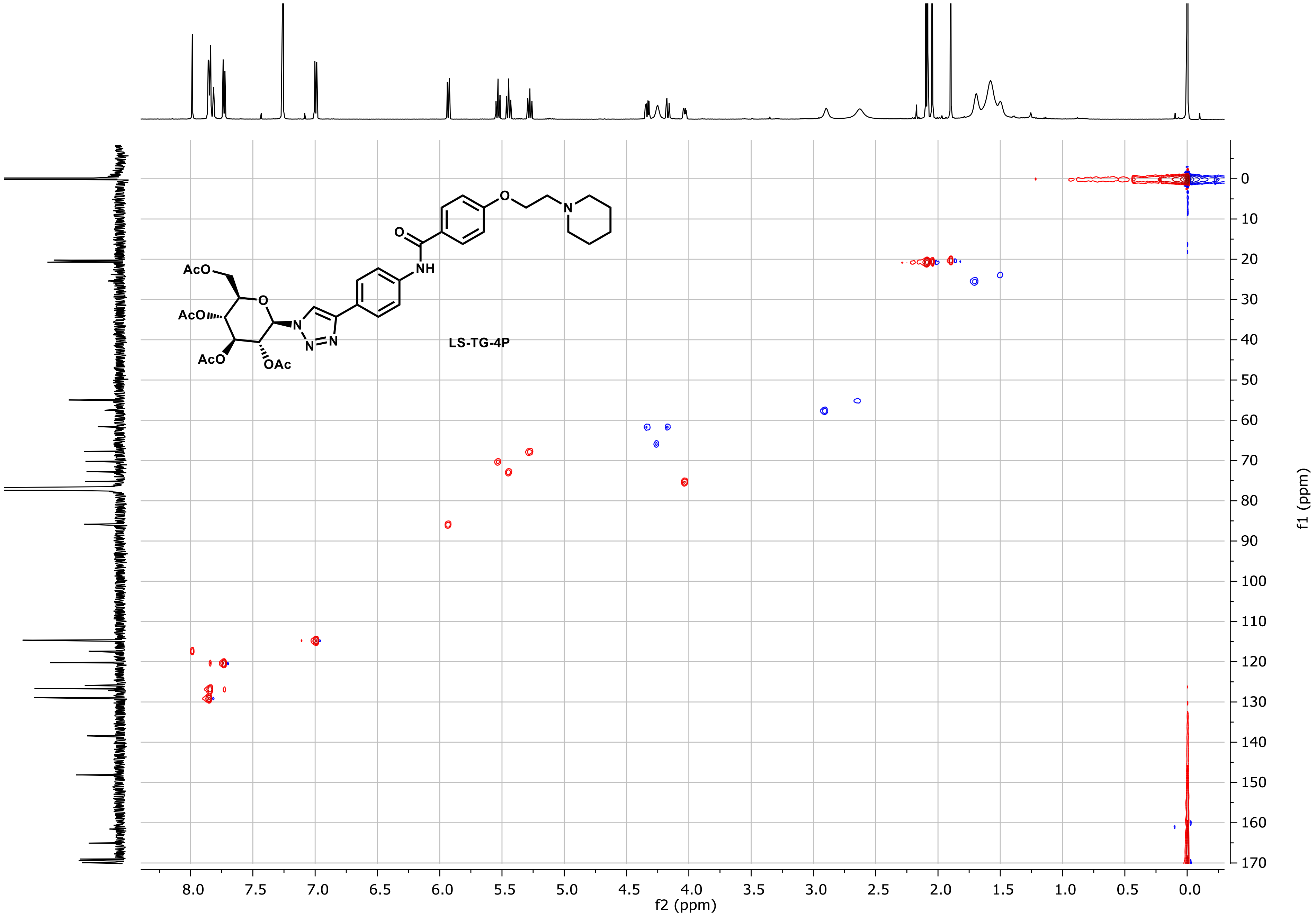

HSQC Spectrum for **LS-TG-4P** ($CDCl_3$).

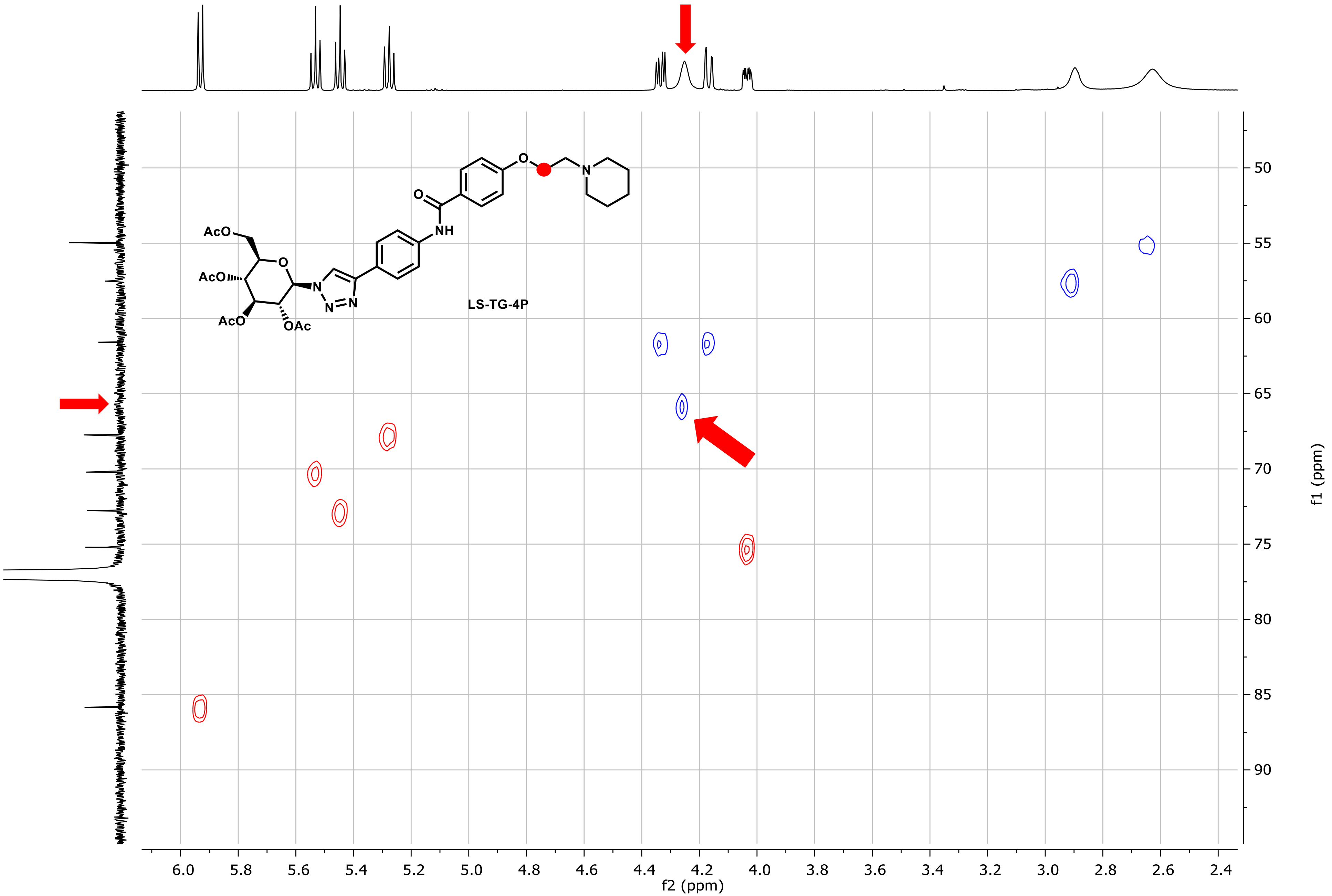

HSQC Spectrum for **LS-TG-4P** ($CDCl_3$) - zoomed-in view of correlation to missing 65.8 ppm ^{13}C signal. Carbon in question indicated by red dot on structure.

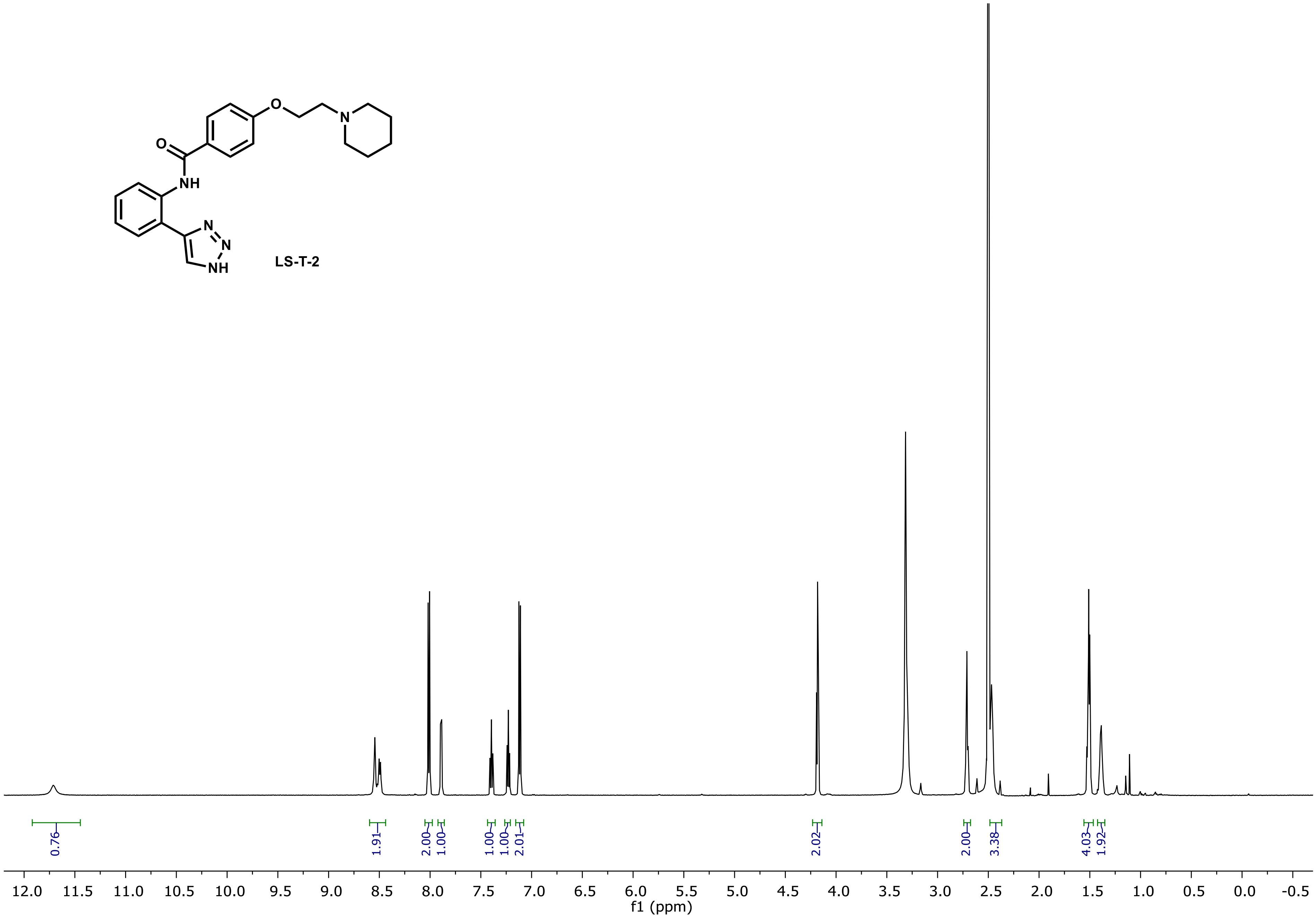

^{1}H NMR Spectrum for **LS-T-2** (DMSO-*d6*, 600 MHz).

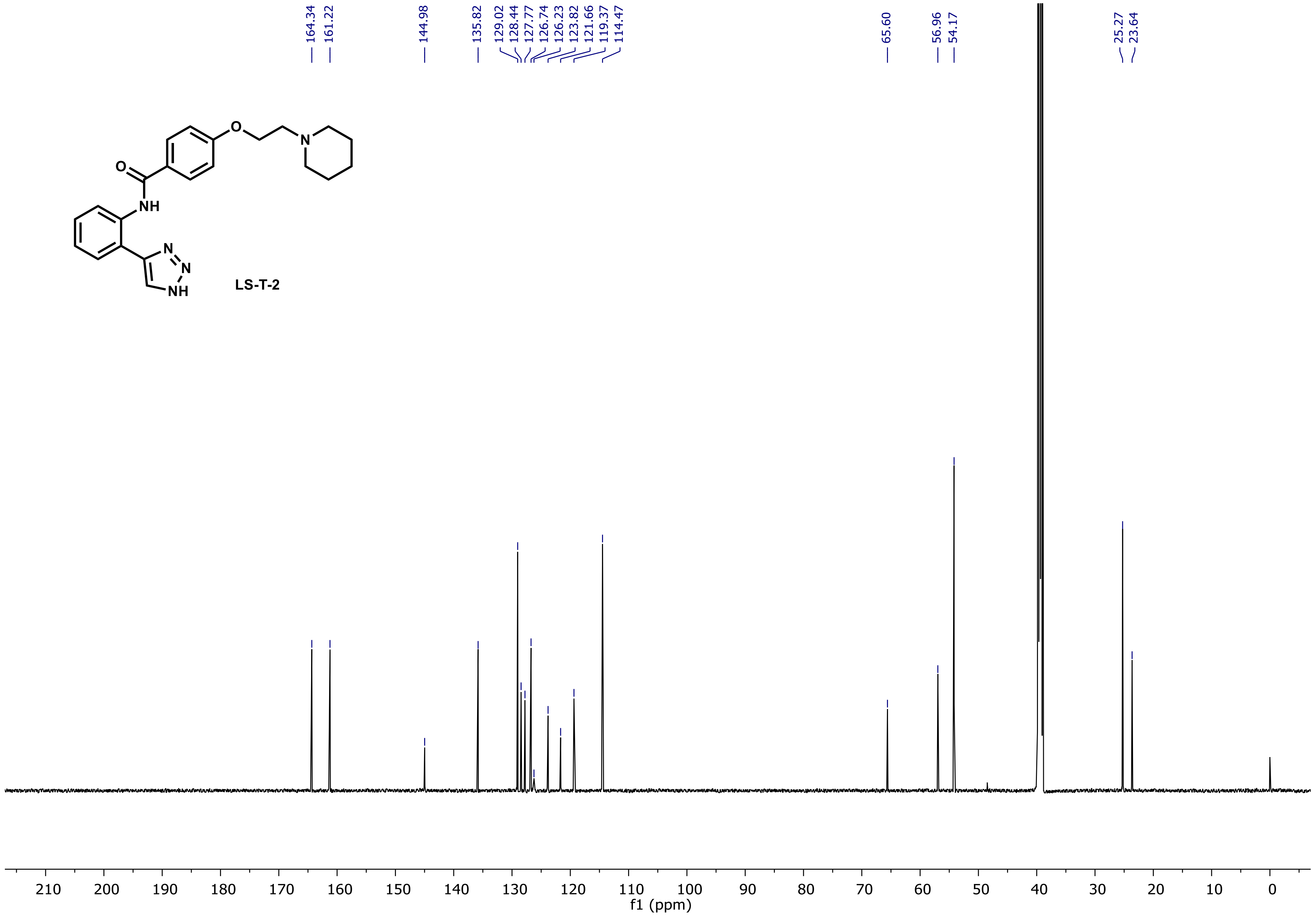

^{13}C NMR Spectrum for **LS-T-2** (DMSO-*d6*, 151 MHz).

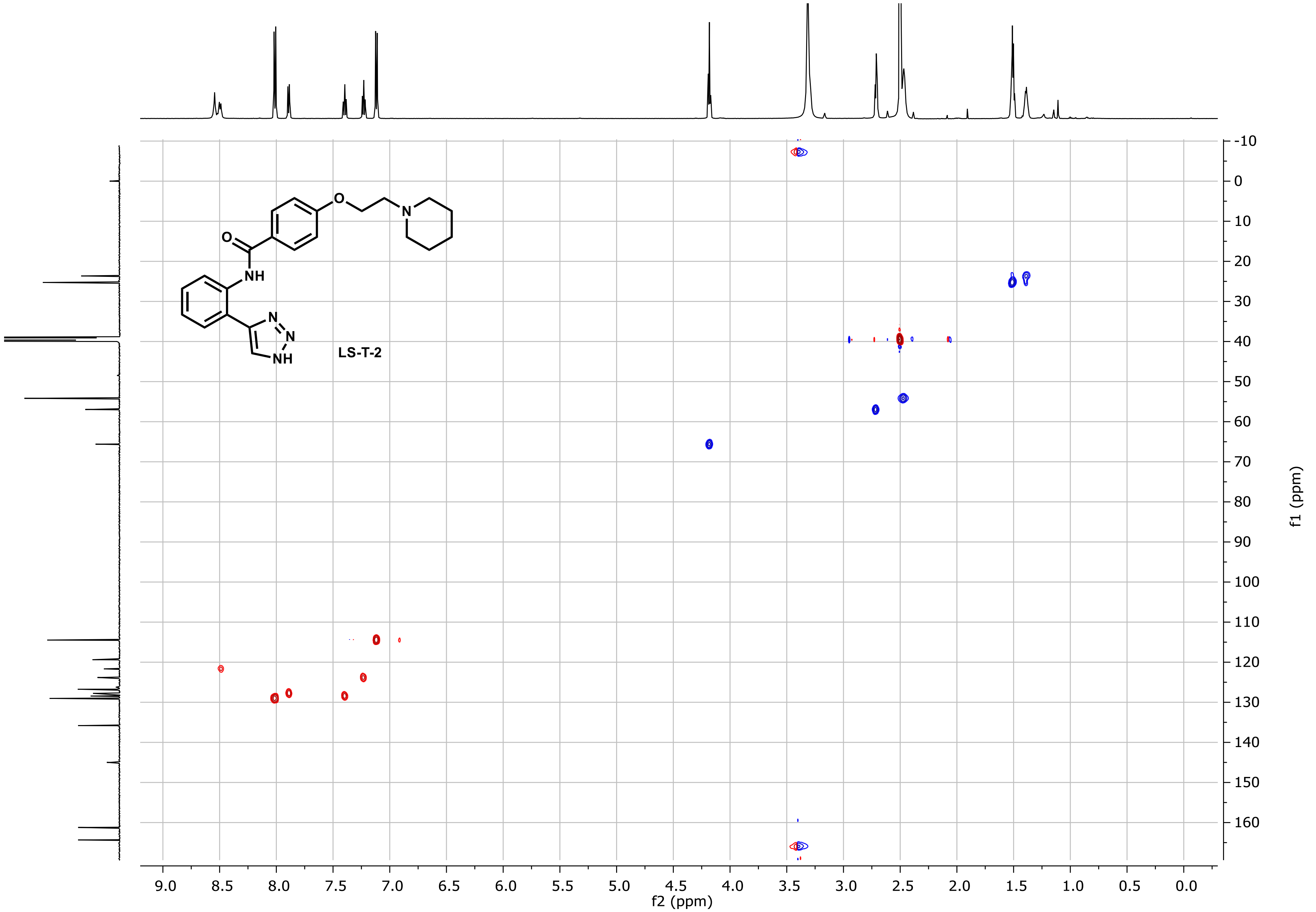

HSQC Spectrum for **LS-T-2** (DMSO-*d6*).

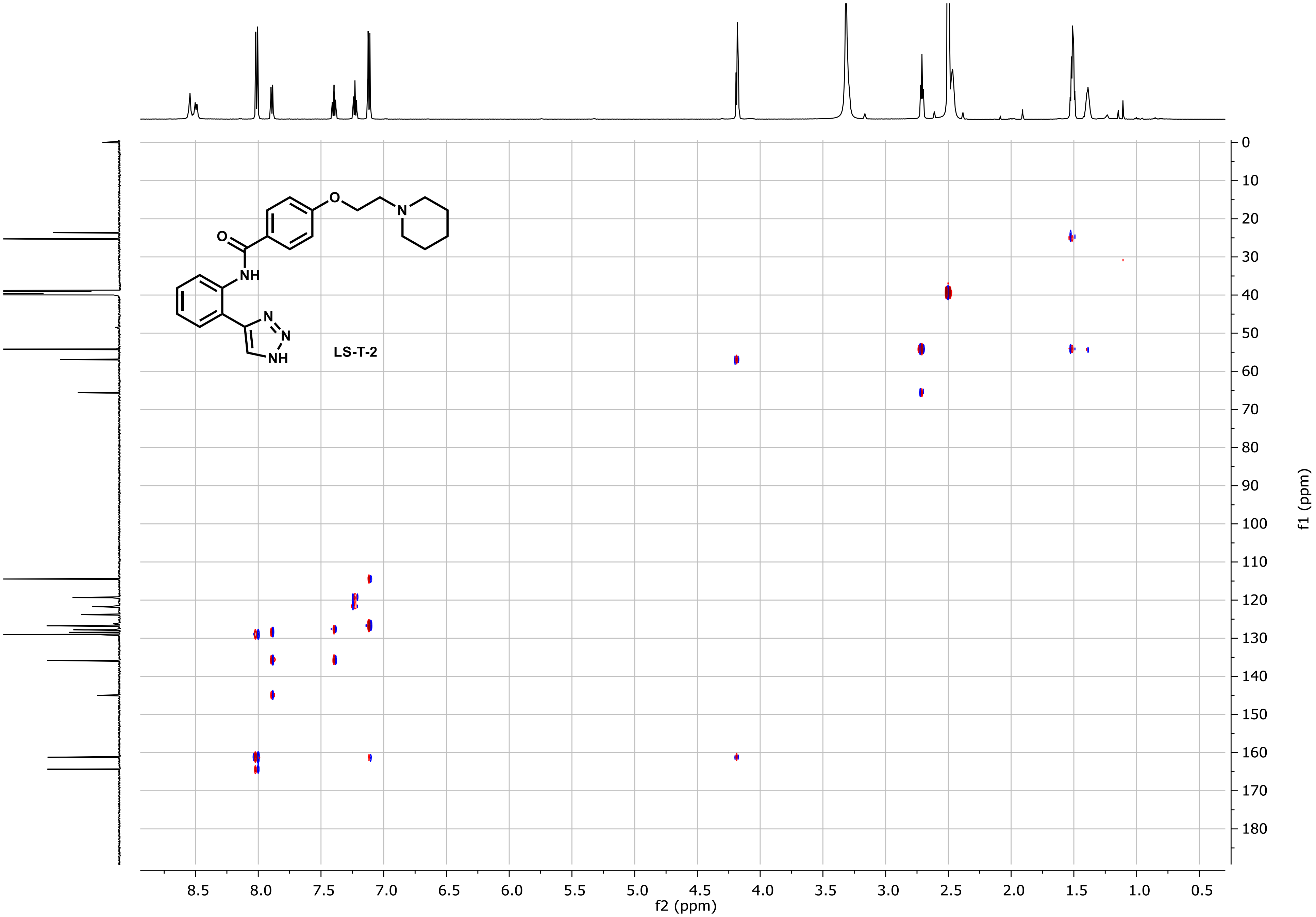

HMBC Spectrum for **LS-T-2** (DMSO-*d6*).

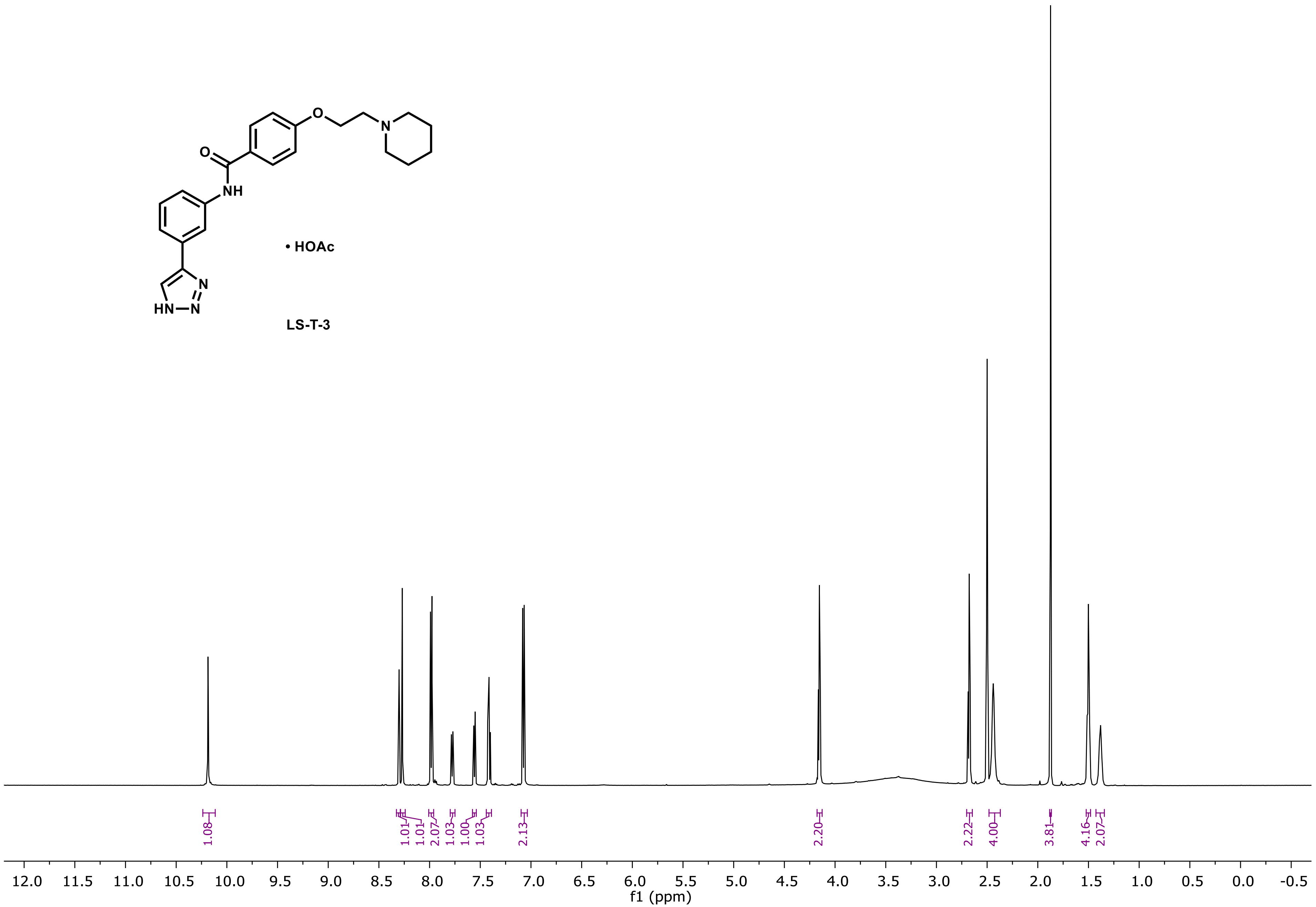

^{1}H NMR Spectrum for **LS-T-3** (DMSO-*d6*, 600 MHz).

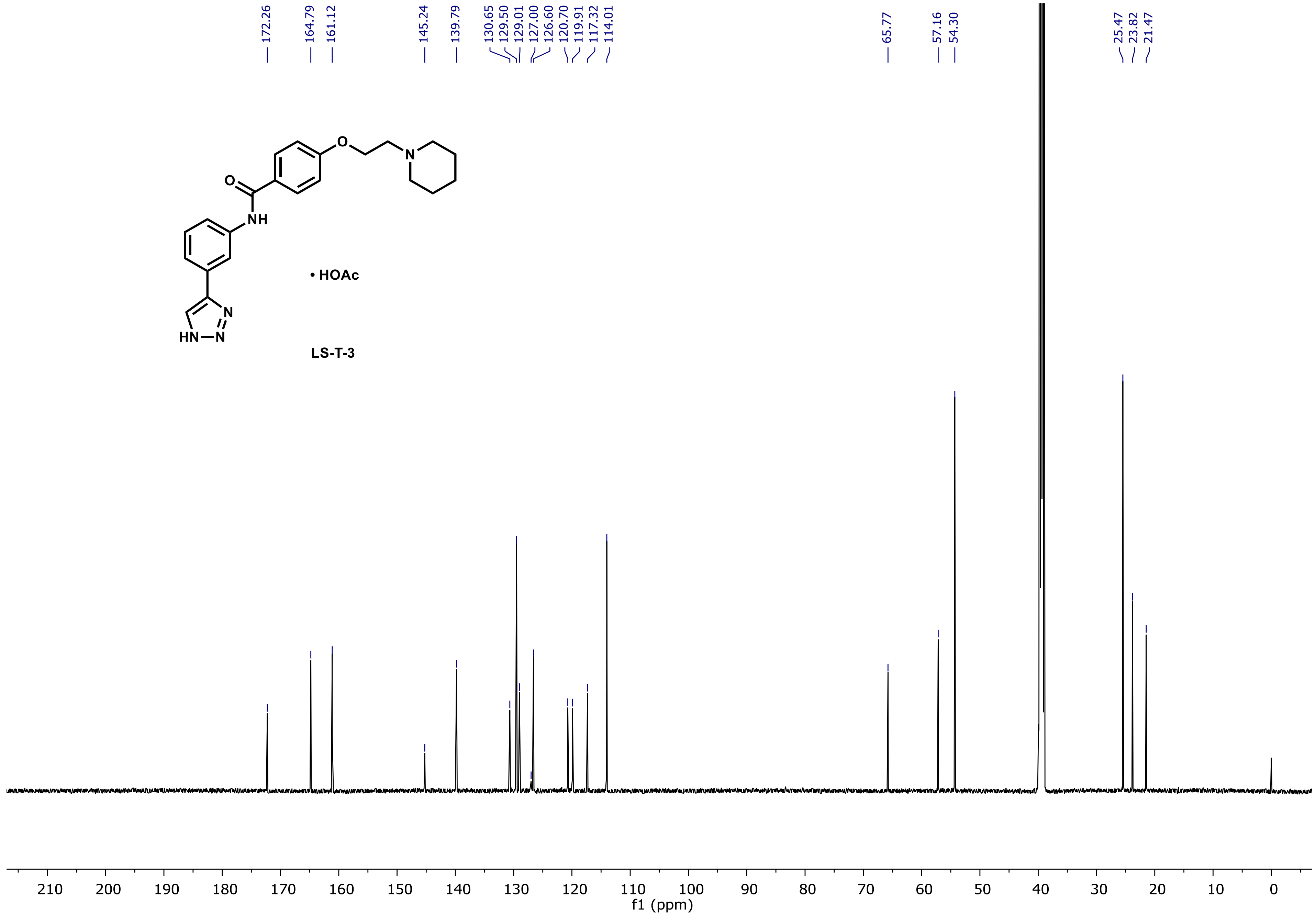

^{13}C NMR Spectrum for **LS-T-3** (DMSO-*d6*, 151 MHz).

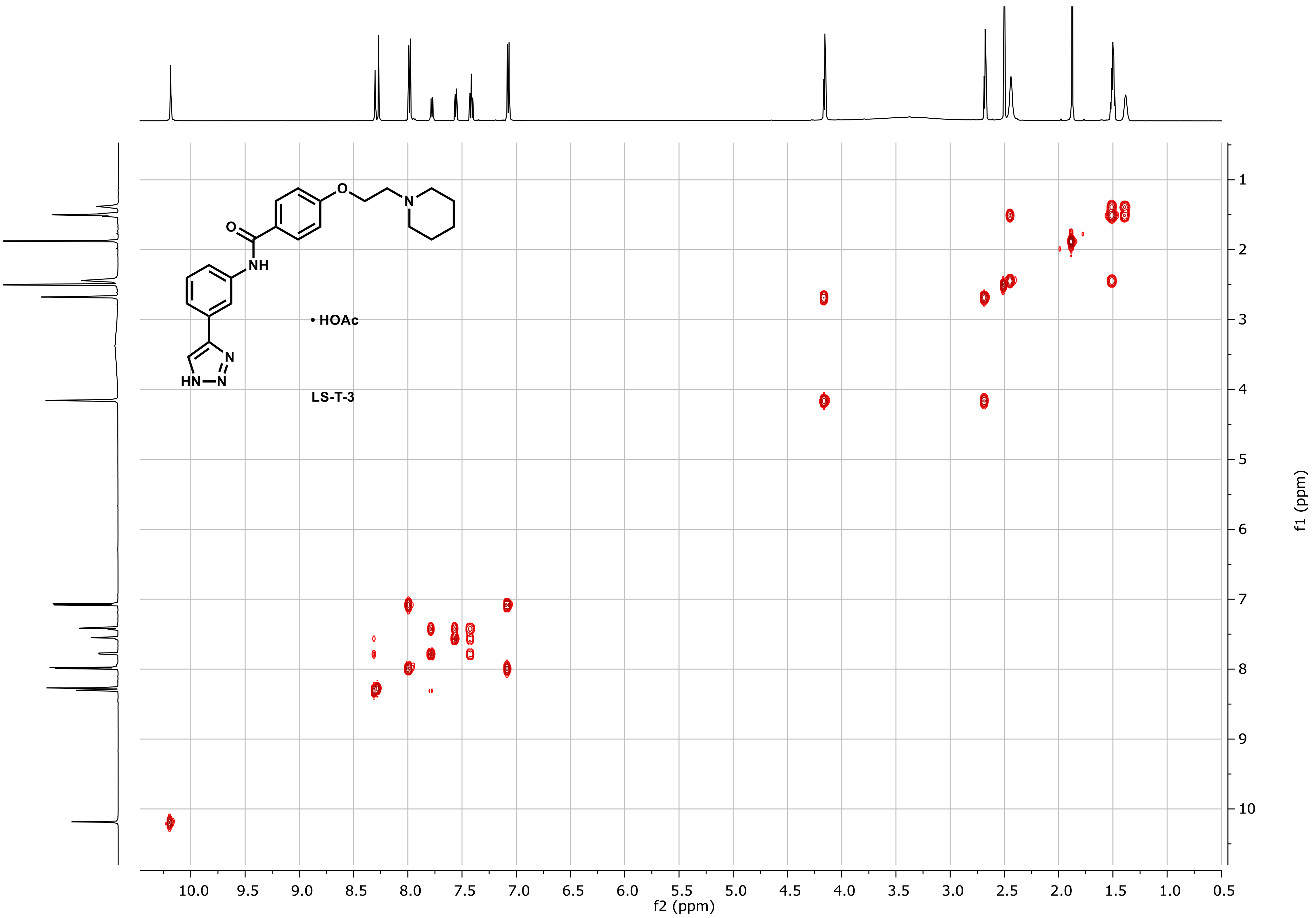

COSY Spectrum for **LS-T-3** (DMSO-*d6*).

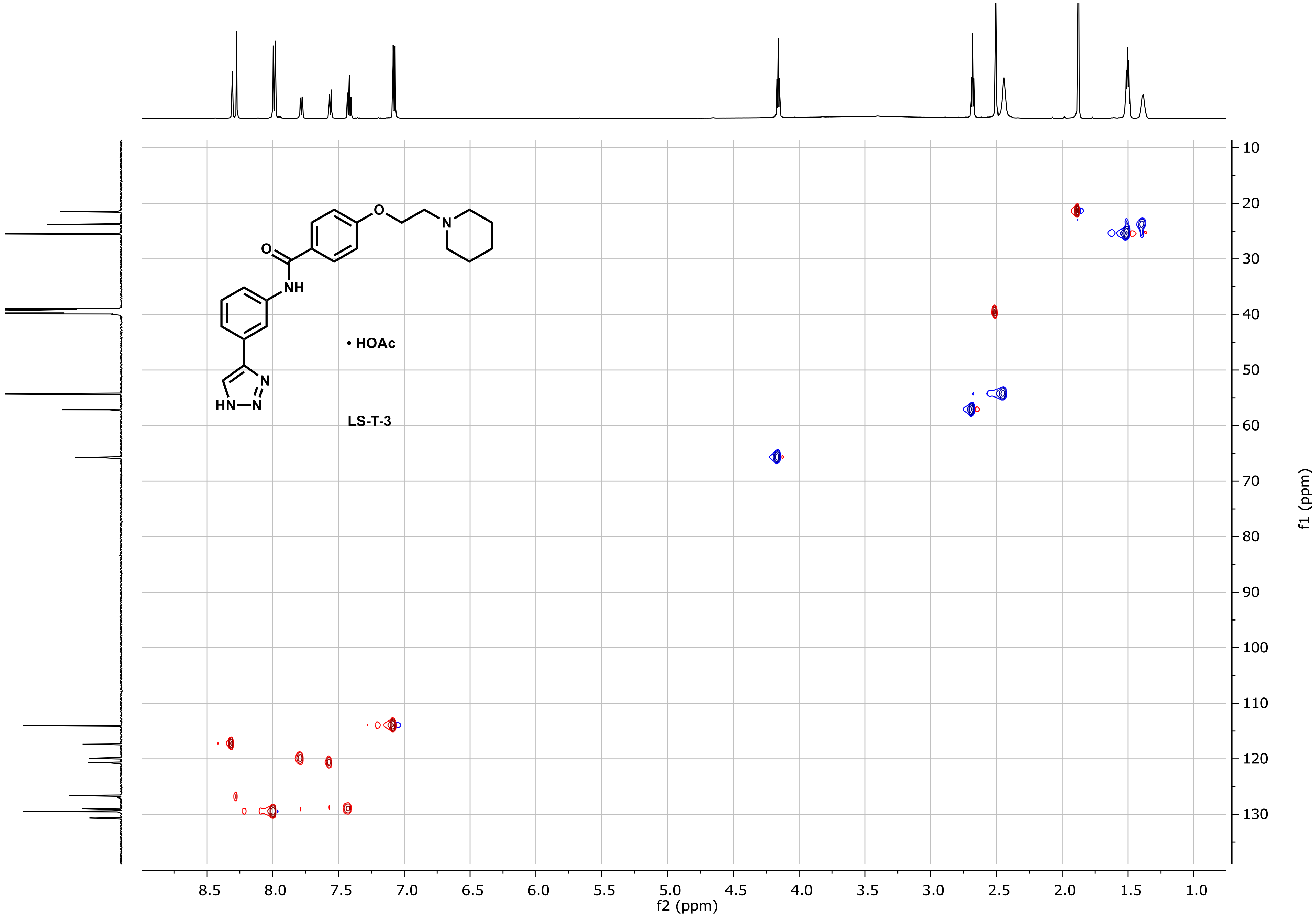

HSQC Spectrum for **LS-T-3** (DMSO-*d6*).

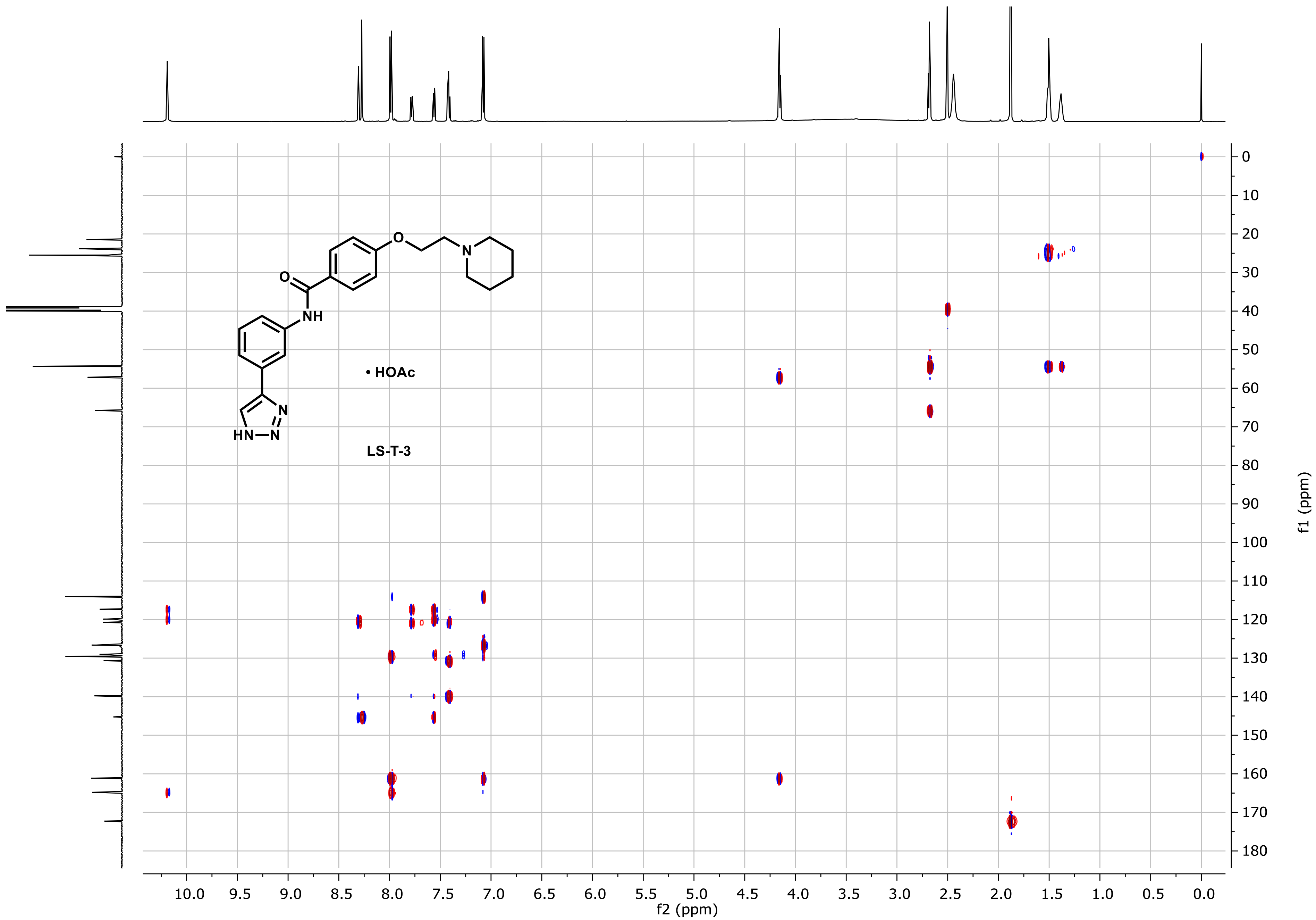

HMBC Spectrum for **LS-T-3** (DMSO-*d6*).

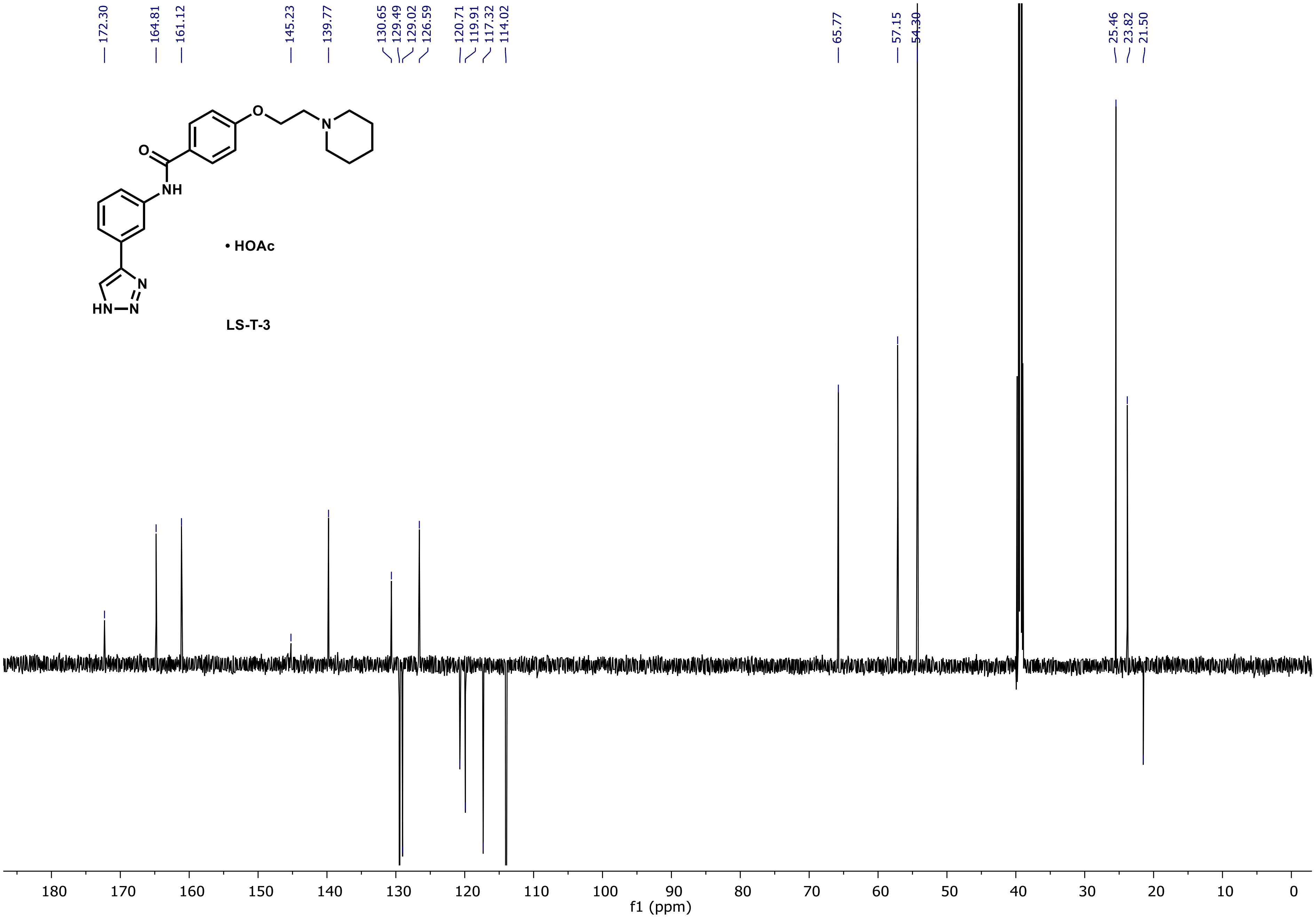

JMOD Spectrum for **LS-T-3** (DMSO-*d6*).

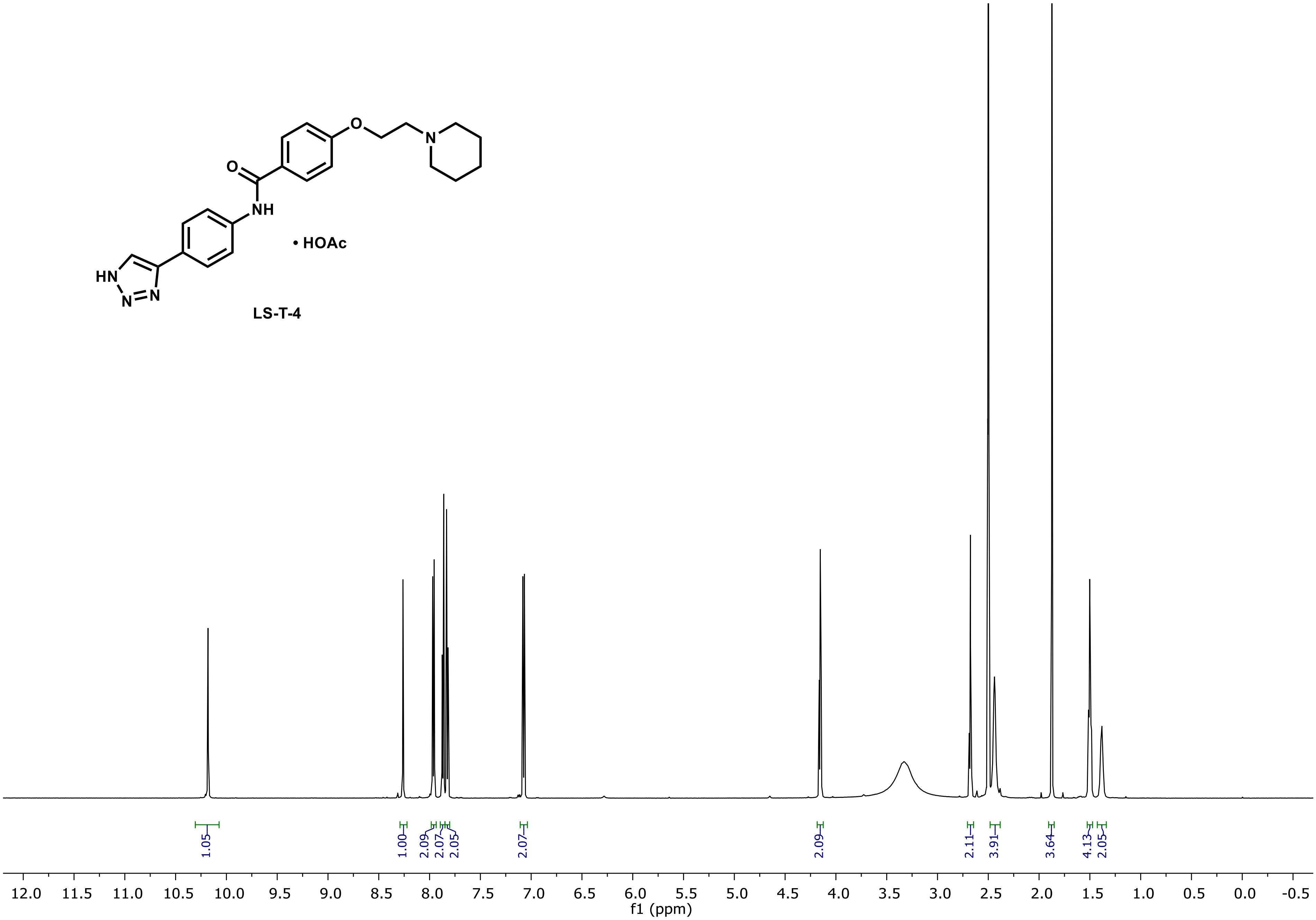

^{1}H NMR Spectrum for **LS-T-4** (DMSO-*d6*, 600 MHz).

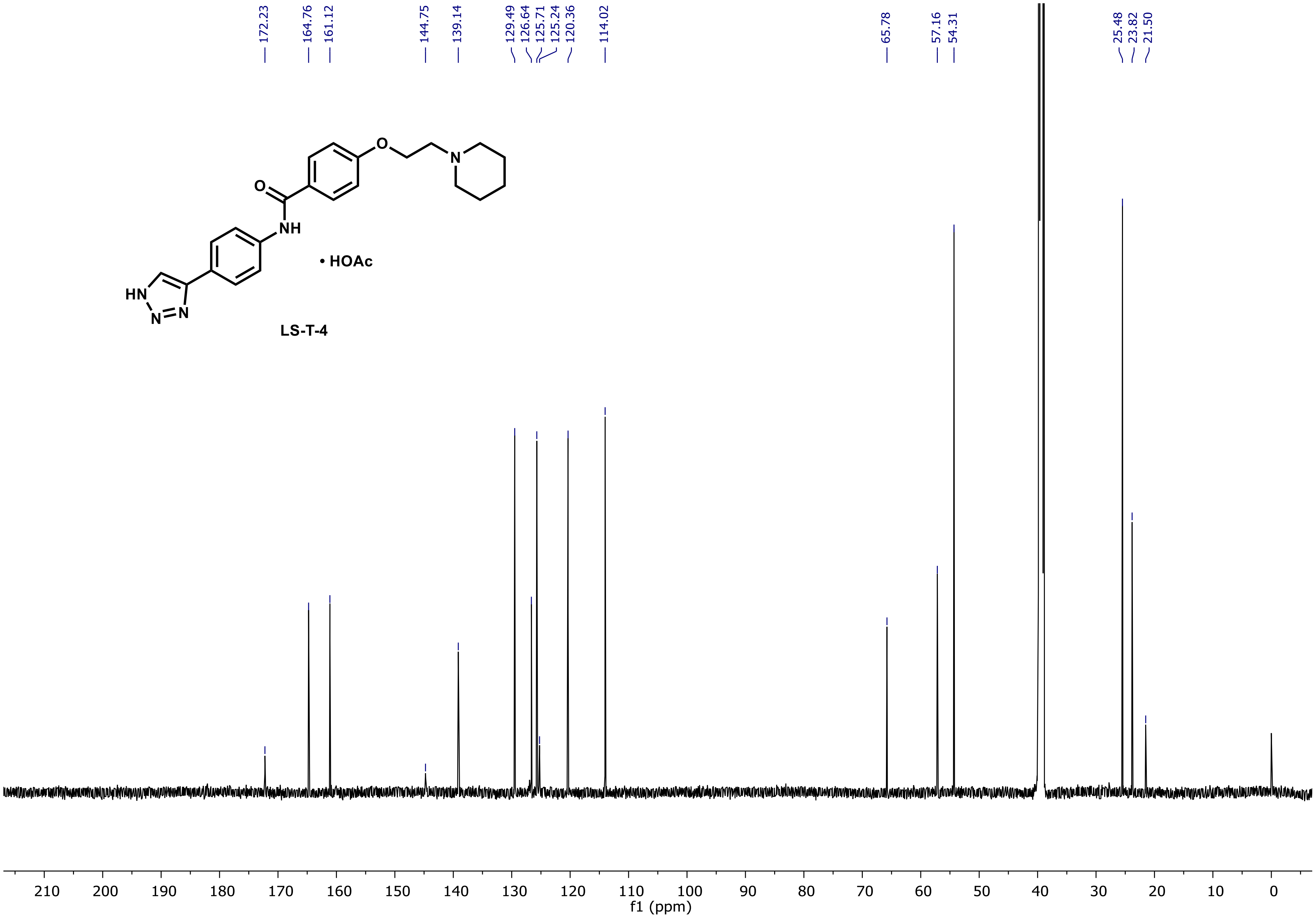

^{13}C NMR Spectrum for **LS-T-4** (DMSO-*d6*, 151 MHz).

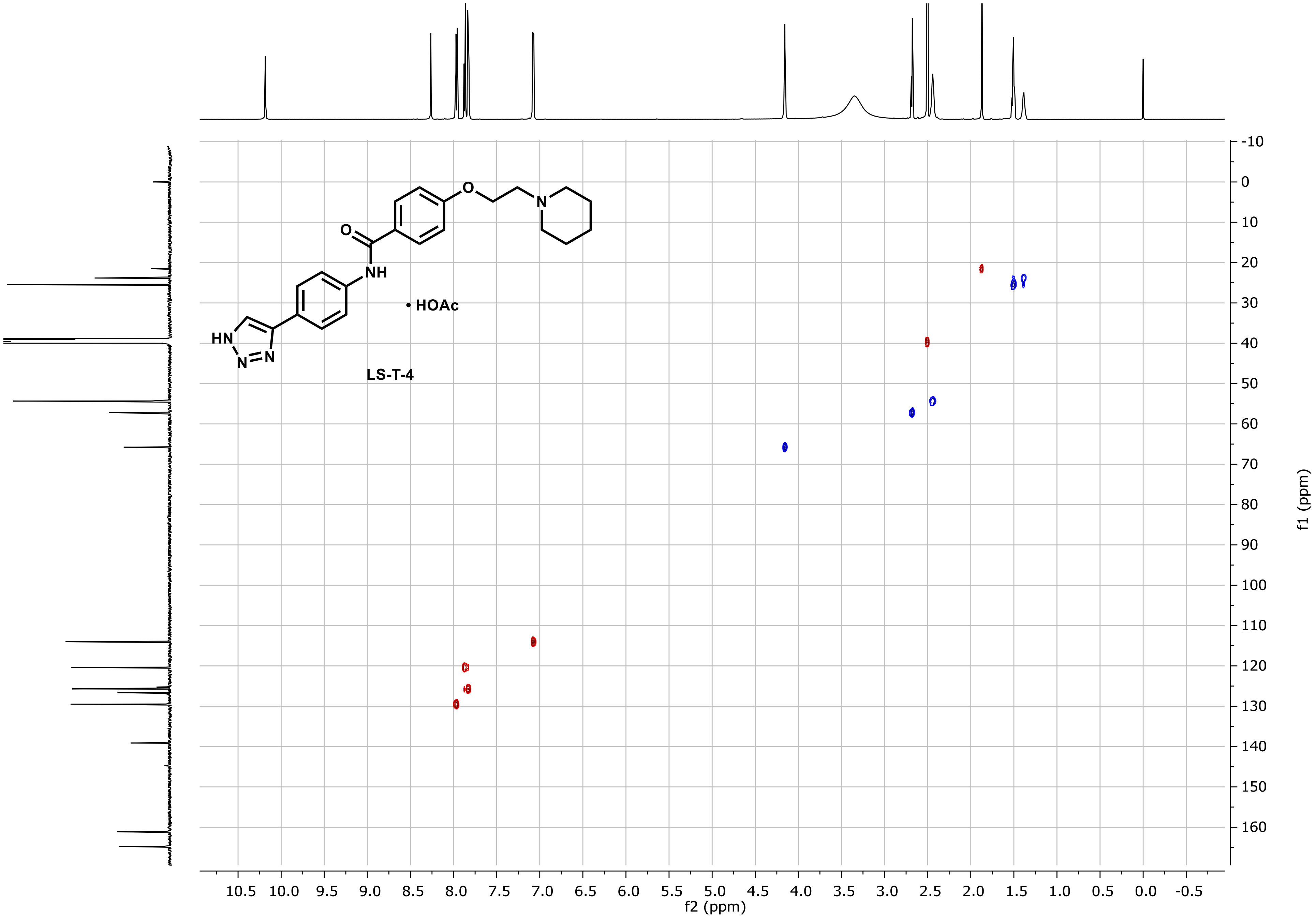

HSQC Spectrum for **LS-T-4** (DMSO-*d6*).

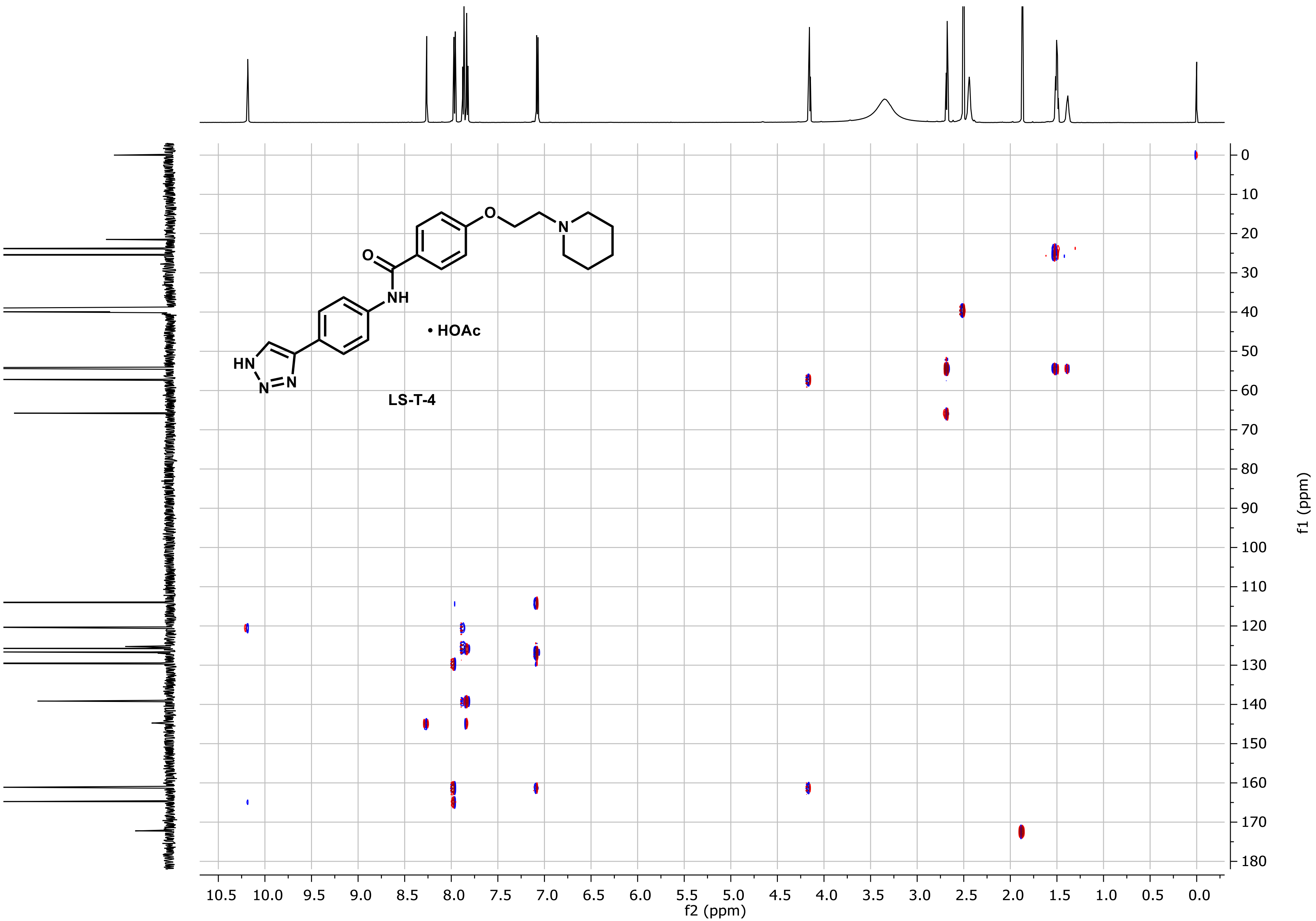

HMBC Spectrum for **LS-T-4** (DMSO-*d6*).

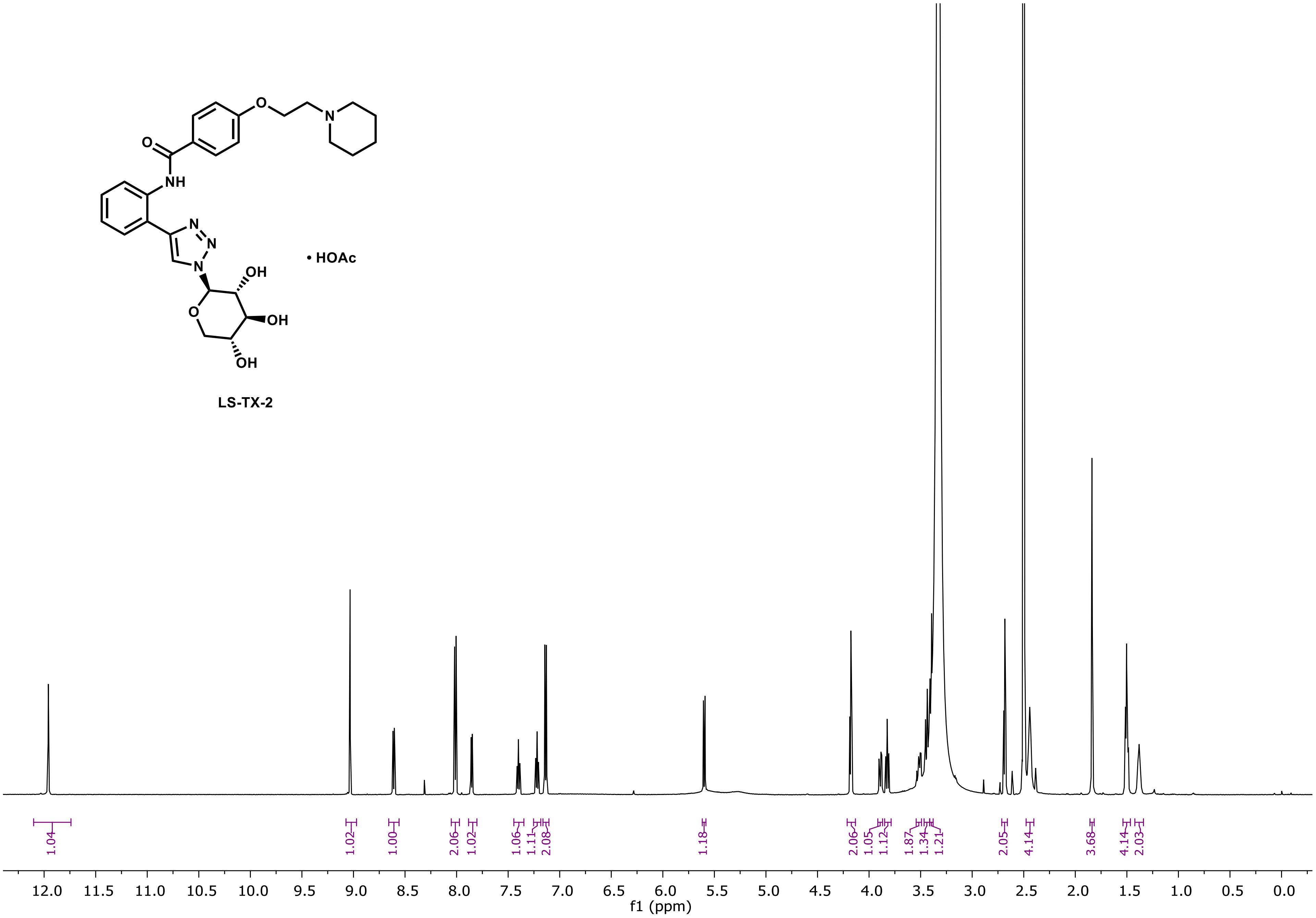

¹H NMR Spectrum for **LS-TX-2** (DMSO-*d6*, 600 MHz).

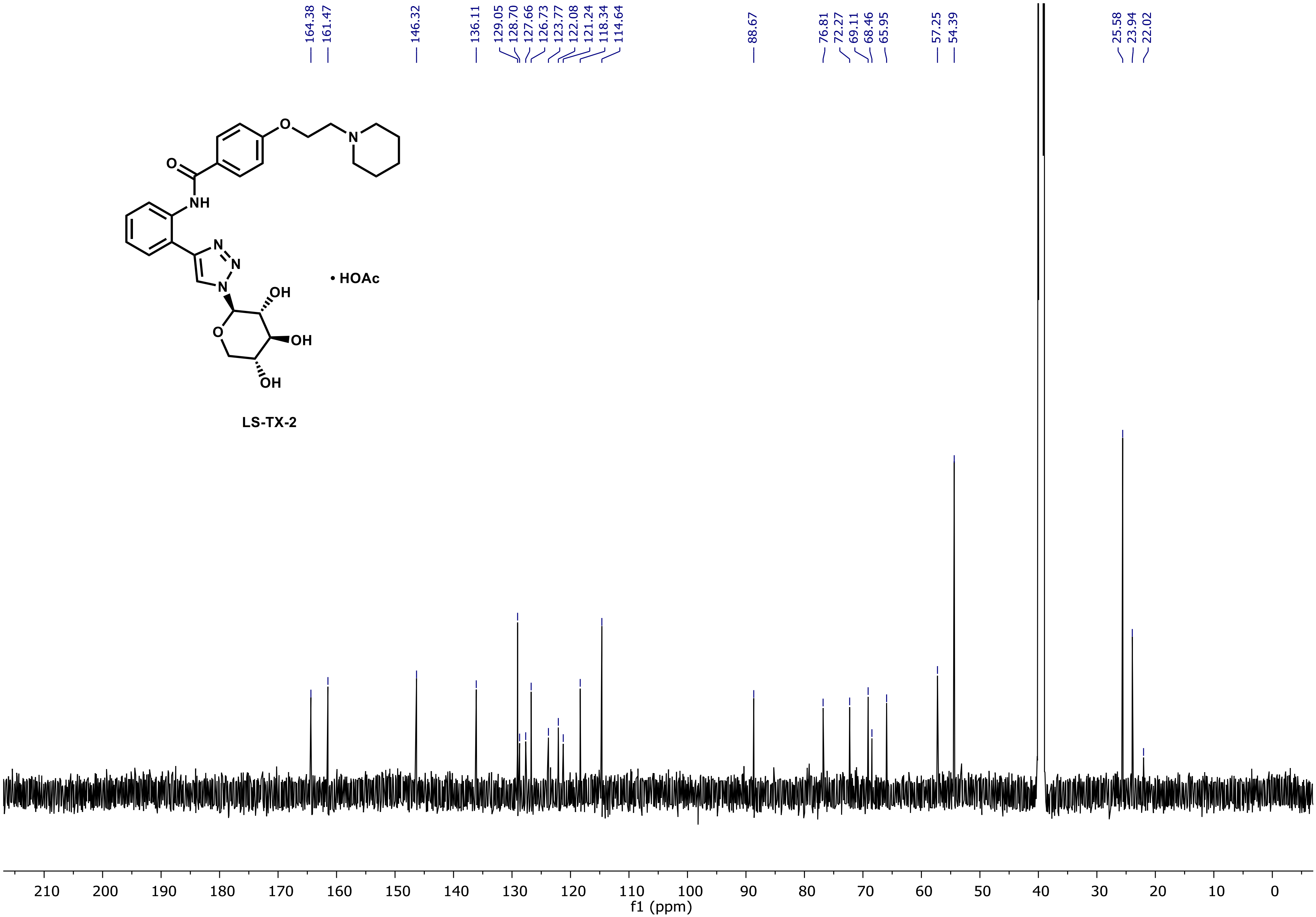

^{13}C NMR Spectrum for **LS-TX-2** (DMSO-*d6*, 151 MHz).

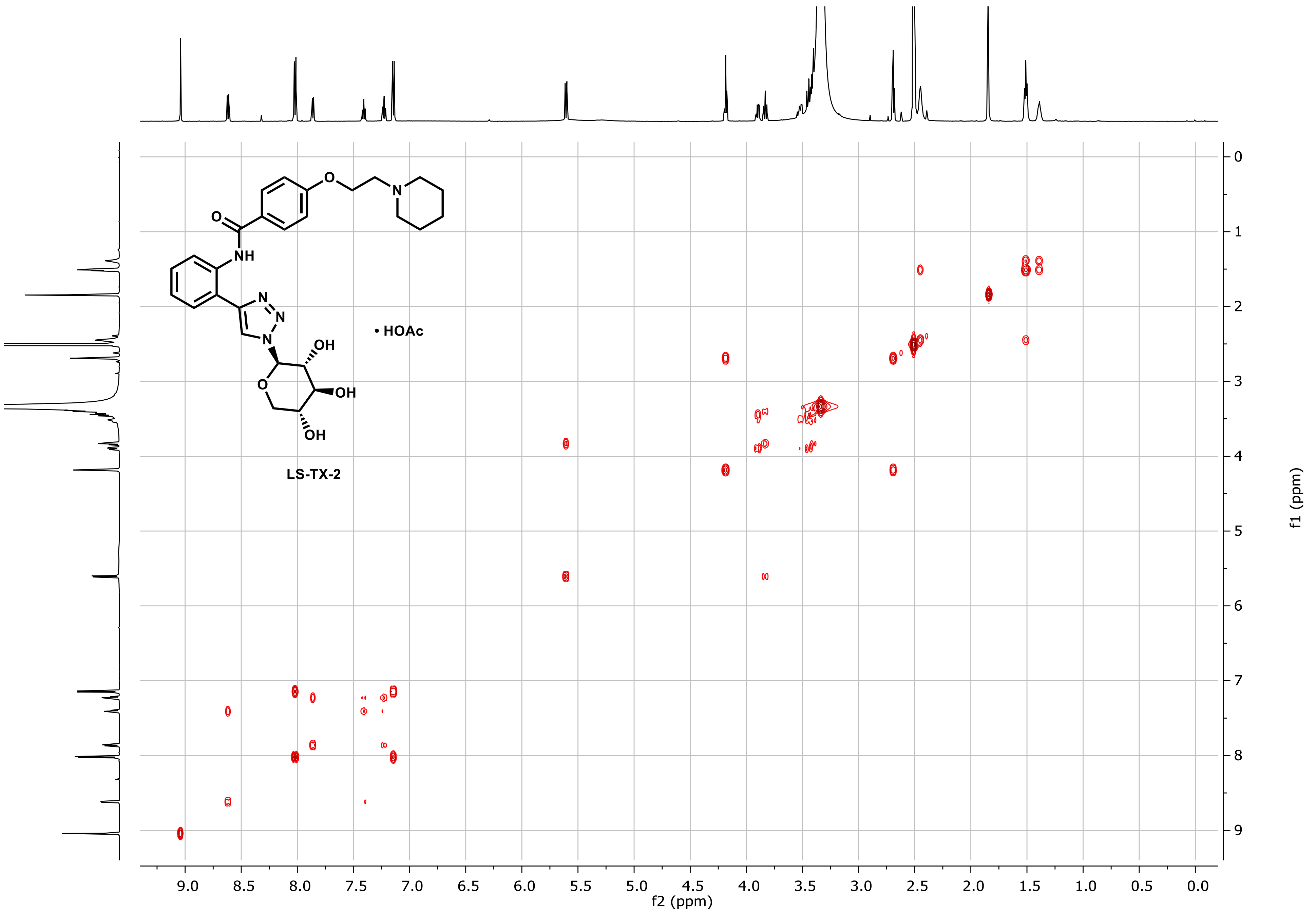

COSY Spectrum for **LS-TX-2** (DMSO-*d6*).

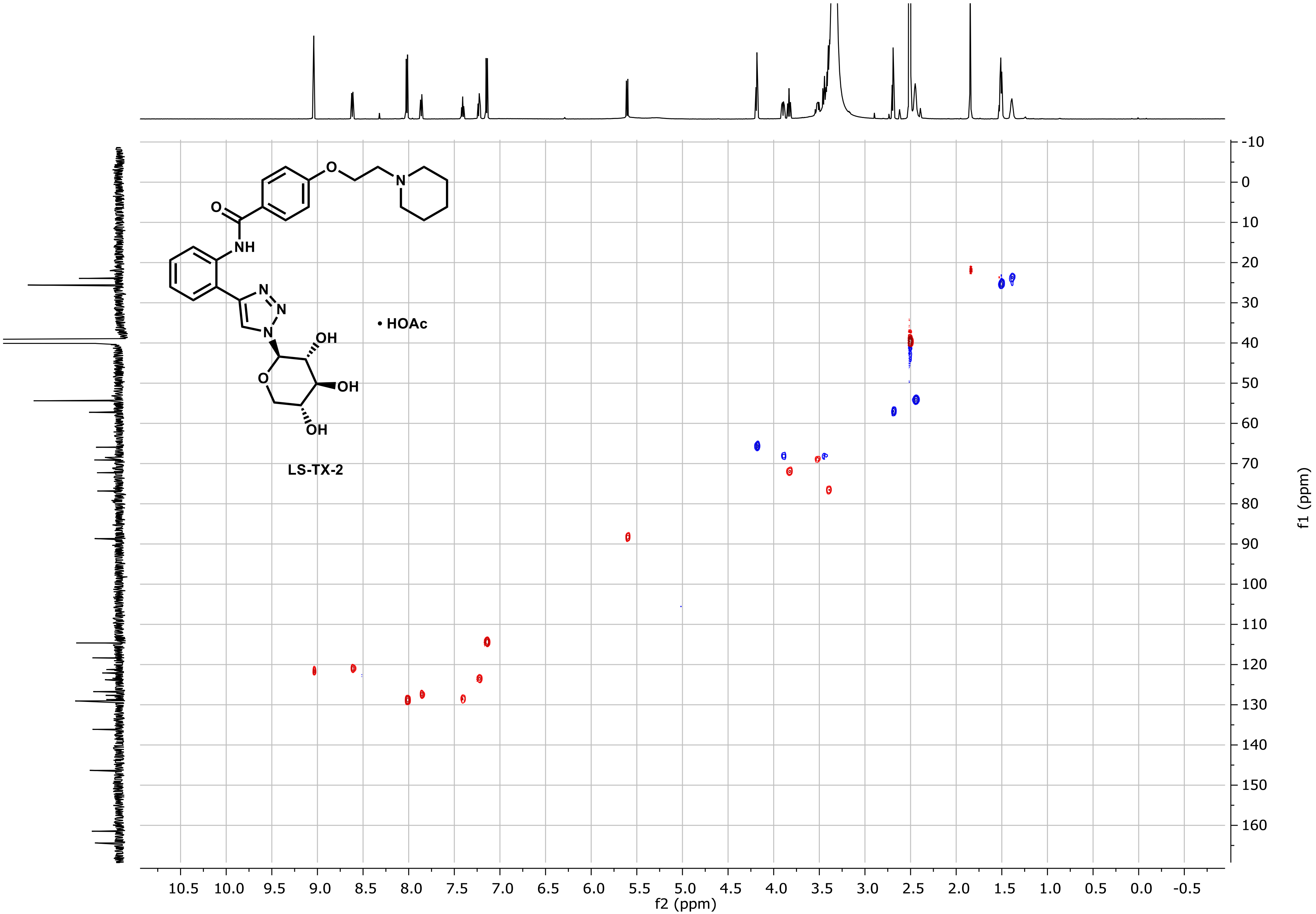

HSQC Spectrum for **LS-TX-2** (DMSO-*d6*).

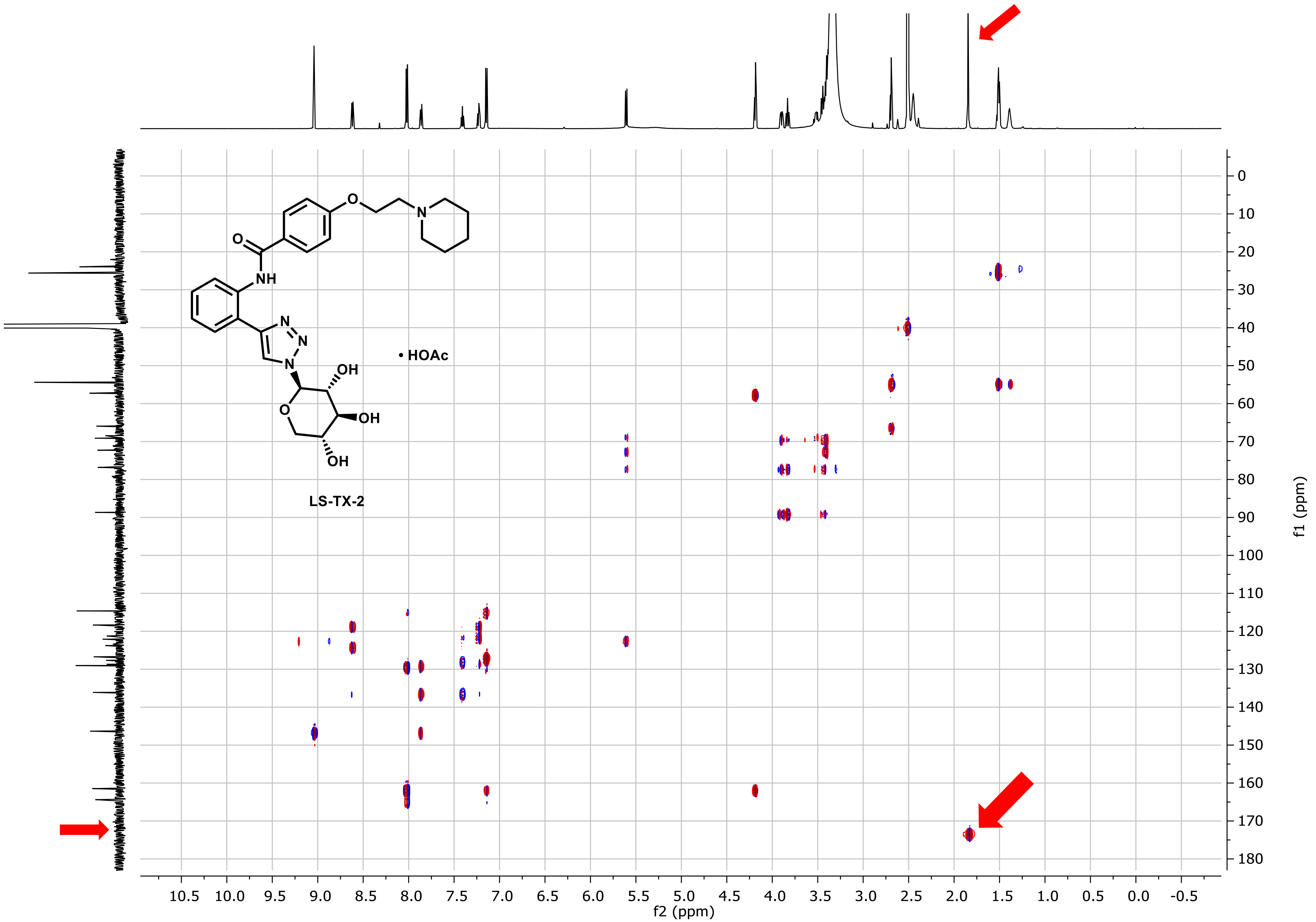

HMBC Spectrum for **LS-TX-2** (DMSO-*d6*). Note correlation of acetate CH_3 to the missing 172.0 ppm ^{13}C signal of the acetate carbonyl carbon.

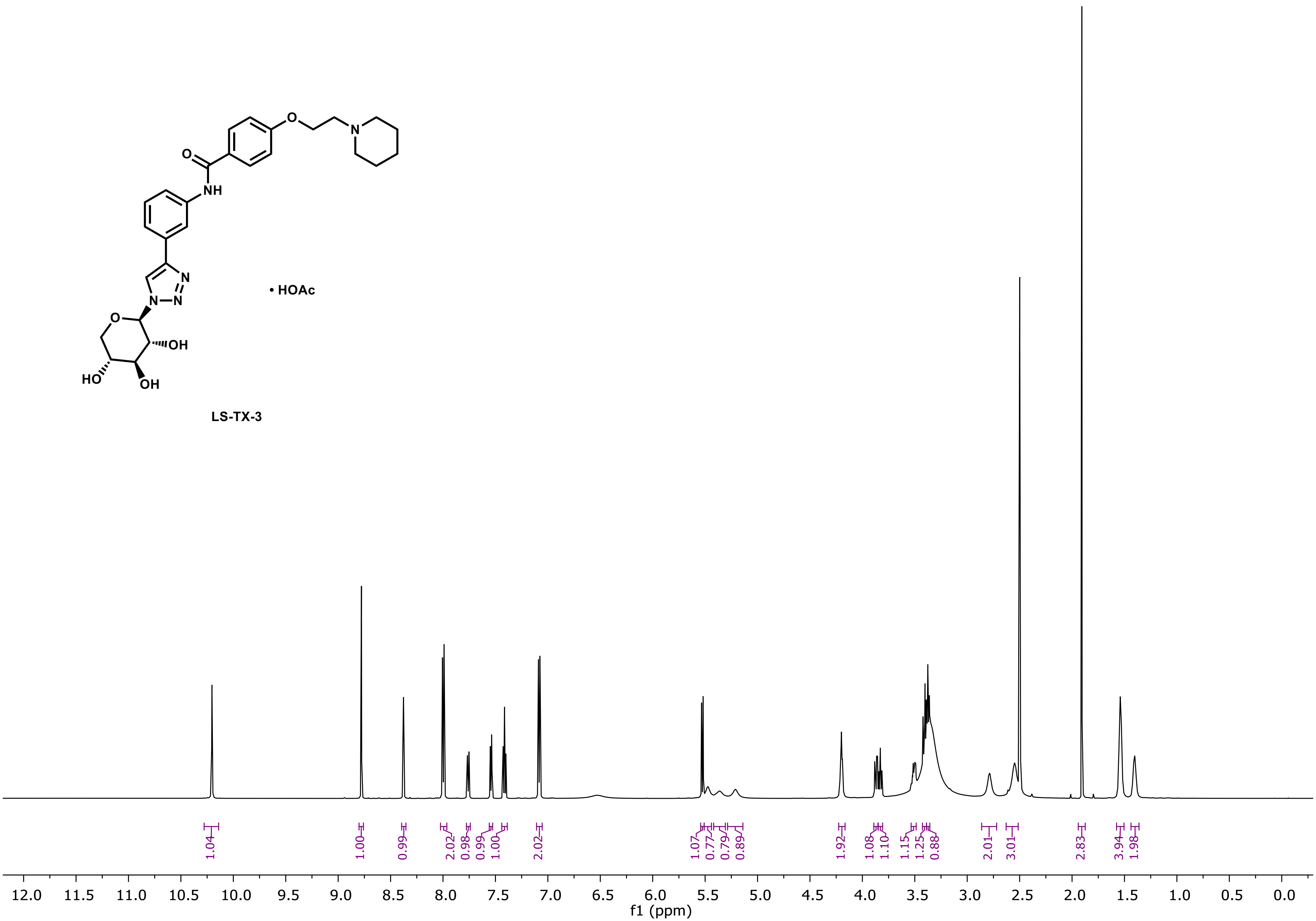

^{1}H NMR Spectrum for **LS-TX-3** (DMSO-*d6*, 600 MHz).

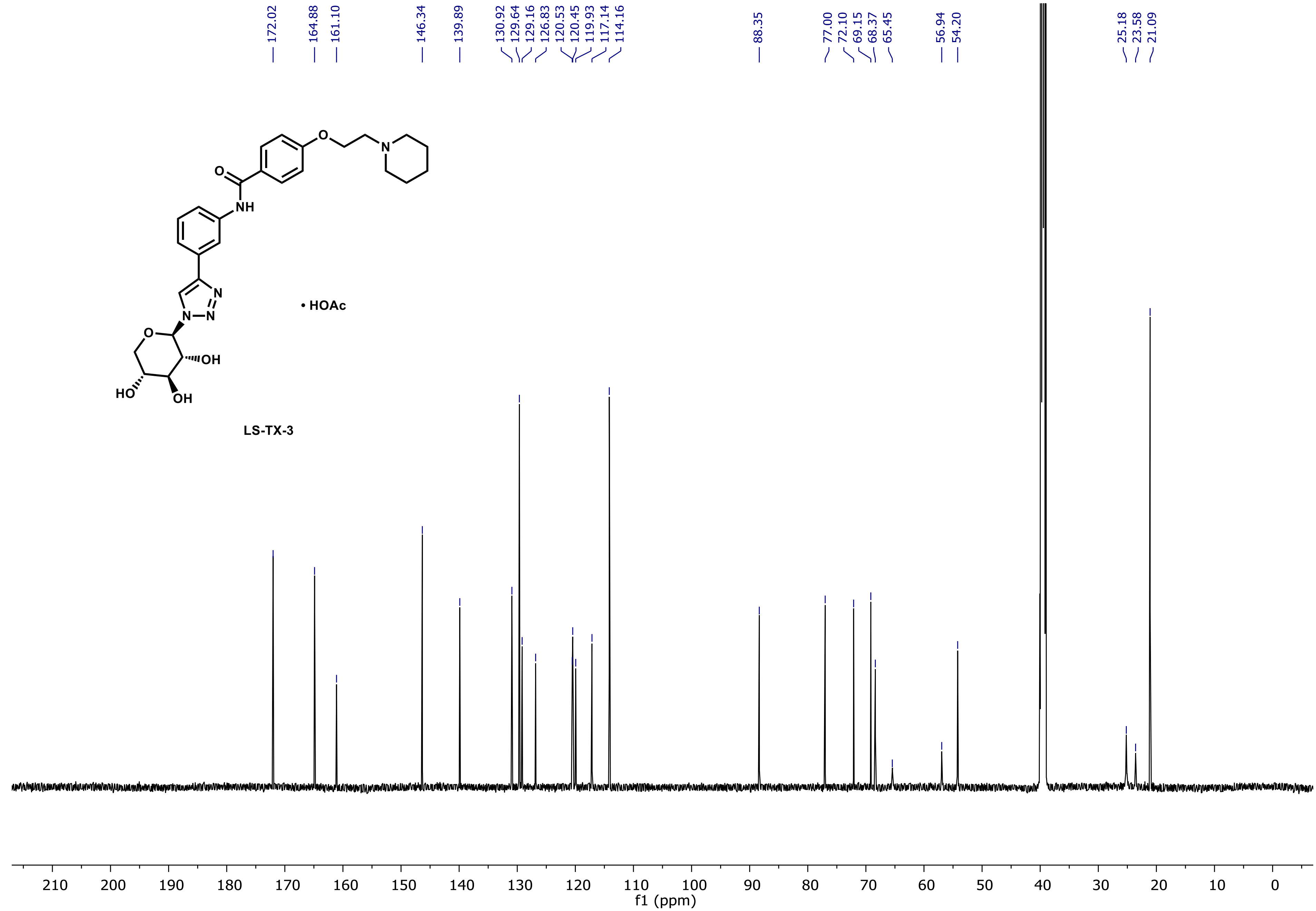

^{13}C NMR Spectrum for **LS-TX-3** (DMSO-*d6*, 151 MHz).

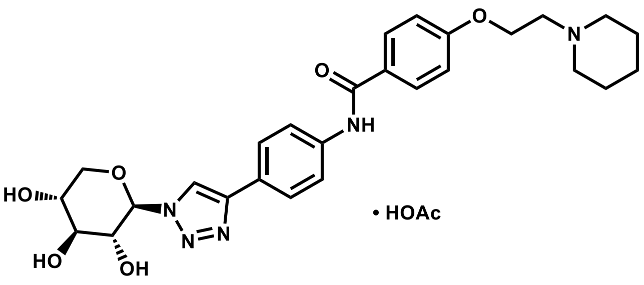

LS-TX-4

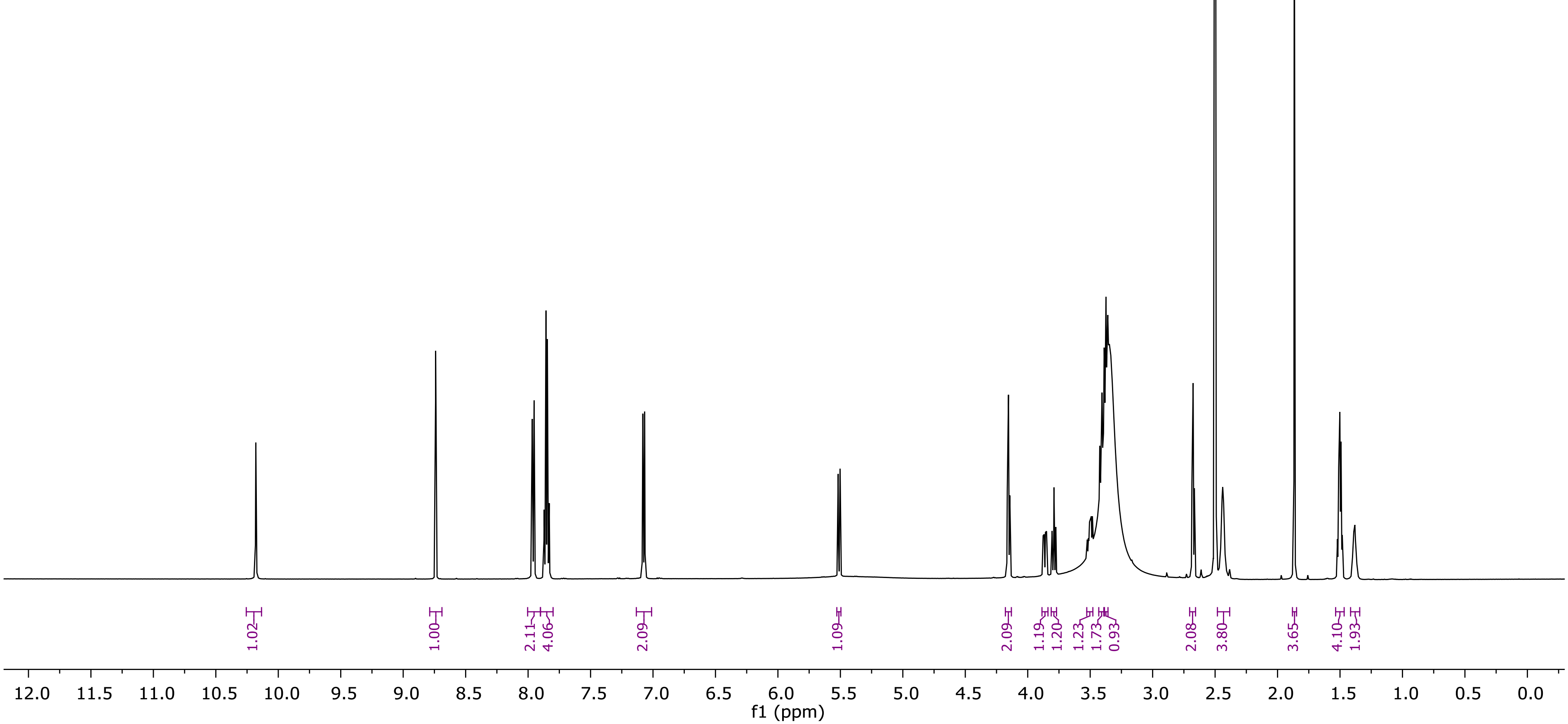

^{1}H NMR Spectrum for **LS-TX-4** (DMSO-*d6*, 600 MHz).

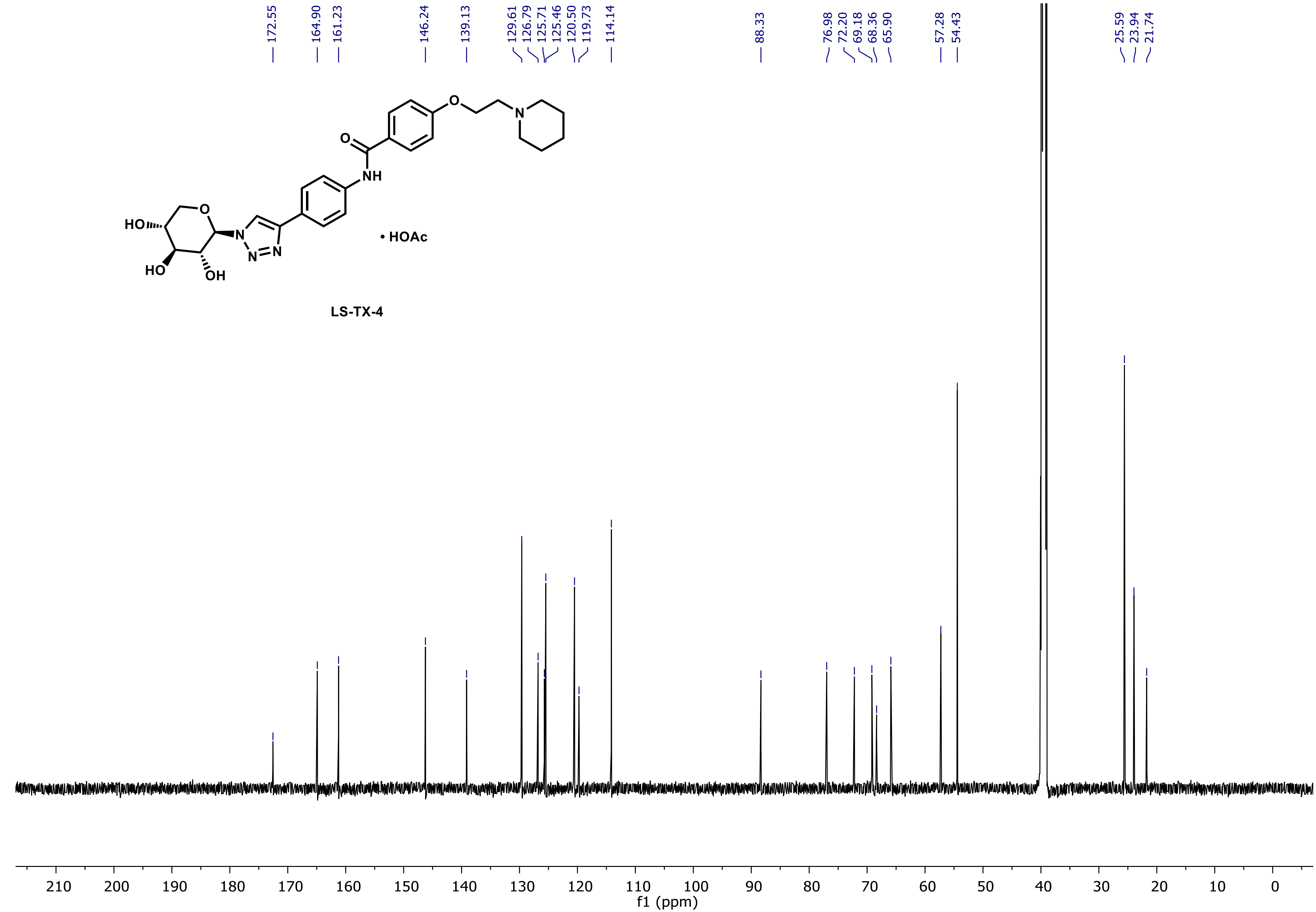

^{13}C NMR Spectrum for **LS-TX-4** (DMSO-*d6*, 151 MHz).

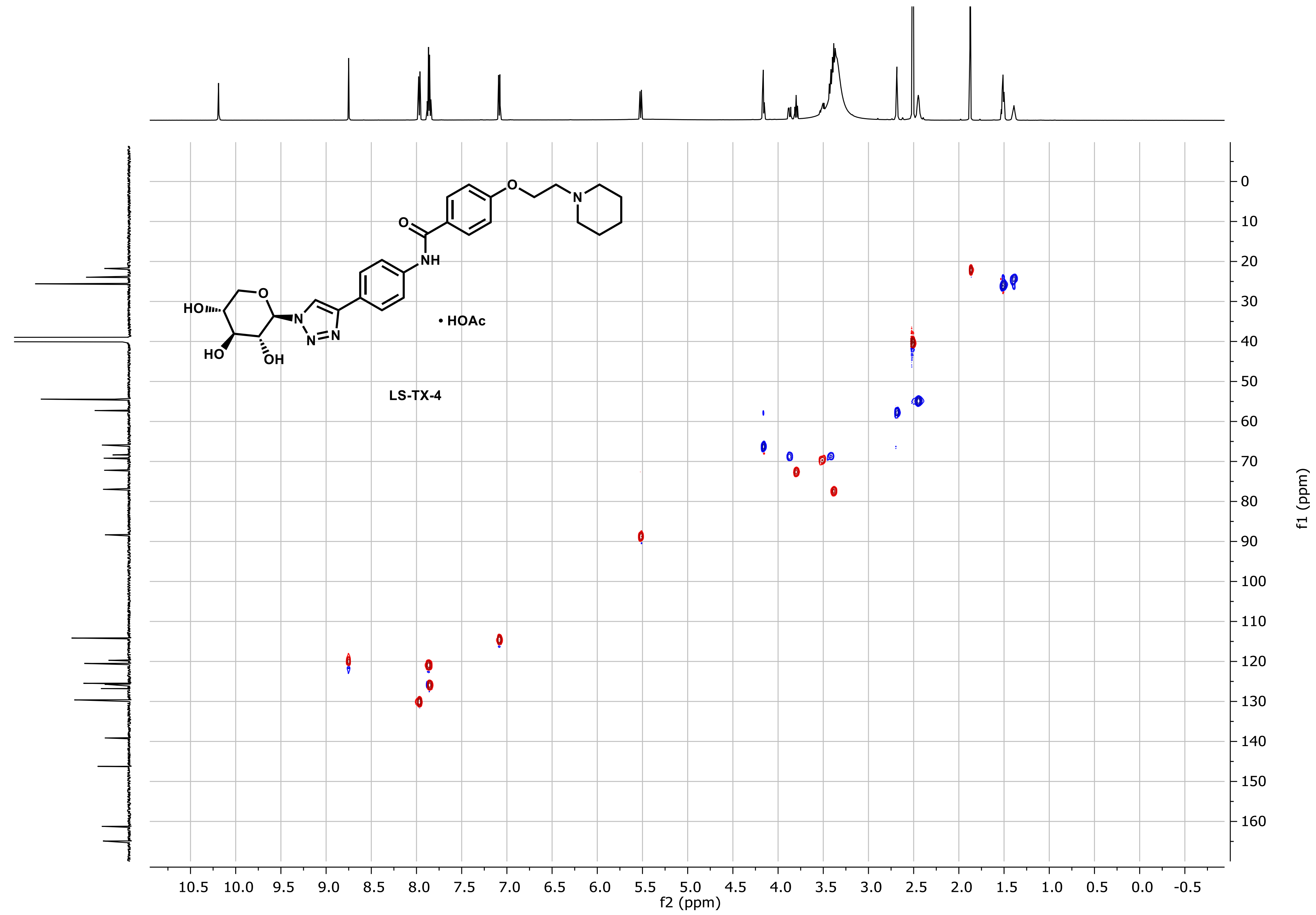

HSQC Spectrum for **LS-TX-4** (DMSO-*d6*).

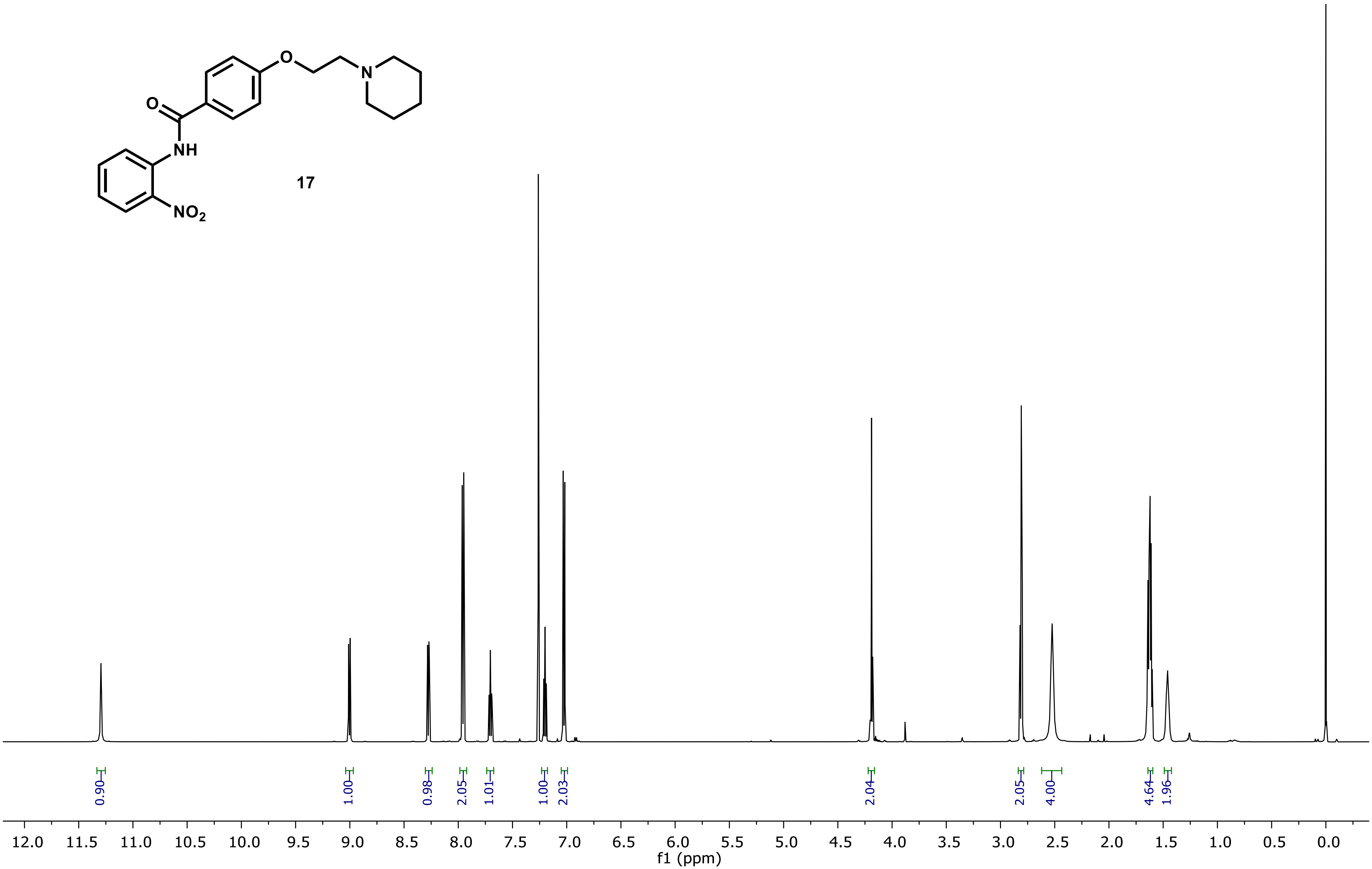

^{1}H NMR Spectrum for Compound **17** ($CDCl_3$, 600 MHz).

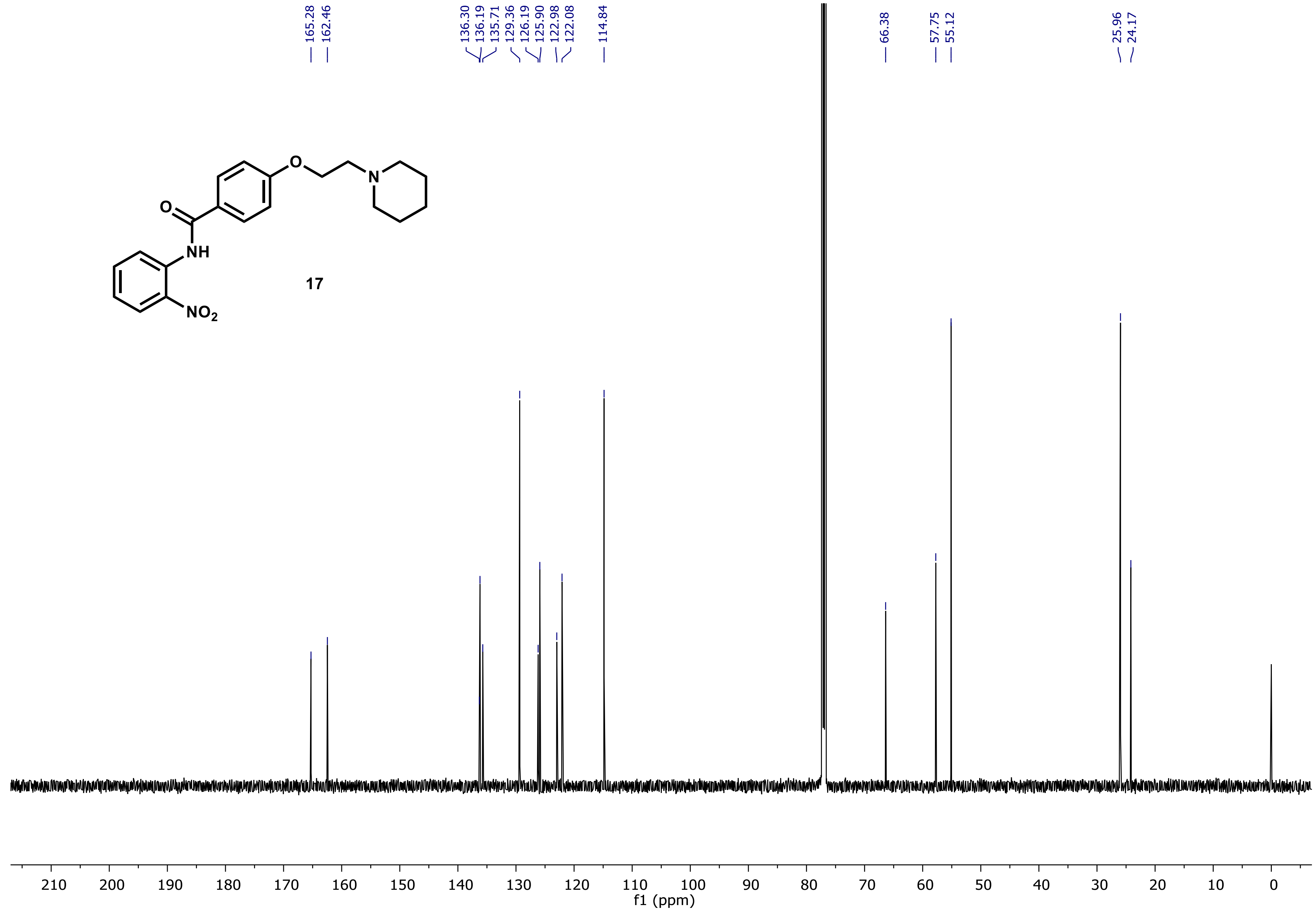

^{13}C NMR Spectrum for Compound **17** ($CDCl_3$, 151 MHz).

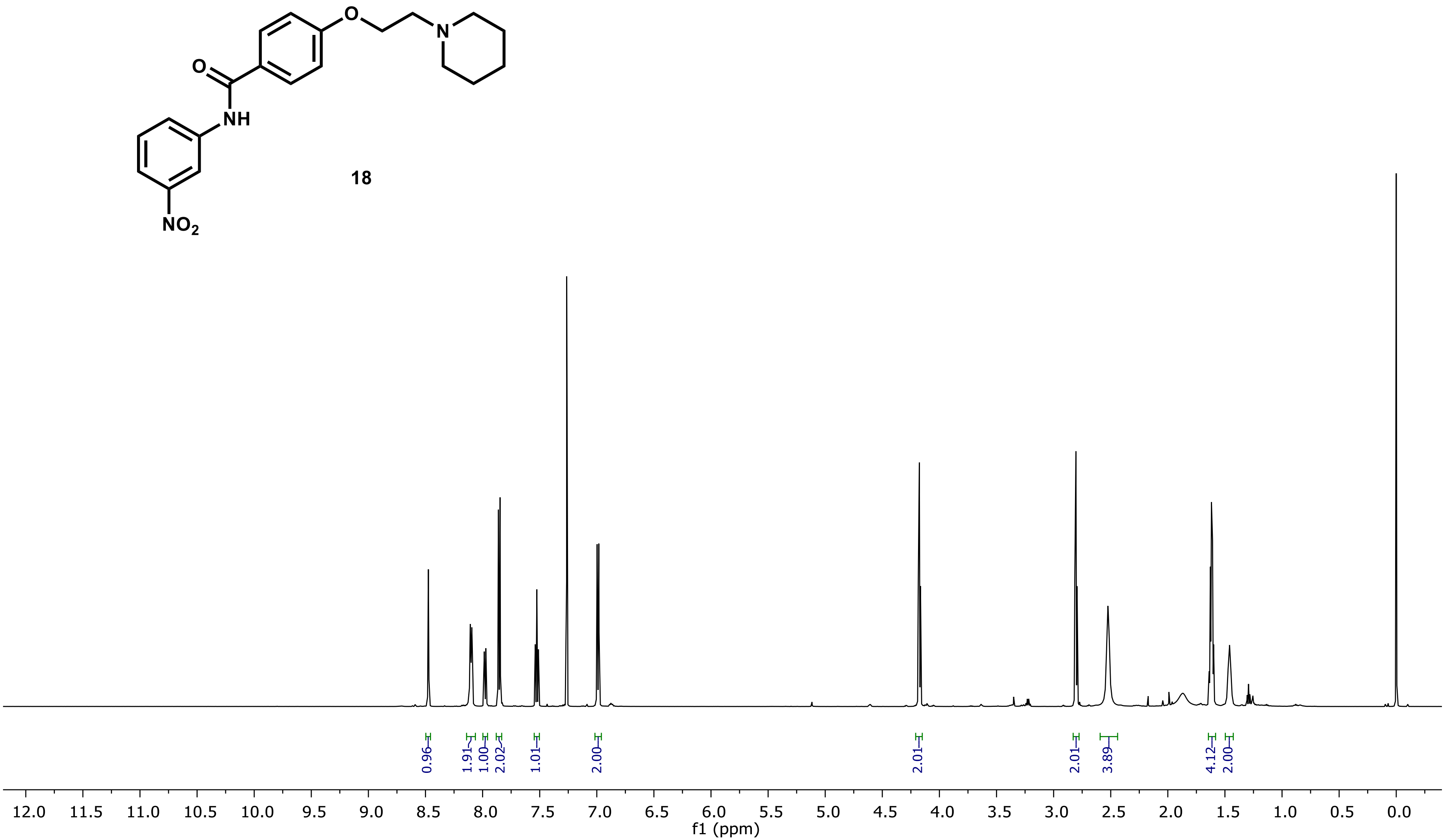

^{1}H NMR Spectrum for Compound **18** ($CDCl_3$, 600 MHz).

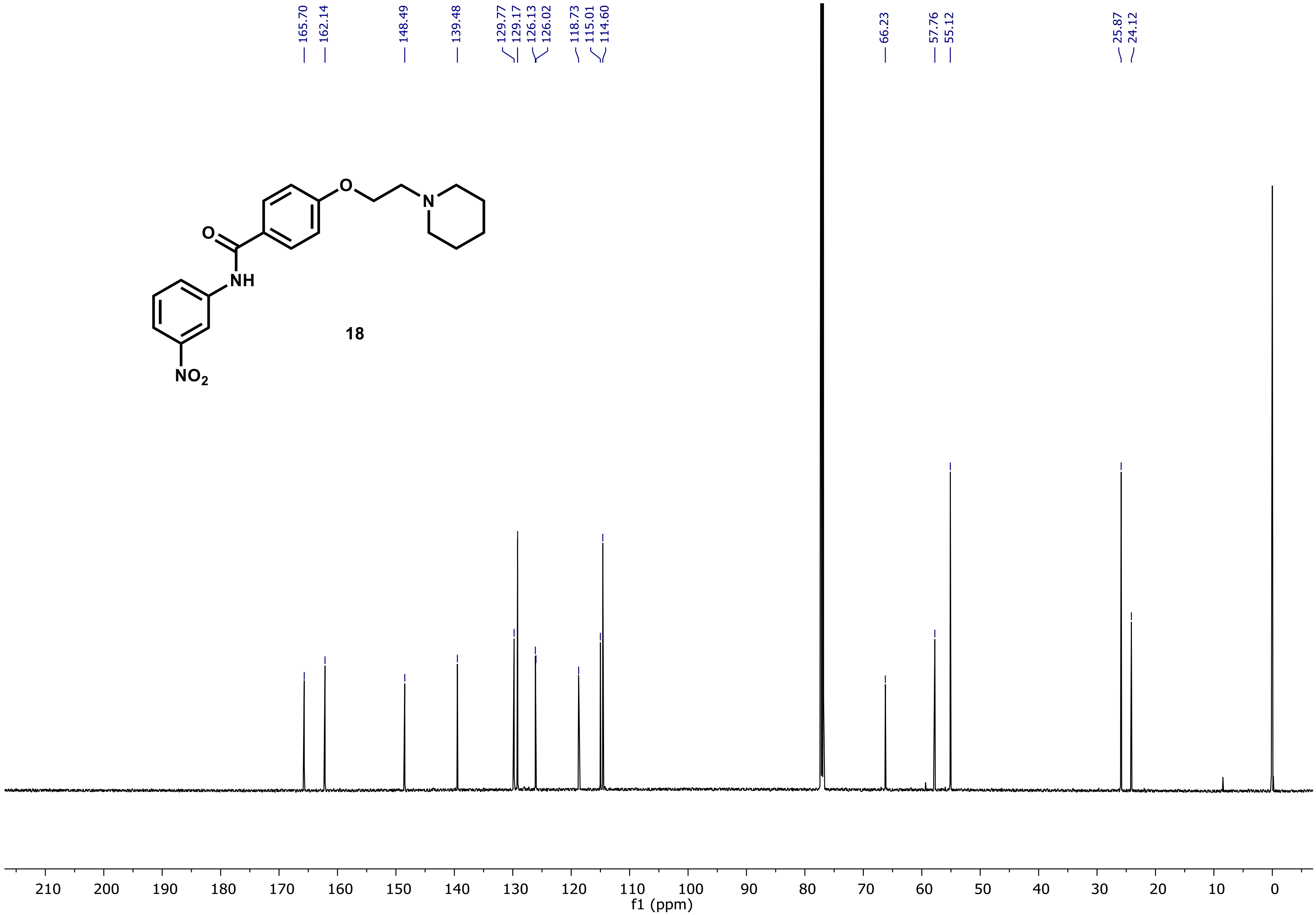

18

^{13}C NMR Spectrum for Compound **18** ($CDCl_3$, 151 MHz).

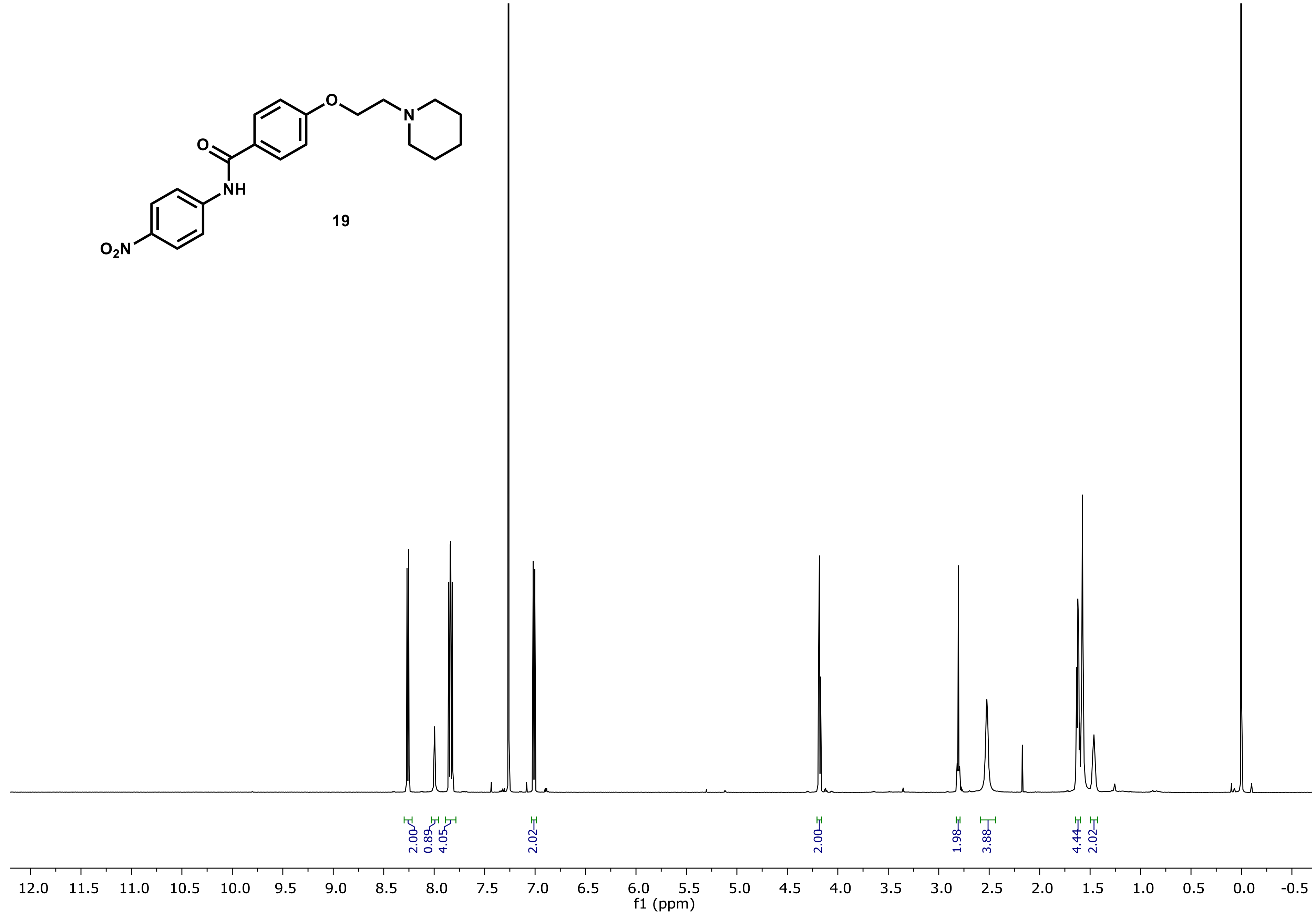

^{1}H NMR Spectrum for Compound **19** ($CDCl_3$, 600 MHz).

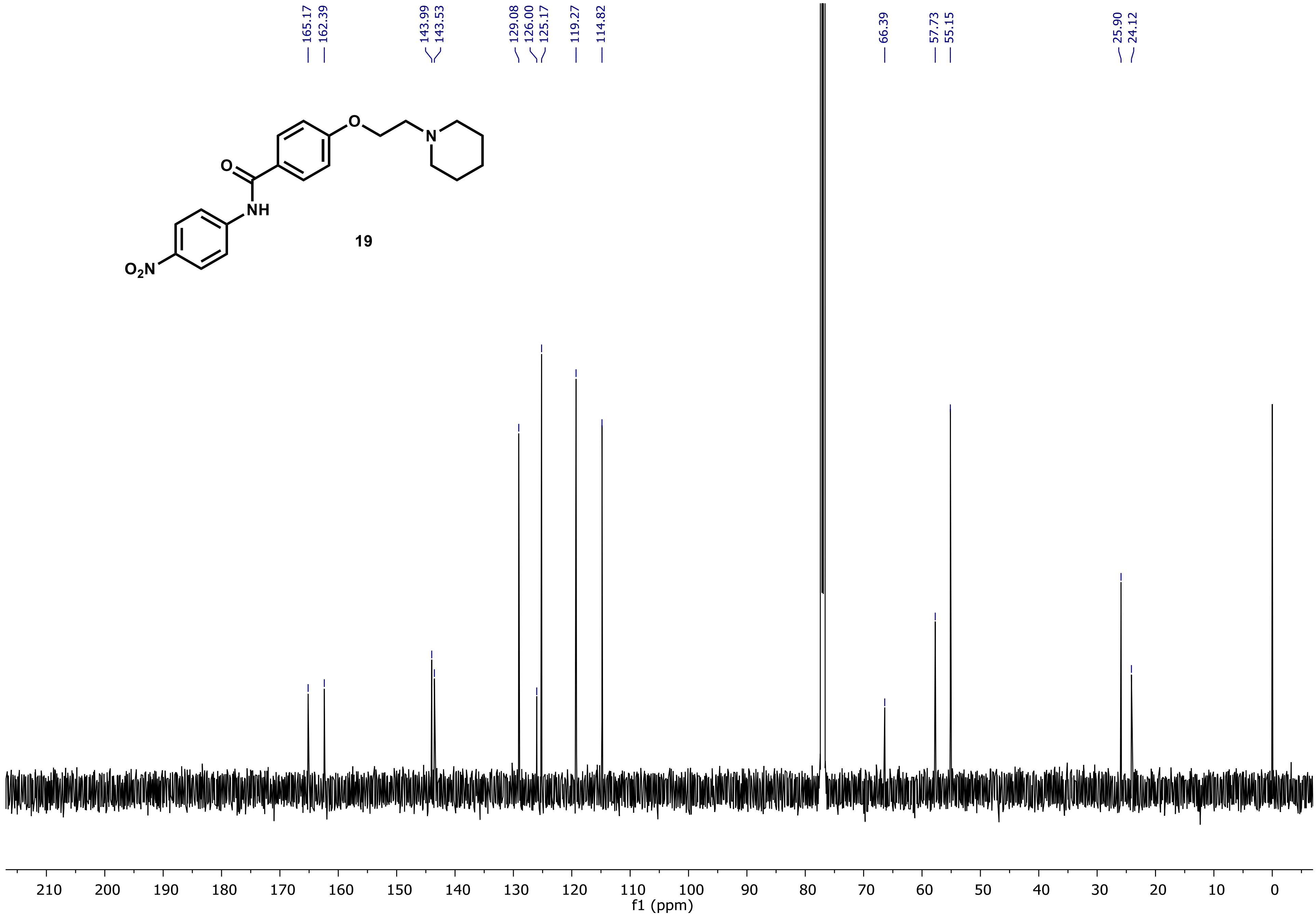

^{13}C NMR Spectrum for Compound **19** ($CDCl_3$, 151 MHz).

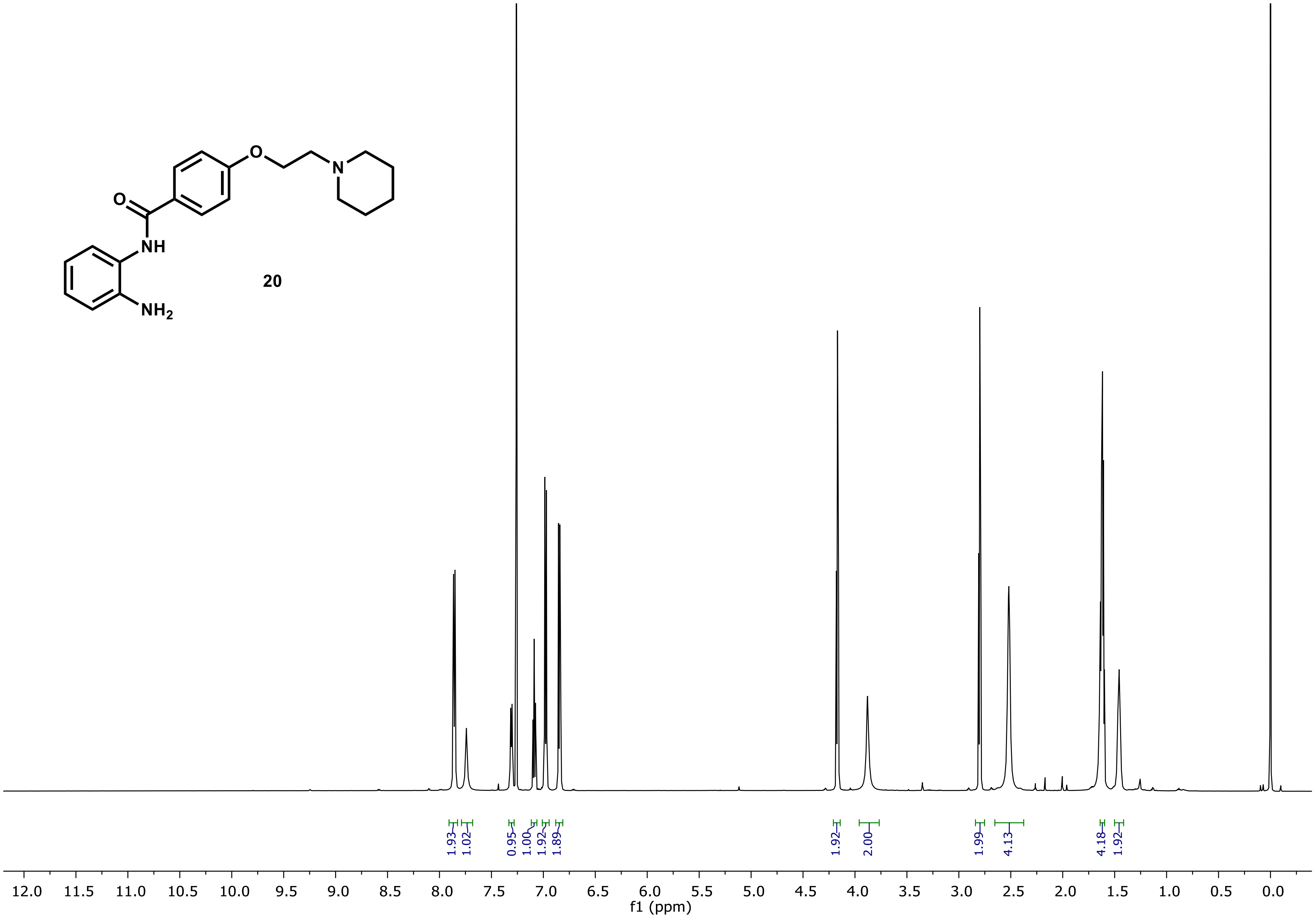

^{1}H NMR Spectrum for Compound **20** ($CDCl_3$, 600 MHz).

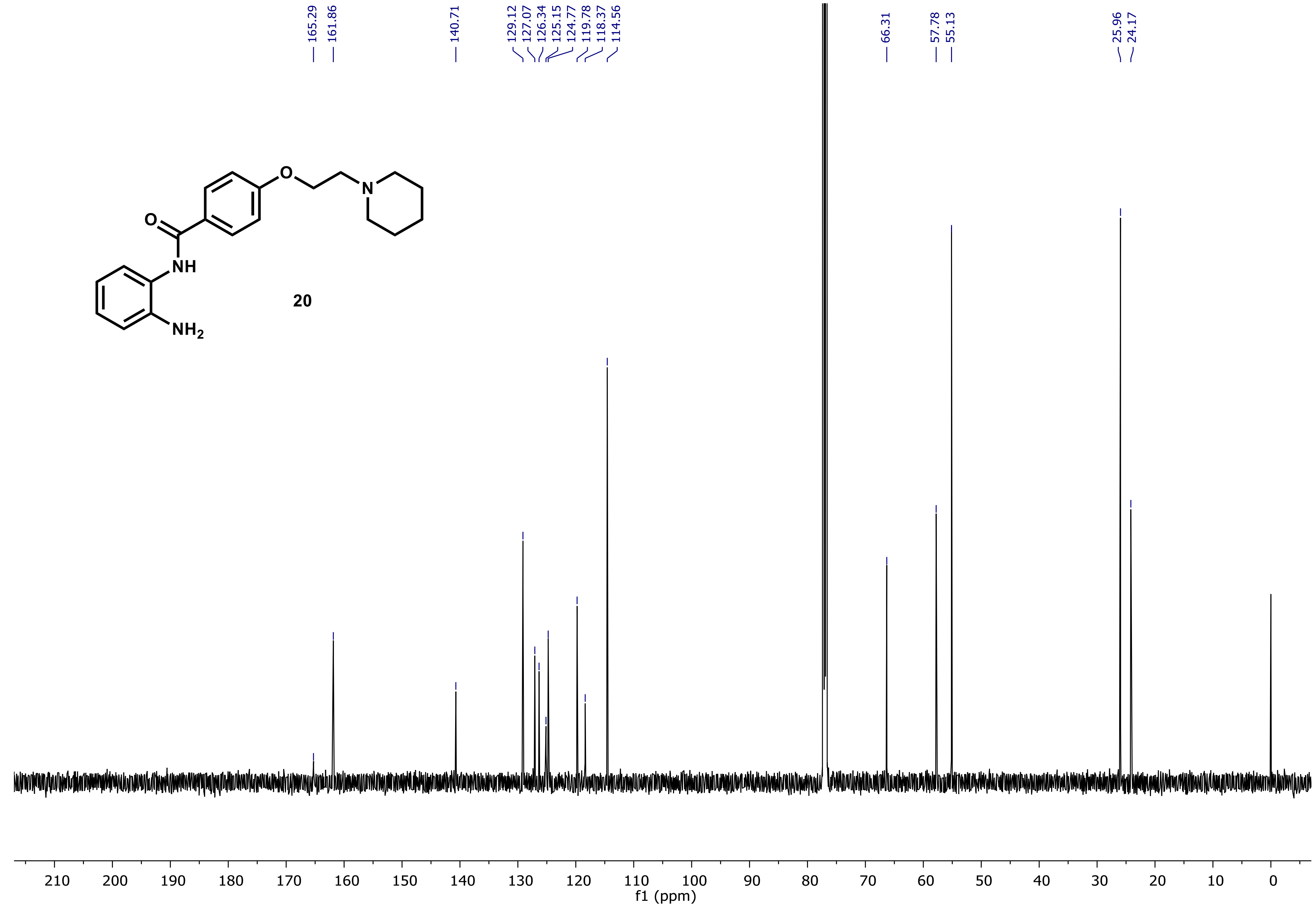

^{13}C NMR Spectrum for Compound **20** ($CDCl_3$, 151 MHz).

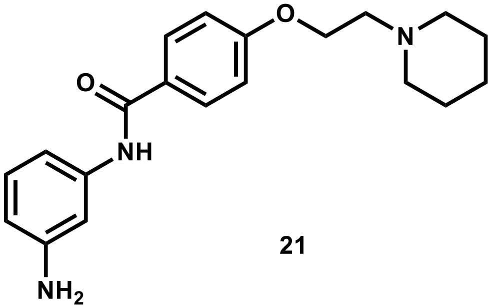

21

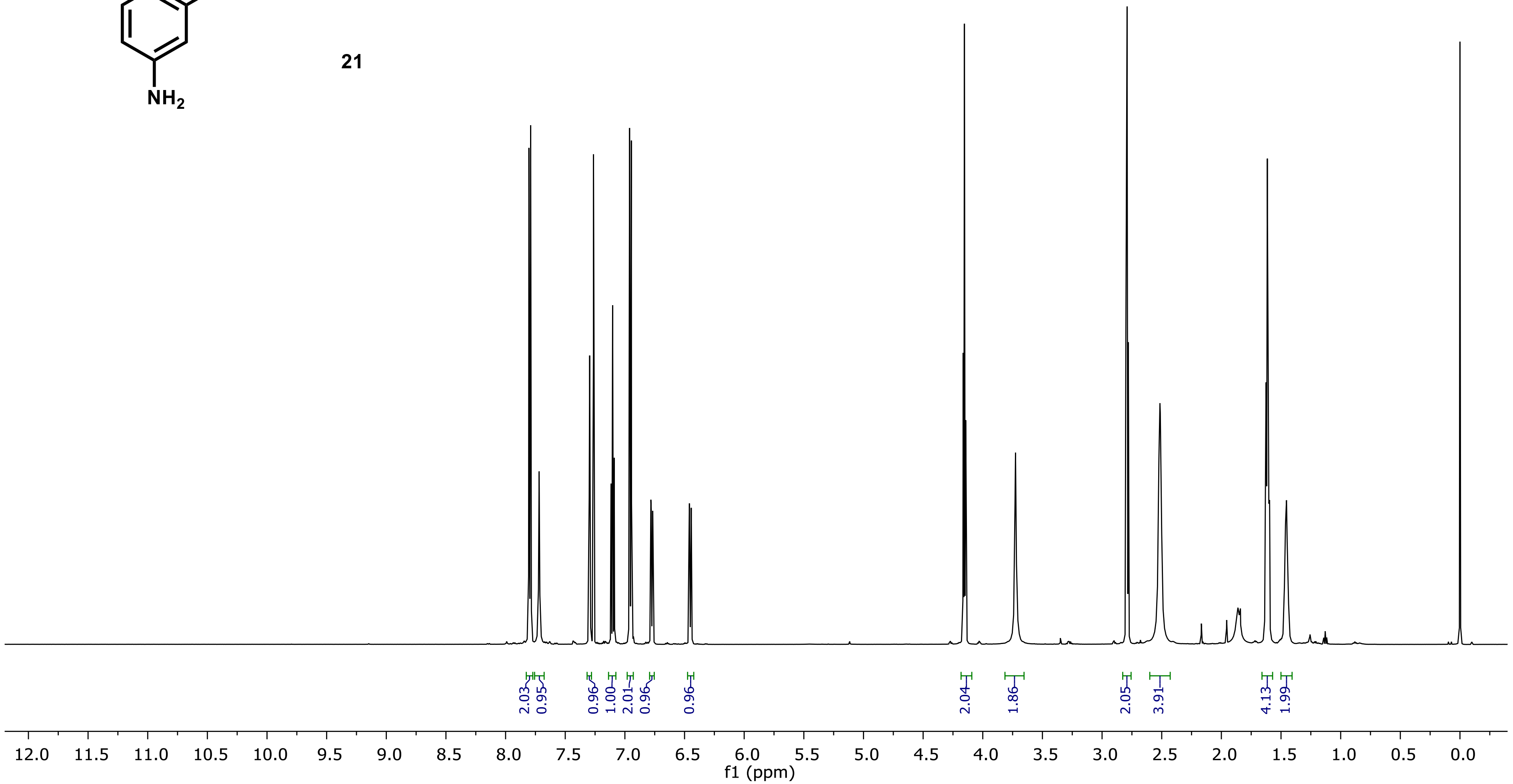

^1H NMR Spectrum for Compound **21** ($CDCl_3$, 600 MHz).

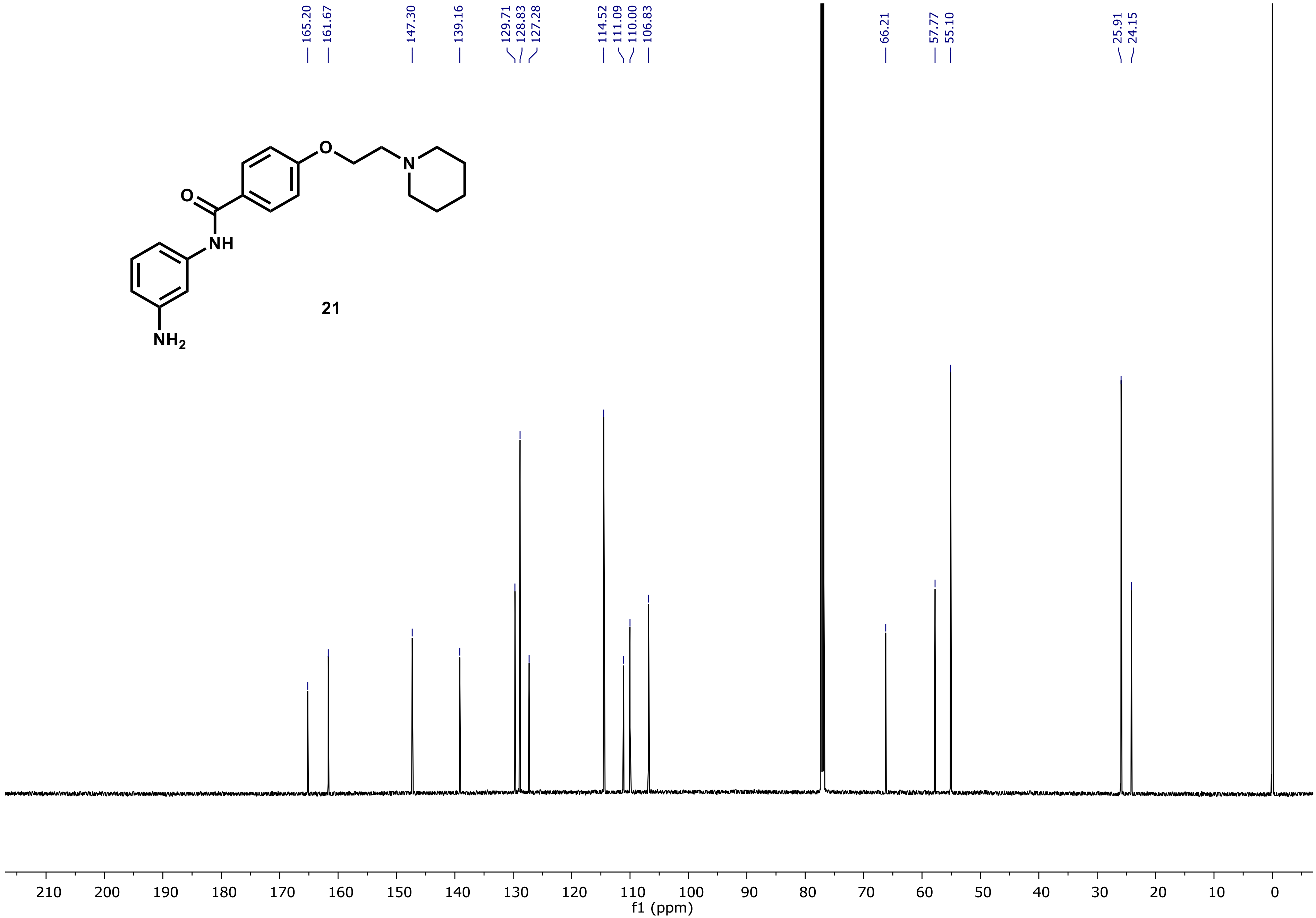

^{13}C NMR Spectrum for Compound **21** ($CDCl_3$, 151 MHz).

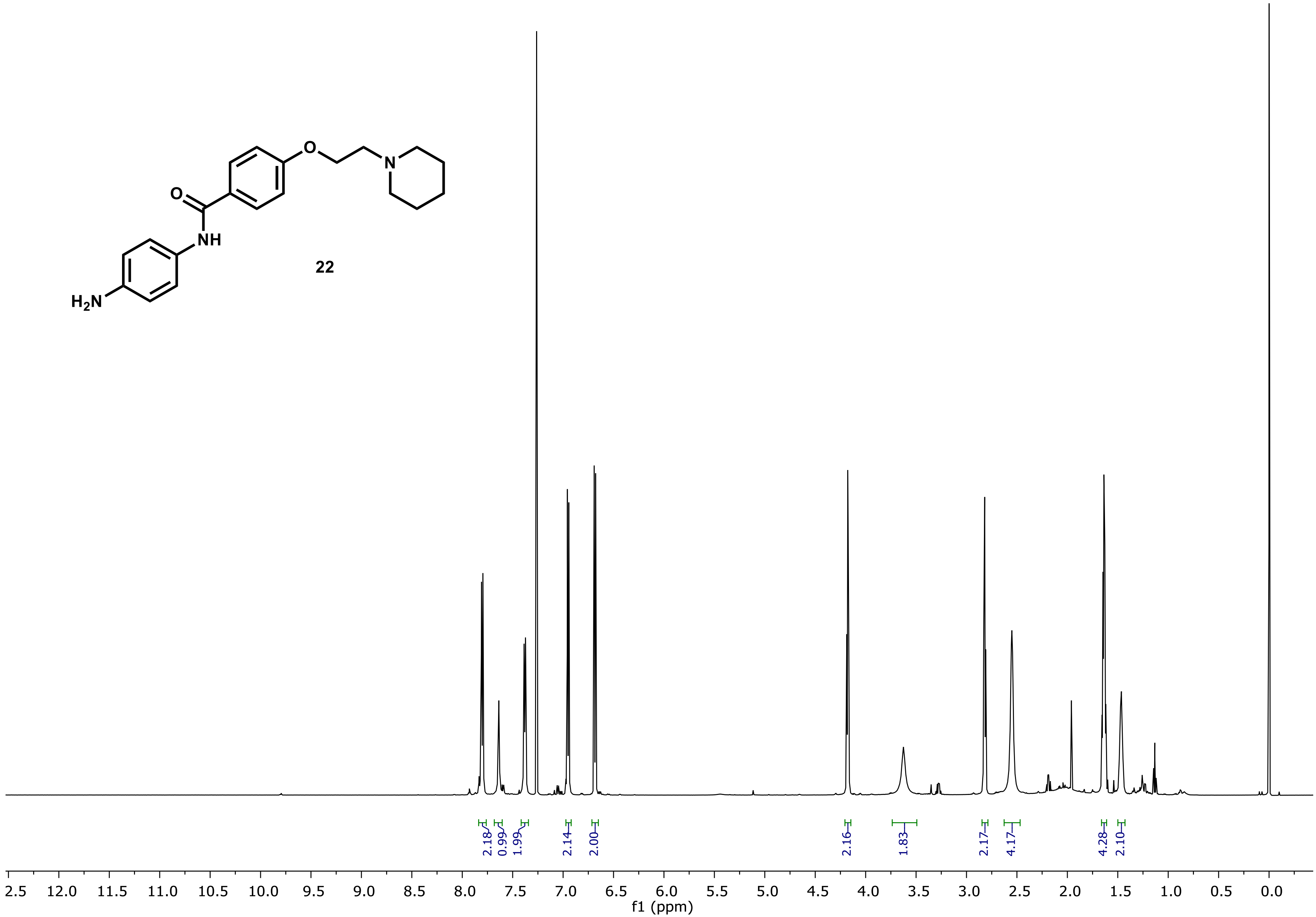

^{1}H NMR Spectrum for Compound **22** ($CDCl_3$, 600 MHz). Crude compound taken directly to next step without further purification.

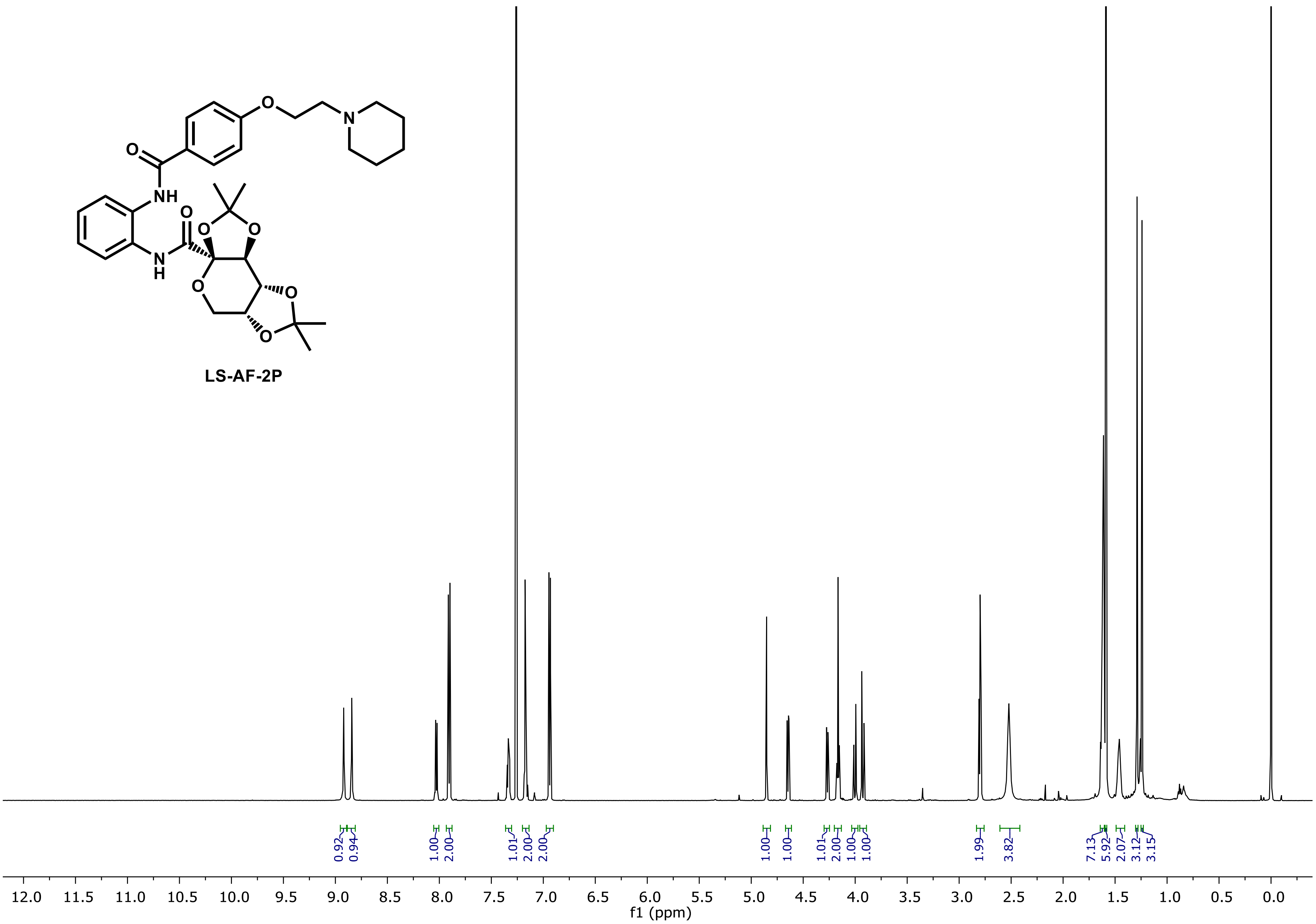

^{1}H NMR Spectrum for **LS-AF-2P** ($CDCl_3$, 600 MHz).

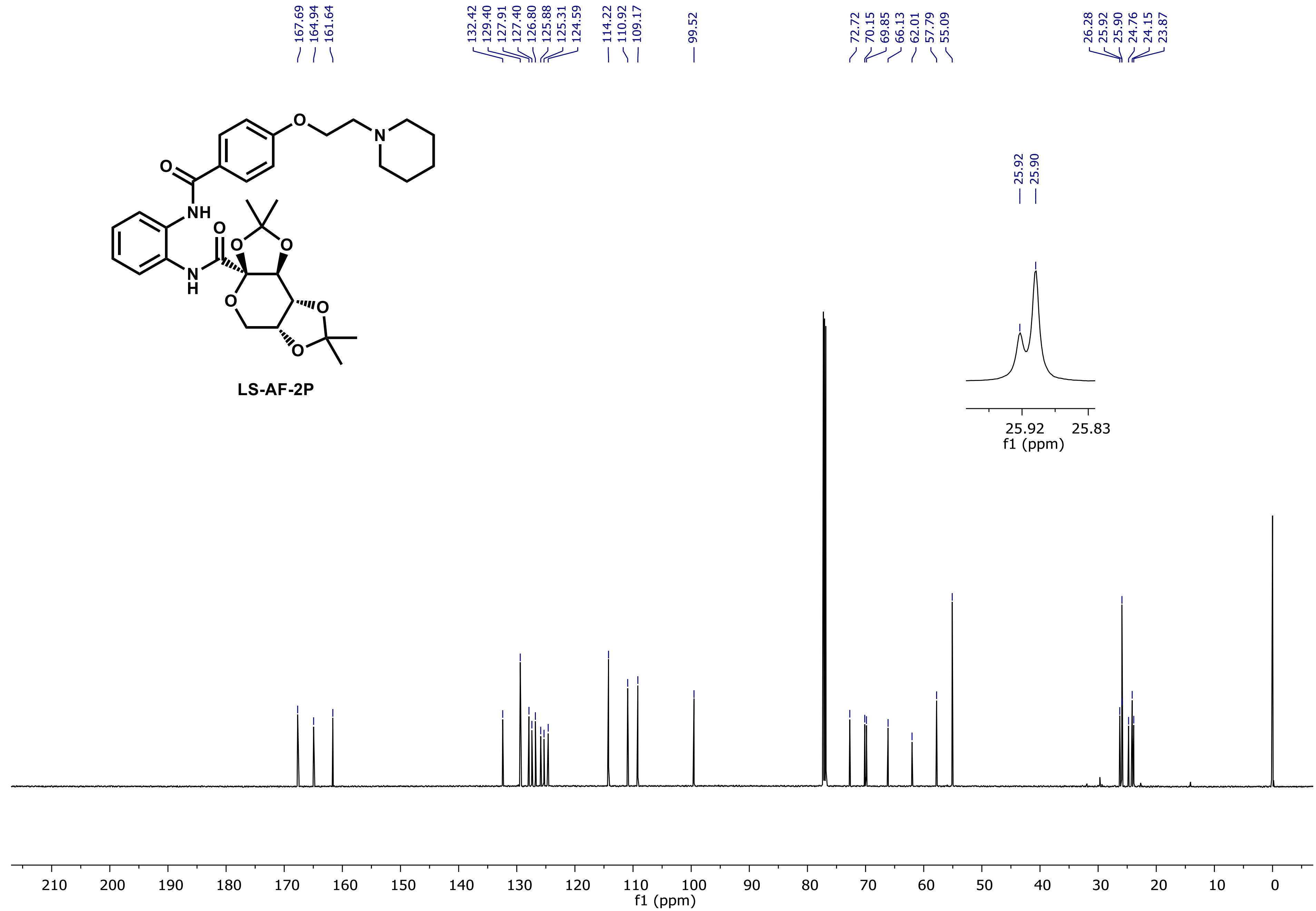

^{13}C NMR Spectrum for **LS-AF-2P** ($CDCl_3$, 151 MHz).

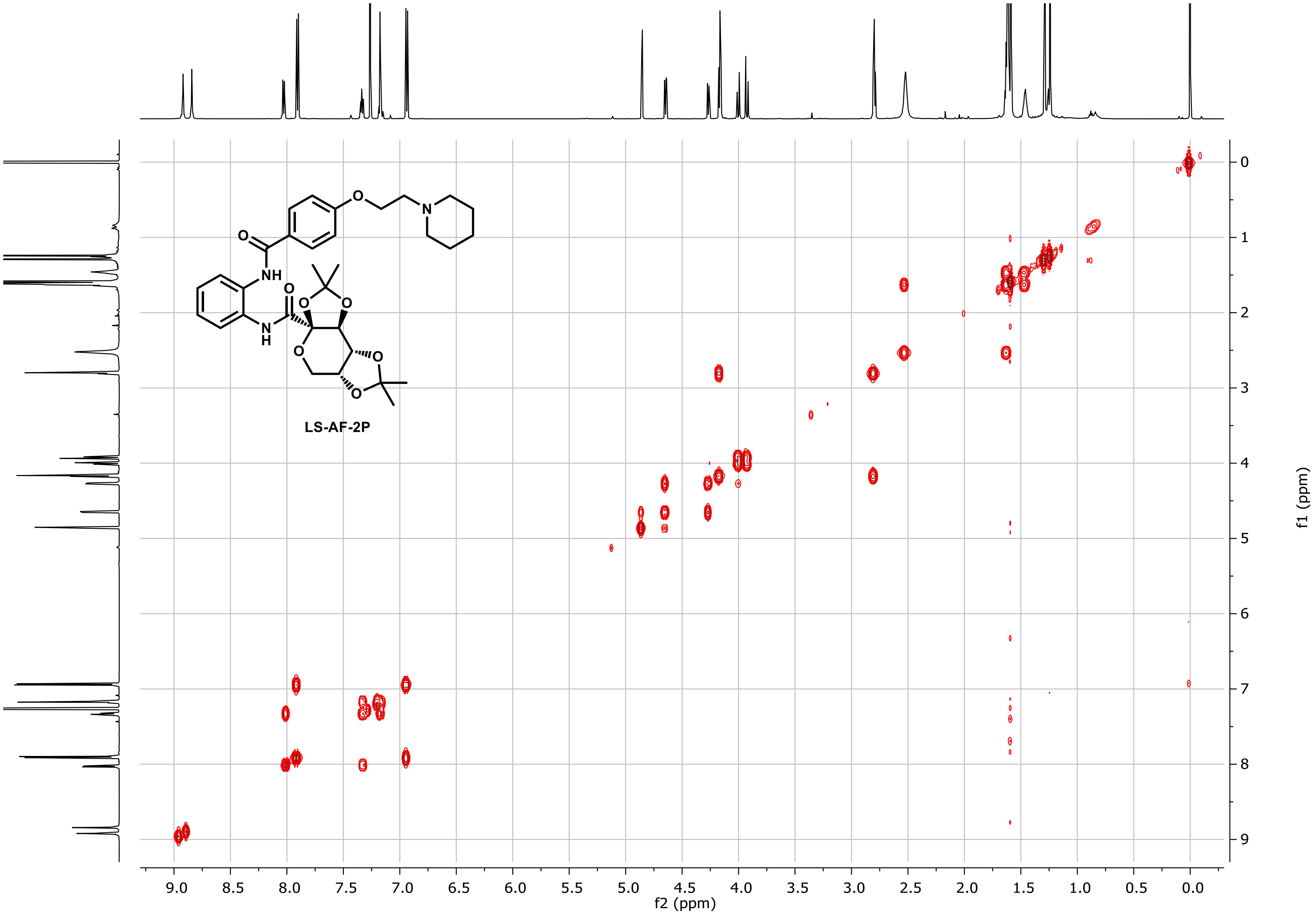

COSY Spectrum for **LS-AF-2P** ($CDCl_3$).

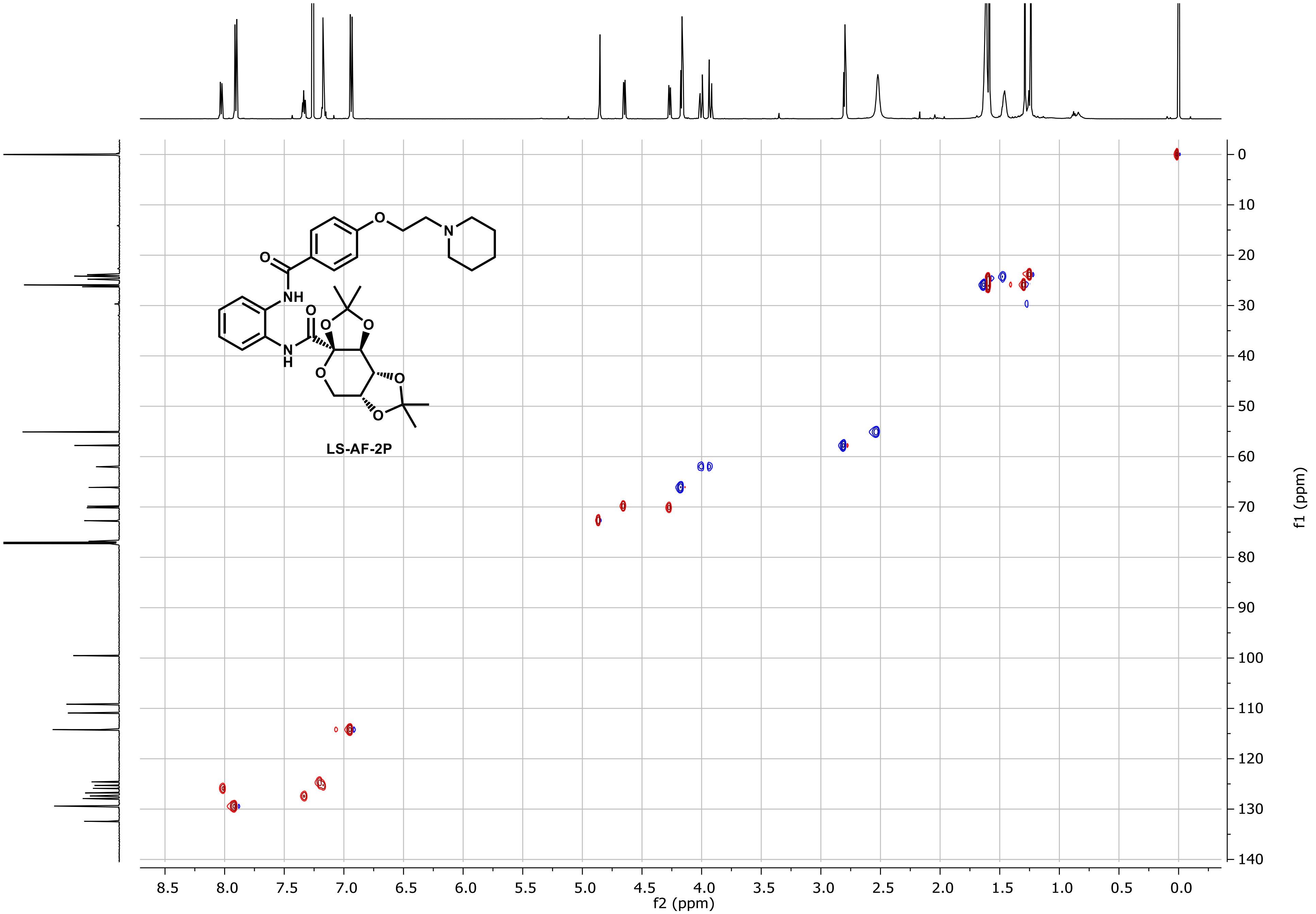

HSQC Spectrum for **LS-AF-2P** ($CDCl_3$).

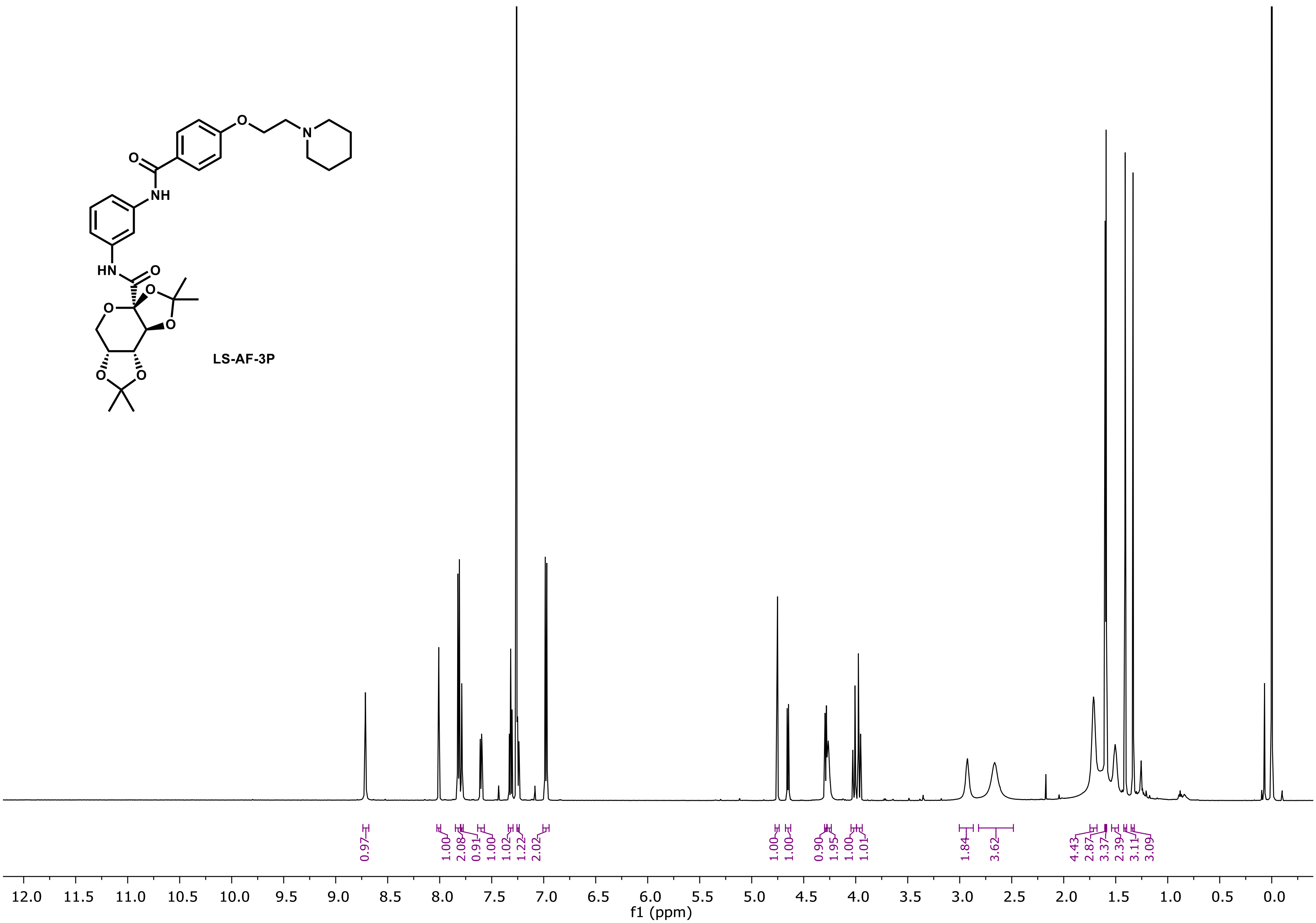

^{1}H NMR Spectrum for **LS-AF-3P** ($CDCl_3$, 600 MHz).

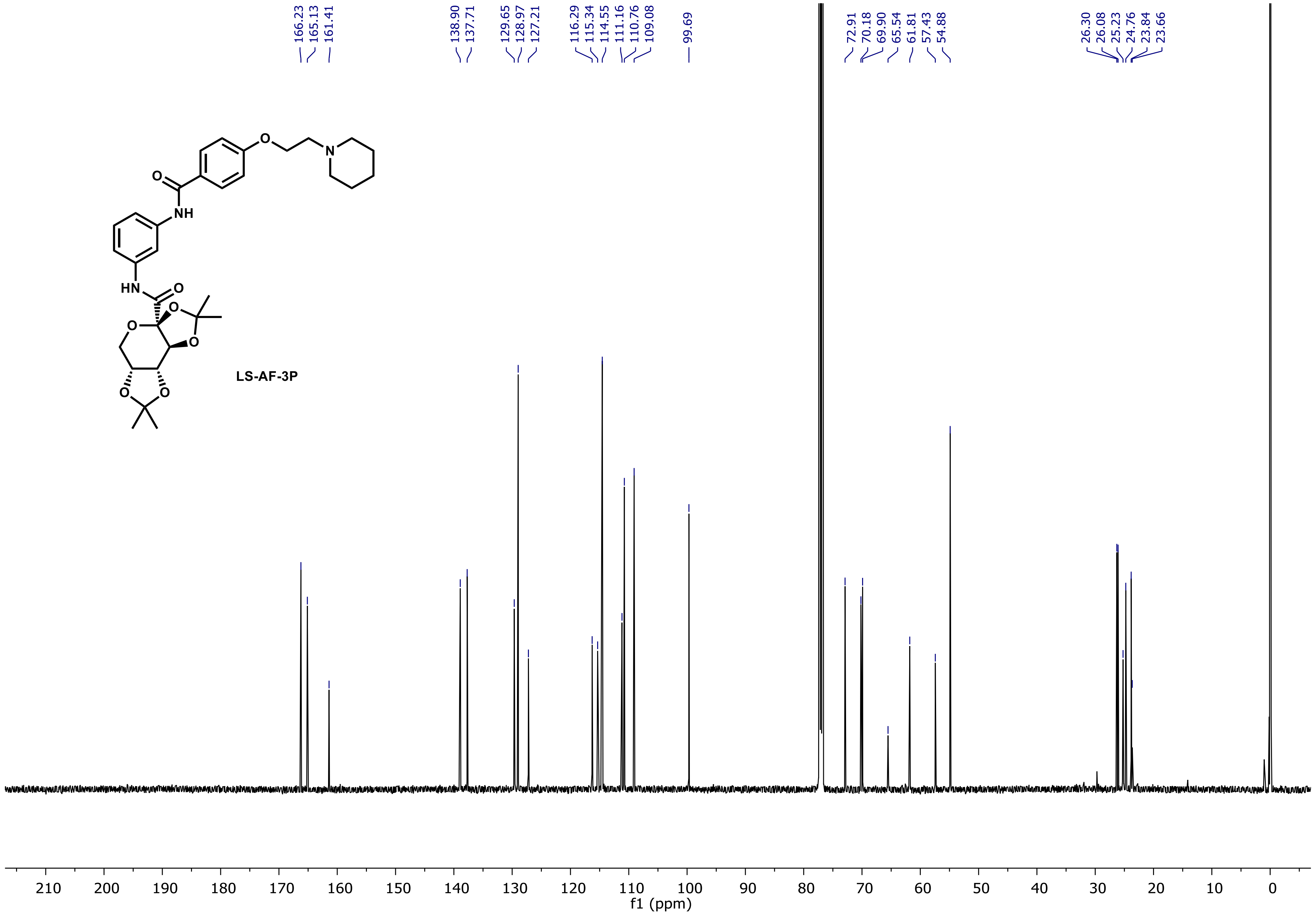

^{13}C NMR Spectrum for **LS-AF-3P** ($CDCl_3$, 151 MHz).

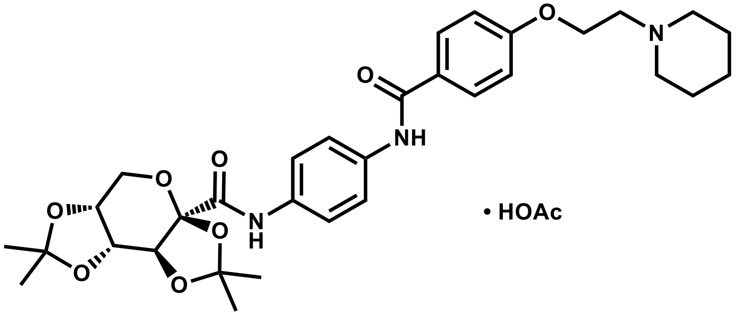

LS-AF-4P

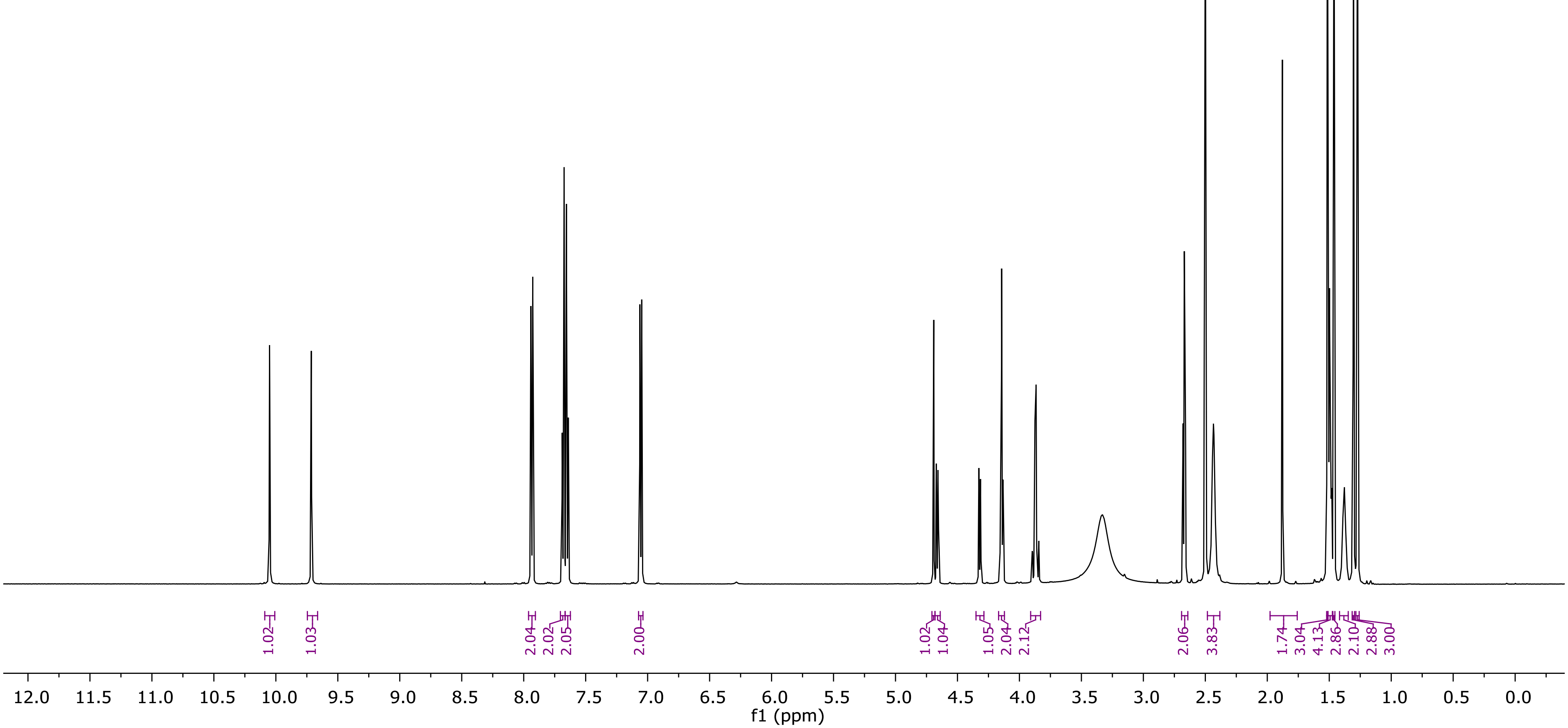

^{1}H NMR Spectrum for **LS-AF-4P** (DMSO-*d6*, 600 MHz).

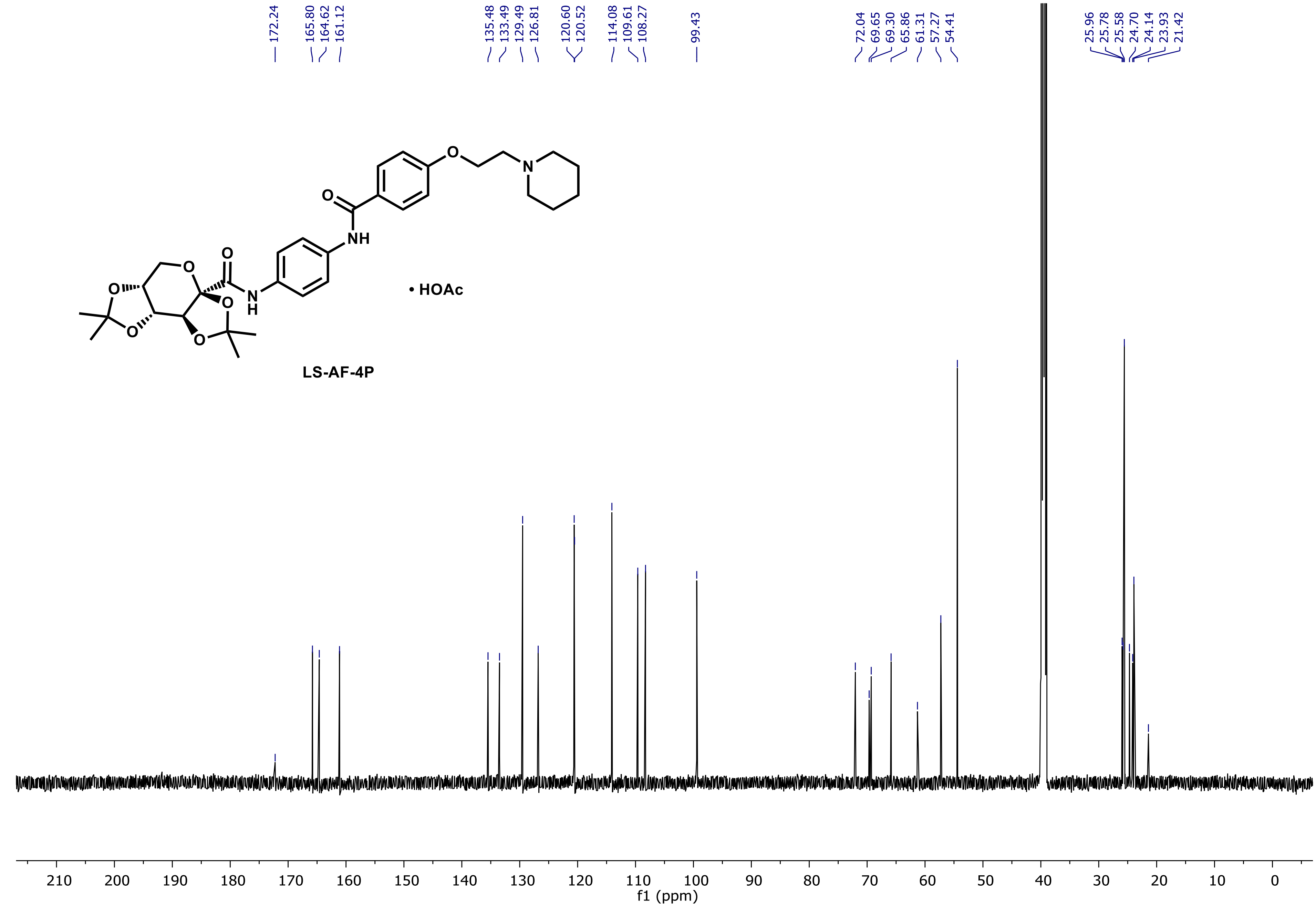

^{13}C NMR Spectrum for **LS-AF-4P** (DMSO-*d6*, 151 MHz).
